# Supplementary material for: Modelling the distribution of Mustela nivalis and M. putorius in the Azores archipelago based on native and introduced ranges
Source: PLoS One. 2020 Aug 7;15(8):e0237216. doi: 10.1371/journal.pone.0237216 (PMC7413552; doi:10.1371/journal.pone.0237216)
Supplement: S1 File — (DOCX) [file pone.0237216.s001.docx]

**S1 File**. Selection of background points selected to model the native area conditions.

Presence records obtained from open access data may be affected by sampling bias, because they are often spatially biased towards easily accessed areas [1, 2], they reflect several sampling campaigns with many different observers, detectability and experience. This spatial bias can lead to misleading results regarding the environmental factors behind the geographical distribution of a species, because this presence data is contrasted with background data usually drawn at random from the entire region [2]. Phillips et al. [2], proposed to select background data that exhibits the same bias as the presence data. For example, if the presence data are taken from a determined portion of the study area, then the background data should be taken from the same areas [2, 3]. Following this reasoning, we (i) created a grid comprising all native area (i.e., Europe), (ii) randomly selected 10,000 cells within <10 km of the species presence cells, and (iii) used 3,000 random background points from this selection to run the native-based models in MaxEnt. Figure S1 shows the geographical distribution of the background locations biased in the same way as the presence records.


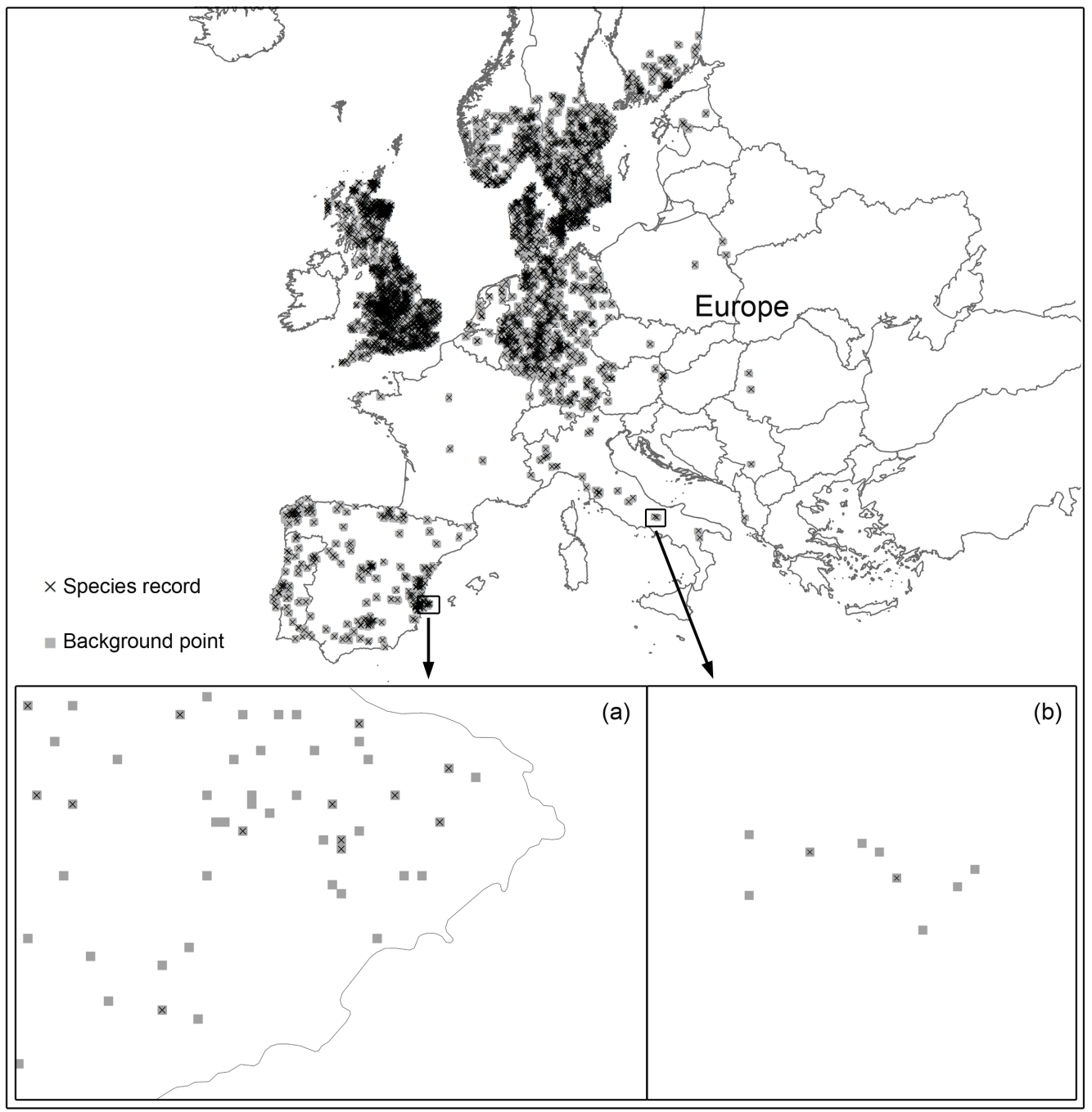


**Fig S1.** Distribution of background points exhibiting the same spatial bias than presence data (a) in areas with high density of presence data and (b) in areas with low density of presence data.

**References**

1. Hortal J, Jiménez‐Valverde A, Gómez JF, Lobo JM, Baselga A. Historical bias in biodiversity inventories affects the observed environmental niche of the species. Oikos. 2008; 117(6): 847−858.
2. Phillips SJ, Dudík M, Elith J, Graham CH, Lehmann A, Leathwick J, Ferrier S. Sample selection bias and presence‐only distribution models: implications for background and pseudo‐absence data. Ecol Appl. 2009; 19(1): 181−197.
3. Ferrier S, Watson G, Pearce J, Drielsma M. Extended statistical approaches to modelling spatial pattern in biodiversity in northeast New South Wales. I. Species-level modelling. Biodivers Conserv. 2002; 11(12): 2275−2307.
