# Supplementary material for: Modelling the distribution of Mustela nivalis and M. putorius in the Azores archipelago based on native and introduced ranges
Source: PLoS One. 2020 Aug 7;15(8):e0237216. doi: 10.1371/journal.pone.0237216 (PMC7413552; doi:10.1371/journal.pone.0237216)
Supplement: S2 File — (DOCX) [file pone.0237216.s002.docx]

**S2 File**. Occurrence records of *Mustela nivalis* and *M. putorius*.

Coordinates, in decimal degrees, of the occurrence records of *Mustela nivalis* and *M. putorius* used to perform the models. The native range include records of Europe, and the introduced range include records of Azores archipelago (Portugal). Additionally, we included the record source, i.e., obtained from open source databases (Open Source) or from sampling campaigns and/or visual observations (Field Data).

| **Species** | **Range** | **Latitude** | **Longitude** | **Record Type** |
| --- | --- | --- | --- | --- |
| *Mustela nivalis* | Native | 37.601022 | -7.685623 | Open Source |
| *Mustela nivalis* | Native | 41.094609 | -8.559251 | Open Source |
| *Mustela nivalis* | Native | 41.16666 | -8.688768999 | Open Source |
| *Mustela nivalis* | Native | 41.765034 | -8.188741 | Open Source |
| *Mustela nivalis* | Native | 42.863475 | -8.545842 | Field Data |
| *Mustela nivalis* | Native | 42.423281 | -5.219963999 | Open Source |
| *Mustela nivalis* | Native | 42.723825 | 0.113336 | Open Source |
| *Mustela nivalis* | Native | 41.585903 | 0.625674 | Open Source |
| *Mustela nivalis* | Native | 40.546283 | -1.654923 | Open Source |
| *Mustela nivalis* | Native | 37.01609 | -3.603022 | Open Source |
| *Mustela nivalis* | Native | 42.89 | -2.11 | Open Source |
| *Mustela nivalis* | Native | 42.978897 | -1.411211 | Open Source |
| *Mustela nivalis* | Native | 42.758892 | -1.411207001 | Open Source |
| *Mustela nivalis* | Native | 42.958897 | -1.401211001 | Open Source |
| *Mustela nivalis* | Native | 42.83 | -1.72 | Open Source |
| *Mustela nivalis* | Native | 39.526 | -8.693 | Open Source |
| *Mustela nivalis* | Native | 41.018 | -6.969 | Open Source |
| *Mustela nivalis* | Native | 40.96 | -6.933 | Open Source |
| *Mustela nivalis* | Native | 39.654 | -8.758 | Open Source |
| *Mustela nivalis* | Native | 39.654 | -8.757 | Open Source |
| *Mustela nivalis* | Native | 40.874 | -7.044 | Open Source |
| *Mustela nivalis* | Native | 40.599 | -7.753 | Open Source |
| *Mustela nivalis* | Native | 39.541 | -8.808 | Open Source |
| *Mustela nivalis* | Native | 40.864 | -6.843 | Open Source |
| *Mustela nivalis* | Native | 40.864 | -6.843 | Open Source |
| *Mustela nivalis* | Native | 40.938 | -7.085 | Open Source |
| *Mustela nivalis* | Native | 38.936 | -8.522 | Open Source |
| *Mustela nivalis* | Native | 38.849 | -7.351000001 | Open Source |
| *Mustela nivalis* | Native | 40.688 | -7.004999999 | Open Source |
| *Mustela nivalis* | Native | 37.191 | -7.451 | Open Source |
| *Mustela nivalis* | Native | 40.152 | -7.911 | Open Source |
| *Mustela nivalis* | Native | 37.087 | -7.881 | Open Source |
| *Mustela nivalis* | Native | 40.462 | -7.359 | Open Source |
| *Mustela nivalis* | Native | 40.456 | -7.365 | Open Source |
| *Mustela nivalis* | Native | 39.508 | -8.789 | Open Source |
| *Mustela nivalis* | Native | 39.55 | -8.797 | Open Source |
| *Mustela nivalis* | Native | 40.178 | -8.689 | Open Source |
| *Mustela nivalis* | Native | 40.405 | -7.899 | Open Source |
| *Mustela nivalis* | Native | 41.331 | -7.958 | Open Source |
| *Mustela nivalis* | Native | 39.209 | -9.001 | Open Source |
| *Mustela nivalis* | Native | 39.492 | -8.857 | Open Source |
| *Mustela nivalis* | Native | 40.933 | -8.17 | Open Source |
| *Mustela nivalis* | Native | 39.468 | -8.874 | Open Source |
| *Mustela nivalis* | Native | 39.542 | -8.794 | Open Source |
| *Mustela nivalis* | Native | 39.49 | -8.74 | Open Source |
| *Mustela nivalis* | Native | 38.963 | -9.303 | Open Source |
| *Mustela nivalis* | Native | 41.105 | -8.547 | Open Source |
| *Mustela nivalis* | Native | 42.586359 | -8.947801 | Field Data |
| *Mustela nivalis* | Native | 42.873172 | -8.531293 | Field Data |
| *Mustela nivalis* | Native | 42.810508 | -8.771061 | Field Data |
| *Mustela nivalis* | Native | 42.810308 | -8.772299 | Field Data |
| *Mustela nivalis* | Native | 42.686895 | -8.21889 | Field Data |
| *Mustela nivalis* | Native | 41.874666 | -8.141965999 | Open Source |
| *Mustela nivalis* | Native | 42.574557 | -8.14013 | Field Data |
| *Mustela nivalis* | Native | 42.448961 | -8.648569 | Open Source |
| *Mustela nivalis* | Native | 43.178758 | -7.599408 | Field Data |
| *Mustela nivalis* | Native | 42.87767 | -8.5656 | Field Data |
| *Mustela nivalis* | Native | 43.014778 | -8.655945 | Field Data |
| *Mustela nivalis* | Native | 43.12979 | -7.571931 | Field Data |
| *Mustela nivalis* | Native | 41.900005 | -8.078248001 | Open Source |
| *Mustela nivalis* | Native | 42.996427 | -8.526674 | Field Data |
| *Mustela nivalis* | Native | 42.894672 | -8.473665 | Field Data |
| *Mustela nivalis* | Native | 58.653108 | 5.588339 | Open Source |
| *Mustela nivalis* | Native | 59.663287 | 6.375739 | Open Source |
| *Mustela nivalis* | Native | 60.522885 | 7.963277999 | Open Source |
| *Mustela nivalis* | Native | 52.569202 | 10.121498 | Open Source |
| *Mustela nivalis* | Native | 58.224403 | 8.084406 | Open Source |
| *Mustela nivalis* | Native | 59.961413 | 11.06251 | Open Source |
| *Mustela nivalis* | Native | 61.10096 | 9.492272999 | Open Source |
| *Mustela nivalis* | Native | 51.191986 | 6.725607 | Open Source |
| *Mustela nivalis* | Native | 53.861389 | 8.706598999 | Open Source |
| *Mustela nivalis* | Native | 51.190533 | 6.076083 | Open Source |
| *Mustela nivalis* | Native | 50.927069 | 4.321488 | Open Source |
| *Mustela nivalis* | Native | 44.439275 | 6.9904 | Open Source |
| *Mustela nivalis* | Native | 50.93832 | 0.539189999 | Open Source |
| *Mustela nivalis* | Native | 49.176468 | 8.757902 | Open Source |
| *Mustela nivalis* | Native | 60.153377 | 15.188948 | Open Source |
| *Mustela nivalis* | Native | 42.999515 | -7.544121 | Field Data |
| *Mustela nivalis* | Native | 43.709549 | -7.558168 | Open Source |
| *Mustela nivalis* | Native | 49.175533 | 9.127481 | Open Source |
| *Mustela nivalis* | Native | 50.427032 | 8.226854 | Open Source |
| *Mustela nivalis* | Native | 50.229198 | 10.324488 | Open Source |
| *Mustela nivalis* | Native | 54.244267 | 10.841746 | Open Source |
| *Mustela nivalis* | Native | 47.766991 | 16.860319 | Open Source |
| *Mustela nivalis* | Native | 58.357585 | 7.684018 | Open Source |
| *Mustela nivalis* | Native | 57.553495 | 12.877152 | Open Source |
| *Mustela nivalis* | Native | 58.602241 | 12.481624 | Open Source |
| *Mustela nivalis* | Native | 52.982964 | 10.58773 | Open Source |
| *Mustela nivalis* | Native | 51.287903 | 6.383014 | Open Source |
| *Mustela nivalis* | Native | 49.753735 | 7.612863999 | Open Source |
| *Mustela nivalis* | Native | 51.325203 | 6.250377 | Open Source |
| *Mustela nivalis* | Native | 45.996482 | 1.815476 | Open Source |
| *Mustela nivalis* | Native | 51.26082 | 5.325038 | Open Source |
| *Mustela nivalis* | Native | 40.774369 | -8.669072 | Open Source |
| *Mustela nivalis* | Native | 58.75278 | 17.010031 | Open Source |
| *Mustela nivalis* | Native | 53.689999 | 8.740654 | Open Source |
| *Mustela nivalis* | Native | 47.785191 | 16.941605 | Open Source |
| *Mustela nivalis* | Native | 49.7658 | 8.576889 | Open Source |
| *Mustela nivalis* | Native | 51.883148 | 9.471270001 | Open Source |
| *Mustela nivalis* | Native | 56.673911 | 16.233177 | Open Source |
| *Mustela nivalis* | Native | 60.479147 | 5.304245999 | Open Source |
| *Mustela nivalis* | Native | 57.832605 | 13.057803 | Open Source |
| *Mustela nivalis* | Native | 60.140284 | 16.13633 | Open Source |
| *Mustela nivalis* | Native | 42.654726 | 13.161951 | Open Source |
| *Mustela nivalis* | Native | 48.830193 | 12.952377 | Open Source |
| *Mustela nivalis* | Native | 57.53308 | -6.588019999 | Open Source |
| *Mustela nivalis* | Native | 57.47161 | -6.54691 | Open Source |
| *Mustela nivalis* | Native | 57.53308 | -6.588019999 | Open Source |
| *Mustela nivalis* | Native | 57.52473 | -6.57025 | Open Source |
| *Mustela nivalis* | Native | 57.53308 | -6.588019999 | Open Source |
| *Mustela nivalis* | Native | 57.53308 | -6.588019999 | Open Source |
| *Mustela nivalis* | Native | 57.47161 | -6.54691 | Open Source |
| *Mustela nivalis* | Native | 57.40057 | -6.52143 | Open Source |
| *Mustela nivalis* | Native | 57.17521 | -2.409319999 | Open Source |
| *Mustela nivalis* | Native | 57.09721 | -2.335530001 | Open Source |
| *Mustela nivalis* | Native | 52.94105 | -3.39196 | Open Source |
| *Mustela nivalis* | Native | 52.96004 | -3.355959999 | Open Source |
| *Mustela nivalis* | Native | 52.94105 | -3.39196 | Open Source |
| *Mustela nivalis* | Native | 52.94218 | -3.35663 | Open Source |
| *Mustela nivalis* | Native | 52.9471 | -3.39474 | Open Source |
| *Mustela nivalis* | Native | 52.95216 | -3.353849999 | Open Source |
| *Mustela nivalis* | Native | 52.89276 | -3.48859 | Open Source |
| *Mustela nivalis* | Native | 52.94309 | -3.39473 | Open Source |
| *Mustela nivalis* | Native | 52.94309 | -3.39473 | Open Source |
| *Mustela nivalis* | Native | 52.7369 | -3.77359 | Open Source |
| *Mustela nivalis* | Native | 52.58756 | -3.83841 | Open Source |
| *Mustela nivalis* | Native | 52.96833 | -3.35436 | Open Source |
| *Mustela nivalis* | Native | 56.24563 | -5.528139999 | Open Source |
| *Mustela nivalis* | Native | 56.20976 | -5.524840001 | Open Source |
| *Mustela nivalis* | Native | 51.49561 | -2.44074 | Open Source |
| *Mustela nivalis* | Native | 53.13241 | -1.75484 | Open Source |
| *Mustela nivalis* | Native | 50.75825 | -1.55478 | Open Source |
| *Mustela nivalis* | Native | 55.71191 | -2.073179999 | Open Source |
| *Mustela nivalis* | Native | 52.00869 | -1.761020001 | Open Source |
| *Mustela nivalis* | Native | 53.20333 | -1.45502 | Open Source |
| *Mustela nivalis* | Native | 52.70804 | -3.01752 | Open Source |
| *Mustela nivalis* | Native | 52.384 | -2.77277 | Open Source |
| *Mustela nivalis* | Native | 52.35277 | -2.659910001 | Open Source |
| *Mustela nivalis* | Native | 52.68459 | -2.448919999 | Open Source |
| *Mustela nivalis* | Native | 52.88685 | -3.027649999 | Open Source |
| *Mustela nivalis* | Native | 52.84125 | -2.46813 | Open Source |
| *Mustela nivalis* | Native | 52.83676 | -3.100689999 | Open Source |
| *Mustela nivalis* | Native | 52.94264 | -2.544589999 | Open Source |
| *Mustela nivalis* | Native | 52.88009 | -3.08099 | Open Source |
| *Mustela nivalis* | Native | 52.86406 | -2.55601 | Open Source |
| *Mustela nivalis* | Native | 52.62249 | -2.45693 | Open Source |
| *Mustela nivalis* | Native | 52.72585 | -2.66108 | Open Source |
| *Mustela nivalis* | Native | 52.64936 | -2.4929 | Open Source |
| *Mustela nivalis* | Native | 52.914 | -2.76656 | Open Source |
| *Mustela nivalis* | Native | 52.68844 | -2.65571 | Open Source |
| *Mustela nivalis* | Native | 52.68347 | -2.82464 | Open Source |
| *Mustela nivalis* | Native | 52.94264 | -2.544589999 | Open Source |
| *Mustela nivalis* | Native | 52.70255 | -3.02923 | Open Source |
| *Mustela nivalis* | Native | 52.83775 | -2.987889999 | Open Source |
| *Mustela nivalis* | Native | 52.69391 | -2.580700001 | Open Source |
| *Mustela nivalis* | Native | 52.32944 | -2.57349 | Open Source |
| *Mustela nivalis* | Native | 52.66933 | -2.44433 | Open Source |
| *Mustela nivalis* | Native | 52.77205 | -2.7522 | Open Source |
| *Mustela nivalis* | Native | 52.7238 | -2.703989999 | Open Source |
| *Mustela nivalis* | Native | 52.91807 | -2.59731 | Open Source |
| *Mustela nivalis* | Native | 52.61077 | -2.92961 | Open Source |
| *Mustela nivalis* | Native | 52.69365 | -2.631 | Open Source |
| *Mustela nivalis* | Native | 52.53348 | -2.46949 | Open Source |
| *Mustela nivalis* | Native | 52.80649 | -3.06729 | Open Source |
| *Mustela nivalis* | Native | 52.54963 | -2.80886 | Open Source |
| *Mustela nivalis* | Native | 52.70738 | -2.53655 | Open Source |
| *Mustela nivalis* | Native | 52.80031 | -2.94848 | Open Source |
| *Mustela nivalis* | Native | 52.46029 | -3.170760001 | Open Source |
| *Mustela nivalis* | Native | 52.69161 | -2.93872 | Open Source |
| *Mustela nivalis* | Native | 52.61886 | -2.66242 | Open Source |
| *Mustela nivalis* | Native | 52.67115 | -2.636600001 | Open Source |
| *Mustela nivalis* | Native | 52.66634 | -2.69272 | Open Source |
| *Mustela nivalis* | Native | 52.41895 | -2.965339999 | Open Source |
| *Mustela nivalis* | Native | 52.72055 | -2.64472 | Open Source |
| *Mustela nivalis* | Native | 52.89563 | -2.94461 | Open Source |
| *Mustela nivalis* | Native | 52.54618 | -2.71663 | Open Source |
| *Mustela nivalis* | Native | 52.54963 | -2.80886 | Open Source |
| *Mustela nivalis* | Native | 52.79728 | -2.92817 | Open Source |
| *Mustela nivalis* | Native | 52.58065 | -3.08836 | Open Source |
| *Mustela nivalis* | Native | 52.72349 | -2.842200001 | Open Source |
| *Mustela nivalis* | Native | 52.94264 | -2.544589999 | Open Source |
| *Mustela nivalis* | Native | 52.80824 | -2.43907 | Open Source |
| *Mustela nivalis* | Native | 52.64193 | -2.7145 | Open Source |
| *Mustela nivalis* | Native | 52.55928 | -2.96097 | Open Source |
| *Mustela nivalis* | Native | 52.49064 | -2.99416 | Open Source |
| *Mustela nivalis* | Native | 52.89847 | -2.80345 | Open Source |
| *Mustela nivalis* | Native | 52.40325 | -3.020779999 | Open Source |
| *Mustela nivalis* | Native | 52.76681 | -2.88009 | Open Source |
| *Mustela nivalis* | Native | 52.53756 | -2.86319 | Open Source |
| *Mustela nivalis* | Native | 52.94264 | -2.544589999 | Open Source |
| *Mustela nivalis* | Native | 52.96255 | -2.39671 | Open Source |
| *Mustela nivalis* | Native | 52.94264 | -2.544589999 | Open Source |
| *Mustela nivalis* | Native | 52.57094 | -2.53924 | Open Source |
| *Mustela nivalis* | Native | 52.77856 | -2.91317 | Open Source |
| *Mustela nivalis* | Native | 52.91088 | -2.510739999 | Open Source |
| *Mustela nivalis* | Native | 52.70284 | -2.9952 | Open Source |
| *Mustela nivalis* | Native | 52.7537 | -2.90176 | Open Source |
| *Mustela nivalis* | Native | 52.50885 | -2.971660001 | Open Source |
| *Mustela nivalis* | Native | 52.65272 | -2.714670001 | Open Source |
| *Mustela nivalis* | Native | 52.47448 | -2.37758 | Open Source |
| *Mustela nivalis* | Native | 52.75224 | -3.00077 | Open Source |
| *Mustela nivalis* | Native | 59.327516 | 9.818892001 | Open Source |
| *Mustela nivalis* | Native | 59.868812 | 10.828275 | Open Source |
| *Mustela nivalis* | Native | 59.372222 | 7.789795 | Open Source |
| *Mustela nivalis* | Native | 60.723482 | 8.412902001 | Open Source |
| *Mustela nivalis* | Native | 58.827881 | 6.28385 | Open Source |
| *Mustela nivalis* | Native | 59.369325 | 10.704693 | Open Source |
| *Mustela nivalis* | Native | 60.040337 | 11.709189 | Open Source |
| *Mustela nivalis* | Native | 57.019674 | 14.559175 | Open Source |
| *Mustela nivalis* | Native | 56.055284 | 14.139447 | Open Source |
| *Mustela nivalis* | Native | 56.625973 | 12.920316 | Open Source |
| *Mustela nivalis* | Native | 59.17473 | 17.53144 | Open Source |
| *Mustela nivalis* | Native | 55.40032 | 13.595804 | Open Source |
| *Mustela nivalis* | Native | 56.988302 | 16.884096 | Open Source |
| *Mustela nivalis* | Native | 57.698079 | 12.053981 | Open Source |
| *Mustela nivalis* | Native | 59.695114 | 16.108626 | Open Source |
| *Mustela nivalis* | Native | 57.891684 | 16.459732 | Open Source |
| *Mustela nivalis* | Native | 52.361233 | 13.810324 | Open Source |
| *Mustela nivalis* | Native | 47.596626 | 9.561962001 | Open Source |
| *Mustela nivalis* | Native | 49.98843 | 9.69595 | Open Source |
| *Mustela nivalis* | Native | 51.565353 | 14.614024 | Open Source |
| *Mustela nivalis* | Native | 53.667103 | 10.093496 | Open Source |
| *Mustela nivalis* | Native | 51.341496 | 6.218625999 | Open Source |
| *Mustela nivalis* | Native | 50.42717 | 8.199925 | Open Source |
| *Mustela nivalis* | Native | 48.905296 | 10.15965 | Open Source |
| *Mustela nivalis* | Native | 58.368116 | 8.586439 | Open Source |
| *Mustela nivalis* | Native | 60.125615 | 10.017437 | Open Source |
| *Mustela nivalis* | Native | 58.999704 | 6.907470001 | Open Source |
| *Mustela nivalis* | Native | 59.144746 | 7.165807 | Open Source |
| *Mustela nivalis* | Native | 52.283277 | 7.727904999 | Open Source |
| *Mustela nivalis* | Native | 54.472759 | 9.778661 | Open Source |
| *Mustela nivalis* | Native | 49.772514 | 7.633307 | Open Source |
| *Mustela nivalis* | Native | 50.390656 | 8.103704 | Open Source |
| *Mustela nivalis* | Native | 53.561432 | 10.911845 | Open Source |
| *Mustela nivalis* | Native | 49.298557 | 8.113596 | Open Source |
| *Mustela nivalis* | Native | 48.790722 | 9.494033 | Open Source |
| *Mustela nivalis* | Native | 52.401737 | 13.369331 | Open Source |
| *Mustela nivalis* | Native | 57.533658 | 12.098223 | Open Source |
| *Mustela nivalis* | Native | 56.036931 | 13.247384 | Open Source |
| *Mustela nivalis* | Native | 57.707674 | 11.788367 | Open Source |
| *Mustela nivalis* | Native | 57.521262 | 12.022216 | Open Source |
| *Mustela nivalis* | Native | 51.218716 | 9.260966 | Open Source |
| *Mustela nivalis* | Native | 58.197949 | 15.643137 | Open Source |
| *Mustela nivalis* | Native | 57.952486 | 12.475282 | Open Source |
| *Mustela nivalis* | Native | 56.259654 | 16.475668 | Open Source |
| *Mustela nivalis* | Native | 57.698895 | 12.053603 | Open Source |
| *Mustela nivalis* | Native | 56.267751 | 16.032296 | Open Source |
| *Mustela nivalis* | Native | 56.076672 | 14.363693 | Open Source |
| *Mustela nivalis* | Native | 60.364297 | 6.405482001 | Open Source |
| *Mustela nivalis* | Native | 60.523504 | 8.123087999 | Open Source |
| *Mustela nivalis* | Native | 61.390348 | 10.249041 | Open Source |
| *Mustela nivalis* | Native | 59.877694 | 11.202351 | Open Source |
| *Mustela nivalis* | Native | 59.877383 | 11.202171 | Open Source |
| *Mustela nivalis* | Native | 61.027368 | 9.078401 | Open Source |
| *Mustela nivalis* | Native | 59.99638 | 10.76488 | Open Source |
| *Mustela nivalis* | Native | 59.378098 | 13.640558 | Open Source |
| *Mustela nivalis* | Native | 57.846907 | 11.909891 | Open Source |
| *Mustela nivalis* | Native | 52.988232 | 14.19174 | Open Source |
| *Mustela nivalis* | Native | 53.689999 | 8.740654 | Open Source |
| *Mustela nivalis* | Native | 49.768261 | 8.447404001 | Open Source |
| *Mustela nivalis* | Native | 56.149422 | 15.361434 | Open Source |
| *Mustela nivalis* | Native | 56.003041 | 14.615644 | Open Source |
| *Mustela nivalis* | Native | 56.651671 | 16.601504 | Open Source |
| *Mustela nivalis* | Native | 57.483284 | 11.942994 | Open Source |
| *Mustela nivalis* | Native | 60.510718 | 5.327279 | Open Source |
| *Mustela nivalis* | Native | 59.667633 | 10.533304 | Open Source |
| *Mustela nivalis* | Native | 58.377306 | 6.89766 | Open Source |
| *Mustela nivalis* | Native | 58.376331 | 6.900202 | Open Source |
| *Mustela nivalis* | Native | 58.245452 | 7.156797 | Open Source |
| *Mustela nivalis* | Native | 59.45534 | 10.70402 | Open Source |
| *Mustela nivalis* | Native | 59.401659 | 10.658475 | Open Source |
| *Mustela nivalis* | Native | 60.005493 | 10.489565 | Open Source |
| *Mustela nivalis* | Native | 58.031197 | 7.510796 | Open Source |
| *Mustela nivalis* | Native | 60.005493 | 10.489565 | Open Source |
| *Mustela nivalis* | Native | 52.273632 | 14.373116 | Open Source |
| *Mustela nivalis* | Native | 52.273628 | 14.289795 | Open Source |
| *Mustela nivalis* | Native | 54.525364 | 9.867867 | Open Source |
| *Mustela nivalis* | Native | 59.683843 | 16.342053 | Open Source |
| *Mustela nivalis* | Native | 37.581865 | -6.088798 | Open Source |
| *Mustela nivalis* | Native | 51.336025 | 9.866071001 | Open Source |
| *Mustela nivalis* | Native | 53.824375 | 9.31741 | Open Source |
| *Mustela nivalis* | Native | 48.283451 | 11.908697 | Open Source |
| *Mustela nivalis* | Native | 49.686909 | 10.859337 | Open Source |
| *Mustela nivalis* | Native | 50.00087 | 8.869507 | Open Source |
| *Mustela nivalis* | Native | 53.724442 | 10.178318 | Open Source |
| *Mustela nivalis* | Native | 59.197789 | 17.844413 | Open Source |
| *Mustela nivalis* | Native | 56.710455 | 13.078554 | Open Source |
| *Mustela nivalis* | Native | 56.641694 | 16.265858 | Open Source |
| *Mustela nivalis* | Native | 56.609682 | 12.924987 | Open Source |
| *Mustela nivalis* | Native | 57.401769 | 14.083095 | Open Source |
| *Mustela nivalis* | Native | 55.82484 | 14.0713 | Open Source |
| *Mustela nivalis* | Native | 59.766839 | 16.189483 | Open Source |
| *Mustela nivalis* | Native | 57.809737 | 12.074337 | Open Source |
| *Mustela nivalis* | Native | 52.177879 | 21.305816 | Open Source |
| *Mustela nivalis* | Native | 57.689034 | 14.664961 | Open Source |
| *Mustela nivalis* | Native | 55.825673 | 14.071291 | Open Source |
| *Mustela nivalis* | Native | 58.356934 | 15.661525 | Open Source |
| *Mustela nivalis* | Native | 59.930501 | 17.33567 | Open Source |
| *Mustela nivalis* | Native | 59.835773 | 17.665828 | Open Source |
| *Mustela nivalis* | Native | 57.413227 | 14.103909 | Open Source |
| *Mustela nivalis* | Native | 59.183581 | 12.8337 | Open Source |
| *Mustela nivalis* | Native | 57.413227 | 14.103909 | Open Source |
| *Mustela nivalis* | Native | 49.898891 | 8.497281 | Open Source |
| *Mustela nivalis* | Native | 47.82988 | 10.723648 | Open Source |
| *Mustela nivalis* | Native | 54.143724 | 10.445884 | Open Source |
| *Mustela nivalis* | Native | 56.404554 | 12.622533 | Open Source |
| *Mustela nivalis* | Native | 57.253236 | 12.445368 | Open Source |
| *Mustela nivalis* | Native | 59.886714 | 16.083617 | Open Source |
| *Mustela nivalis* | Native | 56.785975 | 15.898914 | Open Source |
| *Mustela nivalis* | Native | 57.412086 | 14.106326 | Open Source |
| *Mustela nivalis* | Native | 57.273304 | 16.429827 | Open Source |
| *Mustela nivalis* | Native | 56.750027 | 14.908297 | Open Source |
| *Mustela nivalis* | Native | 58.973669 | 16.15514 | Open Source |
| *Mustela nivalis* | Native | 59.814045 | 17.661606 | Open Source |
| *Mustela nivalis* | Native | 57.413227 | 14.103909 | Open Source |
| *Mustela nivalis* | Native | 55.606163 | 13.767876 | Open Source |
| *Mustela nivalis* | Native | 57.406502 | 15.516016 | Open Source |
| *Mustela nivalis* | Native | 58.427259 | 16.146499 | Open Source |
| *Mustela nivalis* | Native | 59.822978 | 16.193975 | Open Source |
| *Mustela nivalis* | Native | 59.811548 | 16.161536 | Open Source |
| *Mustela nivalis* | Native | 57.412086 | 14.106326 | Open Source |
| *Mustela nivalis* | Native | 57.41331 | 14.105329 | Open Source |
| *Mustela nivalis* | Native | 57.772059 | 13.403798 | Open Source |
| *Mustela nivalis* | Native | 57.019498 | 12.339618 | Open Source |
| *Mustela nivalis* | Native | 57.414258 | 14.105957 | Open Source |
| *Mustela nivalis* | Native | 61.416185 | 14.546752 | Open Source |
| *Mustela nivalis* | Native | 56.611778 | 14.191675 | Open Source |
| *Mustela nivalis* | Native | 56.604204 | 14.172208 | Open Source |
| *Mustela nivalis* | Native | 59.11735 | 17.843713 | Open Source |
| *Mustela nivalis* | Native | 50.142559 | 10.72948 | Open Source |
| *Mustela nivalis* | Native | 50.234627 | 7.332287999 | Open Source |
| *Mustela nivalis* | Native | 50.802578 | 8.809598 | Open Source |
| *Mustela nivalis* | Native | 51.196018 | 6.735306 | Open Source |
| *Mustela nivalis* | Native | 58.477587 | 12.324661 | Open Source |
| *Mustela nivalis* | Native | 59.299556 | 15.209673 | Open Source |
| *Mustela nivalis* | Native | 57.413227 | 14.103909 | Open Source |
| *Mustela nivalis* | Native | 59.844732 | 16.201493 | Open Source |
| *Mustela nivalis* | Native | 59.893298 | 16.101026 | Open Source |
| *Mustela nivalis* | Native | 57.749872 | 12.173538 | Open Source |
| *Mustela nivalis* | Native | 40.729958 | -8.567534 | Open Source |
| *Mustela nivalis* | Native | 45.980672 | 11.091379 | Open Source |
| *Mustela nivalis* | Native | 46.013024 | 11.29776 | Open Source |
| *Mustela nivalis* | Native | 58.886888 | 6.885093 | Open Source |
| *Mustela nivalis* | Native | 58.898244 | 6.883772 | Open Source |
| *Mustela nivalis* | Native | 59.405368 | 10.441873 | Open Source |
| *Mustela nivalis* | Native | 59.34542 | 10.745764 | Open Source |
| *Mustela nivalis* | Native | 60.797874 | 11.797449 | Open Source |
| *Mustela nivalis* | Native | 58.125033 | 6.609157 | Open Source |
| *Mustela nivalis* | Native | 58.742765 | 6.796905001 | Open Source |
| *Mustela nivalis* | Native | 59.084449 | 10.097297 | Open Source |
| *Mustela nivalis* | Native | 58.186875 | 6.923243999 | Open Source |
| *Mustela nivalis* | Native | 59.374313 | 11.296584 | Open Source |
| *Mustela nivalis* | Native | 59.25419 | 9.507958001 | Open Source |
| *Mustela nivalis* | Native | 58.96042 | 11.491707 | Open Source |
| *Mustela nivalis* | Native | 58.900945 | 6.885281001 | Open Source |
| *Mustela nivalis* | Native | 59.941765 | 10.585846 | Open Source |
| *Mustela nivalis* | Native | 58.898244 | 6.883772 | Open Source |
| *Mustela nivalis* | Native | 58.535839 | 5.723103999 | Open Source |
| *Mustela nivalis* | Native | 59.575336 | 6.257709 | Open Source |
| *Mustela nivalis* | Native | 59.275877 | 10.349652 | Open Source |
| *Mustela nivalis* | Native | 58.16173 | 8.099336001 | Open Source |
| *Mustela nivalis* | Native | 59.061199 | 6.667907 | Open Source |
| *Mustela nivalis* | Native | 59.651125 | 10.535127 | Open Source |
| *Mustela nivalis* | Native | 60.005493 | 10.489565 | Open Source |
| *Mustela nivalis* | Native | 58.365722 | 6.890024 | Open Source |
| *Mustela nivalis* | Native | 61.255322 | 10.716776 | Open Source |
| *Mustela nivalis* | Native | 58.827867 | 6.283652999 | Open Source |
| *Mustela nivalis* | Native | 59.230659 | 11.004551 | Open Source |
| *Mustela nivalis* | Native | 57.922921 | 14.744426 | Open Source |
| *Mustela nivalis* | Native | 57.853963 | 14.081299 | Open Source |
| *Mustela nivalis* | Native | 59.639045 | 18.228066 | Open Source |
| *Mustela nivalis* | Native | 56.747101 | 16.648172 | Open Source |
| *Mustela nivalis* | Native | 59.24719 | 14.932587 | Open Source |
| *Mustela nivalis* | Native | 55.682823 | 13.279813 | Open Source |
| *Mustela nivalis* | Native | 57.772433 | 14.279278 | Open Source |
| *Mustela nivalis* | Native | 56.722469 | 13.213562 | Open Source |
| *Mustela nivalis* | Native | 58.583886 | 13.957322 | Open Source |
| *Mustela nivalis* | Native | 57.413227 | 14.103909 | Open Source |
| *Mustela nivalis* | Native | 58.464584 | 15.553521 | Open Source |
| *Mustela nivalis* | Native | 57.88251 | 14.845415 | Open Source |
| *Mustela nivalis* | Native | 57.703973 | 12.917361 | Open Source |
| *Mustela nivalis* | Native | 60.178301 | 17.780112 | Open Source |
| *Mustela nivalis* | Native | 61.530464 | 14.291487 | Open Source |
| *Mustela nivalis* | Native | 60.300487 | 16.890843 | Open Source |
| *Mustela nivalis* | Native | 58.767786 | 17.000994 | Open Source |
| *Mustela nivalis* | Native | 49.199234 | 8.174591 | Open Source |
| *Mustela nivalis* | Native | 51.831535 | 6.631923001 | Open Source |
| *Mustela nivalis* | Native | 51.752193 | 9.930621 | Open Source |
| *Mustela nivalis* | Native | 51.399422 | 9.752646 | Open Source |
| *Mustela nivalis* | Native | 61.648163 | 27.336731 | Open Source |
| *Mustela nivalis* | Native | 53.160717 | 8.292789 | Open Source |
| *Mustela nivalis* | Native | 49.545544 | 10.983457 | Open Source |
| *Mustela nivalis* | Native | 53.287792 | 6.651183001 | Open Source |
| *Mustela nivalis* | Native | 49.405724 | 8.505392 | Open Source |
| *Mustela nivalis* | Native | 50.93259 | 12.830894 | Open Source |
| *Mustela nivalis* | Native | 52.925003 | 11.105869 | Open Source |
| *Mustela nivalis* | Native | 50.935715 | 8.827686001 | Open Source |
| *Mustela nivalis* | Native | 51.572964 | 10.140123 | Open Source |
| *Mustela nivalis* | Native | 50.907475 | 6.607023 | Open Source |
| *Mustela nivalis* | Native | 48.758339 | 10.153556 | Open Source |
| *Mustela nivalis* | Native | 50.926517 | 7.066647 | Open Source |
| *Mustela nivalis* | Native | 50.497665 | 8.661834 | Open Source |
| *Mustela nivalis* | Native | 51.183754 | 6.745155 | Open Source |
| *Mustela nivalis* | Native | 51.709599 | 10.534401 | Open Source |
| *Mustela nivalis* | Native | 51.706715 | 13.213205 | Open Source |
| *Mustela nivalis* | Native | 53.136757 | 9.186851999 | Open Source |
| *Mustela nivalis* | Native | 52.586273 | 11.981298 | Open Source |
| *Mustela nivalis* | Native | 50.19952 | 6.824574999 | Open Source |
| *Mustela nivalis* | Native | 48.385818 | 9.962137 | Open Source |
| *Mustela nivalis* | Native | 51.823883 | 6.738305 | Open Source |
| *Mustela nivalis* | Native | 53.498871 | 8.55217 | Open Source |
| *Mustela nivalis* | Native | 51.359669 | 9.054623 | Open Source |
| *Mustela nivalis* | Native | 50.636086 | 7.168075001 | Open Source |
| *Mustela nivalis* | Native | 52.001648 | 9.863997 | Open Source |
| *Mustela nivalis* | Native | 50.844887 | 8.967831999 | Open Source |
| *Mustela nivalis* | Native | 50.883743 | 12.891357 | Open Source |
| *Mustela nivalis* | Native | 49.810902 | 8.659594999 | Open Source |
| *Mustela nivalis* | Native | 54.882359 | 8.341438999 | Open Source |
| *Mustela nivalis* | Native | 52.140533 | 10.782352 | Open Source |
| *Mustela nivalis* | Native | 52.87286 | 8.830797 | Open Source |
| *Mustela nivalis* | Native | 50.386406 | 9.313695 | Open Source |
| *Mustela nivalis* | Native | 50.67775 | 7.139912 | Open Source |
| *Mustela nivalis* | Native | 53.598381 | 8.603437 | Open Source |
| *Mustela nivalis* | Native | 49.940975 | 6.680052 | Open Source |
| *Mustela nivalis* | Native | 50.631935 | 6.638961 | Open Source |
| *Mustela nivalis* | Native | 51.049187 | 7.625294999 | Open Source |
| *Mustela nivalis* | Native | 47.219421 | 11.100655 | Open Source |
| *Mustela nivalis* | Native | 49.539055 | 6.536503 | Open Source |
| *Mustela nivalis* | Native | 50.213051 | 6.991489001 | Open Source |
| *Mustela nivalis* | Native | 50.929684 | 6.762192 | Open Source |
| *Mustela nivalis* | Native | 50.002338 | 6.67546 | Open Source |
| *Mustela nivalis* | Native | 51.185665 | 12.034843 | Open Source |
| *Mustela nivalis* | Native | 48.859985 | 9.229087 | Open Source |
| *Mustela nivalis* | Native | 53.609859 | 8.605536 | Open Source |
| *Mustela nivalis* | Native | 54.803169 | 8.293648 | Open Source |
| *Mustela nivalis* | Native | 49.39645 | 8.510628 | Open Source |
| *Mustela nivalis* | Native | 48.385792 | 9.962074 | Open Source |
| *Mustela nivalis* | Native | 53.474155 | 12.489228 | Open Source |
| *Mustela nivalis* | Native | 49.970375 | 8.340611999 | Open Source |
| *Mustela nivalis* | Native | 52.324844 | 4.838104 | Open Source |
| *Mustela nivalis* | Native | 51.493084 | 7.405351 | Open Source |
| *Mustela nivalis* | Native | 49.270908 | 7.522715 | Open Source |
| *Mustela nivalis* | Native | 51.772831 | 9.923015 | Open Source |
| *Mustela nivalis* | Native | 50.918701 | 9.197186999 | Open Source |
| *Mustela nivalis* | Native | 52.731869 | 12.247099 | Open Source |
| *Mustela nivalis* | Native | 51.664585 | 10.32648 | Open Source |
| *Mustela nivalis* | Native | 49.743015 | 11.255525 | Open Source |
| *Mustela nivalis* | Native | 51.359669 | 9.054623 | Open Source |
| *Mustela nivalis* | Native | 51.186802 | 6.991558999 | Open Source |
| *Mustela nivalis* | Native | 52.188526 | 9.746278 | Open Source |
| *Mustela nivalis* | Native | 51.621681 | 13.927649 | Open Source |
| *Mustela nivalis* | Native | 51.986641 | 9.832536 | Open Source |
| *Mustela nivalis* | Native | 50.692162 | 8.838415 | Open Source |
| *Mustela nivalis* | Native | 51.997082 | 9.856035001 | Open Source |
| *Mustela nivalis* | Native | 53.051174 | 6.638405001 | Open Source |
| *Mustela nivalis* | Native | 50.80257 | 8.366952 | Open Source |
| *Mustela nivalis* | Native | 52.339214 | 9.650937 | Open Source |
| *Mustela nivalis* | Native | 47.183617 | 11.37837 | Open Source |
| *Mustela nivalis* | Native | 54.023106 | 12.490243 | Open Source |
| *Mustela nivalis* | Native | 50.636124 | 6.4492 | Open Source |
| *Mustela nivalis* | Native | 49.370701 | 11.597204 | Open Source |
| *Mustela nivalis* | Native | 48.377846 | 9.929193999 | Open Source |
| *Mustela nivalis* | Native | 50.551399 | 8.488998 | Open Source |
| *Mustela nivalis* | Native | 52.061569 | 4.869646999 | Open Source |
| *Mustela nivalis* | Native | 48.492023 | 12.944847 | Open Source |
| *Mustela nivalis* | Native | 49.882088 | 8.337178 | Open Source |
| *Mustela nivalis* | Native | 48.745209 | 9.655952 | Open Source |
| *Mustela nivalis* | Native | 50.083759 | 8.508954 | Open Source |
| *Mustela nivalis* | Native | 54.752468 | 8.291845 | Open Source |
| *Mustela nivalis* | Native | 50.632221 | 8.650598 | Open Source |
| *Mustela nivalis* | Native | 50.81266 | 8.806367999 | Open Source |
| *Mustela nivalis* | Native | 51.498966 | 7.44607 | Open Source |
| *Mustela nivalis* | Native | 48.834145 | 12.951089 | Open Source |
| *Mustela nivalis* | Native | 52.533978 | 13.420229 | Open Source |
| *Mustela nivalis* | Native | 51.154022 | 12.404812 | Open Source |
| *Mustela nivalis* | Native | 49.916691 | 9.091744 | Open Source |
| *Mustela nivalis* | Native | 49.371147 | 11.602697 | Open Source |
| *Mustela nivalis* | Native | 50.853363 | 12.917974 | Open Source |
| *Mustela nivalis* | Native | 50.171181 | 6.237059 | Open Source |
| *Mustela nivalis* | Native | 50.715054 | 7.143722 | Open Source |
| *Mustela nivalis* | Native | 52.125481 | 10.152054 | Open Source |
| *Mustela nivalis* | Native | 52.045628 | 4.853382 | Open Source |
| *Mustela nivalis* | Native | 49.326099 | 16.550518 | Open Source |
| *Mustela nivalis* | Native | 52.790359 | 10.127592 | Open Source |
| *Mustela nivalis* | Native | 50.895344 | 12.839519 | Open Source |
| *Mustela nivalis* | Native | 52.301853 | 9.841846 | Open Source |
| *Mustela nivalis* | Native | 50.302608 | 7.072878 | Open Source |
| *Mustela nivalis* | Native | 52.136845 | 9.960357 | Open Source |
| *Mustela nivalis* | Native | 53.665825 | 12.571479 | Open Source |
| *Mustela nivalis* | Native | 49.865738 | 8.372967001 | Open Source |
| *Mustela nivalis* | Native | 51.183754 | 6.745155 | Open Source |
| *Mustela nivalis* | Native | 54.216087 | 10.904054 | Open Source |
| *Mustela nivalis* | Native | 50.861012 | 6.432837999 | Open Source |
| *Mustela nivalis* | Native | 54.176476 | 9.951145 | Open Source |
| *Mustela nivalis* | Native | 51.99865 | 9.856563 | Open Source |
| *Mustela nivalis* | Native | 50.478481 | 8.291344001 | Open Source |
| *Mustela nivalis* | Native | 49.024902 | 8.860335 | Open Source |
| *Mustela nivalis* | Native | 52.583496 | 10.070837 | Open Source |
| *Mustela nivalis* | Native | 52.087856 | 9.736026 | Open Source |
| *Mustela nivalis* | Native | 50.379108 | 7.277477 | Open Source |
| *Mustela nivalis* | Native | 51.552311 | 10.067253 | Open Source |
| *Mustela nivalis* | Native | 47.781963 | 8.844037 | Open Source |
| *Mustela nivalis* | Native | 51.99865 | 9.856281 | Open Source |
| *Mustela nivalis* | Native | 51.401348 | 6.682434 | Open Source |
| *Mustela nivalis* | Native | 51.804241 | 9.111259999 | Open Source |
| *Mustela nivalis* | Native | 50.284603 | 11.631807 | Open Source |
| *Mustela nivalis* | Native | 50.896927 | 9.283024001 | Open Source |
| *Mustela nivalis* | Native | 48.074467 | 11.636667 | Open Source |
| *Mustela nivalis* | Native | 54.405144 | 8.901672001 | Open Source |
| *Mustela nivalis* | Native | 49.976425 | 9.723029 | Open Source |
| *Mustela nivalis* | Native | 48.183029 | 16.477882 | Open Source |
| *Mustela nivalis* | Native | 60.109633 | 18.470189 | Open Source |
| *Mustela nivalis* | Native | 56.178573 | 13.176138 | Open Source |
| *Mustela nivalis* | Native | 58.406765 | 12.043857 | Open Source |
| *Mustela nivalis* | Native | 55.688839 | 13.165754 | Open Source |
| *Mustela nivalis* | Native | 59.32665 | 14.982992 | Open Source |
| *Mustela nivalis* | Native | 59.975809 | 16.035673 | Open Source |
| *Mustela nivalis* | Native | 56.722208 | 15.084277 | Open Source |
| *Mustela nivalis* | Native | 58.34465 | 14.697049 | Open Source |
| *Mustela nivalis* | Native | 57.413227 | 14.103909 | Open Source |
| *Mustela nivalis* | Native | 57.778057 | 14.601109 | Open Source |
| *Mustela nivalis* | Native | 56.915666 | 12.90093 | Open Source |
| *Mustela nivalis* | Native | 56.75831 | 12.637796 | Open Source |
| *Mustela nivalis* | Native | 55.798506 | 14.061671 | Open Source |
| *Mustela nivalis* | Native | 56.395078 | 16.436843 | Open Source |
| *Mustela nivalis* | Native | 58.11823 | 15.651087 | Open Source |
| *Mustela nivalis* | Native | 57.596687 | 12.080472 | Open Source |
| *Mustela nivalis* | Native | 56.412928 | 16.552699 | Open Source |
| *Mustela nivalis* | Native | 58.693923 | 17.143027 | Open Source |
| *Mustela nivalis* | Native | 61.208864 | 8.223331 | Open Source |
| *Mustela nivalis* | Native | 60.756868 | 11.237367 | Open Source |
| *Mustela nivalis* | Native | 60.757426 | 11.235687 | Open Source |
| *Mustela nivalis* | Native | 60.000405 | 11.087268 | Open Source |
| *Mustela nivalis* | Native | 58.788 | 5.737261 | Open Source |
| *Mustela nivalis* | Native | 58.981683 | 9.860493 | Open Source |
| *Mustela nivalis* | Native | 58.982739 | 9.864777001 | Open Source |
| *Mustela nivalis* | Native | 59.42138 | 9.321845 | Open Source |
| *Mustela nivalis* | Native | 59.788104 | 10.95297 | Open Source |
| *Mustela nivalis* | Native | 60.813118 | 11.692822 | Open Source |
| *Mustela nivalis* | Native | 58.196572 | 7.396692 | Open Source |
| *Mustela nivalis* | Native | 60.160726 | 11.557787 | Open Source |
| *Mustela nivalis* | Native | 60.085889 | 10.078711 | Open Source |
| *Mustela nivalis* | Native | 51.09541 | -3.12238 | Open Source |
| *Mustela nivalis* | Native | 51.2583 | -0.23151 | Open Source |
| *Mustela nivalis* | Native | 52.72072 | -3.326520001 | Open Source |
| *Mustela nivalis* | Native | 54.23374 | -3.03705 | Open Source |
| *Mustela nivalis* | Native | 51.32552 | 0.517470001 | Open Source |
| *Mustela nivalis* | Native | 51.29442 | 0.702249999 | Open Source |
| *Mustela nivalis* | Native | 53.7522 | -1.5996 | Open Source |
| *Mustela nivalis* | Native | 51.18975 | -1.522020001 | Open Source |
| *Mustela nivalis* | Native | 51.57464 | -1.20054 | Open Source |
| *Mustela nivalis* | Native | 52.06847 | 0.67579 | Open Source |
| *Mustela nivalis* | Native | 55.90087 | -4.08877 | Open Source |
| *Mustela nivalis* | Native | 50.88097 | -3.74269 | Open Source |
| *Mustela nivalis* | Native | 51.32501 | -0.50164 | Open Source |
| *Mustela nivalis* | Native | 52.70569 | -3.030049999 | Open Source |
| *Mustela nivalis* | Native | 51.52734 | -3.08972 | Open Source |
| *Mustela nivalis* | Native | 53.13241 | -1.75484 | Open Source |
| *Mustela nivalis* | Native | 51.18117 | -1.63656 | Open Source |
| *Mustela nivalis* | Native | 51.00601 | 0.72821 | Open Source |
| *Mustela nivalis* | Native | 55.1721 | -1.5855 | Open Source |
| *Mustela nivalis* | Native | 50.88097 | -3.74269 | Open Source |
| *Mustela nivalis* | Native | 51.61467 | 0.830054 | Open Source |
| *Mustela nivalis* | Native | 50.92483 | 0.73769 | Open Source |
| *Mustela nivalis* | Native | 52.88083 | -1.24732 | Open Source |
| *Mustela nivalis* | Native | 51.052161 | 0.100199 | Open Source |
| *Mustela nivalis* | Native | 54.149 | -2.794800001 | Open Source |
| *Mustela nivalis* | Native | 52.0559 | -3.6591 | Open Source |
| *Mustela nivalis* | Native | 54.25603 | -2.41755 | Open Source |
| *Mustela nivalis* | Native | 55.85778 | -3.242120001 | Open Source |
| *Mustela nivalis* | Native | 51.34264 | 0.88811 | Open Source |
| *Mustela nivalis* | Native | 54.971 | -4.5131 | Open Source |
| *Mustela nivalis* | Native | 51.3358 | 1.137400001 | Open Source |
| *Mustela nivalis* | Native | 53.09158 | -1.79166 | Open Source |
| *Mustela nivalis* | Native | 51.85778 | 0.23555 | Open Source |
| *Mustela nivalis* | Native | 57.7193 | -3.3309 | Open Source |
| *Mustela nivalis* | Native | 51.4906 | 0.217200001 | Open Source |
| *Mustela nivalis* | Native | 53.61312 | -2.458720001 | Open Source |
| *Mustela nivalis* | Native | 56.0312 | -2.7668 | Open Source |
| *Mustela nivalis* | Native | 51.3683 | 0.7357 | Open Source |
| *Mustela nivalis* | Native | 52.3272 | -2.1603 | Open Source |
| *Mustela nivalis* | Native | 54.541602 | -3.525689 | Open Source |
| *Mustela nivalis* | Native | 54.83052 | -1.49817 | Open Source |
| *Mustela nivalis* | Native | 54.6439 | -1.1385 | Open Source |
| *Mustela nivalis* | Native | 53.51157 | -2.98427 | Open Source |
| *Mustela nivalis* | Native | 54.542919 | -3.537393999 | Open Source |
| *Mustela nivalis* | Native | 54.6441 | -3.536389999 | Open Source |
| *Mustela nivalis* | Native | 55.9504 | -3.161740001 | Open Source |
| *Mustela nivalis* | Native | 57.5883 | -4.021800001 | Open Source |
| *Mustela nivalis* | Native | 54.56677 | -3.56388 | Open Source |
| *Mustela nivalis* | Native | 54.65633 | -2.839300001 | Open Source |
| *Mustela nivalis* | Native | 50.14521 | -5.4616 | Open Source |
| *Mustela nivalis* | Native | 56.3077 | -2.785 | Open Source |
| *Mustela nivalis* | Native | 57.3054 | -4.0191 | Open Source |
| *Mustela nivalis* | Native | 51.612 | 0.8187 | Open Source |
| *Mustela nivalis* | Native | 52.86696 | -1.066329999 | Open Source |
| *Mustela nivalis* | Native | 51.7579 | 0.7628 | Open Source |
| *Mustela nivalis* | Native | 51.75952 | 0.29446 | Open Source |
| *Mustela nivalis* | Native | 55.941 | -4.8797 | Open Source |
| *Mustela nivalis* | Native | 52.9792 | -2.2634 | Open Source |
| *Mustela nivalis* | Native | 52.5864 | -1.7054 | Open Source |
| *Mustela nivalis* | Native | 53.4407 | -2.5 | Open Source |
| *Mustela nivalis* | Native | 52.3348 | -2.1585 | Open Source |
| *Mustela nivalis* | Native | 53.7769 | -2.6292 | Open Source |
| *Mustela nivalis* | Native | 52.91335 | -1.27352 | Open Source |
| *Mustela nivalis* | Native | 54.165 | -2.8022 | Open Source |
| *Mustela nivalis* | Native | 53.5278 | -2.3167 | Open Source |
| *Mustela nivalis* | Native | 54.9552 | -1.3708 | Open Source |
| *Mustela nivalis* | Native | 53.33661 | -2.87924 | Open Source |
| *Mustela nivalis* | Native | 50.69812 | -2.640010001 | Open Source |
| *Mustela nivalis* | Native | 51.08927 | -1.87494 | Open Source |
| *Mustela nivalis* | Native | 53.2694 | -1.715829999 | Open Source |
| *Mustela nivalis* | Native | 55.5206 | -4.160799999 | Open Source |
| *Mustela nivalis* | Native | 54.8721 | -4.2013 | Open Source |
| *Mustela nivalis* | Native | 51.328212 | 1.316750999 | Open Source |
| *Mustela nivalis* | Native | 54.8313 | -1.49486 | Open Source |
| *Mustela nivalis* | Native | 56.4463 | -3.4744 | Open Source |
| *Mustela nivalis* | Native | 50.85613 | -1.078410001 | Open Source |
| *Mustela nivalis* | Native | 53.0125 | -0.8538 | Open Source |
| *Mustela nivalis* | Native | 53.571 | -2.87844 | Open Source |
| *Mustela nivalis* | Native | 52.5776 | -0.9441 | Open Source |
| *Mustela nivalis* | Native | 56.1748 | -3.3579 | Open Source |
| *Mustela nivalis* | Native | 52.17642 | -2.342370001 | Open Source |
| *Mustela nivalis* | Native | 52.3583 | -2.314599999 | Open Source |
| *Mustela nivalis* | Native | 50.93426 | -0.81238 | Open Source |
| *Mustela nivalis* | Native | 56.1748 | -3.3581 | Open Source |
| *Mustela nivalis* | Native | 54.54725 | -3.15321 | Open Source |
| *Mustela nivalis* | Native | 54.6187 | -3.314299999 | Open Source |
| *Mustela nivalis* | Native | 51.31817 | 1.20292 | Open Source |
| *Mustela nivalis* | Native | 51.730178 | -5.128752 | Open Source |
| *Mustela nivalis* | Native | 54.38411 | -3.32956 | Open Source |
| *Mustela nivalis* | Native | 52.689477 | -3.363386 | Open Source |
| *Mustela nivalis* | Native | 56.1769 | -3.358699999 | Open Source |
| *Mustela nivalis* | Native | 55.0157 | -1.5304 | Open Source |
| *Mustela nivalis* | Native | 54.243425 | -3.007371001 | Open Source |
| *Mustela nivalis* | Native | 51.7579 | 0.762700001 | Open Source |
| *Mustela nivalis* | Native | 52.44943 | -2.08235 | Open Source |
| *Mustela nivalis* | Native | 51.377 | 0.7847 | Open Source |
| *Mustela nivalis* | Native | 50.74271 | -2.18809 | Open Source |
| *Mustela nivalis* | Native | 50.3217 | -4.2007 | Open Source |
| *Mustela nivalis* | Native | 50.93763 | -0.287109999 | Open Source |
| *Mustela nivalis* | Native | 56.93491 | -2.9594 | Open Source |
| *Mustela nivalis* | Native | 57.30927 | -3.38688 | Open Source |
| *Mustela nivalis* | Native | 57.58752 | -2.712499999 | Open Source |
| *Mustela nivalis* | Native | 57.34172 | -2.29321 | Open Source |
| *Mustela nivalis* | Native | 57.17788 | -3.81804 | Open Source |
| *Mustela nivalis* | Native | 57.2259 | -2.38744 | Open Source |
| *Mustela nivalis* | Native | 57.3259 | -4.22963 | Open Source |
| *Mustela nivalis* | Native | 57.31881 | -3.603939999 | Open Source |
| *Mustela nivalis* | Native | 57.09693 | -2.34863 | Open Source |
| *Mustela nivalis* | Native | 56.76884 | -3.85505 | Open Source |
| *Mustela nivalis* | Native | 57.60654 | -2.643170001 | Open Source |
| *Mustela nivalis* | Native | 57.08475 | -2.861300001 | Open Source |
| *Mustela nivalis* | Native | 56.63132 | -3.842840001 | Open Source |
| *Mustela nivalis* | Native | 56.90763 | -2.94409 | Open Source |
| *Mustela nivalis* | Native | 57.33802 | -2.026560001 | Open Source |
| *Mustela nivalis* | Native | 57.59762 | -3.61537 | Open Source |
| *Mustela nivalis* | Native | 57.65184 | -3.079869999 | Open Source |
| *Mustela nivalis* | Native | 57.07189 | -3.999899999 | Open Source |
| *Mustela nivalis* | Native | 57.1802 | -2.20757 | Open Source |
| *Mustela nivalis* | Native | 57.64154 | -3.55198 | Open Source |
| *Mustela nivalis* | Native | 57.40985 | -2.09319 | Open Source |
| *Mustela nivalis* | Native | 56.73234 | -3.850150001 | Open Source |
| *Mustela nivalis* | Native | 57.02059 | -2.64809 | Open Source |
| *Mustela nivalis* | Native | 57.40872 | -2.425240001 | Open Source |
| *Mustela nivalis* | Native | 57.68269 | -2.928179999 | Open Source |
| *Mustela nivalis* | Native | 57.29482 | -3.310000001 | Open Source |
| *Mustela nivalis* | Native | 56.97617 | -2.26895 | Open Source |
| *Mustela nivalis* | Native | 57.07808 | -4.088510001 | Open Source |
| *Mustela nivalis* | Native | 57.67484 | -3.1208 | Open Source |
| *Mustela nivalis* | Native | 56.99399 | -2.328330001 | Open Source |
| *Mustela nivalis* | Native | 57.07339 | -4.018130001 | Open Source |
| *Mustela nivalis* | Native | 57.04953 | -2.38486 | Open Source |
| *Mustela nivalis* | Native | 57.2939 | -3.31163 | Open Source |
| *Mustela nivalis* | Native | 56.7705 | -3.91748 | Open Source |
| *Mustela nivalis* | Native | 57.15043 | -2.27352 | Open Source |
| *Mustela nivalis* | Native | 57.6425 | -3.23229 | Open Source |
| *Mustela nivalis* | Native | 57.32118 | -2.530490001 | Open Source |
| *Mustela nivalis* | Native | 57.64897 | -3.53219 | Open Source |
| *Mustela nivalis* | Native | 57.08547 | -2.301 | Open Source |
| *Mustela nivalis* | Native | 57.14167 | -2.6849 | Open Source |
| *Mustela nivalis* | Native | 57.15583 | -2.723180001 | Open Source |
| *Mustela nivalis* | Native | 56.7705 | -3.91748 | Open Source |
| *Mustela nivalis* | Native | 57.45902 | -3.240860001 | Open Source |
| *Mustela nivalis* | Native | 57.22958 | -3.66053 | Open Source |
| *Mustela nivalis* | Native | 57.16477 | -2.73163 | Open Source |
| *Mustela nivalis* | Native | 57.38439 | -4.039349999 | Open Source |
| *Mustela nivalis* | Native | 57.29381 | -3.64442 | Open Source |
| *Mustela nivalis* | Native | 56.58299 | -3.54179 | Open Source |
| *Mustela nivalis* | Native | 57.29955 | -3.70269 | Open Source |
| *Mustela nivalis* | Native | 57.23756 | -2.72644 | Open Source |
| *Mustela nivalis* | Native | 56.98396 | -4.330120001 | Open Source |
| *Mustela nivalis* | Native | 57.59383 | -3.559990001 | Open Source |
| *Mustela nivalis* | Native | 57.58999 | -3.579899999 | Open Source |
| *Mustela nivalis* | Native | 57.56831 | -2.16966 | Open Source |
| *Mustela nivalis* | Native | 57.51758 | -1.84307 | Open Source |
| *Mustela nivalis* | Native | 57.2212 | -2.75924 | Open Source |
| *Mustela nivalis* | Native | 57.5306 | -3.6575 | Open Source |
| *Mustela nivalis* | Native | 56.77038 | -3.91674 | Open Source |
| *Mustela nivalis* | Native | 57.42995 | -3.6596 | Open Source |
| *Mustela nivalis* | Native | 57.24444 | -3.757320001 | Open Source |
| *Mustela nivalis* | Native | 56.96449 | -2.26722 | Open Source |
| *Mustela nivalis* | Native | 57.10506 | -2.434959999 | Open Source |
| *Mustela nivalis* | Native | 57.40113 | -2.21074 | Open Source |
| *Mustela nivalis* | Native | 57.49967 | -1.893199999 | Open Source |
| *Mustela nivalis* | Native | 57.67795 | -2.5969 | Open Source |
| *Mustela nivalis* | Native | 57.29381 | -3.64442 | Open Source |
| *Mustela nivalis* | Native | 57.67418 | -2.716710001 | Open Source |
| *Mustela nivalis* | Native | 57.09144 | -2.4744 | Open Source |
| *Mustela nivalis* | Native | 57.5306 | -3.6575 | Open Source |
| *Mustela nivalis* | Native | 57.3195 | -2.503900001 | Open Source |
| *Mustela nivalis* | Native | 57.51104 | -2.808650001 | Open Source |
| *Mustela nivalis* | Native | 56.97046 | -3.12916 | Open Source |
| *Mustela nivalis* | Native | 57.59609 | -3.278989999 | Open Source |
| *Mustela nivalis* | Native | 57.3379 | -2.17607 | Open Source |
| *Mustela nivalis* | Native | 57.08141 | -3.96576 | Open Source |
| *Mustela nivalis* | Native | 57.30126 | -2.45802 | Open Source |
| *Mustela nivalis* | Native | 57.092 | -2.934 | Open Source |
| *Mustela nivalis* | Native | 57.22555 | -3.62557 | Open Source |
| *Mustela nivalis* | Native | 56.90837 | -2.940489999 | Open Source |
| *Mustela nivalis* | Native | 56.7705 | -3.91748 | Open Source |
| *Mustela nivalis* | Native | 57.04898 | -2.24805 | Open Source |
| *Mustela nivalis* | Native | 57.39551 | -2.350360001 | Open Source |
| *Mustela nivalis* | Native | 57.25397 | -3.750319999 | Open Source |
| *Mustela nivalis* | Native | 57.22741 | -3.62068 | Open Source |
| *Mustela nivalis* | Native | 57.293 | -3.311599999 | Open Source |
| *Mustela nivalis* | Native | 57.44195 | -2.79713 | Open Source |
| *Mustela nivalis* | Native | 57.26309 | -2.121419999 | Open Source |
| *Mustela nivalis* | Native | 57.37874 | -2.60458 | Open Source |
| *Mustela nivalis* | Native | 57.67484 | -3.1208 | Open Source |
| *Mustela nivalis* | Native | 57.67382 | -2.912870001 | Open Source |
| *Mustela nivalis* | Native | 57.33648 | -2.89629 | Open Source |
| *Mustela nivalis* | Native | 57.2175 | -2.97028 | Open Source |
| *Mustela nivalis* | Native | 57.08112 | -2.5782 | Open Source |
| *Mustela nivalis* | Native | 57.18462 | -2.536810001 | Open Source |
| *Mustela nivalis* | Native | 57.30983 | -3.603549999 | Open Source |
| *Mustela nivalis* | Native | 57.30875 | -3.68651 | Open Source |
| *Mustela nivalis* | Native | 57.51541 | -2.67687 | Open Source |
| *Mustela nivalis* | Native | 57.49745 | -1.9491 | Open Source |
| *Mustela nivalis* | Native | 57.14735 | -3.66263 | Open Source |
| *Mustela nivalis* | Native | 57.31323 | -2.498840001 | Open Source |
| *Mustela nivalis* | Native | 57.06094 | -2.41626 | Open Source |
| *Mustela nivalis* | Native | 57.42619 | -3.09987 | Open Source |
| *Mustela nivalis* | Native | 56.95151 | -3.138460001 | Open Source |
| *Mustela nivalis* | Native | 57.15288 | -2.37106 | Open Source |
| *Mustela nivalis* | Native | 57.5436 | -2.90794 | Open Source |
| *Mustela nivalis* | Native | 57.0586 | -2.487999999 | Open Source |
| *Mustela nivalis* | Native | 57.35104 | -1.942649999 | Open Source |
| *Mustela nivalis* | Native | 57.14333 | -2.2305 | Open Source |
| *Mustela nivalis* | Native | 57.07889 | -4.039049999 | Open Source |
| *Mustela nivalis* | Native | 57.30516 | -3.935360001 | Open Source |
| *Mustela nivalis* | Native | 57.09989 | -2.36557 | Open Source |
| *Mustela nivalis* | Native | 57.43685 | -3.30175 | Open Source |
| *Mustela nivalis* | Native | 56.80191 | -2.539509999 | Open Source |
| *Mustela nivalis* | Native | 57.51064 | -2.99057 | Open Source |
| *Mustela nivalis* | Native | 57.03602 | -2.23503 | Open Source |
| *Mustela nivalis* | Native | 57.09506 | -3.954930001 | Open Source |
| *Mustela nivalis* | Native | 57.4907 | -1.90991 | Open Source |
| *Mustela nivalis* | Native | 57.49745 | -1.9491 | Open Source |
| *Mustela nivalis* | Native | 57.22209 | -2.75926 | Open Source |
| *Mustela nivalis* | Native | 57.35748 | -2.436280001 | Open Source |
| *Mustela nivalis* | Native | 57.46801 | -2.79436 | Open Source |
| *Mustela nivalis* | Native | 57.28159 | -3.70186 | Open Source |
| *Mustela nivalis* | Native | 57.12935 | -2.720189999 | Open Source |
| *Mustela nivalis* | Native | 57.1872 | -2.561660001 | Open Source |
| *Mustela nivalis* | Native | 57.2212 | -2.75924 | Open Source |
| *Mustela nivalis* | Native | 57.09657 | -2.337749999 | Open Source |
| *Mustela nivalis* | Native | 55.94196 | -3.07774 | Open Source |
| *Mustela nivalis* | Native | 55.41807 | -3.288250001 | Open Source |
| *Mustela nivalis* | Native | 54.9358 | -3.96245 | Open Source |
| *Mustela nivalis* | Native | 55.42076 | -3.28834 | Open Source |
| *Mustela nivalis* | Native | 57.35669 | -2.76935 | Open Source |
| *Mustela nivalis* | Native | 56.5228 | -4.16434 | Open Source |
| *Mustela nivalis* | Native | 57.3207 | -5.68546 | Open Source |
| *Mustela nivalis* | Native | 57.672491 | 10.498896 | Open Source |
| *Mustela nivalis* | Native | 55.944278 | 8.472622 | Open Source |
| *Mustela nivalis* | Native | 55.109299 | 10.205836 | Open Source |
| *Mustela nivalis* | Native | 55.86645 | 12.417212 | Open Source |
| *Mustela nivalis* | Native | 55.695293 | 8.224727999 | Open Source |
| *Mustela nivalis* | Native | 56.420785 | 9.719788 | Open Source |
| *Mustela nivalis* | Native | 55.462361 | 11.512343 | Open Source |
| *Mustela nivalis* | Native | 55.886817 | 11.568775 | Open Source |
| *Mustela nivalis* | Native | 55.076839 | 10.433454 | Open Source |
| *Mustela nivalis* | Native | 57.021004 | 9.967117001 | Open Source |
| *Mustela nivalis* | Native | 55.161612 | 9.487896001 | Open Source |
| *Mustela nivalis* | Native | 55.79276 | 9.704552001 | Open Source |
| *Mustela nivalis* | Native | 56.001972 | 12.513856 | Open Source |
| *Mustela nivalis* | Native | 56.984417 | 9.631686 | Open Source |
| *Mustela nivalis* | Native | 55.743878 | 11.181101 | Open Source |
| *Mustela nivalis* | Native | 56.652782 | 9.063549 | Open Source |
| *Mustela nivalis* | Native | 57.06911 | 9.883404 | Open Source |
| *Mustela nivalis* | Native | 56.89841 | 10.18836 | Open Source |
| *Mustela nivalis* | Native | 57.732569 | 10.617724 | Open Source |
| *Mustela nivalis* | Native | 56.150683 | 10.104096 | Open Source |
| *Mustela nivalis* | Native | 55.61302 | 9.484978 | Open Source |
| *Mustela nivalis* | Native | 55.879175 | 10.170615 | Open Source |
| *Mustela nivalis* | Native | 56.890408 | 8.585664 | Open Source |
| *Mustela nivalis* | Native | 56.887815 | 10.209475 | Open Source |
| *Mustela nivalis* | Native | 55.652459 | 12.528148 | Open Source |
| *Mustela nivalis* | Native | 56.441015 | 9.97697 | Open Source |
| *Mustela nivalis* | Native | 56.886315 | 10.2178 | Open Source |
| *Mustela nivalis* | Native | 56.27837 | 12.955907 | Open Source |
| *Mustela nivalis* | Native | 59.944757 | 17.308954 | Open Source |
| *Mustela nivalis* | Native | 58.659958 | 16.764827 | Open Source |
| *Mustela nivalis* | Native | 57.571791 | 13.127503 | Open Source |
| *Mustela nivalis* | Native | 55.690333 | 13.493585 | Open Source |
| *Mustela nivalis* | Native | 56.234773 | 16.458789 | Open Source |
| *Mustela nivalis* | Native | 59.399908 | 17.922189 | Open Source |
| *Mustela nivalis* | Native | 59.964107 | 17.274836 | Open Source |
| *Mustela nivalis* | Native | 59.767897 | 17.505452 | Open Source |
| *Mustela nivalis* | Native | 56.069236 | 14.473674 | Open Source |
| *Mustela nivalis* | Native | 56.499121 | 13.635249 | Open Source |
| *Mustela nivalis* | Native | 58.179046 | 13.942931 | Open Source |
| *Mustela nivalis* | Native | 58.459867 | 15.565801 | Open Source |
| *Mustela nivalis* | Native | 58.459867 | 15.565801 | Open Source |
| *Mustela nivalis* | Native | 59.247925 | 17.095641 | Open Source |
| *Mustela nivalis* | Native | 55.883875 | 12.945154 | Open Source |
| *Mustela nivalis* | Native | 58.325684 | 13.583515 | Open Source |
| *Mustela nivalis* | Native | 58.049656 | 15.060148 | Open Source |
| *Mustela nivalis* | Native | 59.72634 | 17.308317 | Open Source |
| *Mustela nivalis* | Native | 59.846695 | 17.670104 | Open Source |
| *Mustela nivalis* | Native | 58.083297 | 13.408254 | Open Source |
| *Mustela nivalis* | Native | 56.656476 | 12.910875 | Open Source |
| *Mustela nivalis* | Native | 57.352308 | 14.103882 | Open Source |
| *Mustela nivalis* | Native | 57.811269 | 14.27511 | Open Source |
| *Mustela nivalis* | Native | 60.000701 | 15.022086 | Open Source |
| *Mustela nivalis* | Native | 56.626215 | 12.917819 | Open Source |
| *Mustela nivalis* | Native | 58.542852 | 11.96529 | Open Source |
| *Mustela nivalis* | Native | 61.074006 | 14.654091 | Open Source |
| *Mustela nivalis* | Native | 55.536394 | 13.420085 | Open Source |
| *Mustela nivalis* | Native | 57.958153 | 15.828252 | Open Source |
| *Mustela nivalis* | Native | 42.723825 | 0.113336 | Open Source |
| *Mustela nivalis* | Native | 51.286259 | 9.275414 | Open Source |
| *Mustela nivalis* | Native | 52.799492 | 7.365603 | Open Source |
| *Mustela nivalis* | Native | 49.273949 | 11.706909 | Open Source |
| *Mustela nivalis* | Native | 49.964638 | 10.183897 | Open Source |
| *Mustela nivalis* | Native | 53.349834 | 10.507393 | Open Source |
| *Mustela nivalis* | Native | 49.624557 | 8.636112 | Open Source |
| *Mustela nivalis* | Native | 49.971748 | 10.219 | Open Source |
| *Mustela nivalis* | Native | 50.088028 | 8.649333 | Open Source |
| *Mustela nivalis* | Native | 50.368652 | 8.885365 | Open Source |
| *Mustela nivalis* | Native | 42.423281 | -5.219963999 | Open Source |
| *Mustela nivalis* | Native | 60.406146 | 22.286765 | Open Source |
| *Mustela nivalis* | Native | 61.311764 | 25.937045 | Open Source |
| *Mustela nivalis* | Native | 60.462841 | 22.138306 | Open Source |
| *Mustela nivalis* | Native | 60.218912 | 24.818157 | Open Source |
| *Mustela nivalis* | Native | 60.206364 | 24.680945 | Open Source |
| *Mustela nivalis* | Native | 60.217863 | 24.780979 | Open Source |
| *Mustela nivalis* | Native | 60.202967 | 24.774518 | Open Source |
| *Mustela nivalis* | Native | 60.463802 | 22.139983 | Open Source |
| *Mustela nivalis* | Native | 60.861667 | 28.298752 | Open Source |
| *Mustela nivalis* | Native | 60.241107 | 24.872971 | Open Source |
| *Mustela nivalis* | Native | 60.206126 | 24.808418 | Open Source |
| *Mustela nivalis* | Native | 60.220617 | 25.009311 | Open Source |
| *Mustela nivalis* | Native | 60.78137 | 21.427266 | Open Source |
| *Mustela nivalis* | Native | 60.197174 | 24.676561 | Open Source |
| *Mustela nivalis* | Native | 60.224253 | 24.775955 | Open Source |
| *Mustela nivalis* | Native | 60.233669 | 24.781112 | Open Source |
| *Mustela nivalis* | Native | 60.222116 | 24.818089 | Open Source |
| *Mustela nivalis* | Native | 60.23196 | 24.773988 | Open Source |
| *Mustela nivalis* | Native | 60.160888 | 24.890608 | Open Source |
| *Mustela nivalis* | Native | 60.194164 | 24.775925 | Open Source |
| *Mustela nivalis* | Native | 60.219594 | 24.819863 | Open Source |
| *Mustela nivalis* | Native | 60.237111 | 25.003697 | Open Source |
| *Mustela nivalis* | Native | 60.223848 | 24.86326 | Open Source |
| *Mustela nivalis* | Native | 60.230632 | 24.786362 | Open Source |
| *Mustela nivalis* | Native | 60.212256 | 24.771073 | Open Source |
| *Mustela nivalis* | Native | 60.219027 | 24.814865 | Open Source |
| *Mustela nivalis* | Native | 60.206762 | 24.814274 | Open Source |
| *Mustela nivalis* | Native | 60.216309 | 24.755838 | Open Source |
| *Mustela nivalis* | Native | 60.222364 | 24.764557 | Open Source |
| *Mustela nivalis* | Native | 60.224328 | 24.789061 | Open Source |
| *Mustela nivalis* | Native | 60.210273 | 24.816458 | Open Source |
| *Mustela nivalis* | Native | 60.235586 | 24.784612 | Open Source |
| *Mustela nivalis* | Native | 60.205089 | 24.765625 | Open Source |
| *Mustela nivalis* | Native | 61.174391 | 25.531405 | Open Source |
| *Mustela nivalis* | Native | 60.206865 | 24.764981 | Open Source |
| *Mustela nivalis* | Native | 60.208689 | 24.766661 | Open Source |
| *Mustela nivalis* | Native | 60.214543 | 24.768735 | Open Source |
| *Mustela nivalis* | Native | 60.228341 | 24.786896 | Open Source |
| *Mustela nivalis* | Native | 61.347916 | 22.110746 | Open Source |
| *Mustela nivalis* | Native | 60.550595 | 27.003286 | Open Source |
| *Mustela nivalis* | Native | 60.222427 | 24.746503 | Open Source |
| *Mustela nivalis* | Native | 60.206382 | 24.680926 | Open Source |
| *Mustela nivalis* | Native | 61.362337 | 25.292425 | Open Source |
| *Mustela nivalis* | Native | 60.214974 | 24.887747 | Open Source |
| *Mustela nivalis* | Native | 60.212358 | 24.790031 | Open Source |
| *Mustela nivalis* | Native | 61.241529 | 26.055898 | Open Source |
| *Mustela nivalis* | Native | 60.233655 | 24.775541 | Open Source |
| *Mustela nivalis* | Native | 60.217887 | 24.781284 | Open Source |
| *Mustela nivalis* | Native | 60.404221 | 22.463259 | Open Source |
| *Mustela nivalis* | Native | 61.203952 | 26.04441 | Open Source |
| *Mustela nivalis* | Native | 60.3494 | 24.838687 | Open Source |
| *Mustela nivalis* | Native | 60.220608 | 24.800152 | Open Source |
| *Mustela nivalis* | Native | 60.273507 | 24.629398 | Open Source |
| *Mustela nivalis* | Native | 60.245707 | 24.874841 | Open Source |
| *Mustela nivalis* | Native | 60.453103 | 22.247921 | Open Source |
| *Mustela nivalis* | Native | 60.217112 | 24.815715 | Open Source |
| *Mustela nivalis* | Native | 60.232135 | 24.787199 | Open Source |
| *Mustela nivalis* | Native | 61.833035 | 24.521412 | Open Source |
| *Mustela nivalis* | Native | 60.863006 | 28.30142 | Open Source |
| *Mustela nivalis* | Native | 59.843397 | 23.208757 | Open Source |
| *Mustela nivalis* | Native | 60.152862 | 19.603909 | Open Source |
| *Mustela nivalis* | Native | 60.169918 | 24.768771 | Open Source |
| *Mustela nivalis* | Native | 60.423149 | 25.106074 | Open Source |
| *Mustela nivalis* | Native | 61.170391 | 28.784878 | Open Source |
| *Mustela nivalis* | Native | 60.208724 | 24.780479 | Open Source |
| *Mustela nivalis* | Native | 60.224211 | 24.782608 | Open Source |
| *Mustela nivalis* | Native | 60.900951 | 22.245213 | Open Source |
| *Mustela nivalis* | Native | 61.222705 | 21.70402 | Open Source |
| *Mustela nivalis* | Native | 60.236442 | 24.903067 | Open Source |
| *Mustela nivalis* | Native | 60.278824 | 24.650754 | Open Source |
| *Mustela nivalis* | Native | 60.454075 | 22.285701 | Open Source |
| *Mustela nivalis* | Native | 60.274483 | 24.623759 | Open Source |
| *Mustela nivalis* | Native | 60.203968 | 24.659015 | Open Source |
| *Mustela nivalis* | Native | 60.832336 | 23.817038 | Open Source |
| *Mustela nivalis* | Native | 60.861356 | 28.298556 | Open Source |
| *Mustela nivalis* | Native | 60.286243 | 24.646261 | Open Source |
| *Mustela nivalis* | Native | 60.212301 | 24.789835 | Open Source |
| *Mustela nivalis* | Native | 60.830993 | 21.386427 | Open Source |
| *Mustela nivalis* | Native | 60.448031 | 25.088259 | Open Source |
| *Mustela nivalis* | Native | 60.375913 | 25.000003 | Open Source |
| *Mustela nivalis* | Native | 60.269008 | 24.632941 | Open Source |
| *Mustela nivalis* | Native | 60.227464 | 24.873766 | Open Source |
| *Mustela nivalis* | Native | 60.171248 | 24.764572 | Open Source |
| *Mustela nivalis* | Native | 60.219731 | 24.891006 | Open Source |
| *Mustela nivalis* | Native | 60.226497 | 25.006896 | Open Source |
| *Mustela nivalis* | Native | 60.170981 | 24.774322 | Open Source |
| *Mustela nivalis* | Native | 60.27528 | 24.629125 | Open Source |
| *Mustela nivalis* | Native | 60.22307 | 24.800319 | Open Source |
| *Mustela nivalis* | Native | 60.459793 | 22.153329 | Open Source |
| *Mustela nivalis* | Native | 61.423628 | 23.634204 | Open Source |
| *Mustela nivalis* | Native | 60.29206 | 24.614513 | Open Source |
| *Mustela nivalis* | Native | 61.064314 | 22.353657 | Open Source |
| *Mustela nivalis* | Native | 60.589094 | 26.136574 | Open Source |
| *Mustela nivalis* | Native | 60.192273 | 24.613539 | Open Source |
| *Mustela nivalis* | Native | 60.861891 | 28.298577 | Open Source |
| *Mustela nivalis* | Native | 60.223814 | 24.863371 | Open Source |
| *Mustela nivalis* | Native | 60.190544 | 24.601999 | Open Source |
| *Mustela nivalis* | Native | 61.162559 | 21.59826 | Open Source |
| *Mustela nivalis* | Native | 60.230617 | 24.621149 | Open Source |
| *Mustela nivalis* | Native | 60.218802 | 24.818598 | Open Source |
| *Mustela nivalis* | Native | 60.228799 | 24.746354 | Open Source |
| *Mustela nivalis* | Native | 60.216998 | 24.83838 | Open Source |
| *Mustela nivalis* | Native | 60.216948 | 24.781529 | Open Source |
| *Mustela nivalis* | Native | 61.021911 | 25.507704 | Open Source |
| *Mustela nivalis* | Native | 60.226762 | 24.804198 | Open Source |
| *Mustela nivalis* | Native | 60.981617 | 24.363324 | Open Source |
| *Mustela nivalis* | Native | 60.224968 | 25.009101 | Open Source |
| *Mustela nivalis* | Native | 60.861007 | 28.298358 | Open Source |
| *Mustela nivalis* | Native | 60.287868 | 24.656941 | Open Source |
| *Mustela nivalis* | Native | 60.21718 | 24.817136 | Open Source |
| *Mustela nivalis* | Native | 60.359803 | 24.718618 | Open Source |
| *Mustela nivalis* | Native | 60.272067 | 21.595831 | Open Source |
| *Mustela nivalis* | Native | 60.214312 | 24.81148 | Open Source |
| *Mustela nivalis* | Native | 60.751927 | 22.095373 | Open Source |
| *Mustela nivalis* | Native | 60.22518 | 25.008864 | Open Source |
| *Mustela nivalis* | Native | 60.661988 | 23.61098 | Open Source |
| *Mustela nivalis* | Native | 60.196676 | 24.938202 | Open Source |
| *Mustela nivalis* | Native | 60.964385 | 24.561057 | Open Source |
| *Mustela nivalis* | Native | 60.258811 | 24.876457 | Open Source |
| *Mustela nivalis* | Native | 60.210188 | 25.0388 | Open Source |
| *Mustela nivalis* | Native | 60.195291 | 24.93865 | Open Source |
| *Mustela nivalis* | Native | 61.153424 | 24.037816 | Open Source |
| *Mustela nivalis* | Native | 60.172489 | 24.934817 | Open Source |
| *Mustela nivalis* | Native | 61.10283 | 28.496402 | Open Source |
| *Mustela nivalis* | Native | 60.493194 | 22.300951 | Open Source |
| *Mustela nivalis* | Native | 60.224629 | 24.796045 | Open Source |
| *Mustela nivalis* | Native | 60.221079 | 24.764473 | Open Source |
| *Mustela nivalis* | Native | 60.224416 | 24.688613 | Open Source |
| *Mustela nivalis* | Native | 60.218092 | 24.804741 | Open Source |
| *Mustela nivalis* | Native | 60.561909 | 22.240389 | Open Source |
| *Mustela nivalis* | Native | 61.251455 | 24.225351 | Open Source |
| *Mustela nivalis* | Native | 60.558488 | 22.248326 | Open Source |
| *Mustela nivalis* | Native | 60.230513 | 24.772604 | Open Source |
| *Mustela nivalis* | Native | 60.211462 | 24.781277 | Open Source |
| *Mustela nivalis* | Native | 61.25836 | 24.230204 | Open Source |
| *Mustela nivalis* | Native | 60.217224 | 24.801081 | Open Source |
| *Mustela nivalis* | Native | 61.026209 | 26.144501 | Open Source |
| *Mustela nivalis* | Native | 60.213371 | 24.799913 | Open Source |
| *Mustela nivalis* | Native | 60.716923 | 22.343373 | Open Source |
| *Mustela nivalis* | Native | 60.439381 | 22.375218 | Open Source |
| *Mustela nivalis* | Native | 60.227512 | 24.789904 | Open Source |
| *Mustela nivalis* | Native | 60.835763 | 21.252021 | Open Source |
| *Mustela nivalis* | Native | 61.248361 | 21.711576 | Open Source |
| *Mustela nivalis* | Native | 60.288533 | 22.189968 | Open Source |
| *Mustela nivalis* | Native | 60.860869 | 28.298288 | Open Source |
| *Mustela nivalis* | Native | 60.564813 | 22.23914 | Open Source |
| *Mustela nivalis* | Native | 61.224181 | 21.591933 | Open Source |
| *Mustela nivalis* | Native | 60.49031 | 22.209451 | Open Source |
| *Mustela nivalis* | Native | 60.835763 | 21.252021 | Open Source |
| *Mustela nivalis* | Native | 60.223568 | 24.786035 | Open Source |
| *Mustela nivalis* | Native | 60.227351 | 24.793191 | Open Source |
| *Mustela nivalis* | Native | 60.432242 | 22.261567 | Open Source |
| *Mustela nivalis* | Native | 60.648486 | 24.841854 | Open Source |
| *Mustela nivalis* | Native | 60.285701 | 24.852434 | Open Source |
| *Mustela nivalis* | Native | 60.250889 | 25.033602 | Open Source |
| *Mustela nivalis* | Native | 61.25207 | 24.222519 | Open Source |
| *Mustela nivalis* | Native | 60.209767 | 24.781951 | Open Source |
| *Mustela nivalis* | Native | 60.629227 | 24.771207 | Open Source |
| *Mustela nivalis* | Native | 60.590014 | 22.309929 | Open Source |
| *Mustela nivalis* | Native | 60.229433 | 24.818604 | Open Source |
| *Mustela nivalis* | Native | 60.42519 | 22.291839 | Open Source |
| *Mustela nivalis* | Native | 60.95504 | 21.689302 | Open Source |
| *Mustela nivalis* | Native | 60.174169 | 24.200353 | Open Source |
| *Mustela nivalis* | Native | 60.088426 | 24.486772 | Open Source |
| *Mustela nivalis* | Native | 50.767395 | 6.48622 | Open Source |
| *Mustela nivalis* | Native | 54.525364 | 9.867498001 | Open Source |
| *Mustela nivalis* | Native | 48.928589 | 9.479140999 | Open Source |
| *Mustela nivalis* | Native | 50.150616 | 8.675508 | Open Source |
| *Mustela nivalis* | Native | 50.005642 | 10.55563 | Open Source |
| *Mustela nivalis* | Native | 50.911572 | 6.720543 | Open Source |
| *Mustela nivalis* | Native | 52.854805 | 13.708167 | Open Source |
| *Mustela nivalis* | Native | 50.936539 | 11.610897 | Open Source |
| *Mustela nivalis* | Native | 51.416874 | 7.229004001 | Open Source |
| *Mustela nivalis* | Native | 49.862221 | 8.384972 | Open Source |
| *Mustela nivalis* | Native | 50.732349 | 11.439039 | Open Source |
| *Mustela nivalis* | Native | 50.84668 | 6.158245 | Open Source |
| *Mustela nivalis* | Native | 53.184547 | 8.247764 | Open Source |
| *Mustela nivalis* | Native | 50.89724 | 8.835175 | Open Source |
| *Mustela nivalis* | Native | 51.310913 | 6.250126 | Open Source |
| *Mustela nivalis* | Native | 53.716068 | 10.019603 | Open Source |
| *Mustela nivalis* | Native | 51.583645 | 6.509604 | Open Source |
| *Mustela nivalis* | Native | 48.849197 | 9.403396 | Open Source |
| *Mustela nivalis* | Native | 47.429482 | 11.297224 | Open Source |
| *Mustela nivalis* | Native | 53.591419 | 9.652898001 | Open Source |
| *Mustela nivalis* | Native | 50.991333 | 14.190014 | Open Source |
| *Mustela nivalis* | Native | 48.151638 | 11.594277 | Open Source |
| *Mustela nivalis* | Native | 48.85112 | 8.555293 | Open Source |
| *Mustela nivalis* | Native | 50.012154 | 7.008591 | Open Source |
| *Mustela nivalis* | Native | 49.267357 | 8.516893 | Open Source |
| *Mustela nivalis* | Native | 52.360828 | 9.808314 | Open Source |
| *Mustela nivalis* | Native | 50.820686 | 7.920971 | Open Source |
| *Mustela nivalis* | Native | 48.039787 | 7.622658001 | Open Source |
| *Mustela nivalis* | Native | 51.328018 | 7.556979 | Open Source |
| *Mustela nivalis* | Native | 47.347088 | 11.84981 | Open Source |
| *Mustela nivalis* | Native | 50.107548 | 8.569528999 | Open Source |
| *Mustela nivalis* | Native | 50.849468 | 6.157537 | Open Source |
| *Mustela nivalis* | Native | 49.895519 | 7.67601 | Open Source |
| *Mustela nivalis* | Native | 51.887882 | 11.081933 | Open Source |
| *Mustela nivalis* | Native | 50.645092 | 11.19921 | Open Source |
| *Mustela nivalis* | Native | 49.697365 | 8.836311999 | Open Source |
| *Mustela nivalis* | Native | 49.721851 | 9.23004 | Open Source |
| *Mustela nivalis* | Native | 52.360828 | 9.808314 | Open Source |
| *Mustela nivalis* | Native | 50.041344 | 10.241849 | Open Source |
| *Mustela nivalis* | Native | 49.833015 | 8.496841 | Open Source |
| *Mustela nivalis* | Native | 52.365669 | 9.788797 | Open Source |
| *Mustela nivalis* | Native | 47.844635 | 12.120605 | Open Source |
| *Mustela nivalis* | Native | 49.021549 | 8.844917 | Open Source |
| *Mustela nivalis* | Native | 52.565296 | 13.513433 | Open Source |
| *Mustela nivalis* | Native | 47.6217 | 16.772305 | Open Source |
| *Mustela nivalis* | Native | 53.904415 | 10.721283 | Open Source |
| *Mustela nivalis* | Native | 51.820606 | 7.184544001 | Open Source |
| *Mustela nivalis* | Native | 49.7658 | 8.576889 | Open Source |
| *Mustela nivalis* | Native | 49.964638 | 10.183897 | Open Source |
| *Mustela nivalis* | Native | 50.147511 | 10.736003 | Open Source |
| *Mustela nivalis* | Native | 50.43055 | 8.655005 | Open Source |
| *Mustela nivalis* | Native | 49.993477 | 10.254885 | Open Source |
| *Mustela nivalis* | Native | 53.552036 | 9.258986 | Open Source |
| *Mustela nivalis* | Native | 49.48204 | 7.627462 | Open Source |
| *Mustela nivalis* | Native | 49.964638 | 10.183897 | Open Source |
| *Mustela nivalis* | Native | 51.182911 | 8.933516 | Open Source |
| *Mustela nivalis* | Native | 54.52808 | 8.872404 | Open Source |
| *Mustela nivalis* | Native | 52.975502 | 10.48673 | Open Source |
| *Mustela nivalis* | Native | 48.717842 | 9.357795 | Open Source |
| *Mustela nivalis* | Native | 53.184547 | 8.247764 | Open Source |
| *Mustela nivalis* | Native | 49.8008 | 7.508278999 | Open Source |
| *Mustela nivalis* | Native | 53.184547 | 8.247764 | Open Source |
| *Mustela nivalis* | Native | 50.021805 | 10.257239 | Open Source |
| *Mustela nivalis* | Native | 47.347088 | 11.84981 | Open Source |
| *Mustela nivalis* | Native | 51.929703 | 8.113472 | Open Source |
| *Mustela nivalis* | Native | 50.570644 | 6.437473 | Open Source |
| *Mustela nivalis* | Native | 52.358829 | 12.687235 | Open Source |
| *Mustela nivalis* | Native | 51.821297 | 7.143517 | Open Source |
| *Mustela nivalis* | Native | 54.236942 | 11.064949 | Open Source |
| *Mustela nivalis* | Native | 52.360828 | 9.808314 | Open Source |
| *Mustela nivalis* | Native | 41.551852 | 14.368387 | Open Source |
| *Mustela nivalis* | Native | 52.3378 | -2.187 | Open Source |
| *Mustela nivalis* | Native | 52.90538 | -1.22087 | Open Source |
| *Mustela nivalis* | Native | 55.6201 | -4.1995 | Open Source |
| *Mustela nivalis* | Native | 52.687 | -1.150299999 | Open Source |
| *Mustela nivalis* | Native | 53.3429 | -0.9656 | Open Source |
| *Mustela nivalis* | Native | 54.61654 | -1.66627 | Open Source |
| *Mustela nivalis* | Native | 51.96073 | -2.66868 | Open Source |
| *Mustela nivalis* | Native | 52.20579 | -0.24316 | Open Source |
| *Mustela nivalis* | Native | 53.96847 | -0.717180001 | Open Source |
| *Mustela nivalis* | Native | 53.4428 | -2.5058 | Open Source |
| *Mustela nivalis* | Native | 53.7445 | -0.58374 | Open Source |
| *Mustela nivalis* | Native | 51.67622 | -0.50671 | Open Source |
| *Mustela nivalis* | Native | 52.38525 | 0.228970001 | Open Source |
| *Mustela nivalis* | Native | 52.07951 | -2.650029999 | Open Source |
| *Mustela nivalis* | Native | 53.51778 | -0.142589999 | Open Source |
| *Mustela nivalis* | Native | 54.67588 | -2.32485 | Open Source |
| *Mustela nivalis* | Native | 52.34105 | -1.51925 | Open Source |
| *Mustela nivalis* | Native | 55.80596 | -4.611110001 | Open Source |
| *Mustela nivalis* | Native | 55.69597 | -4.17481 | Open Source |
| *Mustela nivalis* | Native | 53.53675 | -1.69216 | Open Source |
| *Mustela nivalis* | Native | 52.83654 | 1.02645 | Open Source |
| *Mustela nivalis* | Native | 51.99728 | 0.94362 | Open Source |
| *Mustela nivalis* | Native | 55.94948 | -3.163320001 | Open Source |
| *Mustela nivalis* | Native | 54.94 | -3.1786 | Open Source |
| *Mustela nivalis* | Native | 54.93883 | -1.71277 | Open Source |
| *Mustela nivalis* | Native | 54.8749 | -1.4647 | Open Source |
| *Mustela nivalis* | Native | 52.71695 | 1.648339999 | Open Source |
| *Mustela nivalis* | Native | 51.35529 | -8.06E-02 | Open Source |
| *Mustela nivalis* | Native | 53.29666 | -2.10723 | Open Source |
| *Mustela nivalis* | Native | 52.06273 | -2.3741 | Open Source |
| *Mustela nivalis* | Native | 55.87733 | -3.191529999 | Open Source |
| *Mustela nivalis* | Native | 56.36617 | -5.41375 | Open Source |
| *Mustela nivalis* | Native | 55.95312 | -3.15862 | Open Source |
| *Mustela nivalis* | Native | 55.94411 | -3.16155 | Open Source |
| *Mustela nivalis* | Native | 54.38889 | -2.422670001 | Open Source |
| *Mustela nivalis* | Native | 53.60611 | -1.589649999 | Open Source |
| *Mustela nivalis* | Native | 55.8253 | -3.279700001 | Open Source |
| *Mustela nivalis* | Native | 50.84242 | -0.99226 | Open Source |
| *Mustela nivalis* | Native | 50.66754 | -3.46945 | Open Source |
| *Mustela nivalis* | Native | 54.8837 | -1.385829999 | Open Source |
| *Mustela nivalis* | Native | 56.3596 | -2.816100001 | Open Source |
| *Mustela nivalis* | Native | 52.57994 | 1.274469999 | Open Source |
| *Mustela nivalis* | Native | 51.71352 | -5.01591 | Open Source |
| *Mustela nivalis* | Native | 52.88014 | -1.702030001 | Open Source |
| *Mustela nivalis* | Native | 51.33063 | -0.4917 | Open Source |
| *Mustela nivalis* | Native | 58.58526 | -3.7243 | Open Source |
| *Mustela nivalis* | Native | 50.79895 | -2.78329 | Open Source |
| *Mustela nivalis* | Native | 55.94578 | -3.174410001 | Open Source |
| *Mustela nivalis* | Native | 51.6572 | 0.116510001 | Open Source |
| *Mustela nivalis* | Native | 55.9433 | -3.15192 | Open Source |
| *Mustela nivalis* | Native | 55.95302 | -3.169830001 | Open Source |
| *Mustela nivalis* | Native | 50.9851 | -1.421099999 | Open Source |
| *Mustela nivalis* | Native | 52.83149 | 1.18498 | Open Source |
| *Mustela nivalis* | Native | 52.32393 | -2.16942 | Open Source |
| *Mustela nivalis* | Native | 53.66224 | -1.46501 | Open Source |
| *Mustela nivalis* | Native | 55.950581 | -3.155807999 | Open Source |
| *Mustela nivalis* | Native | 52.3341 | -2.1589 | Open Source |
| *Mustela nivalis* | Native | 54.8548 | -1.4389 | Open Source |
| *Mustela nivalis* | Native | 52.20422 | -2.83476 | Open Source |
| *Mustela nivalis* | Native | 58.61589 | -3.34015 | Open Source |
| *Mustela nivalis* | Native | 53.90266 | -1.089049999 | Open Source |
| *Mustela nivalis* | Native | 55.94944 | -3.16812 | Open Source |
| *Mustela nivalis* | Native | 52.3026 | -1.57246 | Open Source |
| *Mustela nivalis* | Native | 55.94498 | -3.164780001 | Open Source |
| *Mustela nivalis* | Native | 51.74544 | -0.185749999 | Open Source |
| *Mustela nivalis* | Native | 55.94668 | -3.174439999 | Open Source |
| *Mustela nivalis* | Native | 51.69843 | 4.83E-02 | Open Source |
| *Mustela nivalis* | Native | 55.94406 | -3.16636 | Open Source |
| *Mustela nivalis* | Native | 58.62914 | -3.077159999 | Open Source |
| *Mustela nivalis* | Native | 51.06305 | 1.05773 | Open Source |
| *Mustela nivalis* | Native | 51.78475 | -4.047710001 | Open Source |
| *Mustela nivalis* | Native | 56.9653 | -5.8113 | Open Source |
| *Mustela nivalis* | Native | 54.26319 | -2.76374 | Open Source |
| *Mustela nivalis* | Native | 55.94683 | -3.15843 | Open Source |
| *Mustela nivalis* | Native | 51.24439 | -0.562289999 | Open Source |
| *Mustela nivalis* | Native | 55.92541 | -3.14339 | Open Source |
| *Mustela nivalis* | Native | 56.48604 | -3.629529999 | Open Source |
| *Mustela nivalis* | Native | 51.0253 | -2.3425 | Open Source |
| *Mustela nivalis* | Native | 51.5043 | -2.3041 | Open Source |
| *Mustela nivalis* | Native | 52.3638 | -2.61454 | Open Source |
| *Mustela nivalis* | Native | 53.7103 | -2.664 | Open Source |
| *Mustela nivalis* | Native | 51.51903 | -3.247349999 | Open Source |
| *Mustela nivalis* | Native | 53.3454 | -0.9647 | Open Source |
| *Mustela nivalis* | Native | 52.07849 | -1.328010001 | Open Source |
| *Mustela nivalis* | Native | 50.07637 | -5.70172 | Open Source |
| *Mustela nivalis* | Native | 50.82805 | -0.89459 | Open Source |
| *Mustela nivalis* | Native | 54.98974 | -1.780389999 | Open Source |
| *Mustela nivalis* | Native | 55.94406 | -3.16636 | Open Source |
| *Mustela nivalis* | Native | 51.63977 | 0.682989999 | Open Source |
| *Mustela nivalis* | Native | 54.59187 | -3.185419999 | Open Source |
| *Mustela nivalis* | Native | 55.94582 | -3.171210001 | Open Source |
| *Mustela nivalis* | Native | 55.95038 | -3.163339999 | Open Source |
| *Mustela nivalis* | Native | 57.165 | -3.8586 | Open Source |
| *Mustela nivalis* | Native | 52.86239 | -3.67621 | Open Source |
| *Mustela nivalis* | Native | 52.30247 | 0.25789 | Open Source |
| *Mustela nivalis* | Native | 50.99201 | -4.26769 | Open Source |
| *Mustela nivalis* | Native | 55.18377 | -2.53313 | Open Source |
| *Mustela nivalis* | Native | 51.54737 | 0.51022 | Open Source |
| *Mustela nivalis* | Native | 55.94324 | -3.15833 | Open Source |
| *Mustela nivalis* | Native | 56.03739 | -2.85138 | Open Source |
| *Mustela nivalis* | Native | 50.8285 | -3.54123 | Open Source |
| *Mustela nivalis* | Native | 55.4738 | -2.5543 | Open Source |
| *Mustela nivalis* | Native | 54.6157 | -3.2204 | Open Source |
| *Mustela nivalis* | Native | 53.89494 | -1.35476 | Open Source |
| *Mustela nivalis* | Native | 51.56964 | -4.56E-02 | Open Source |
| *Mustela nivalis* | Native | 54.6049 | -1.680300001 | Open Source |
| *Mustela nivalis* | Native | 52.9437 | -0.89502 | Open Source |
| *Mustela nivalis* | Native | 53.620859 | -2.867377 | Open Source |
| *Mustela nivalis* | Native | 52.43492 | -1.62267 | Open Source |
| *Mustela nivalis* | Native | 52.50885 | -2.971660001 | Open Source |
| *Mustela nivalis* | Native | 55.858041 | -3.26355 | Open Source |
| *Mustela nivalis* | Native | 51.67038 | -1.89512 | Open Source |
| *Mustela nivalis* | Native | 54.21691 | -1.310650001 | Open Source |
| *Mustela nivalis* | Native | 55.9504 | -3.161740001 | Open Source |
| *Mustela nivalis* | Native | 54.92988 | -1.72844 | Open Source |
| *Mustela nivalis* | Native | 53.56183 | -3.04092 | Open Source |
| *Mustela nivalis* | Native | 56.45396 | -5.3924 | Open Source |
| *Mustela nivalis* | Native | 53.3626 | -0.925999999 | Open Source |
| *Mustela nivalis* | Native | 53.3015 | -0.765699999 | Open Source |
| *Mustela nivalis* | Native | 53.338638 | -0.965492 | Open Source |
| *Mustela nivalis* | Native | 51.50082 | 0.184899999 | Open Source |
| *Mustela nivalis* | Native | 52.28584 | -1.43482 | Open Source |
| *Mustela nivalis* | Native | 51.73064 | 0.47692 | Open Source |
| *Mustela nivalis* | Native | 50.95859 | -0.104569999 | Open Source |
| *Mustela nivalis* | Native | 50.93635 | -0.26154 | Open Source |
| *Mustela nivalis* | Native | 52.5914 | -1.7021 | Open Source |
| *Mustela nivalis* | Native | 52.29775 | -0.24718 | Open Source |
| *Mustela nivalis* | Native | 52.08378 | 1.2307 | Open Source |
| *Mustela nivalis* | Native | 51.9481 | 1.45E-02 | Open Source |
| *Mustela nivalis* | Native | 54.03846 | -2.77031 | Open Source |
| *Mustela nivalis* | Native | 54.49674 | -1.57615 | Open Source |
| *Mustela nivalis* | Native | 55.80596 | -4.611110001 | Open Source |
| *Mustela nivalis* | Native | 55.94405 | -3.167960001 | Open Source |
| *Mustela nivalis* | Native | 50.8731 | -0.411 | Open Source |
| *Mustela nivalis* | Native | 53.61187 | 0.14424 | Open Source |
| *Mustela nivalis* | Native | 56.05404 | -3.73563 | Open Source |
| *Mustela nivalis* | Native | 53.4283 | -1.943 | Open Source |
| *Mustela nivalis* | Native | 53.112 | -0.973 | Open Source |
| *Mustela nivalis* | Native | 52.60428 | -1.754110001 | Open Source |
| *Mustela nivalis* | Native | 50.93175 | -6.38E-02 | Open Source |
| *Mustela nivalis* | Native | 55.94138 | -3.16468 | Open Source |
| *Mustela nivalis* | Native | 52.89953 | 0.503679999 | Open Source |
| *Mustela nivalis* | Native | 53.59217 | -2.526470001 | Open Source |
| *Mustela nivalis* | Native | 51.9693 | 1.23993 | Open Source |
| *Mustela nivalis* | Native | 55.946 | -3.152 | Open Source |
| *Mustela nivalis* | Native | 53.28972 | -3.809170001 | Open Source |
| *Mustela nivalis* | Native | 51.0334 | -1.1199 | Open Source |
| *Mustela nivalis* | Native | 54.67879 | -1.3013 | Open Source |
| *Mustela nivalis* | Native | 51.93924 | -2.463260001 | Open Source |
| *Mustela nivalis* | Native | 55.10268 | -1.65596 | Open Source |
| *Mustela nivalis* | Native | 54.94781 | -1.712710001 | Open Source |
| *Mustela nivalis* | Native | 57.9536 | -4.2718 | Open Source |
| *Mustela nivalis* | Native | 51.575331 | 0.252459 | Open Source |
| *Mustela nivalis* | Native | 53.05144 | -2.78093 | Open Source |
| *Mustela nivalis* | Native | 54.9688 | -1.6047 | Open Source |
| *Mustela nivalis* | Native | 52.55243 | -1.546440001 | Open Source |
| *Mustela nivalis* | Native | 55.94324 | -3.15833 | Open Source |
| *Mustela nivalis* | Native | 53.18939 | -1.28383 | Open Source |
| *Mustela nivalis* | Native | 53.63475 | -2.44231 | Open Source |
| *Mustela nivalis* | Native | 50.90705 | -1.244199999 | Open Source |
| *Mustela nivalis* | Native | 55.95125 | -3.166570001 | Open Source |
| *Mustela nivalis* | Native | 52.96873 | 0.52629 | Open Source |
| *Mustela nivalis* | Native | 51.54789 | -4.21591 | Open Source |
| *Mustela nivalis* | Native | 54.20959 | -0.39921 | Open Source |
| *Mustela nivalis* | Native | 50.97951 | -2.284130001 | Open Source |
| *Mustela nivalis* | Native | 54.92972 | -1.66602 | Open Source |
| *Mustela nivalis* | Native | 52.96629 | -1.64635 | Open Source |
| *Mustela nivalis* | Native | 55.94227 | -3.1663 | Open Source |
| *Mustela nivalis* | Native | 52.98752 | -0.969880001 | Open Source |
| *Mustela nivalis* | Native | 51.0304 | -1.1134 | Open Source |
| *Mustela nivalis* | Native | 54.5343 | -3.435600001 | Open Source |
| *Mustela nivalis* | Native | 54.5684 | -1.2642 | Open Source |
| *Mustela nivalis* | Native | 53.76499 | -2.03713 | Open Source |
| *Mustela nivalis* | Native | 50.92199 | -3.474600001 | Open Source |
| *Mustela nivalis* | Native | 54.45049 | -3.07106 | Open Source |
| *Mustela nivalis* | Native | 50.66753 | -2.525619999 | Open Source |
| *Mustela nivalis* | Native | 53.92714 | -0.41689 | Open Source |
| *Mustela nivalis* | Native | 52.026 | 0.18417 | Open Source |
| *Mustela nivalis* | Native | 52.3294 | 0.931230001 | Open Source |
| *Mustela nivalis* | Native | 53.338638 | -0.965492 | Open Source |
| *Mustela nivalis* | Native | 52.31849 | -0.95027 | Open Source |
| *Mustela nivalis* | Native | 52.23977 | -1.60236 | Open Source |
| *Mustela nivalis* | Native | 56.03739 | -2.85138 | Open Source |
| *Mustela nivalis* | Native | 51.67788 | -1.20E-02 | Open Source |
| *Mustela nivalis* | Native | 51.97408 | -3.32255 | Open Source |
| *Mustela nivalis* | Native | 50.97951 | -2.284130001 | Open Source |
| *Mustela nivalis* | Native | 54.6431 | -1.182700001 | Open Source |
| *Mustela nivalis* | Native | 55.01779 | -1.53001 | Open Source |
| *Mustela nivalis* | Native | 50.97951 | -2.28555 | Open Source |
| *Mustela nivalis* | Native | 55.93882 | -3.15019 | Open Source |
| *Mustela nivalis* | Native | 52.10816 | -2.489829999 | Open Source |
| *Mustela nivalis* | Native | 53.88909 | -1.35257 | Open Source |
| *Mustela nivalis* | Native | 53.2825 | -0.8905 | Open Source |
| *Mustela nivalis* | Native | 55.94857 | -3.164890001 | Open Source |
| *Mustela nivalis* | Native | 51.3851 | -2.59408 | Open Source |
| *Mustela nivalis* | Native | 53.1139 | -0.7929 | Open Source |
| *Mustela nivalis* | Native | 51.04518 | -1.24403 | Open Source |
| *Mustela nivalis* | Native | 54.5961 | -1.2221 | Open Source |
| *Mustela nivalis* | Native | 51.3766 | 0.783969999 | Open Source |
| *Mustela nivalis* | Native | 50.76322 | -0.78276 | Open Source |
| *Mustela nivalis* | Native | 53.171 | -0.7756 | Open Source |
| *Mustela nivalis* | Native | 51.3797 | 1.206 | Open Source |
| *Mustela nivalis* | Native | 53.41982 | -1.66291 | Open Source |
| *Mustela nivalis* | Native | 52.72349 | -2.842200001 | Open Source |
| *Mustela nivalis* | Native | 52.00473 | 0.98892 | Open Source |
| *Mustela nivalis* | Native | 50.84532 | -0.914069999 | Open Source |
| *Mustela nivalis* | Native | 55.94324 | -3.15833 | Open Source |
| *Mustela nivalis* | Native | 52.90923 | -0.68772 | Open Source |
| *Mustela nivalis* | Native | 54.37621 | -1.35109 | Open Source |
| *Mustela nivalis* | Native | 52.5098 | 1.1339 | Open Source |
| *Mustela nivalis* | Native | 50.84276 | -0.590290001 | Open Source |
| *Mustela nivalis* | Native | 52.3659 | -1.5425 | Open Source |
| *Mustela nivalis* | Native | 50.21328 | -5.2777 | Open Source |
| *Mustela nivalis* | Native | 51.37342 | -0.5252 | Open Source |
| *Mustela nivalis* | Native | 56.4816 | -3.142199999 | Open Source |
| *Mustela nivalis* | Native | 52.58065 | -3.08836 | Open Source |
| *Mustela nivalis* | Native | 50.94377 | -0.217129999 | Open Source |
| *Mustela nivalis* | Native | 55.94227 | -3.1663 | Open Source |
| *Mustela nivalis* | Native | 56.12997 | -5.485339999 | Open Source |
| *Mustela nivalis* | Native | 50.98938 | 0.47522 | Open Source |
| *Mustela nivalis* | Native | 51.88288 | -1.399210001 | Open Source |
| *Mustela nivalis* | Native | 51.28941 | 0.236519999 | Open Source |
| *Mustela nivalis* | Native | 53.66335 | -2.13995 | Open Source |
| *Mustela nivalis* | Native | 56.48348 | -3.13252 | Open Source |
| *Mustela nivalis* | Native | 52.8102 | -0.8709 | Open Source |
| *Mustela nivalis* | Native | 55.9449 | -3.17279 | Open Source |
| *Mustela nivalis* | Native | 53.59873 | -1.43484 | Open Source |
| *Mustela nivalis* | Native | 51.51138 | -3.68018 | Open Source |
| *Mustela nivalis* | Native | 54.30664 | -1.458230001 | Open Source |
| *Mustela nivalis* | Native | 55.0139 | -1.5403 | Open Source |
| *Mustela nivalis* | Native | 55.9959 | -5.4572 | Open Source |
| *Mustela nivalis* | Native | 51.44923 | -2.01794 | Open Source |
| *Mustela nivalis* | Native | 51.16248 | 0.86117 | Open Source |
| *Mustela nivalis* | Native | 50.90794 | -0.286779999 | Open Source |
| *Mustela nivalis* | Native | 55.94237 | -3.155100001 | Open Source |
| *Mustela nivalis* | Native | 53.4927 | -2.538500001 | Open Source |
| *Mustela nivalis* | Native | 55.94237 | -3.155100001 | Open Source |
| *Mustela nivalis* | Native | 53.77895 | -0.46117 | Open Source |
| *Mustela nivalis* | Native | 52.08774 | 0.61855 | Open Source |
| *Mustela nivalis* | Native | 51.1994 | -2.1405 | Open Source |
| *Mustela nivalis* | Native | 53.31699 | -1.69299 | Open Source |
| *Mustela nivalis* | Native | 54.32872 | -2.33987 | Open Source |
| *Mustela nivalis* | Native | 51.57056 | -4.70E-02 | Open Source |
| *Mustela nivalis* | Native | 54.48984 | -2.329550001 | Open Source |
| *Mustela nivalis* | Native | 53.24422 | -1.7085 | Open Source |
| *Mustela nivalis* | Native | 51.301 | -2.1546 | Open Source |
| *Mustela nivalis* | Native | 53.24771 | -1.668009999 | Open Source |
| *Mustela nivalis* | Native | 54.10843 | -0.46506 | Open Source |
| *Mustela nivalis* | Native | 51.65455 | -1.25045 | Open Source |
| *Mustela nivalis* | Native | 52.6134 | -1.0441 | Open Source |
| *Mustela nivalis* | Native | 52.7771 | -0.9147 | Open Source |
| *Mustela nivalis* | Native | 53.2567 | -0.572899999 | Open Source |
| *Mustela nivalis* | Native | 53.31099 | -3.046820001 | Open Source |
| *Mustela nivalis* | Native | 54.6011 | -1.219000001 | Open Source |
| *Mustela nivalis* | Native | 51.02563 | -3.03442 | Open Source |
| *Mustela nivalis* | Native | 53.05859 | -0.96312 | Open Source |
| *Mustela nivalis* | Native | 52.34259 | -1.45905 | Open Source |
| *Mustela nivalis* | Native | 52.09614 | 0.27484 | Open Source |
| *Mustela nivalis* | Native | 55.94851 | -3.17129 | Open Source |
| *Mustela nivalis* | Native | 50.88547 | 0.195789999 | Open Source |
| *Mustela nivalis* | Native | 53.5866 | -2.864790001 | Open Source |
| *Mustela nivalis* | Native | 51.64553 | -4.78298 | Open Source |
| *Mustela nivalis* | Native | 53.23204 | -0.540030001 | Open Source |
| *Mustela nivalis* | Native | 53.76292 | -2.992470001 | Open Source |
| *Mustela nivalis* | Native | 54.4978 | -2.8289 | Open Source |
| *Mustela nivalis* | Native | 55.94052 | -3.16145 | Open Source |
| *Mustela nivalis* | Native | 52.53348 | -2.46949 | Open Source |
| *Mustela nivalis* | Native | 55.01779 | -1.53001 | Open Source |
| *Mustela nivalis* | Native | 50.32145 | -4.824279999 | Open Source |
| *Mustela nivalis* | Native | 50.74916 | -3.07793 | Open Source |
| *Mustela nivalis* | Native | 53.2724 | -1.9467 | Open Source |
| *Mustela nivalis* | Native | 51.8599 | 1.09406 | Open Source |
| *Mustela nivalis* | Native | 52.267 | -2.130699999 | Open Source |
| *Mustela nivalis* | Native | 51.0805 | -0.945400001 | Open Source |
| *Mustela nivalis* | Native | 50.9623 | -0.21422 | Open Source |
| *Mustela nivalis* | Native | 51.40841 | 0.43595 | Open Source |
| *Mustela nivalis* | Native | 55.17325 | -2.025879999 | Open Source |
| *Mustela nivalis* | Native | 55.95131 | -3.16017 | Open Source |
| *Mustela nivalis* | Native | 54.91156 | -1.60378 | Open Source |
| *Mustela nivalis* | Native | 52.4847 | -1.1746 | Open Source |
| *Mustela nivalis* | Native | 52.90741 | -2.35086 | Open Source |
| *Mustela nivalis* | Native | 51.0488 | -1.5182 | Open Source |
| *Mustela nivalis* | Native | 55.94044 | -3.16945 | Open Source |
| *Mustela nivalis* | Native | 54.11908 | -0.45395 | Open Source |
| *Mustela nivalis* | Native | 55.94311 | -3.17273 | Open Source |
| *Mustela nivalis* | Native | 55.58053 | -3.42669 | Open Source |
| *Mustela nivalis* | Native | 51.70145 | -3.5577 | Open Source |
| *Mustela nivalis* | Native | 51.54659 | 0.50441 | Open Source |
| *Mustela nivalis* | Native | 51.20659 | 1.17319 | Open Source |
| *Mustela nivalis* | Native | 51.2785 | -0.29024 | Open Source |
| *Mustela nivalis* | Native | 53.237933 | -2.607864 | Open Source |
| *Mustela nivalis* | Native | 55.78934 | -2.27665 | Open Source |
| *Mustela nivalis* | Native | 53.91057 | -0.76767 | Open Source |
| *Mustela nivalis* | Native | 51.96992 | -0.28443 | Open Source |
| *Mustela nivalis* | Native | 54.91171 | -1.650570001 | Open Source |
| *Mustela nivalis* | Native | 53.51869 | -2.09271 | Open Source |
| *Mustela nivalis* | Native | 55.94775 | -3.15686 | Open Source |
| *Mustela nivalis* | Native | 51.6296 | -2.1323 | Open Source |
| *Mustela nivalis* | Native | 52.06639 | -2.579820001 | Open Source |
| *Mustela nivalis* | Native | 52.8817 | 1.1245 | Open Source |
| *Mustela nivalis* | Native | 56.51245 | -3.204879999 | Open Source |
| *Mustela nivalis* | Native | 51.44713 | -0.75596 | Open Source |
| *Mustela nivalis* | Native | 51.63828 | -1.390889999 | Open Source |
| *Mustela nivalis* | Native | 51.54701 | 0.60677 | Open Source |
| *Mustela nivalis* | Native | 53.92465 | -1.146440001 | Open Source |
| *Mustela nivalis* | Native | 52.37202 | 1.07979 | Open Source |
| *Mustela nivalis* | Native | 51.327 | -3.0273 | Open Source |
| *Mustela nivalis* | Native | 53.08968 | -2.530769999 | Open Source |
| *Mustela nivalis* | Native | 50.82027 | -3.07105 | Open Source |
| *Mustela nivalis* | Native | 53.45165 | -1.6333 | Open Source |
| *Mustela nivalis* | Native | 51.01374 | -1.4718 | Open Source |
| *Mustela nivalis* | Native | 51.83957 | 0.47128 | Open Source |
| *Mustela nivalis* | Native | 53.23369 | -2.48611 | Open Source |
| *Mustela nivalis* | Native | 52.86406 | -2.55601 | Open Source |
| *Mustela nivalis* | Native | 52.1377 | -1.10E-02 | Open Source |
| *Mustela nivalis* | Native | 53.37445 | -0.81777 | Open Source |
| *Mustela nivalis* | Native | 51.99058 | -1.702869999 | Open Source |
| *Mustela nivalis* | Native | 51.63416 | -0.45318 | Open Source |
| *Mustela nivalis* | Native | 53.07207 | -0.543959999 | Open Source |
| *Mustela nivalis* | Native | 51.88722 | 0.42884 | Open Source |
| *Mustela nivalis* | Native | 50.77784 | -3.31523 | Open Source |
| *Mustela nivalis* | Native | 50.43741 | -5.04104 | Open Source |
| *Mustela nivalis* | Native | 52.3624 | -1.5594 | Open Source |
| *Mustela nivalis* | Native | 52.5752 | -1.7394 | Open Source |
| *Mustela nivalis* | Native | 52.38905 | -1.615709999 | Open Source |
| *Mustela nivalis* | Native | 53.84854 | -2.1056 | Open Source |
| *Mustela nivalis* | Native | 55.94411 | -3.16155 | Open Source |
| *Mustela nivalis* | Native | 52.07218 | 1.10544 | Open Source |
| *Mustela nivalis* | Native | 53.28519 | -1.738969999 | Open Source |
| *Mustela nivalis* | Native | 55.0805 | -4.130300001 | Open Source |
| *Mustela nivalis* | Native | 54.07946 | -0.38053 | Open Source |
| *Mustela nivalis* | Native | 52.7534 | -0.7884 | Open Source |
| *Mustela nivalis* | Native | 52.52579 | 1.3132 | Open Source |
| *Mustela nivalis* | Native | 55.94504 | -3.158380001 | Open Source |
| *Mustela nivalis* | Native | 54.14565 | -0.169719999 | Open Source |
| *Mustela nivalis* | Native | 52.00211 | 1.25687 | Open Source |
| *Mustela nivalis* | Native | 54.09892 | -0.49447 | Open Source |
| *Mustela nivalis* | Native | 52.8979 | -1.230499999 | Open Source |
| *Mustela nivalis* | Native | 57.4075 | -2.4516 | Open Source |
| *Mustela nivalis* | Native | 54.5377 | -2.764600001 | Open Source |
| *Mustela nivalis* | Native | 52.91862 | -1.53405 | Open Source |
| *Mustela nivalis* | Native | 54.28223 | -1.427829999 | Open Source |
| *Mustela nivalis* | Native | 51.58373 | -1.70047 | Open Source |
| *Mustela nivalis* | Native | 52.48208 | 1.066860001 | Open Source |
| *Mustela nivalis* | Native | 50.78486 | -2.77922 | Open Source |
| *Mustela nivalis* | Native | 58.5642 | -3.61667 | Open Source |
| *Mustela nivalis* | Native | 54.21766 | -0.502419999 | Open Source |
| *Mustela nivalis* | Native | 54.51231 | -1.67175 | Open Source |
| *Mustela nivalis* | Native | 53.52412 | -1.98563 | Open Source |
| *Mustela nivalis* | Native | 55.90628 | -2.905290001 | Open Source |
| *Mustela nivalis* | Native | 51.1138 | 0.5198 | Open Source |
| *Mustela nivalis* | Native | 52.08758 | -2.06344 | Open Source |
| *Mustela nivalis* | Native | 52.88415 | -0.69591 | Open Source |
| *Mustela nivalis* | Native | 55.94859 | -3.16329 | Open Source |
| *Mustela nivalis* | Native | 53.44315 | -1.51745 | Open Source |
| *Mustela nivalis* | Native | 53.0797 | -2.528200001 | Open Source |
| *Mustela nivalis* | Native | 52.07207 | -3.323980001 | Open Source |
| *Mustela nivalis* | Native | 51.12362 | -1.834910001 | Open Source |
| *Mustela nivalis* | Native | 55.86122 | -3.18464 | Open Source |
| *Mustela nivalis* | Native | 51.25364 | 0.734969999 | Open Source |
| *Mustela nivalis* | Native | 52.96158 | 1.02476 | Open Source |
| *Mustela nivalis* | Native | 51.28955 | 3.14E-02 | Open Source |
| *Mustela nivalis* | Native | 52.91716 | -1.16787 | Open Source |
| *Mustela nivalis* | Native | 52.88971 | 1.19073 | Open Source |
| *Mustela nivalis* | Native | 53.2929 | -0.9173 | Open Source |
| *Mustela nivalis* | Native | 51.2637 | -0.1792 | Open Source |
| *Mustela nivalis* | Native | 52.08932 | 1.568400001 | Open Source |
| *Mustela nivalis* | Native | 50.80975 | -0.46922 | Open Source |
| *Mustela nivalis* | Native | 55.94403 | -3.169560001 | Open Source |
| *Mustela nivalis* | Native | 55.94403 | -3.169560001 | Open Source |
| *Mustela nivalis* | Native | 51.37461 | -0.875740001 | Open Source |
| *Mustela nivalis* | Native | 55.03019 | -1.486059999 | Open Source |
| *Mustela nivalis* | Native | 56.60271 | -2.675120001 | Open Source |
| *Mustela nivalis* | Native | 55.94227 | -3.1663 | Open Source |
| *Mustela nivalis* | Native | 52.63871 | 0.16432 | Open Source |
| *Mustela nivalis* | Native | 51.87081 | -2.16626 | Open Source |
| *Mustela nivalis* | Native | 58.44654 | -3.10926 | Open Source |
| *Mustela nivalis* | Native | 54.93202 | -1.685509999 | Open Source |
| *Mustela nivalis* | Native | 57.3968 | -2.0992 | Open Source |
| *Mustela nivalis* | Native | 54.6038 | -1.217199999 | Open Source |
| *Mustela nivalis* | Native | 53.45026 | -1.49779 | Open Source |
| *Mustela nivalis* | Native | 51.45025 | -0.557270001 | Open Source |
| *Mustela nivalis* | Native | 56.24874 | -4.668639999 | Open Source |
| *Mustela nivalis* | Native | 55.95127 | -3.16497 | Open Source |
| *Mustela nivalis* | Native | 51.46631 | 0.48828 | Open Source |
| *Mustela nivalis* | Native | 54.3073 | -0.844700001 | Open Source |
| *Mustela nivalis* | Native | 51.69111 | -2.64879 | Open Source |
| *Mustela nivalis* | Native | 50.94041 | -0.49902 | Open Source |
| *Mustela nivalis* | Native | 55.94522 | -3.15318 | Open Source |
| *Mustela nivalis* | Native | 54.16126 | -0.67446 | Open Source |
| *Mustela nivalis* | Native | 52.59999 | 1.4547 | Open Source |
| *Mustela nivalis* | Native | 50.3583 | -4.265700001 | Open Source |
| *Mustela nivalis* | Native | 54.3073 | -0.55604 | Open Source |
| *Mustela nivalis* | Native | 53.3627 | -0.9237 | Open Source |
| *Mustela nivalis* | Native | 53.3075 | -3.46396 | Open Source |
| *Mustela nivalis* | Native | 50.9803 | -1.08258 | Open Source |
| *Mustela nivalis* | Native | 54.5635 | -1.6765 | Open Source |
| *Mustela nivalis* | Native | 51.3532 | 0.5561 | Open Source |
| *Mustela nivalis* | Native | 51.22064 | -1.778719999 | Open Source |
| *Mustela nivalis* | Native | 55.92789 | -2.83458 | Open Source |
| *Mustela nivalis* | Native | 55.93912 | -3.11818 | Open Source |
| *Mustela nivalis* | Native | 53.98537 | -1.21993 | Open Source |
| *Mustela nivalis* | Native | 51.06615 | -2.08201 | Open Source |
| *Mustela nivalis* | Native | 54.02279 | -1.0498 | Open Source |
| *Mustela nivalis* | Native | 56.0067 | -2.87156 | Open Source |
| *Mustela nivalis* | Native | 51.00214 | -1.49331 | Open Source |
| *Mustela nivalis* | Native | 54.11131 | -1.395 | Open Source |
| *Mustela nivalis* | Native | 54.58051 | -1.63562 | Open Source |
| *Mustela nivalis* | Native | 55.93959 | -3.16462 | Open Source |
| *Mustela nivalis* | Native | 52.509 | -3.6329 | Open Source |
| *Mustela nivalis* | Native | 53.71463 | -2.09466 | Open Source |
| *Mustela nivalis* | Native | 52.13116 | -1.65884 | Open Source |
| *Mustela nivalis* | Native | 53.2762 | -1.73903 | Open Source |
| *Mustela nivalis* | Native | 54.16257 | -1.24264 | Open Source |
| *Mustela nivalis* | Native | 54.6303 | -3.3169 | Open Source |
| *Mustela nivalis* | Native | 52.281 | -1.4099 | Open Source |
| *Mustela nivalis* | Native | 51.88893 | -4.10621 | Open Source |
| *Mustela nivalis* | Native | 55.94411 | -3.16155 | Open Source |
| *Mustela nivalis* | Native | 53.28301 | -2.51367 | Open Source |
| *Mustela nivalis* | Native | 51.09261 | 0.40982 | Open Source |
| *Mustela nivalis* | Native | 51.41023 | -2.28248 | Open Source |
| *Mustela nivalis* | Native | 53.69334 | -0.586139999 | Open Source |
| *Mustela nivalis* | Native | 51.49092 | -2.74713 | Open Source |
| *Mustela nivalis* | Native | 52.8058 | -1.0007 | Open Source |
| *Mustela nivalis* | Native | 52.40325 | -3.020779999 | Open Source |
| *Mustela nivalis* | Native | 52.32091 | 0.646970001 | Open Source |
| *Mustela nivalis* | Native | 50.81863 | 0.16284 | Open Source |
| *Mustela nivalis* | Native | 51.8836 | 0.4301 | Open Source |
| *Mustela nivalis* | Native | 55.94853 | -3.169690001 | Open Source |
| *Mustela nivalis* | Native | 54.35831 | -2.63317 | Open Source |
| *Mustela nivalis* | Native | 52.01568 | 1.08731 | Open Source |
| *Mustela nivalis* | Native | 51.0938 | -0.3236 | Open Source |
| *Mustela nivalis* | Native | 52.90113 | -0.77122 | Open Source |
| *Mustela nivalis* | Native | 55.2021 | -1.6103 | Open Source |
| *Mustela nivalis* | Native | 52.20837 | 1.528159999 | Open Source |
| *Mustela nivalis* | Native | 50.82166 | -2.789490001 | Open Source |
| *Mustela nivalis* | Native | 50.93826 | -3.45397 | Open Source |
| *Mustela nivalis* | Native | 51.07661 | 0.356160001 | Open Source |
| *Mustela nivalis* | Native | 52.01568 | 1.08731 | Open Source |
| *Mustela nivalis* | Native | 57.59383 | -3.559990001 | Open Source |
| *Mustela nivalis* | Native | 51.25412 | -1.9849 | Open Source |
| *Mustela nivalis* | Native | 52.22865 | -1.84842 | Open Source |
| *Mustela nivalis* | Native | 53.76073 | -1.23016 | Open Source |
| *Mustela nivalis* | Native | 52.30693 | -1.906820001 | Open Source |
| *Mustela nivalis* | Native | 55.6305 | -2.3748 | Open Source |
| *Mustela nivalis* | Native | 51.59807 | -1.1431 | Open Source |
| *Mustela nivalis* | Native | 52.00181 | 1.256629999 | Open Source |
| *Mustela nivalis* | Native | 57.59767 | -3.61512 | Open Source |
| *Mustela nivalis* | Native | 51.05014 | -3.36311 | Open Source |
| *Mustela nivalis* | Native | 52.3294 | 1.674200001 | Open Source |
| *Mustela nivalis* | Native | 52.71542 | 0.214049999 | Open Source |
| *Mustela nivalis* | Native | 54.38244 | -2.014590001 | Open Source |
| *Mustela nivalis* | Native | 52.32302 | -2.17088 | Open Source |
| *Mustela nivalis* | Native | 52.66933 | -2.44433 | Open Source |
| *Mustela nivalis* | Native | 54.70307 | -1.48245 | Open Source |
| *Mustela nivalis* | Native | 57.28605 | -2.553210001 | Open Source |
| *Mustela nivalis* | Native | 58.58509 | -3.669240001 | Open Source |
| *Mustela nivalis* | Native | 54.40659 | -2.17174 | Open Source |
| *Mustela nivalis* | Native | 55.94146 | -3.15667 | Open Source |
| *Mustela nivalis* | Native | 56.07279 | -3.30832 | Open Source |
| *Mustela nivalis* | Native | 55.83933 | -4.51911 | Open Source |
| *Mustela nivalis* | Native | 55.95139 | -3.15216 | Open Source |
| *Mustela nivalis* | Native | 50.74756 | 0.258790001 | Open Source |
| *Mustela nivalis* | Native | 52.61318 | -1.151480001 | Open Source |
| *Mustela nivalis* | Native | 56.05387 | -3.73514 | Open Source |
| *Mustela nivalis* | Native | 54.55793 | -0.78998 | Open Source |
| *Mustela nivalis* | Native | 53.359 | -1.5528 | Open Source |
| *Mustela nivalis* | Native | 55.94403 | -3.169560001 | Open Source |
| *Mustela nivalis* | Native | 55.7503 | -3.9243 | Open Source |
| *Mustela nivalis* | Native | 51.81921 | -5.033130001 | Open Source |
| *Mustela nivalis* | Native | 53.5262 | -2.639 | Open Source |
| *Mustela nivalis* | Native | 55.94311 | -3.17273 | Open Source |
| *Mustela nivalis* | Native | 52.66374 | -1.82475 | Open Source |
| *Mustela nivalis* | Native | 53.40149 | -0.9208 | Open Source |
| *Mustela nivalis* | Native | 54.00478 | -1.30806 | Open Source |
| *Mustela nivalis* | Native | 56.2203 | -3.398 | Open Source |
| *Mustela nivalis* | Native | 52.93283 | -1.22559 | Open Source |
| *Mustela nivalis* | Native | 55.01779 | -1.53001 | Open Source |
| *Mustela nivalis* | Native | 55.9433 | -3.15192 | Open Source |
| *Mustela nivalis* | Native | 55.94228 | -3.1647 | Open Source |
| *Mustela nivalis* | Native | 58.50454 | -3.64654 | Open Source |
| *Mustela nivalis* | Native | 54.69988 | -4.905870001 | Open Source |
| *Mustela nivalis* | Native | 51.96522 | -0.77223 | Open Source |
| *Mustela nivalis* | Native | 52.96867 | 0.612669999 | Open Source |
| *Mustela nivalis* | Native | 52.31575 | -3.19E-02 | Open Source |
| *Mustela nivalis* | Native | 53.3743 | -0.808 | Open Source |
| *Mustela nivalis* | Native | 55.0014 | -2.70111 | Open Source |
| *Mustela nivalis* | Native | 55.03441 | -1.664370001 | Open Source |
| *Mustela nivalis* | Native | 51.52269 | -2.254350001 | Open Source |
| *Mustela nivalis* | Native | 57.33798 | -4.2071 | Open Source |
| *Mustela nivalis* | Native | 52.32596 | -0.791619999 | Open Source |
| *Mustela nivalis* | Native | 56.182 | -3.379860001 | Open Source |
| *Mustela nivalis* | Native | 54.9113 | -1.66695 | Open Source |
| *Mustela nivalis* | Native | 52.37457 | 0.152850001 | Open Source |
| *Mustela nivalis* | Native | 53.32916 | -1.45342 | Open Source |
| *Mustela nivalis* | Native | 51.80009 | -3.437819999 | Open Source |
| *Mustela nivalis* | Native | 52.6668 | -1.3745 | Open Source |
| *Mustela nivalis* | Native | 53.92438 | -0.59289 | Open Source |
| *Mustela nivalis* | Native | 51.62726 | -1.20323 | Open Source |
| *Mustela nivalis* | Native | 52.25204 | -1.857130001 | Open Source |
| *Mustela nivalis* | Native | 52.72725 | 1.649940001 | Open Source |
| *Mustela nivalis* | Native | 55.9414 | -3.16307 | Open Source |
| *Mustela nivalis* | Native | 54.3429 | -2.6568 | Open Source |
| *Mustela nivalis* | Native | 52.45496 | 0.68445 | Open Source |
| *Mustela nivalis* | Native | 51.17944 | -1.899089999 | Open Source |
| *Mustela nivalis* | Native | 56.51286 | -5.372189999 | Open Source |
| *Mustela nivalis* | Native | 58.55221 | -3.6419 | Open Source |
| *Mustela nivalis* | Native | 51.0148 | -1.32923 | Open Source |
| *Mustela nivalis* | Native | 51.99522 | 1.40499 | Open Source |
| *Mustela nivalis* | Native | 53.2886 | -1.6283 | Open Source |
| *Mustela nivalis* | Native | 51.25827 | -2.28154 | Open Source |
| *Mustela nivalis* | Native | 52.25751 | -1.9582 | Open Source |
| *Mustela nivalis* | Native | 52.2459 | 1.611 | Open Source |
| *Mustela nivalis* | Native | 52.36337 | -2.17125 | Open Source |
| *Mustela nivalis* | Native | 52.16722 | 0.13259 | Open Source |
| *Mustela nivalis* | Native | 52.7171 | 0.109399999 | Open Source |
| *Mustela nivalis* | Native | 55.93873 | -3.15979 | Open Source |
| *Mustela nivalis* | Native | 52.33473 | -2.15919 | Open Source |
| *Mustela nivalis* | Native | 54.21485 | -0.42277 | Open Source |
| *Mustela nivalis* | Native | 52.22449 | -2.088400001 | Open Source |
| *Mustela nivalis* | Native | 53.05184 | -0.92359 | Open Source |
| *Mustela nivalis* | Native | 55.178 | -1.6156 | Open Source |
| *Mustela nivalis* | Native | 53.13121 | -0.85584 | Open Source |
| *Mustela nivalis* | Native | 55.01 | -1.535399999 | Open Source |
| *Mustela nivalis* | Native | 56.32314 | -2.70413 | Open Source |
| *Mustela nivalis* | Native | 51.2489 | -1.206200001 | Open Source |
| *Mustela nivalis* | Native | 52.9603 | -1.5533 | Open Source |
| *Mustela nivalis* | Native | 51.4886 | -3.38256 | Open Source |
| *Mustela nivalis* | Native | 53.2871 | -2.009599999 | Open Source |
| *Mustela nivalis* | Native | 50.76938 | -2.427549999 | Open Source |
| *Mustela nivalis* | Native | 51.3334 | -1.37914 | Open Source |
| *Mustela nivalis* | Native | 52.59507 | 1.469079999 | Open Source |
| *Mustela nivalis* | Native | 51.88123 | 1.26992 | Open Source |
| *Mustela nivalis* | Native | 51.80798 | 0.83515 | Open Source |
| *Mustela nivalis* | Native | 51.38006 | 1.21105 | Open Source |
| *Mustela nivalis* | Native | 51.73055 | -0.271780001 | Open Source |
| *Mustela nivalis* | Native | 51.16543 | -0.57619 | Open Source |
| *Mustela nivalis* | Native | 51.09225 | -0.222860001 | Open Source |
| *Mustela nivalis* | Native | 50.87826 | -0.111599999 | Open Source |
| *Mustela nivalis* | Native | 54.9296 | -3.938700001 | Open Source |
| *Mustela nivalis* | Native | 51.7093 | 0.5778 | Open Source |
| *Mustela nivalis* | Native | 55.94326 | -3.15673 | Open Source |
| *Mustela nivalis* | Native | 55.94309 | -3.174330001 | Open Source |
| *Mustela nivalis* | Native | 50.91752 | -0.14843 | Open Source |
| *Mustela nivalis* | Native | 54.91516 | -2.69492 | Open Source |
| *Mustela nivalis* | Native | 56.49062 | -2.745 | Open Source |
| *Mustela nivalis* | Native | 50.75818 | 0.267809999 | Open Source |
| *Mustela nivalis* | Native | 52.7342 | 0.9874 | Open Source |
| *Mustela nivalis* | Native | 51.1484 | 1.243899999 | Open Source |
| *Mustela nivalis* | Native | 53.0514 | -2.4555 | Open Source |
| *Mustela nivalis* | Native | 52.22773 | -1.82793 | Open Source |
| *Mustela nivalis* | Native | 52.55442 | -2.10E-03 | Open Source |
| *Mustela nivalis* | Native | 55.950617 | -3.161448999 | Open Source |
| *Mustela nivalis* | Native | 50.5465 | -3.8921 | Open Source |
| *Mustela nivalis* | Native | 52.46186 | 0.36099 | Open Source |
| *Mustela nivalis* | Native | 53.79823 | -1.92785 | Open Source |
| *Mustela nivalis* | Native | 51.75304 | -0.86347 | Open Source |
| *Mustela nivalis* | Native | 55.78271 | -3.218930001 | Open Source |
| *Mustela nivalis* | Native | 52.79492 | -0.597720001 | Open Source |
| *Mustela nivalis* | Native | 53.92022 | -1.15567 | Open Source |
| *Mustela nivalis* | Native | 50.94167 | -0.49243 | Open Source |
| *Mustela nivalis* | Native | 52.34609 | -2.72587 | Open Source |
| *Mustela nivalis* | Native | 52.02456 | -2.160999999 | Open Source |
| *Mustela nivalis* | Native | 50.74832 | 0.265920001 | Open Source |
| *Mustela nivalis* | Native | 51.30901 | -2.0092 | Open Source |
| *Mustela nivalis* | Native | 53.07198 | -1.99026 | Open Source |
| *Mustela nivalis* | Native | 52.6603 | -0.9919 | Open Source |
| *Mustela nivalis* | Native | 51.64794 | -2.038249999 | Open Source |
| *Mustela nivalis* | Native | 50.72056 | -2.4379 | Open Source |
| *Mustela nivalis* | Native | 54.16136 | -2.80475 | Open Source |
| *Mustela nivalis* | Native | 52.4644 | -1.4904 | Open Source |
| *Mustela nivalis* | Native | 51.69707 | 0.53297 | Open Source |
| *Mustela nivalis* | Native | 54.60713 | -1.5363 | Open Source |
| *Mustela nivalis* | Native | 52.06187 | -3.3514 | Open Source |
| *Mustela nivalis* | Native | 51.55458 | -3.58008 | Open Source |
| *Mustela nivalis* | Native | 52.57642 | 1.17235 | Open Source |
| *Mustela nivalis* | Native | 51.9122 | -0.3321 | Open Source |
| *Mustela nivalis* | Native | 55.95405 | -3.15545 | Open Source |
| *Mustela nivalis* | Native | 51.04924 | -3.36308 | Open Source |
| *Mustela nivalis* | Native | 54.92988 | -1.72844 | Open Source |
| *Mustela nivalis* | Native | 52.54662 | -1.359959999 | Open Source |
| *Mustela nivalis* | Native | 52.20711 | -2.12361 | Open Source |
| *Mustela nivalis* | Native | 51.81658 | -0.66312 | Open Source |
| *Mustela nivalis* | Native | 53.32981 | -1.61858 | Open Source |
| *Mustela nivalis* | Native | 54.99736 | -1.59747 | Open Source |
| *Mustela nivalis* | Native | 58.4144 | -3.738189999 | Open Source |
| *Mustela nivalis* | Native | 56.28838 | -5.2985 | Open Source |
| *Mustela nivalis* | Native | 53.98592 | -1.047589999 | Open Source |
| *Mustela nivalis* | Native | 58.57343 | -3.73575 | Open Source |
| *Mustela nivalis* | Native | 53.20921 | -1.7297 | Open Source |
| *Mustela nivalis* | Native | 51.54659 | 0.50441 | Open Source |
| *Mustela nivalis* | Native | 52.5967 | -1.3259 | Open Source |
| *Mustela nivalis* | Native | 54.67924 | -1.59751 | Open Source |
| *Mustela nivalis* | Native | 52.3627 | 0.3383 | Open Source |
| *Mustela nivalis* | Native | 51.0704 | -0.29577 | Open Source |
| *Mustela nivalis* | Native | 55.93274 | -3.31008 | Open Source |
| *Mustela nivalis* | Native | 52.86789 | -0.69349 | Open Source |
| *Mustela nivalis* | Native | 55.94412 | -3.15995 | Open Source |
| *Mustela nivalis* | Native | 53.06043 | -2.427449999 | Open Source |
| *Mustela nivalis* | Native | 51.17906 | 0.94802 | Open Source |
| *Mustela nivalis* | Native | 52.384 | -2.77277 | Open Source |
| *Mustela nivalis* | Native | 58.41341 | -3.74499 | Open Source |
| *Mustela nivalis* | Native | 53.9962 | -0.79107 | Open Source |
| *Mustela nivalis* | Native | 50.7875 | -0.798100001 | Open Source |
| *Mustela nivalis* | Native | 50.869531 | -0.125900999 | Open Source |
| *Mustela nivalis* | Native | 55.94411 | -3.16155 | Open Source |
| *Mustela nivalis* | Native | 52.4822 | -1.109099999 | Open Source |
| *Mustela nivalis* | Native | 55.0144 | -1.5273 | Open Source |
| *Mustela nivalis* | Native | 55.90495 | -4.24017 | Open Source |
| *Mustela nivalis* | Native | 52.02069 | 1.3005 | Open Source |
| *Mustela nivalis* | Native | 51.36305 | -0.23107 | Open Source |
| *Mustela nivalis* | Native | 55.01779 | -1.53001 | Open Source |
| *Mustela nivalis* | Native | 53.80743 | -2.6218 | Open Source |
| *Mustela nivalis* | Native | 55.94228 | -3.1647 | Open Source |
| *Mustela nivalis* | Native | 52.25697 | -3.5009 | Open Source |
| *Mustela nivalis* | Native | 50.86315 | 0.02002 | Open Source |
| *Mustela nivalis* | Native | 52.71326 | -2.662370001 | Open Source |
| *Mustela nivalis* | Native | 50.9586 | 0.739699999 | Open Source |
| *Mustela nivalis* | Native | 51.1256 | -1.294900001 | Open Source |
| *Mustela nivalis* | Native | 53.39022 | -1.13687 | Open Source |
| *Mustela nivalis* | Native | 50.9125 | -3.883989999 | Open Source |
| *Mustela nivalis* | Native | 52.74309 | -0.98009 | Open Source |
| *Mustela nivalis* | Native | 55.51592 | -1.6207 | Open Source |
| *Mustela nivalis* | Native | 57.1802 | -2.20757 | Open Source |
| *Mustela nivalis* | Native | 50.77739 | -0.80452 | Open Source |
| *Mustela nivalis* | Native | 51.26719 | -2.052910001 | Open Source |
| *Mustela nivalis* | Native | 53.63204 | 0.12257 | Open Source |
| *Mustela nivalis* | Native | 50.96097 | -3.774879999 | Open Source |
| *Mustela nivalis* | Native | 51.0558 | -3.973989999 | Open Source |
| *Mustela nivalis* | Native | 56.4343 | -3.706199999 | Open Source |
| *Mustela nivalis* | Native | 54.90253 | -1.58827 | Open Source |
| *Mustela nivalis* | Native | 53.6682 | -2.9908 | Open Source |
| *Mustela nivalis* | Native | 50.59317 | -2.0388 | Open Source |
| *Mustela nivalis* | Native | 51.5884 | -1.927299999 | Open Source |
| *Mustela nivalis* | Native | 50.7971 | -0.69759 | Open Source |
| *Mustela nivalis* | Native | 54.36455 | -1.55596 | Open Source |
| *Mustela nivalis* | Native | 52.5915 | -1.7004 | Open Source |
| *Mustela nivalis* | Native | 52.38943 | 2.42E-02 | Open Source |
| *Mustela nivalis* | Native | 55.9486 | -3.16169 | Open Source |
| *Mustela nivalis* | Native | 51.49334 | 0.22416 | Open Source |
| *Mustela nivalis* | Native | 52.41314 | -4.081849999 | Open Source |
| *Mustela nivalis* | Native | 52.81764 | -2.305619999 | Open Source |
| *Mustela nivalis* | Native | 51.3239 | 0.449600001 | Open Source |
| *Mustela nivalis* | Native | 52.14923 | 0.54225 | Open Source |
| *Mustela nivalis* | Native | 55.95136 | -3.155369999 | Open Source |
| *Mustela nivalis* | Native | 51.0272 | -0.514899999 | Open Source |
| *Mustela nivalis* | Native | 51.4923 | 0.2308 | Open Source |
| *Mustela nivalis* | Native | 54.64017 | -2.76847 | Open Source |
| *Mustela nivalis* | Native | 52.6212 | 1.2291 | Open Source |
| *Mustela nivalis* | Native | 51.80065 | -1.182769999 | Open Source |
| *Mustela nivalis* | Native | 54.886 | -2.074699999 | Open Source |
| *Mustela nivalis* | Native | 54.4576 | -3.025100001 | Open Source |
| *Mustela nivalis* | Native | 51.26096 | -0.32099 | Open Source |
| *Mustela nivalis* | Native | 51.0237 | -0.1092 | Open Source |
| *Mustela nivalis* | Native | 50.71106 | -1.36615 | Open Source |
| *Mustela nivalis* | Native | 52.60921 | -2.61945 | Open Source |
| *Mustela nivalis* | Native | 55.94227 | -3.1663 | Open Source |
| *Mustela nivalis* | Native | 53.06675 | -2.420049999 | Open Source |
| *Mustela nivalis* | Native | 52.90226 | -1.22465 | Open Source |
| *Mustela nivalis* | Native | 54.94795 | -1.77516 | Open Source |
| *Mustela nivalis* | Native | 52.86151 | -3.67469 | Open Source |
| *Mustela nivalis* | Native | 55.94403 | -3.169560001 | Open Source |
| *Mustela nivalis* | Native | 53.37765 | -2.64709 | Open Source |
| *Mustela nivalis* | Native | 54.18328 | -2.92538 | Open Source |
| *Mustela nivalis* | Native | 52.267 | -2.130699999 | Open Source |
| *Mustela nivalis* | Native | 53.79364 | -1.8307 | Open Source |
| *Mustela nivalis* | Native | 55.68616 | -1.798779999 | Open Source |
| *Mustela nivalis* | Native | 53.3473 | -0.9636 | Open Source |
| *Mustela nivalis* | Native | 53.0495 | -2.0985 | Open Source |
| *Mustela nivalis* | Native | 51.74682 | -1.07655 | Open Source |
| *Mustela nivalis* | Native | 51.39784 | -1.81524 | Open Source |
| *Mustela nivalis* | Native | 51.89018 | 0.41592 | Open Source |
| *Mustela nivalis* | Native | 55.94668 | -3.174439999 | Open Source |
| *Mustela nivalis* | Native | 52.77856 | -2.91317 | Open Source |
| *Mustela nivalis* | Native | 55.94504 | -3.158380001 | Open Source |
| *Mustela nivalis* | Native | 54.59941 | -1.64474 | Open Source |
| *Mustela nivalis* | Native | 51.53032 | -0.79982 | Open Source |
| *Mustela nivalis* | Native | 54.92988 | -1.72844 | Open Source |
| *Mustela nivalis* | Native | 52.7189 | -3.329399999 | Open Source |
| *Mustela nivalis* | Native | 51.00466 | -0.384420001 | Open Source |
| *Mustela nivalis* | Native | 51.69119 | -3.20722 | Open Source |
| *Mustela nivalis* | Native | 56.02488 | -2.841479999 | Open Source |
| *Mustela nivalis* | Native | 54.14832 | -1.66698 | Open Source |
| *Mustela nivalis* | Native | 55.14775 | -4.24855 | Open Source |
| *Mustela nivalis* | Native | 55.98972 | -5.007569999 | Open Source |
| *Mustela nivalis* | Native | 55.95311 | -3.16022 | Open Source |
| *Mustela nivalis* | Native | 51.8637 | -2.4222 | Open Source |
| *Mustela nivalis* | Native | 56.44484 | -3.47527 | Open Source |
| *Mustela nivalis* | Native | 55.86428 | -4.252219999 | Open Source |
| *Mustela nivalis* | Native | 51.91895 | 0.37239 | Open Source |
| *Mustela nivalis* | Native | 55.05323 | -1.647000001 | Open Source |
| *Mustela nivalis* | Native | 55.129134 | -3.441824 | Open Source |
| *Mustela nivalis* | Native | 53.66245 | -2.357100001 | Open Source |
| *Mustela nivalis* | Native | 55.4921 | -1.5991 | Open Source |
| *Mustela nivalis* | Native | 50.91134 | -0.50074 | Open Source |
| *Mustela nivalis* | Native | 56.54632 | -4.30559 | Open Source |
| *Mustela nivalis* | Native | 55.95218 | -3.1634 | Open Source |
| *Mustela nivalis* | Native | 54.93659 | -1.71513 | Open Source |
| *Mustela nivalis* | Native | 58.55736 | -3.694559999 | Open Source |
| *Mustela nivalis* | Native | 51.6253 | -1.18015 | Open Source |
| *Mustela nivalis* | Native | 54.6424 | -3.3346 | Open Source |
| *Mustela nivalis* | Native | 55.9477 | -3.161659999 | Open Source |
| *Mustela nivalis* | Native | 55.2862 | -1.574000001 | Open Source |
| *Mustela nivalis* | Native | 55.94403 | -3.169560001 | Open Source |
| *Mustela nivalis* | Native | 56.25913 | -2.996739999 | Open Source |
| *Mustela nivalis* | Native | 53.7085 | -2.6598 | Open Source |
| *Mustela nivalis* | Native | 57.35046 | -6.4512 | Open Source |
| *Mustela nivalis* | Native | 52.13407 | -2.25928 | Open Source |
| *Mustela nivalis* | Native | 52.58369 | -2.92749 | Open Source |
| *Mustela nivalis* | Native | 52.5426 | -1.807200001 | Open Source |
| *Mustela nivalis* | Native | 53.88454 | -2.029630001 | Open Source |
| *Mustela nivalis* | Native | 55.94668 | -3.174439999 | Open Source |
| *Mustela nivalis* | Native | 53.86651 | -1.88515 | Open Source |
| *Mustela nivalis* | Native | 55.80596 | -4.611110001 | Open Source |
| *Mustela nivalis* | Native | 52.76975 | -0.28947 | Open Source |
| *Mustela nivalis* | Native | 52.34193 | -2.154809999 | Open Source |
| *Mustela nivalis* | Native | 54.90253 | -1.58827 | Open Source |
| *Mustela nivalis* | Native | 53.26714 | -1.70909 | Open Source |
| *Mustela nivalis* | Native | 52.8086 | 0.575500001 | Open Source |
| *Mustela nivalis* | Native | 51.05014 | -3.36311 | Open Source |
| *Mustela nivalis* | Native | 51.34327 | -2.623759999 | Open Source |
| *Mustela nivalis* | Native | 54.60902 | -3.130950001 | Open Source |
| *Mustela nivalis* | Native | 53.1539 | -1.992 | Open Source |
| *Mustela nivalis* | Native | 51.59467 | -2.827869999 | Open Source |
| *Mustela nivalis* | Native | 55.80599 | -4.609520001 | Open Source |
| *Mustela nivalis* | Native | 56.55872 | -5.09899 | Open Source |
| *Mustela nivalis* | Native | 51.04628 | -1.330210001 | Open Source |
| *Mustela nivalis* | Native | 54.8998 | -1.4854 | Open Source |
| *Mustela nivalis* | Native | 55.49433 | -1.612989999 | Open Source |
| *Mustela nivalis* | Native | 52.25646 | 1.82E-02 | Open Source |
| *Mustela nivalis* | Native | 56.13841 | -3.40075 | Open Source |
| *Mustela nivalis* | Native | 50.95211 | 0.72506 | Open Source |
| *Mustela nivalis* | Native | 50.91721 | -0.14837 | Open Source |
| *Mustela nivalis* | Native | 51.4962 | 0.2359 | Open Source |
| *Mustela nivalis* | Native | 51.91697 | 0.38101 | Open Source |
| *Mustela nivalis* | Native | 53.0753 | -1.4832 | Open Source |
| *Mustela nivalis* | Native | 54.21401 | -0.42741 | Open Source |
| *Mustela nivalis* | Native | 54.49307 | -2.53151 | Open Source |
| *Mustela nivalis* | Native | 54.9983 | -1.5397 | Open Source |
| *Mustela nivalis* | Native | 53.38793 | -1.66993 | Open Source |
| *Mustela nivalis* | Native | 50.93062 | 2.96E-02 | Open Source |
| *Mustela nivalis* | Native | 50.8476 | -4.509599999 | Open Source |
| *Mustela nivalis* | Native | 51.2463 | -1.144584 | Open Source |
| *Mustela nivalis* | Native | 52.4743 | -2.3767 | Open Source |
| *Mustela nivalis* | Native | 52.30409 | -2.50372 | Open Source |
| *Mustela nivalis* | Native | 53.97004 | -0.87415 | Open Source |
| *Mustela nivalis* | Native | 50.97005 | -3.76953 | Open Source |
| *Mustela nivalis* | Native | 53.54838 | -2.014299999 | Open Source |
| *Mustela nivalis* | Native | 50.96425 | -3.155529999 | Open Source |
| *Mustela nivalis* | Native | 53.45586 | -2.44949 | Open Source |
| *Mustela nivalis* | Native | 53.57444 | -2.03847 | Open Source |
| *Mustela nivalis* | Native | 54.21485 | -0.42277 | Open Source |
| *Mustela nivalis* | Native | 52.8261 | 1.4634 | Open Source |
| *Mustela nivalis* | Native | 50.96275 | 0.15101 | Open Source |
| *Mustela nivalis* | Native | 55.95128 | -3.16337 | Open Source |
| *Mustela nivalis* | Native | 52.58295 | -1.199300001 | Open Source |
| *Mustela nivalis* | Native | 55.94137 | -3.16628 | Open Source |
| *Mustela nivalis* | Native | 56.16935 | -5.44234 | Open Source |
| *Mustela nivalis* | Native | 55.94146 | -3.15667 | Open Source |
| *Mustela nivalis* | Native | 53.37681 | -1.56329 | Open Source |
| *Mustela nivalis* | Native | 54.21485 | -0.42277 | Open Source |
| *Mustela nivalis* | Native | 51.99058 | -1.702869999 | Open Source |
| *Mustela nivalis* | Native | 51.8644 | 0.2464 | Open Source |
| *Mustela nivalis* | Native | 52.4489 | -2.1065 | Open Source |
| *Mustela nivalis* | Native | 51.7576 | -2.087600001 | Open Source |
| *Mustela nivalis* | Native | 51.1875 | -4.89E-02 | Open Source |
| *Mustela nivalis* | Native | 50.16103 | -5.670450001 | Open Source |
| *Mustela nivalis* | Native | 51.7744 | -2.3797 | Open Source |
| *Mustela nivalis* | Native | 52.35272 | -2.151910001 | Open Source |
| *Mustela nivalis* | Native | 53.96925 | -1.0998 | Open Source |
| *Mustela nivalis* | Native | 52.28093 | -0.40584 | Open Source |
| *Mustela nivalis* | Native | 50.96544 | 0.59408 | Open Source |
| *Mustela nivalis* | Native | 54.69864 | -1.498019999 | Open Source |
| *Mustela nivalis* | Native | 54.5669 | -1.2841 | Open Source |
| *Mustela nivalis* | Native | 54.30664 | -1.458230001 | Open Source |
| *Mustela nivalis* | Native | 51.17855 | -1.909110001 | Open Source |
| *Mustela nivalis* | Native | 50.94508 | -0.17659 | Open Source |
| *Mustela nivalis* | Native | 52.01568 | 1.08731 | Open Source |
| *Mustela nivalis* | Native | 55.97655 | -3.15131 | Open Source |
| *Mustela nivalis* | Native | 55.94406 | -3.16636 | Open Source |
| *Mustela nivalis* | Native | 52.5136 | -1.249199999 | Open Source |
| *Mustela nivalis* | Native | 53.3167 | -1.4615 | Open Source |
| *Mustela nivalis* | Native | 51.73627 | -1.10718 | Open Source |
| *Mustela nivalis* | Native | 54.96586 | -1.74382 | Open Source |
| *Mustela nivalis* | Native | 50.59137 | -2.03738 | Open Source |
| *Mustela nivalis* | Native | 52.91093 | 0.70967 | Open Source |
| *Mustela nivalis* | Native | 53.3078 | -2.731899999 | Open Source |
| *Mustela nivalis* | Native | 58.53146 | -3.50181 | Open Source |
| *Mustela nivalis* | Native | 52.5472 | -1.702600001 | Open Source |
| *Mustela nivalis* | Native | 56.45304 | -3.21598 | Open Source |
| *Mustela nivalis* | Native | 55.94323 | -3.159929999 | Open Source |
| *Mustela nivalis* | Native | 55.95646 | -2.98094 | Open Source |
| *Mustela nivalis* | Native | 54.18995 | -2.19842 | Open Source |
| *Mustela nivalis* | Native | 50.83668 | -1.80731 | Open Source |
| *Mustela nivalis* | Native | 53.2894 | -1.568999999 | Open Source |
| *Mustela nivalis* | Native | 53.26272 | -1.582439999 | Open Source |
| *Mustela nivalis* | Native | 50.86162 | -0.38084 | Open Source |
| *Mustela nivalis* | Native | 53.61187 | 0.14424 | Open Source |
| *Mustela nivalis* | Native | 51.71432 | -3.231 | Open Source |
| *Mustela nivalis* | Native | 55.95487 | -3.16348 | Open Source |
| *Mustela nivalis* | Native | 52.11081 | 1.14574 | Open Source |
| *Mustela nivalis* | Native | 54.90253 | -1.58827 | Open Source |
| *Mustela nivalis* | Native | 52.05699 | -0.54072 | Open Source |
| *Mustela nivalis* | Native | 52.88452 | -1.85796 | Open Source |
| *Mustela nivalis* | Native | 51.43843 | 0.44575 | Open Source |
| *Mustela nivalis* | Native | 50.97951 | -2.28555 | Open Source |
| *Mustela nivalis* | Native | 56.46064 | -5.38642 | Open Source |
| *Mustela nivalis* | Native | 51.563 | -2.58E-02 | Open Source |
| *Mustela nivalis* | Native | 53.60623 | -0.48635 | Open Source |
| *Mustela nivalis* | Native | 52.9618 | -1.64936 | Open Source |
| *Mustela nivalis* | Native | 51.70027 | -1.103549999 | Open Source |
| *Mustela nivalis* | Native | 54.6429 | -1.181200001 | Open Source |
| *Mustela nivalis* | Native | 55.94406 | -3.16636 | Open Source |
| *Mustela nivalis* | Native | 55.94053 | -3.15985 | Open Source |
| *Mustela nivalis* | Native | 57.0586 | -2.487999999 | Open Source |
| *Mustela nivalis* | Native | 52.5641 | -0.7519 | Open Source |
| *Mustela nivalis* | Native | 50.8494 | -0.2955 | Open Source |
| *Mustela nivalis* | Native | 51.829 | -1.63645 | Open Source |
| *Mustela nivalis* | Native | 55.94315 | -3.167929999 | Open Source |
| *Mustela nivalis* | Native | 51.2176 | 0.321819999 | Open Source |
| *Mustela nivalis* | Native | 53.8165 | -3.0094 | Open Source |
| *Mustela nivalis* | Native | 53.3927 | -0.4996 | Open Source |
| *Mustela nivalis* | Native | 56.57726 | -3.54894 | Open Source |
| *Mustela nivalis* | Native | 55.79039 | -4.20209 | Open Source |
| *Mustela nivalis* | Native | 55.79063 | -4.20011 | Open Source |
| *Mustela nivalis* | Native | 56.57726 | -3.54894 | Open Source |
| *Mustela nivalis* | Native | 56.57726 | -3.54894 | Open Source |
| *Mustela nivalis* | Native | 56.57726 | -3.54894 | Open Source |
| *Mustela nivalis* | Native | 55.79039 | -4.20209 | Open Source |
| *Mustela nivalis* | Native | 56.01113 | -3.73414 | Open Source |
| *Mustela nivalis* | Native | 55.79063 | -4.20011 | Open Source |
| *Mustela nivalis* | Native | 55.588 | -4.63153 | Open Source |
| *Mustela nivalis* | Native | 56.57726 | -3.54894 | Open Source |
| *Mustela nivalis* | Native | 56.57726 | -3.54894 | Open Source |
| *Mustela nivalis* | Native | 56.57726 | -3.54894 | Open Source |
| *Mustela nivalis* | Native | 55.79063 | -4.20011 | Open Source |
| *Mustela nivalis* | Native | 55.79063 | -4.20011 | Open Source |
| *Mustela nivalis* | Native | 55.79063 | -4.20011 | Open Source |
| *Mustela nivalis* | Native | 51.31606 | -0.46965 | Open Source |
| *Mustela nivalis* | Native | 51.3143 | -0.47258 | Open Source |
| *Mustela nivalis* | Native | 51.37447 | -0.183929999 | Open Source |
| *Mustela nivalis* | Native | 52.46742 | -2.067670001 | Open Source |
| *Mustela nivalis* | Native | 52.22473 | 1.256309999 | Open Source |
| *Mustela nivalis* | Native | 53.56391 | -2.22039 | Open Source |
| *Mustela nivalis* | Native | 51.24446 | -2.223439999 | Open Source |
| *Mustela nivalis* | Native | 51.03749 | -2.29375 | Open Source |
| *Mustela nivalis* | Native | 51.26171 | 0.556979999 | Open Source |
| *Mustela nivalis* | Native | 52.98882 | -2.05359 | Open Source |
| *Mustela nivalis* | Native | 51.10227 | -3.96526 | Open Source |
| *Mustela nivalis* | Native | 52.39524 | -1.74425 | Open Source |
| *Mustela nivalis* | Native | 51.06846 | -0.16739 | Open Source |
| *Mustela nivalis* | Native | 53.16633 | -1.26103 | Open Source |
| *Mustela nivalis* | Native | 57.48554 | -3.144180001 | Open Source |
| *Mustela nivalis* | Native | 51.02189 | 0.41542 | Open Source |
| *Mustela nivalis* | Native | 51.02577 | -2.84977 | Open Source |
| *Mustela nivalis* | Native | 50.15275 | -5.66211 | Open Source |
| *Mustela nivalis* | Native | 51.77749 | -0.74762 | Open Source |
| *Mustela nivalis* | Native | 53.86507 | -0.94465 | Open Source |
| *Mustela nivalis* | Native | 51.18117 | -1.63656 | Open Source |
| *Mustela nivalis* | Native | 53.13241 | -1.75484 | Open Source |
| *Mustela nivalis* | Native | 50.71251 | -1.38525 | Open Source |
| *Mustela nivalis* | Native | 52.32397 | 1.55718 | Open Source |
| *Mustela nivalis* | Native | 53.29266 | -2.65405 | Open Source |
| *Mustela nivalis* | Native | 53.18521 | -1.425319999 | Open Source |
| *Mustela nivalis* | Native | 54.03047 | -1.50533 | Open Source |
| *Mustela nivalis* | Native | 50.48555 | -4.24969 | Open Source |
| *Mustela nivalis* | Native | 57.25035 | -4.49612 | Open Source |
| *Mustela nivalis* | Native | 51.84562 | 0.139849999 | Open Source |
| *Mustela nivalis* | Native | 51.24843 | -0.174570001 | Open Source |
| *Mustela nivalis* | Native | 52.88496 | 0.75554 | Open Source |
| *Mustela nivalis* | Native | 53.22672 | -4.330869999 | Open Source |
| *Mustela nivalis* | Native | 51.02577 | -2.84977 | Open Source |
| *Mustela nivalis* | Native | 52.0713 | -4.547449999 | Open Source |
| *Mustela nivalis* | Native | 53.86507 | -0.94465 | Open Source |
| *Mustela nivalis* | Native | 55.75086 | -4.8457 | Open Source |
| *Mustela nivalis* | Native | 52.70569 | -3.030049999 | Open Source |
| *Mustela nivalis* | Native | 50.95824 | -3.97341 | Open Source |
| *Mustela nivalis* | Native | 53.16633 | -1.26103 | Open Source |
| *Mustela nivalis* | Native | 52.26008 | -3.50321 | Open Source |
| *Mustela nivalis* | Native | 51.38589 | 1.34012 | Open Source |
| *Mustela nivalis* | Native | 53.5993 | -1.57082 | Open Source |
| *Mustela nivalis* | Native | 51.54624 | 0.70272 | Open Source |
| *Mustela nivalis* | Native | 52.04188 | -0.317399999 | Open Source |
| *Mustela nivalis* | Native | 51.0132 | -3.24869 | Open Source |
| *Mustela nivalis* | Native | 51.81622 | 0.26904 | Open Source |
| *Mustela nivalis* | Native | 52.0538 | -2.13996 | Open Source |
| *Mustela nivalis* | Native | 52.0713 | -4.547449999 | Open Source |
| *Mustela nivalis* | Native | 52.70569 | -3.030049999 | Open Source |
| *Mustela nivalis* | Native | 53.13241 | -1.75484 | Open Source |
| *Mustela nivalis* | Native | 53.64488 | -2.17543 | Open Source |
| *Mustela nivalis* | Native | 51.03749 | -2.29375 | Open Source |
| *Mustela nivalis* | Native | 53.86507 | -0.94465 | Open Source |
| *Mustela nivalis* | Native | 52.0756 | 0.34055 | Open Source |
| *Mustela nivalis* | Native | 51.18975 | -1.522020001 | Open Source |
| *Mustela nivalis* | Native | 51.26171 | 0.556979999 | Open Source |
| *Mustela nivalis* | Native | 51.75872 | -0.67569 | Open Source |
| *Mustela nivalis* | Native | 52.28017 | -3.32798 | Open Source |
| *Mustela nivalis* | Native | 53.86507 | -0.94465 | Open Source |
| *Mustela nivalis* | Native | 50.84442 | -4.29533 | Open Source |
| *Mustela nivalis* | Native | 52.88496 | 0.75554 | Open Source |
| *Mustela nivalis* | Native | 52.28017 | -3.32798 | Open Source |
| *Mustela nivalis* | Native | 51.8849 | 0.43235 | Open Source |
| *Mustela nivalis* | Native | 52.32397 | 1.55718 | Open Source |
| *Mustela nivalis* | Native | 53.22672 | -4.330869999 | Open Source |
| *Mustela nivalis* | Native | 51.0132 | -3.24869 | Open Source |
| *Mustela nivalis* | Native | 51.97904 | 0.219020001 | Open Source |
| *Mustela nivalis* | Native | 51.06846 | -0.16739 | Open Source |
| *Mustela nivalis* | Native | 50.71251 | -1.38525 | Open Source |
| *Mustela nivalis* | Native | 53.64488 | -2.17543 | Open Source |
| *Mustela nivalis* | Native | 52.62769 | -1.307810001 | Open Source |
| *Mustela nivalis* | Native | 52.59401 | -0.99832 | Open Source |
| *Mustela nivalis* | Native | 52.58421 | -1.00888 | Open Source |
| *Mustela nivalis* | Native | 52.55324 | -0.77359 | Open Source |
| *Mustela nivalis* | Native | 52.64901 | -1.43015 | Open Source |
| *Mustela nivalis* | Native | 52.70476 | -1.43239 | Open Source |
| *Mustela nivalis* | Native | 52.55324 | -0.77359 | Open Source |
| *Mustela nivalis* | Native | 52.55165 | -1.09663 | Open Source |
| *Mustela nivalis* | Native | 52.63111 | -1.27968 | Open Source |
| *Mustela nivalis* | Native | 52.63122 | -1.29741 | Open Source |
| *Mustela nivalis* | Native | 52.74078 | -1.44525 | Open Source |
| *Mustela nivalis* | Native | 52.79173 | -1.23106 | Open Source |
| *Mustela nivalis* | Native | 52.52653 | -1.231279999 | Open Source |
| *Mustela nivalis* | Native | 52.88752 | -0.84889 | Open Source |
| *Mustela nivalis* | Native | 52.57295 | -1.18325 | Open Source |
| *Mustela nivalis* | Native | 52.79809 | -1.241329999 | Open Source |
| *Mustela nivalis* | Native | 57.54759 | -2.97487 | Open Source |
| *Mustela nivalis* | Native | 57.04506 | -3.90532 | Open Source |
| *Mustela nivalis* | Native | 57.10435 | -2.90377 | Open Source |
| *Mustela nivalis* | Native | 57.14084 | -2.056990001 | Open Source |
| *Mustela nivalis* | Native | 57.42897 | -2.238959999 | Open Source |
| *Mustela nivalis* | Native | 57.33129 | -2.00081 | Open Source |
| *Mustela nivalis* | Native | 57.35733 | -1.97256 | Open Source |
| *Mustela nivalis* | Native | 57.09342 | -2.71949 | Open Source |
| *Mustela nivalis* | Native | 56.99184 | -3.49027 | Open Source |
| *Mustela nivalis* | Native | 57.33668 | -1.997489999 | Open Source |
| *Mustela nivalis* | Native | 57.31431 | -3.335600001 | Open Source |
| *Mustela nivalis* | Native | 56.37798 | -3.838680001 | Open Source |
| *Mustela nivalis* | Native | 57.42888 | -3.37475 | Open Source |
| *Mustela nivalis* | Native | 57.2694 | -3.41689 | Open Source |
| *Mustela nivalis* | Native | 57.09764 | -3.990559999 | Open Source |
| *Mustela nivalis* | Native | 57.40576 | -2.142280001 | Open Source |
| *Mustela nivalis* | Native | 57.40958 | -2.276279999 | Open Source |
| *Mustela nivalis* | Native | 57.06679 | -3.000150001 | Open Source |
| *Mustela nivalis* | Native | 57.44623 | -2.13702 | Open Source |
| *Mustela nivalis* | Native | 57.70381 | -2.86328 | Open Source |
| *Mustela nivalis* | Native | 57.40716 | -2.348659999 | Open Source |
| *Mustela nivalis* | Native | 57.47924 | -3.735229999 | Open Source |
| *Mustela nivalis* | Native | 57.01347 | -3.558659999 | Open Source |
| *Mustela nivalis* | Native | 57.32666 | -2.77387 | Open Source |
| *Mustela nivalis* | Native | 57.11358 | -2.15279 | Open Source |
| *Mustela nivalis* | Native | 57.17163 | -2.40597 | Open Source |
| *Mustela nivalis* | Native | 57.4283 | -1.9775 | Open Source |
| *Mustela nivalis* | Native | 57.513 | -2.78365 | Open Source |
| *Mustela nivalis* | Native | 56.97617 | -2.26895 | Open Source |
| *Mustela nivalis* | Native | 57.44247 | -2.225719999 | Open Source |
| *Mustela nivalis* | Native | 57.36721 | -2.03407 | Open Source |
| *Mustela nivalis* | Native | 57.10609 | -2.912070001 | Open Source |
| *Mustela nivalis* | Native | 57.06793 | -3.079350001 | Open Source |
| *Mustela nivalis* | Native | 57.24861 | -2.088640001 | Open Source |
| *Mustela nivalis* | Native | 56.99771 | -2.27569 | Open Source |
| *Mustela nivalis* | Native | 57.16306 | -3.189589999 | Open Source |
| *Mustela nivalis* | Native | 57.35104 | -1.942649999 | Open Source |
| *Mustela nivalis* | Native | 56.97617 | -2.26895 | Open Source |
| *Mustela nivalis* | Native | 56.97617 | -2.26895 | Open Source |
| *Mustela nivalis* | Native | 57.15971 | -2.800580001 | Open Source |
| *Mustela nivalis* | Native | 57.26088 | -3.10156 | Open Source |
| *Mustela nivalis* | Native | 57.24928 | -2.720040001 | Open Source |
| *Mustela nivalis* | Native | 57.04134 | -4.135900001 | Open Source |
| *Mustela nivalis* | Native | 56.82475 | -4.189099999 | Open Source |
| *Mustela nivalis* | Native | 57.4856 | -2.21764 | Open Source |
| *Mustela nivalis* | Native | 57.14318 | -2.30156 | Open Source |
| *Mustela nivalis* | Native | 57.4462 | -2.13699 | Open Source |
| *Mustela nivalis* | Native | 57.25571 | -2.173189999 | Open Source |
| *Mustela nivalis* | Native | 56.97617 | -2.26895 | Open Source |
| *Mustela nivalis* | Native | 57.40199 | -2.25376 | Open Source |
| *Mustela nivalis* | Native | 57.16226 | -2.802190001 | Open Source |
| *Mustela nivalis* | Native | 57.15809 | -2.79885 | Open Source |
| *Mustela nivalis* | Native | 57.14173 | -2.0636 | Open Source |
| *Mustela nivalis* | Native | 57.06008 | -3.051089999 | Open Source |
| *Mustela nivalis* | Native | 57.27305 | -3.66829 | Open Source |
| *Mustela nivalis* | Native | 57.33776 | -2.25913 | Open Source |
| *Mustela nivalis* | Native | 57.30613 | -1.99749 | Open Source |
| *Mustela nivalis* | Native | 57.15674 | -2.721550001 | Open Source |
| *Mustela nivalis* | Native | 57.28982 | -2.511790001 | Open Source |
| *Mustela nivalis* | Native | 55.64762 | -4.659370001 | Open Source |
| *Mustela nivalis* | Native | 55.64196 | -4.6717 | Open Source |
| *Mustela nivalis* | Native | 55.63995 | -4.63978 | Open Source |
| *Mustela nivalis* | Native | 51.44956 | -2.67054 | Open Source |
| *Mustela nivalis* | Native | 51.38736 | -2.52592 | Open Source |
| *Mustela nivalis* | Native | 51.44081 | -2.62725 | Open Source |
| *Mustela nivalis* | Native | 51.44081 | -2.62725 | Open Source |
| *Mustela nivalis* | Native | 51.37673 | -2.813149999 | Open Source |
| *Mustela nivalis* | Native | 51.37772 | -2.65512 | Open Source |
| *Mustela nivalis* | Native | 51.58503 | -2.55707 | Open Source |
| *Mustela nivalis* | Native | 51.41343 | -2.698769999 | Open Source |
| *Mustela nivalis* | Native | 51.32393 | -2.62565 | Open Source |
| *Mustela nivalis* | Native | 51.38562 | -2.827680001 | Open Source |
| *Mustela nivalis* | Native | 51.36045 | -2.51125 | Open Source |
| *Mustela nivalis* | Native | 51.45856 | -2.67067 | Open Source |
| *Mustela nivalis* | Native | 51.36888 | -2.62627 | Open Source |
| *Mustela nivalis* | Native | 51.36774 | -2.81299 | Open Source |
| *Mustela nivalis* | Native | 51.45764 | -2.81459 | Open Source |
| *Mustela nivalis* | Native | 51.42188 | -2.78519 | Open Source |
| *Mustela nivalis* | Native | 51.4939 | -2.77202 | Open Source |
| *Mustela nivalis* | Native | 51.53115 | -2.541990001 | Open Source |
| *Mustela nivalis* | Native | 51.30348 | -2.984 | Open Source |
| *Mustela nivalis* | Native | 51.44875 | -2.80004 | Open Source |
| *Mustela nivalis* | Native | 51.59476 | -2.369520001 | Open Source |
| *Mustela nivalis* | Native | 51.41458 | -2.46872 | Open Source |
| *Mustela nivalis* | Native | 51.47637 | -2.69973 | Open Source |
| *Mustela nivalis* | Native | 51.38562 | -2.827680001 | Open Source |
| *Mustela nivalis* | Native | 51.47628 | -2.71413 | Open Source |
| *Mustela nivalis* | Native | 51.34199 | -2.611540001 | Open Source |
| *Mustela nivalis* | Native | 51.45856 | -2.67067 | Open Source |
| *Mustela nivalis* | Native | 51.44875 | -2.80004 | Open Source |
| *Mustela nivalis* | Native | 51.38736 | -2.52592 | Open Source |
| *Mustela nivalis* | Native | 51.42234 | -2.713289999 | Open Source |
| *Mustela nivalis* | Native | 51.30595 | -2.62541 | Open Source |
| *Mustela nivalis* | Native | 52.15217 | -3.62E-02 | Open Source |
| *Mustela nivalis* | Native | 52.14489 | -3.07E-02 | Open Source |
| *Mustela nivalis* | Native | 52.71833 | 1.61809 | Open Source |
| *Mustela nivalis* | Native | 52.91311 | 1.2594 | Open Source |
| *Mustela nivalis* | Native | 52.96158 | 1.02476 | Open Source |
| *Mustela nivalis* | Native | 52.08626 | 1.55063 | Open Source |
| *Mustela nivalis* | Native | 52.90846 | 1.265 | Open Source |
| *Mustela nivalis* | Native | 51.4385 | -0.55189 | Open Source |
| *Mustela nivalis* | Native | 54.79928 | -1.31167 | Open Source |
| *Mustela nivalis* | Native | 52.15217 | -3.62E-02 | Open Source |
| *Mustela nivalis* | Native | 52.90846 | 1.265 | Open Source |
| *Mustela nivalis* | Native | 52.71833 | 1.61809 | Open Source |
| *Mustela nivalis* | Native | 50.58219 | -4.90202 | Open Source |
| *Mustela nivalis* | Native | 50.58312 | -4.90067 | Open Source |
| *Mustela nivalis* | Native | 51.85772 | 0.23846 | Open Source |
| *Mustela nivalis* | Native | 52.90846 | 1.265 | Open Source |
| *Mustela nivalis* | Native | 54.92086 | -1.7129 | Open Source |
| *Mustela nivalis* | Native | 52.089 | 1.549389999 | Open Source |
| *Mustela nivalis* | Native | 50.58219 | -4.90202 | Open Source |
| *Mustela nivalis* | Native | 54.92089 | -1.7285 | Open Source |
| *Mustela nivalis* | Native | 53.34957 | -1.80092 | Open Source |
| *Mustela nivalis* | Native | 55.51592 | -1.6207 | Open Source |
| *Mustela nivalis* | Native | 52.14489 | -3.07E-02 | Open Source |
| *Mustela nivalis* | Native | 52.95791 | 0.731150001 | Open Source |
| *Mustela nivalis* | Native | 51.85778 | 0.23555 | Open Source |
| *Mustela nivalis* | Native | 51.85741 | -0.54283 | Open Source |
| *Mustela nivalis* | Native | 51.85778 | 0.23555 | Open Source |
| *Mustela nivalis* | Native | 51.85772 | 0.23846 | Open Source |
| *Mustela nivalis* | Native | 51.85778 | 0.23555 | Open Source |
| *Mustela nivalis* | Native | 52.30898 | 0.293429999 | Open Source |
| *Mustela nivalis* | Native | 52.31059 | 0.279579999 | Open Source |
| *Mustela nivalis* | Native | 52.30202 | 0.28134 | Open Source |
| *Mustela nivalis* | Native | 52.31173 | 0.29064 | Open Source |
| *Mustela nivalis* | Native | 56.2787 | -5.62816 | Open Source |
| *Mustela nivalis* | Native | 56.12997 | -5.485339999 | Open Source |
| *Mustela nivalis* | Native | 56.28489 | -5.09803 | Open Source |
| *Mustela nivalis* | Native | 58.16488 | -4.98755 | Open Source |
| *Mustela nivalis* | Native | 58.59459 | -3.073000001 | Open Source |
| *Mustela nivalis* | Native | 57.90022 | -4.32079 | Open Source |
| *Mustela nivalis* | Native | 56.84152 | -5.04033 | Open Source |
| *Mustela nivalis* | Native | 57.92681 | -4.02696 | Open Source |
| *Mustela nivalis* | Native | 58.11017 | -4.94392 | Open Source |
| *Mustela nivalis* | Native | 58.57578 | -3.55186 | Open Source |
| *Mustela nivalis* | Native | 58.14516 | -5.24256 | Open Source |
| *Mustela nivalis* | Native | 58.01182 | -4.2603 | Open Source |
| *Mustela nivalis* | Native | 57.92964 | -4.07441 | Open Source |
| *Mustela nivalis* | Native | 58.55956 | -3.480659999 | Open Source |
| *Mustela nivalis* | Native | 58.19285 | -5.327759999 | Open Source |
| *Mustela nivalis* | Native | 58.24155 | -5.19852 | Open Source |
| *Mustela nivalis* | Native | 57.62603 | -4.690190001 | Open Source |
| *Mustela nivalis* | Native | 57.62345 | -4.70591 | Open Source |
| *Mustela nivalis* | Native | 56.66101 | -3.57834 | Open Source |
| *Mustela nivalis* | Native | 58.14124 | -5.237949999 | Open Source |
| *Mustela nivalis* | Native | 58.63927 | -3.337599999 | Open Source |
| *Mustela nivalis* | Native | 57.62619 | -4.690439999 | Open Source |
| *Mustela nivalis* | Native | 57.62345 | -4.70591 | Open Source |
| *Mustela nivalis* | Native | 57.64911 | -4.08686 | Open Source |
| *Mustela nivalis* | Native | 57.47131 | -4.47865 | Open Source |
| *Mustela nivalis* | Native | 58.64988 | -3.353519999 | Open Source |
| *Mustela nivalis* | Native | 58.2497 | -5.213749999 | Open Source |
| *Mustela nivalis* | Native | 58.23676 | -5.17423 | Open Source |
| *Mustela nivalis* | Native | 58.4067 | -3.414350001 | Open Source |
| *Mustela nivalis* | Native | 58.45981 | -3.13196 | Open Source |
| *Mustela nivalis* | Native | 57.56403 | -4.5602 | Open Source |
| *Mustela nivalis* | Native | 57.48898 | -4.204530001 | Open Source |
| *Mustela nivalis* | Native | 57.24667 | -5.928200001 | Open Source |
| *Mustela nivalis* | Native | 58.59401 | -3.45287 | Open Source |
| *Mustela nivalis* | Native | 56.82475 | -4.189099999 | Open Source |
| *Mustela nivalis* | Native | 58.3881 | -3.5838 | Open Source |
| *Mustela nivalis* | Native | 58.4707 | -3.394630001 | Open Source |
| *Mustela nivalis* | Native | 57.75286 | -4.06091 | Open Source |
| *Mustela nivalis* | Native | 57.29384 | -3.64441 | Open Source |
| *Mustela nivalis* | Native | 57.47924 | -3.735229999 | Open Source |
| *Mustela nivalis* | Native | 58.17353 | -5.00189 | Open Source |
| *Mustela nivalis* | Native | 57.63 | -4.25829 | Open Source |
| *Mustela nivalis* | Native | 58.2377 | -5.172609999 | Open Source |
| *Mustela nivalis* | Native | 57.62345 | -4.70591 | Open Source |
| *Mustela nivalis* | Native | 58.40674 | -3.41092 | Open Source |
| *Mustela nivalis* | Native | 58.22916 | -5.314120001 | Open Source |
| *Mustela nivalis* | Native | 57.28218 | -4.4543 | Open Source |
| *Mustela nivalis* | Native | 58.62899 | -3.375099999 | Open Source |
| *Mustela nivalis* | Native | 58.23676 | -5.17423 | Open Source |
| *Mustela nivalis* | Native | 57.62603 | -4.690190001 | Open Source |
| *Mustela nivalis* | Native | 57.62619 | -4.690439999 | Open Source |
| *Mustela nivalis* | Native | 57.01935 | -4.28702 | Open Source |
| *Mustela nivalis* | Native | 57.47137 | -4.18345 | Open Source |
| *Mustela nivalis* | Native | 58.20035 | -5.00756 | Open Source |
| *Mustela nivalis* | Native | 57.29655 | -5.56184 | Open Source |
| *Mustela nivalis* | Native | 57.37568 | -4.022240001 | Open Source |
| *Mustela nivalis* | Native | 58.15063 | -5.221809999 | Open Source |
| *Mustela nivalis* | Native | 58.12093 | -4.96434 | Open Source |
| *Mustela nivalis* | Native | 57.55975 | -4.54987 | Open Source |
| *Mustela nivalis* | Native | 57.23888 | -5.85557 | Open Source |
| *Mustela nivalis* | Native | 58.31979 | -5.1236 | Open Source |
| *Mustela nivalis* | Native | 57.626 | -4.691870001 | Open Source |
| *Mustela nivalis* | Native | 58.23676 | -5.17423 | Open Source |
| *Mustela nivalis* | Native | 58.58062 | -3.362879999 | Open Source |
| *Mustela nivalis* | Native | 57.53732 | -4.514299999 | Open Source |
| *Mustela nivalis* | Native | 57.14772 | -5.33498 | Open Source |
| *Mustela nivalis* | Native | 57.5708 | -4.58075 | Open Source |
| *Mustela nivalis* | Native | 57.57436 | -4.582670001 | Open Source |
| *Mustela nivalis* | Native | 57.65648 | -4.385280001 | Open Source |
| *Mustela nivalis* | Native | 57.76354 | -4.772720001 | Open Source |
| *Mustela nivalis* | Native | 57.09995 | -3.986550001 | Open Source |
| *Mustela nivalis* | Native | 58.52355 | -3.37827 | Open Source |
| *Mustela nivalis* | Native | 57.04531 | -4.14025 | Open Source |
| *Mustela nivalis* | Native | 58.20137 | -5.345580001 | Open Source |
| *Mustela nivalis* | Native | 58.23676 | -5.17423 | Open Source |
| *Mustela nivalis* | Native | 58.47528 | -3.10845 | Open Source |
| *Mustela nivalis* | Native | 57.4809 | -4.17818 | Open Source |
| *Mustela nivalis* | Native | 58.062 | -5.32503 | Open Source |
| *Mustela nivalis* | Native | 57.28002 | -5.51044 | Open Source |
| *Mustela nivalis* | Native | 58.2462 | -5.34981 | Open Source |
| *Mustela nivalis* | Native | 58.59383 | -3.46835 | Open Source |
| *Mustela nivalis* | Native | 55.94904 | -3.1625 | Open Source |
| *Mustela nivalis* | Native | 57.90003 | -4.35538 | Open Source |
| *Mustela nivalis* | Native | 57.43276 | -4.12951 | Open Source |
| *Mustela nivalis* | Native | 57.57505 | -4.434689999 | Open Source |
| *Mustela nivalis* | Native | 57.25608 | -4.409460001 | Open Source |
| *Mustela nivalis* | Native | 57.58305 | -4.48541 | Open Source |
| *Mustela nivalis* | Native | 58.24334 | -5.23448 | Open Source |
| *Mustela nivalis* | Native | 57.40747 | -4.33948 | Open Source |
| *Mustela nivalis* | Native | 57.50545 | -4.31319 | Open Source |
| *Mustela nivalis* | Native | 57.86524 | -4.11117 | Open Source |
| *Mustela nivalis* | Native | 57.0429 | -3.89944 | Open Source |
| *Mustela nivalis* | Native | 58.55345 | -3.614459999 | Open Source |
| *Mustela nivalis* | Native | 58.55489 | -3.41688 | Open Source |
| *Mustela nivalis* | Native | 58.19763 | -5.334160001 | Open Source |
| *Mustela nivalis* | Native | 59.294354 | 15.262269 | Open Source |
| *Mustela nivalis* | Native | 58.954134 | 12.239618 | Open Source |
| *Mustela nivalis* | Native | 38.87 | -0.26 | Open Source |
| *Mustela nivalis* | Native | 40.05 | -1.35 | Open Source |
| *Mustela nivalis* | Native | 38.1 | -0.95 | Open Source |
| *Mustela nivalis* | Native | 39.17 | -0.99 | Open Source |
| *Mustela nivalis* | Native | 40.44 | -0.140000001 | Open Source |
| *Mustela nivalis* | Native | 38.85 | -3.00E-02 | Open Source |
| *Mustela nivalis* | Native | 39.9 | -0.33 | Open Source |
| *Mustela nivalis* | Native | 39.88 | -0.48 | Open Source |
| *Mustela nivalis* | Native | 39.89 | -0.46 | Open Source |
| *Mustela nivalis* | Native | 39.9 | -0.45 | Open Source |
| *Mustela nivalis* | Native | 38.8 | 9.00E-02 | Open Source |
| *Mustela nivalis* | Native | 40.45 | -0.15 | Open Source |
| *Mustela nivalis* | Native | 40.5 | -0.19 | Open Source |
| *Mustela nivalis* | Native | 38.52 | -0.66 | Open Source |
| *Mustela nivalis* | Native | 38.59 | -0.499999999 | Open Source |
| *Mustela nivalis* | Native | 38.58 | -0.5 | Open Source |
| *Mustela nivalis* | Native | 38.69 | -0.55 | Open Source |
| *Mustela nivalis* | Native | 39.57 | -0.42 | Open Source |
| *Mustela nivalis* | Native | 39.26 | -0.35 | Open Source |
| *Mustela nivalis* | Native | 38.79 | -0.51 | Open Source |
| *Mustela nivalis* | Native | 39.86 | -0.46 | Open Source |
| *Mustela nivalis* | Native | 39.97 | -0.58 | Open Source |
| *Mustela nivalis* | Native | 40.38 | 0.14 | Open Source |
| *Mustela nivalis* | Native | 38.48 | -0.69 | Open Source |
| *Mustela nivalis* | Native | 38.49 | -0.66 | Open Source |
| *Mustela nivalis* | Native | 40.44 | 0.1 | Open Source |
| *Mustela nivalis* | Native | 38.79 | -0.44 | Open Source |
| *Mustela nivalis* | Native | 39.9 | -0.45 | Open Source |
| *Mustela nivalis* | Native | 39.73 | -0.53 | Open Source |
| *Mustela nivalis* | Native | 39.69 | -0.63 | Open Source |
| *Mustela nivalis* | Native | 39.89 | -0.46 | Open Source |
| *Mustela nivalis* | Native | 39.6 | -0.5 | Open Source |
| *Mustela nivalis* | Native | 38.66 | -0.529999999 | Open Source |
| *Mustela nivalis* | Native | 39.59 | -0.71 | Open Source |
| *Mustela nivalis* | Native | 39.9 | -0.33 | Open Source |
| *Mustela nivalis* | Native | 39.88 | -0.48 | Open Source |
| *Mustela nivalis* | Native | 38.66 | -0.529999999 | Open Source |
| *Mustela nivalis* | Native | 39.6 | -0.460000001 | Open Source |
| *Mustela nivalis* | Native | 39.6 | -0.47 | Open Source |
| *Mustela nivalis* | Native | 39.07 | -0.519999999 | Open Source |
| *Mustela nivalis* | Native | 38.88 | -0.48 | Open Source |
| *Mustela nivalis* | Native | 39.1 | -0.46 | Open Source |
| *Mustela nivalis* | Native | 39.07 | -0.519999999 | Open Source |
| *Mustela nivalis* | Native | 39.6 | -0.73 | Open Source |
| *Mustela nivalis* | Native | 38.72 | -0.61 | Open Source |
| *Mustela nivalis* | Native | 38.71 | -0.52 | Open Source |
| *Mustela nivalis* | Native | 38.69 | -0.58 | Open Source |
| *Mustela nivalis* | Native | 38.77 | -0.54 | Open Source |
| *Mustela nivalis* | Native | 38.7 | -0.62 | Open Source |
| *Mustela nivalis* | Native | 38.71 | -0.63 | Open Source |
| *Mustela nivalis* | Native | 38.66 | -0.529999999 | Open Source |
| *Mustela nivalis* | Native | 38.72 | -0.72 | Open Source |
| *Mustela nivalis* | Native | 38.7 | -0.8 | Open Source |
| *Mustela nivalis* | Native | 38.75 | -0.909999999 | Open Source |
| *Mustela nivalis* | Native | 39.03 | -0.18 | Open Source |
| *Mustela nivalis* | Native | 38.65 | -0.55 | Open Source |
| *Mustela nivalis* | Native | 38.74 | -6.00E-02 | Open Source |
| *Mustela nivalis* | Native | 38.91 | -0.26 | Open Source |
| *Mustela nivalis* | Native | 38.67 | -0.56 | Open Source |
| *Mustela nivalis* | Native | 39.6 | -0.47 | Open Source |
| *Mustela nivalis* | Native | 38.75 | 7.00E-02 | Open Source |
| *Mustela nivalis* | Native | 38.75 | 7.00E-02 | Open Source |
| *Mustela nivalis* | Native | 38.84 | -0.49 | Open Source |
| *Mustela nivalis* | Native | 38.69 | -0.8 | Open Source |
| *Mustela nivalis* | Native | 38.79 | -0.5 | Open Source |
| *Mustela nivalis* | Native | 38.65 | -0.55 | Open Source |
| *Mustela nivalis* | Native | 40.08 | 5.00E-02 | Open Source |
| *Mustela nivalis* | Native | 40.02 | -1.26 | Open Source |
| *Mustela nivalis* | Native | 40.2 | 4.00E-02 | Open Source |
| *Mustela nivalis* | Native | 38.71 | -0.51 | Open Source |
| *Mustela nivalis* | Native | 39.77 | -0.159999999 | Open Source |
| *Mustela nivalis* | Native | 38.87 | -0.72 | Open Source |
| *Mustela nivalis* | Native | 40.11 | -1.37 | Open Source |
| *Mustela nivalis* | Native | 38.75 | -0.18 | Open Source |
| *Mustela nivalis* | Native | 39.24 | -0.89 | Open Source |
| *Mustela nivalis* | Native | 38.78 | -0.4 | Open Source |
| *Mustela nivalis* | Native | 38.96 | -0.92 | Open Source |
| *Mustela nivalis* | Native | 39.14 | -1.06 | Open Source |
| *Mustela nivalis* | Native | 40.05 | -1.35 | Open Source |
| *Mustela nivalis* | Native | 39.59 | -0.73 | Open Source |
| *Mustela nivalis* | Native | 38.85 | -0.52 | Open Source |
| *Mustela nivalis* | Native | 38.73 | -6.00E-02 | Open Source |
| *Mustela nivalis* | Native | 39.83 | -0.490000001 | Open Source |
| *Mustela nivalis* | Native | 38.6 | -0.8 | Open Source |
| *Mustela nivalis* | Native | 38.78 | 2.00E-02 | Open Source |
| *Mustela nivalis* | Native | 38.77 | -7.00E-02 | Open Source |
| *Mustela nivalis* | Native | 38.88 | -0.45 | Open Source |
| *Mustela nivalis* | Native | 40.05 | -1.35 | Open Source |
| *Mustela nivalis* | Native | 39.65 | -0.61 | Open Source |
| *Mustela nivalis* | Native | 39.19 | -0.44 | Open Source |
| *Mustela nivalis* | Native | 38.68 | -0.56 | Open Source |
| *Mustela nivalis* | Native | 40.53 | 0.33 | Open Source |
| *Mustela nivalis* | Native | 39.03 | -0.64 | Open Source |
| *Mustela nivalis* | Native | 38.64 | -0.759999999 | Open Source |
| *Mustela nivalis* | Native | 38.79 | -0.5 | Open Source |
| *Mustela nivalis* | Native | 38.78 | -0.510000001 | Open Source |
| *Mustela nivalis* | Native | 38.65 | -0.79 | Open Source |
| *Mustela nivalis* | Native | 38.66 | -0.54 | Open Source |
| *Mustela nivalis* | Native | 38.91 | -0.26 | Open Source |
| *Mustela nivalis* | Native | 39.62 | -0.28 | Open Source |
| *Mustela nivalis* | Native | 38.66 | -0.54 | Open Source |
| *Mustela nivalis* | Native | 39.6 | -0.679999999 | Open Source |
| *Mustela nivalis* | Native | 39.61 | -0.68 | Open Source |
| *Mustela nivalis* | Native | 39.53 | -0.5 | Open Source |
| *Mustela nivalis* | Native | 39.6 | -0.7 | Open Source |
| *Mustela nivalis* | Native | 39.42 | -1.169999999 | Open Source |
| *Mustela nivalis* | Native | 39.24 | -1.04 | Open Source |
| *Mustela nivalis* | Native | 39.7 | -0.47 | Open Source |
| *Mustela nivalis* | Native | 38.57 | -0.29 | Open Source |
| *Mustela nivalis* | Native | 39.18 | -1.09 | Open Source |
| *Mustela nivalis* | Native | 38.66 | -0.529999999 | Open Source |
| *Mustela nivalis* | Native | 39.95 | -0.7 | Open Source |
| *Mustela nivalis* | Native | 38.71 | -0.56 | Open Source |
| *Mustela nivalis* | Native | 38.05 | -0.82 | Open Source |
| *Mustela nivalis* | Native | 38.66 | -0.54 | Open Source |
| *Mustela nivalis* | Native | 38.66 | -0.529999999 | Open Source |
| *Mustela nivalis* | Native | 38.67 | -0.52 | Open Source |
| *Mustela nivalis* | Native | 38.66 | -0.5 | Open Source |
| *Mustela nivalis* | Native | 38.67 | -0.52 | Open Source |
| *Mustela nivalis* | Native | 39.95 | -0.449999999 | Open Source |
| *Mustela nivalis* | Native | 58.920682 | 17.1415 | Open Source |
| *Mustela nivalis* | Native | 55.676314 | 14.232638 | Open Source |
| *Mustela nivalis* | Native | 58.162384 | 13.489165 | Open Source |
| *Mustela nivalis* | Native | 55.696282 | 13.553871 | Open Source |
| *Mustela nivalis* | Native | 55.69336 | 14.130706 | Open Source |
| *Mustela nivalis* | Native | 59.839213 | 17.553036 | Open Source |
| *Mustela nivalis* | Native | 59.160522 | 16.021696 | Open Source |
| *Mustela nivalis* | Native | 57.910983 | 16.53952 | Open Source |
| *Mustela nivalis* | Native | 56.593394 | 15.682501 | Open Source |
| *Mustela nivalis* | Native | 57.338737 | 13.616933 | Open Source |
| *Mustela nivalis* | Native | 55.392847 | 12.850957 | Open Source |
| *Mustela nivalis* | Native | 56.741052 | 12.624168 | Open Source |
| *Mustela nivalis* | Native | 59.766558 | 17.059618 | Open Source |
| *Mustela nivalis* | Native | 57.89536 | 16.08359 | Open Source |
| *Mustela nivalis* | Native | 56.861893 | 16.853787 | Open Source |
| *Mustela nivalis* | Native | 59.85067 | 16.249751 | Open Source |
| *Mustela nivalis* | Native | 55.901147 | 12.828382 | Open Source |
| *Mustela nivalis* | Native | 57.707741 | 11.789068 | Open Source |
| *Mustela nivalis* | Native | 56.566429 | 14.861791 | Open Source |
| *Mustela nivalis* | Native | 59.846808 | 17.679896 | Open Source |
| *Mustela nivalis* | Native | 57.591589 | 13.918353 | Open Source |
| *Mustela nivalis* | Native | 55.829003 | 14.092573 | Open Source |
| *Mustela nivalis* | Native | 57.017136 | 12.341181 | Open Source |
| *Mustela nivalis* | Native | 57.699284 | 12.053047 | Open Source |
| *Mustela nivalis* | Native | 56.02255 | 13.272384 | Open Source |
| *Mustela nivalis* | Native | 57.490681 | 14.107601 | Open Source |
| *Mustela nivalis* | Native | 57.699937 | 12.052777 | Open Source |
| *Mustela nivalis* | Native | 57.700095 | 12.051879 | Open Source |
| *Mustela nivalis* | Native | 57.699937 | 12.052777 | Open Source |
| *Mustela nivalis* | Native | 57.700095 | 12.051879 | Open Source |
| *Mustela nivalis* | Native | 57.698285 | 12.0539 | Open Source |
| *Mustela nivalis* | Native | 58.915933 | 12.360782 | Open Source |
| *Mustela nivalis* | Native | 58.332179 | 14.822921 | Open Source |
| *Mustela nivalis* | Native | 56.654051 | 16.54218 | Open Source |
| *Mustela nivalis* | Native | 60.433842 | 18.426701 | Open Source |
| *Mustela nivalis* | Native | 60.109633 | 18.470189 | Open Source |
| *Mustela nivalis* | Native | 59.33575 | 18.899251 | Open Source |
| *Mustela nivalis* | Native | 57.827248 | 12.388325 | Open Source |
| *Mustela nivalis* | Native | 60.506972 | 17.673006 | Open Source |
| *Mustela nivalis* | Native | 59.641401 | 15.065646 | Open Source |
| *Mustela nivalis* | Native | 57.697071 | 12.054214 | Open Source |
| *Mustela nivalis* | Native | 57.633326 | 11.911373 | Open Source |
| *Mustela nivalis* | Native | 60.133525 | 15.270174 | Open Source |
| *Mustela nivalis* | Native | 57.835202 | 11.906468 | Open Source |
| *Mustela nivalis* | Native | 58.441333 | 11.434718 | Open Source |
| *Mustela nivalis* | Native | 60.270319 | 13.018376 | Open Source |
| *Mustela nivalis* | Native | 57.544597 | 12.042168 | Open Source |
| *Mustela nivalis* | Native | 57.700374 | 12.052184 | Open Source |
| *Mustela nivalis* | Native | 57.936095 | 14.076196 | Open Source |
| *Mustela nivalis* | Native | 57.018227 | 12.340454 | Open Source |
| *Mustela nivalis* | Native | 59.672932 | 17.36704 | Open Source |
| *Mustela nivalis* | Native | 59.533722 | 15.08257 | Open Source |
| *Mustela nivalis* | Native | 57.01787 | 14.729676 | Open Source |
| *Mustela nivalis* | Native | 58.305947 | 14.848954 | Open Source |
| *Mustela nivalis* | Native | 57.765788 | 11.939643 | Open Source |
| *Mustela nivalis* | Native | 56.38853 | 16.436663 | Open Source |
| *Mustela nivalis* | Native | 56.676093 | 16.62027 | Open Source |
| *Mustela nivalis* | Native | 57.549061 | 15.113158 | Open Source |
| *Mustela nivalis* | Native | 59.119106 | 15.232858 | Open Source |
| *Mustela nivalis* | Native | 61.15361 | 24.038107 | Open Source |
| *Mustela nivalis* | Native | 56.408619 | 12.827089 | Open Source |
| *Mustela nivalis* | Native | 57.401459 | 14.078244 | Open Source |
| *Mustela nivalis* | Native | 57.078659 | 12.408869 | Open Source |
| *Mustela nivalis* | Native | 60.439117 | 16.459517 | Open Source |
| *Mustela nivalis* | Native | 56.828962 | 13.787603 | Open Source |
| *Mustela nivalis* | Native | 59.654756 | 17.882286 | Open Source |
| *Mustela nivalis* | Native | 58.284212 | 13.493845 | Open Source |
| *Mustela nivalis* | Native | 59.665261 | 18.347685 | Open Source |
| *Mustela nivalis* | Native | 59.765644 | 16.205536 | Open Source |
| *Mustela nivalis* | Native | 56.737889 | 16.522291 | Open Source |
| *Mustela nivalis* | Native | 59.917207 | 18.150667 | Open Source |
| *Mustela nivalis* | Native | 60.348075 | 15.918946 | Open Source |
| *Mustela nivalis* | Native | 59.276566 | 15.267614 | Open Source |
| *Mustela nivalis* | Native | 57.000848 | 14.032188 | Open Source |
| *Mustela nivalis* | Native | 58.490519 | 15.465405 | Open Source |
| *Mustela nivalis* | Native | 59.305444 | 15.213643 | Open Source |
| *Mustela nivalis* | Native | 57.721766 | 12.836746 | Open Source |
| *Mustela nivalis* | Native | 56.799671 | 13.805705 | Open Source |
| *Mustela nivalis* | Native | 58.548098 | 13.306538 | Open Source |
| *Mustela nivalis* | Native | 60.428908 | 17.361533 | Open Source |
| *Mustela nivalis* | Native | 58.977832 | 16.350533 | Open Source |
| *Mustela nivalis* | Native | 58.161635 | 13.943973 | Open Source |
| *Mustela nivalis* | Native | 56.250522 | 16.48228 | Open Source |
| *Mustela nivalis* | Native | 56.884682 | 16.191908 | Open Source |
| *Mustela nivalis* | Native | 57.716422 | 12.986414 | Open Source |
| *Mustela nivalis* | Native | 58.462789 | 12.171013 | Open Source |
| *Mustela nivalis* | Native | 58.374579 | 15.610959 | Open Source |
| *Mustela nivalis* | Native | 58.374579 | 15.610959 | Open Source |
| *Mustela nivalis* | Native | 57.527229 | 16.078793 | Open Source |
| *Mustela nivalis* | Native | 57.721099 | 11.716475 | Open Source |
| *Mustela nivalis* | Native | 58.426648 | 15.654087 | Open Source |
| *Mustela nivalis* | Native | 60.149509 | 17.696317 | Open Source |
| *Mustela nivalis* | Native | 58.42622 | 15.649658 | Open Source |
| *Mustela nivalis* | Native | 57.056277 | 16.455636 | Open Source |
| *Mustela nivalis* | Native | 57.412086 | 14.106326 | Open Source |
| *Mustela nivalis* | Native | 56.390449 | 16.544434 | Open Source |
| *Mustela nivalis* | Native | 57.381592 | 14.656194 | Open Source |
| *Mustela nivalis* | Native | 57.41331 | 14.105329 | Open Source |
| *Mustela nivalis* | Native | 57.768294 | 11.937811 | Open Source |
| *Mustela nivalis* | Native | 57.842403 | 12.083823 | Open Source |
| *Mustela nivalis* | Native | 56.638824 | 15.353951 | Open Source |
| *Mustela nivalis* | Native | 59.414986 | 17.871866 | Open Source |
| *Mustela nivalis* | Native | 58.808388 | 17.398804 | Open Source |
| *Mustela nivalis* | Native | 59.322081 | 16.958576 | Open Source |
| *Mustela nivalis* | Native | 59.582609 | 18.208716 | Open Source |
| *Mustela nivalis* | Native | 57.533985 | 14.217609 | Open Source |
| *Mustela nivalis* | Native | 57.400869 | 15.227468 | Open Source |
| *Mustela nivalis* | Native | 58.383862 | 12.342044 | Open Source |
| *Mustela nivalis* | Native | 58.383872 | 12.341756 | Open Source |
| *Mustela nivalis* | Native | 57.54976 | 12.884689 | Open Source |
| *Mustela nivalis* | Native | 57.958153 | 15.828252 | Open Source |
| *Mustela nivalis* | Native | 58.402732 | 11.553071 | Open Source |
| *Mustela nivalis* | Native | 58.621101 | 16.094639 | Open Source |
| *Mustela nivalis* | Native | 59.160494 | 15.375537 | Open Source |
| *Mustela nivalis* | Native | 58.620259 | 16.093678 | Open Source |
| *Mustela nivalis* | Native | 55.412826 | 12.864486 | Open Source |
| *Mustela nivalis* | Native | 57.593712 | 12.070097 | Open Source |
| *Mustela nivalis* | Native | 57.911503 | 16.54014 | Open Source |
| *Mustela nivalis* | Native | 58.67053 | 13.633237 | Open Source |
| *Mustela nivalis* | Native | 56.90311 | 14.871717 | Open Source |
| *Mustela nivalis* | Native | 57.958153 | 15.828252 | Open Source |
| *Mustela nivalis* | Native | 56.064187 | 13.011432 | Open Source |
| *Mustela nivalis* | Native | 57.910191 | 16.545566 | Open Source |
| *Mustela nivalis* | Native | 56.626309 | 16.543383 | Open Source |
| *Mustela nivalis* | Native | 57.739425 | 11.741861 | Open Source |
| *Mustela nivalis* | Native | 58.895896 | 17.516169 | Open Source |
| *Mustela nivalis* | Native | 57.319956 | 15.141886 | Open Source |
| *Mustela nivalis* | Native | 59.833692 | 17.689041 | Open Source |
| *Mustela nivalis* | Native | 59.187202 | 15.236245 | Open Source |
| *Mustela nivalis* | Native | 59.166844 | 17.726833 | Open Source |
| *Mustela nivalis* | Native | 56.419502 | 14.528103 | Open Source |
| *Mustela nivalis* | Native | 57.680928 | 11.947782 | Open Source |
| *Mustela nivalis* | Native | 56.957525 | 16.791426 | Open Source |
| *Mustela nivalis* | Native | 58.903413 | 11.942679 | Open Source |
| *Mustela nivalis* | Native | 55.567203 | 12.899691 | Open Source |
| *Mustela nivalis* | Native | 55.574872 | 13.277872 | Open Source |
| *Mustela nivalis* | Native | 57.346502 | 12.553678 | Open Source |
| *Mustela nivalis* | Native | 55.500453 | 14.325137 | Open Source |
| *Mustela nivalis* | Native | 56.361703 | 13.684953 | Open Source |
| *Mustela nivalis* | Native | 59.177022 | 15.377307 | Open Source |
| *Mustela nivalis* | Native | 56.341046 | 14.942585 | Open Source |
| *Mustela nivalis* | Native | 57.356805 | 14.867468 | Open Source |
| *Mustela nivalis* | Native | 57.252031 | 15.255163 | Open Source |
| *Mustela nivalis* | Native | 58.811398 | 16.733413 | Open Source |
| *Mustela nivalis* | Native | 58.67053 | 13.633237 | Open Source |
| *Mustela nivalis* | Native | 57.697225 | 12.053972 | Open Source |
| *Mustela nivalis* | Native | 55.529373 | 13.420121 | Open Source |
| *Mustela nivalis* | Native | 55.371234 | 13.088346 | Open Source |
| *Mustela nivalis* | Native | 59.758813 | 12.470736 | Open Source |
| *Mustela nivalis* | Native | 59.107181 | 12.648576 | Open Source |
| *Mustela nivalis* | Native | 58.588315 | 13.116715 | Open Source |
| *Mustela nivalis* | Native | 58.355968 | 13.903783 | Open Source |
| *Mustela nivalis* | Native | 60.625245 | 17.178295 | Open Source |
| *Mustela nivalis* | Native | 56.33654 | 12.746878 | Open Source |
| *Mustela nivalis* | Native | 57.853752 | 14.305985 | Open Source |
| *Mustela nivalis* | Native | 59.260595 | 15.166697 | Open Source |
| *Mustela nivalis* | Native | 57.088042 | 12.315408 | Open Source |
| *Mustela nivalis* | Native | 56.102888 | 13.716214 | Open Source |
| *Mustela nivalis* | Native | 57.808756 | 12.074373 | Open Source |
| *Mustela nivalis* | Native | 58.19544 | 13.970006 | Open Source |
| *Mustela nivalis* | Native | 58.710084 | 13.810277 | Open Source |
| *Mustela nivalis* | Native | 59.762573 | 16.211555 | Open Source |
| *Mustela nivalis* | Native | 60.461915 | 15.880723 | Open Source |
| *Mustela nivalis* | Native | 57.796368 | 12.300389 | Open Source |
| *Mustela nivalis* | Native | 55.598688 | 13.777066 | Open Source |
| *Mustela nivalis* | Native | 56.593394 | 15.682501 | Open Source |
| *Mustela nivalis* | Native | 61.093887 | 14.657092 | Open Source |
| *Mustela nivalis* | Native | 56.609628 | 12.924987 | Open Source |
| *Mustela nivalis* | Native | 61.258825 | 15.022895 | Open Source |
| *Mustela nivalis* | Native | 59.789162 | 16.238208 | Open Source |
| *Mustela nivalis* | Native | 60.068068 | 18.524249 | Open Source |
| *Mustela nivalis* | Native | 59.766612 | 16.190193 | Open Source |
| *Mustela nivalis* | Native | 61.061456 | 16.318292 | Open Source |
| *Mustela nivalis* | Native | 59.154374 | 18.022199 | Open Source |
| *Mustela nivalis* | Native | 56.004713 | 14.259956 | Open Source |
| *Mustela nivalis* | Native | 59.830329 | 18.734949 | Open Source |
| *Mustela nivalis* | Native | 59.797004 | 16.223727 | Open Source |
| *Mustela nivalis* | Native | 59.780744 | 16.227796 | Open Source |
| *Mustela nivalis* | Native | 60.178301 | 17.780112 | Open Source |
| *Mustela nivalis* | Native | 60.178301 | 17.780112 | Open Source |
| *Mustela nivalis* | Native | 59.705479 | 14.538344 | Open Source |
| *Mustela nivalis* | Native | 59.675504 | 16.031658 | Open Source |
| *Mustela nivalis* | Native | 57.847887 | 14.839459 | Open Source |
| *Mustela nivalis* | Native | 59.199629 | 15.248722 | Open Source |
| *Mustela nivalis* | Native | 59.325812 | 17.085912 | Open Source |
| *Mustela nivalis* | Native | 58.049656 | 15.060148 | Open Source |
| *Mustela nivalis* | Native | 59.767526 | 16.03765 | Open Source |
| *Mustela nivalis* | Native | 59.925514 | 17.337692 | Open Source |
| *Mustela nivalis* | Native | 58.539786 | 15.748428 | Open Source |
| *Mustela nivalis* | Native | 58.596975 | 16.366271 | Open Source |
| *Mustela nivalis* | Native | 60.083412 | 15.269348 | Open Source |
| *Mustela nivalis* | Native | 59.766839 | 16.189483 | Open Source |
| *Mustela nivalis* | Native | 59.322718 | 14.762042 | Open Source |
| *Mustela nivalis* | Native | 60.666251 | 17.303133 | Open Source |
| *Mustela nivalis* | Native | 59.239105 | 16.920757 | Open Source |
| *Mustela nivalis* | Native | 58.797696 | 17.465136 | Open Source |
| *Mustela nivalis* | Native | 59.314252 | 5.536164 | Open Source |
| *Mustela nivalis* | Native | 58.379398 | 6.896492 | Open Source |
| *Mustela nivalis* | Native | 59.646055 | 10.510118 | Open Source |
| *Mustela nivalis* | Native | 59.315755 | 11.740181 | Open Source |
| *Mustela nivalis* | Native | 60.523504 | 8.123087999 | Open Source |
| *Mustela nivalis* | Native | 60.74881 | 11.205109 | Open Source |
| *Mustela nivalis* | Native | 59.235328 | 10.244208 | Open Source |
| *Mustela nivalis* | Native | 59.281091 | 10.139042 | Open Source |
| *Mustela nivalis* | Native | 60.795332 | 10.772336 | Open Source |
| *Mustela nivalis* | Native | 58.103879 | 6.607235001 | Open Source |
| *Mustela nivalis* | Native | 51.406555 | 9.895678 | Open Source |
| *Mustela nivalis* | Native | 50.00069 | 8.561525 | Open Source |
| *Mustela nivalis* | Native | 51.389927 | 9.625397 | Open Source |
| *Mustela nivalis* | Native | 52.502525 | 8.514441 | Open Source |
| *Mustela nivalis* | Native | 51.059093 | 9.285679 | Open Source |
| *Mustela nivalis* | Native | 53.724442 | 10.178318 | Open Source |
| *Mustela nivalis* | Native | 50.767395 | 6.48622 | Open Source |
| *Mustela nivalis* | Native | 50.187229 | 7.026014 | Open Source |
| *Mustela nivalis* | Native | 50.991711 | 6.822599 | Open Source |
| *Mustela nivalis* | Native | 51.772831 | 9.923015 | Open Source |
| *Mustela nivalis* | Native | 47.241016 | 12.822418 | Open Source |
| *Mustela nivalis* | Native | 48.79044 | 9.489226 | Open Source |
| *Mustela nivalis* | Native | 50.098499 | 10.208167 | Open Source |
| *Mustela nivalis* | Native | 51.140156 | 9.150109001 | Open Source |
| *Mustela nivalis* | Native | 60.320827 | 7.158966 | Open Source |
| *Mustela nivalis* | Native | 52.566124 | 13.728447 | Open Source |
| *Mustela nivalis* | Native | 51.590508 | 7.409763001 | Open Source |
| *Mustela nivalis* | Native | 51.336617 | 9.855794 | Open Source |
| *Mustela nivalis* | Native | 51.480686 | 7.624598 | Open Source |
| *Mustela nivalis* | Native | 54.030125 | 11.524227 | Open Source |
| *Mustela nivalis* | Native | 53.904415 | 10.721283 | Open Source |
| *Mustela nivalis* | Native | 50.217339 | 10.236168 | Open Source |
| *Mustela nivalis* | Native | 53.723427 | 9.040586001 | Open Source |
| *Mustela nivalis* | Native | 60.485954 | 7.101067999 | Open Source |
| *Mustela nivalis* | Native | 50.699196 | 7.225466 | Open Source |
| *Mustela nivalis* | Native | 52.083385 | 9.803657 | Open Source |
| *Mustela nivalis* | Native | 51.937702 | 8.445225 | Open Source |
| *Mustela nivalis* | Native | 49.501144 | 7.777006 | Open Source |
| *Mustela nivalis* | Native | 61.197704 | 6.441374001 | Open Source |
| *Mustela nivalis* | Native | 53.64082 | 10.874751 | Open Source |
| *Mustela nivalis* | Native | 49.094959 | 9.701114 | Open Source |
| *Mustela nivalis* | Native | 51.55624 | 9.956178 | Open Source |
| *Mustela nivalis* | Native | 50.128666 | 8.583713 | Open Source |
| *Mustela nivalis* | Native | 51.117023 | 7.956806001 | Open Source |
| *Mustela nivalis* | Native | 54.10128 | 12.707062 | Open Source |
| *Mustela nivalis* | Native | 50.222939 | 10.103989 | Open Source |
| *Mustela nivalis* | Native | 54.176052 | 10.550008 | Open Source |
| *Mustela nivalis* | Native | 51.804241 | 9.111259999 | Open Source |
| *Mustela nivalis* | Native | 52.027332 | 7.650646999 | Open Source |
| *Mustela nivalis* | Native | 49.120209 | 10.391168 | Open Source |
| *Mustela nivalis* | Native | 52.854805 | 13.708167 | Open Source |
| *Mustela nivalis* | Native | 52.027332 | 7.650646999 | Open Source |
| *Mustela nivalis* | Native | 50.098499 | 10.208167 | Open Source |
| *Mustela nivalis* | Native | 50.982452 | 6.824702 | Open Source |
| *Mustela nivalis* | Native | 50.854496 | 6.103678 | Open Source |
| *Mustela nivalis* | Native | 46.918262 | 10.062532 | Open Source |
| *Mustela nivalis* | Native | 47.765175 | 16.827621 | Open Source |
| *Mustela nivalis* | Native | 53.864159 | 9.844558001 | Open Source |
| *Mustela nivalis* | Native | 52.498249 | 13.042145 | Open Source |
| *Mustela nivalis* | Native | 47.866116 | 12.008657 | Open Source |
| *Mustela nivalis* | Native | 51.480099 | 9.817742999 | Open Source |
| *Mustela nivalis* | Native | 52.723923 | 14.096117 | Open Source |
| *Mustela nivalis* | Native | 50.083801 | 10.174971 | Open Source |
| *Mustela nivalis* | Native | 50.173832 | 9.040620001 | Open Source |
| *Mustela nivalis* | Native | 54.277496 | 10.354904 | Open Source |
| *Mustela nivalis* | Native | 51.907768 | 6.743298 | Open Source |
| *Mustela nivalis* | Native | 48.209213 | 13.430529 | Open Source |
| *Mustela nivalis* | Native | 52.136974 | 9.960413 | Open Source |
| *Mustela nivalis* | Native | 47.49807 | 10.314445 | Open Source |
| *Mustela nivalis* | Native | 49.145279 | 10.705876 | Open Source |
| *Mustela nivalis* | Native | 49.743202 | 8.825776999 | Open Source |
| *Mustela nivalis* | Native | 50.549026 | 7.867649 | Open Source |
| *Mustela nivalis* | Native | 49.841415 | 9.564972 | Open Source |
| *Mustela nivalis* | Native | 47.571808 | 12.14176 | Open Source |
| *Mustela nivalis* | Native | 53.275791 | 10.803787 | Open Source |
| *Mustela nivalis* | Native | 53.498871 | 8.55217 | Open Source |
| *Mustela nivalis* | Native | 49.736248 | 8.96906 | Open Source |
| *Mustela nivalis* | Native | 51.715279 | 9.989166001 | Open Source |
| *Mustela nivalis* | Native | 49.921055 | 8.450890001 | Open Source |
| *Mustela nivalis* | Native | 50.377117 | 7.272495 | Open Source |
| *Mustela nivalis* | Native | 37.01609 | -3.603022 | Open Source |
| *Mustela nivalis* | Native | 50.17984 | 10.162911 | Open Source |
| *Mustela nivalis* | Native | 50.966663 | 11.002685 | Open Source |
| *Mustela nivalis* | Native | 53.344608 | 10.503187 | Open Source |
| *Mustela nivalis* | Native | 50.427818 | 8.242393 | Open Source |
| *Mustela nivalis* | Native | 50.173931 | 10.247927 | Open Source |
| *Mustela nivalis* | Native | 49.964638 | 10.183897 | Open Source |
| *Mustela nivalis* | Native | 50.995842 | 14.188448 | Open Source |
| *Mustela nivalis* | Native | 51.268147 | 6.413269 | Open Source |
| *Mustela nivalis* | Native | 50.860962 | 6.153283 | Open Source |
| *Mustela nivalis* | Native | 47.800358 | 10.625487 | Open Source |
| *Mustela nivalis* | Native | 48.93066 | 9.453981999 | Open Source |
| *Mustela nivalis* | Native | 54.17786 | 12.104702 | Open Source |
| *Mustela nivalis* | Native | 52.171959 | 8.263092 | Open Source |
| *Mustela nivalis* | Native | 50.07251 | 10.029659 | Open Source |
| *Mustela nivalis* | Native | 51.824108 | 6.736164 | Open Source |
| *Mustela nivalis* | Native | 47.561005 | 9.921877 | Open Source |
| *Mustela nivalis* | Native | 50.00069 | 8.561525 | Open Source |
| *Mustela nivalis* | Native | 49.773876 | 7.374199999 | Open Source |
| *Mustela nivalis* | Native | 47.792976 | 16.827793 | Open Source |
| *Mustela nivalis* | Native | 53.235806 | 10.427243 | Open Source |
| *Mustela nivalis* | Native | 51.82365 | 10.540377 | Open Source |
| *Mustela nivalis* | Native | 51.961456 | 10.50529 | Open Source |
| *Mustela nivalis* | Native | 49.867477 | 9.726934 | Open Source |
| *Mustela nivalis* | Native | 50.083801 | 10.174971 | Open Source |
| *Mustela nivalis* | Native | 51.314445 | 9.324166001 | Open Source |
| *Mustela nivalis* | Native | 51.91853 | 10.451023 | Open Source |
| *Mustela nivalis* | Native | 52.075497 | 8.498955 | Open Source |
| *Mustela nivalis* | Native | 51.409378 | 9.803753001 | Open Source |
| *Mustela nivalis* | Native | 51.268147 | 6.413269 | Open Source |
| *Mustela nivalis* | Native | 53.380898 | 10.475335 | Open Source |
| *Mustela nivalis* | Native | 50.147511 | 10.736003 | Open Source |
| *Mustela nivalis* | Native | 53.473461 | 10.373711 | Open Source |
| *Mustela nivalis* | Native | 51.406555 | 9.895678 | Open Source |
| *Mustela nivalis* | Native | 52.423611 | 14.123149 | Open Source |
| *Mustela nivalis* | Native | 54.153942 | 12.159129 | Open Source |
| *Mustela nivalis* | Native | 50.787251 | 6.078778 | Open Source |
| *Mustela nivalis* | Native | 51.555515 | 7.565804 | Open Source |
| *Mustela nivalis* | Native | 50.666058 | 8.996215 | Open Source |
| *Mustela nivalis* | Native | 48.907722 | 10.258741 | Open Source |
| *Mustela nivalis* | Native | 51.577206 | 14.612997 | Open Source |
| *Mustela nivalis* | Native | 51.631416 | 9.315854 | Open Source |
| *Mustela nivalis* | Native | 50.147511 | 10.736003 | Open Source |
| *Mustela nivalis* | Native | 52.423611 | 14.123149 | Open Source |
| *Mustela nivalis* | Native | 50.373806 | 6.70763 | Open Source |
| *Mustela nivalis* | Native | 50.278671 | 10.360579 | Open Source |
| *Mustela nivalis* | Native | 50.995842 | 14.188448 | Open Source |
| *Mustela nivalis* | Native | 49.421276 | 8.179793 | Open Source |
| *Mustela nivalis* | Native | 53.689999 | 8.740654 | Open Source |
| *Mustela nivalis* | Native | 49.473911 | 7.707485999 | Open Source |
| *Mustela nivalis* | Native | 53.790714 | 8.549422999 | Open Source |
| *Mustela nivalis* | Native | 51.4743 | 10.840244 | Open Source |
| *Mustela nivalis* | Native | 49.423916 | 6.957594999 | Open Source |
| *Mustela nivalis* | Native | 48.901543 | 9.431526 | Open Source |
| *Mustela nivalis* | Native | 51.560852 | 10.82943 | Open Source |
| *Mustela nivalis* | Native | 51.444164 | 10.982037 | Open Source |
| *Mustela nivalis* | Native | 50.428589 | 12.954597 | Open Source |
| *Mustela nivalis* | Native | 53.70612 | 10.913094 | Open Source |
| *Mustela nivalis* | Native | 53.233833 | 10.918307 | Open Source |
| *Mustela nivalis* | Native | 50.891251 | 6.146421 | Open Source |
| *Mustela nivalis* | Native | 50.849468 | 6.157537 | Open Source |
| *Mustela nivalis* | Native | 53.672104 | 8.817215001 | Open Source |
| *Mustela nivalis* | Native | 52.528648 | 13.580303 | Open Source |
| *Mustela nivalis* | Native | 53.985924 | 10.826854 | Open Source |
| *Mustela nivalis* | Native | 49.923859 | 8.374052999 | Open Source |
| *Mustela nivalis* | Native | 51.393322 | 9.741203 | Open Source |
| *Mustela nivalis* | Native | 51.183754 | 6.745155 | Open Source |
| *Mustela nivalis* | Native | 53.444614 | 10.015411 | Open Source |
| *Mustela nivalis* | Native | 49.722816 | 8.955059 | Open Source |
| *Mustela nivalis* | Native | 49.464314 | 7.602551999 | Open Source |
| *Mustela nivalis* | Native | 52.002811 | 9.860173 | Open Source |
| *Mustela nivalis* | Native | 53.665131 | 8.497892001 | Open Source |
| *Mustela nivalis* | Native | 51.835381 | 7.132359001 | Open Source |
| *Mustela nivalis* | Native | 49.938736 | 8.440247 | Open Source |
| *Mustela nivalis* | Native | 48.492023 | 12.944847 | Open Source |
| *Mustela nivalis* | Native | 52.529922 | 10.186146 | Open Source |
| *Mustela nivalis* | Native | 50.574299 | 8.61105 | Open Source |
| *Mustela nivalis* | Native | 53.197281 | 8.243941 | Open Source |
| *Mustela nivalis* | Native | 53.689999 | 8.740654 | Open Source |
| *Mustela nivalis* | Native | 47.087891 | 11.476593 | Open Source |
| *Mustela nivalis* | Native | 50.147755 | 8.648879 | Open Source |
| *Mustela nivalis* | Native | 50.416176 | 8.227386 | Open Source |
| *Mustela nivalis* | Native | 49.964638 | 10.183897 | Open Source |
| *Mustela nivalis* | Native | 52.083344 | 9.80748 | Open Source |
| *Mustela nivalis* | Native | 50.901138 | 8.038559001 | Open Source |
| *Mustela nivalis* | Native | 53.128441 | 11.251373 | Open Source |
| *Mustela nivalis* | Native | 51.296146 | 9.496305001 | Open Source |
| *Mustela nivalis* | Native | 50.458378 | 10.231361 | Open Source |
| *Mustela nivalis* | Native | 49.790375 | 7.486757 | Open Source |
| *Mustela nivalis* | Native | 48.849197 | 9.403396 | Open Source |
| *Mustela nivalis* | Native | 58.760305 | 5.508865 | Open Source |
| *Mustela nivalis* | Native | 59.588076 | 11.594403 | Open Source |
| *Mustela nivalis* | Native | 59.862254 | 11.080234 | Open Source |
| *Mustela nivalis* | Native | 40.612784 | 19.464486 | Open Source |
| *Mustela nivalis* | Native | 59.769118 | 11.122787 | Open Source |
| *Mustela nivalis* | Native | 58.774926 | 5.528707999 | Open Source |
| *Mustela nivalis* | Native | 59.973768 | 11.138624 | Open Source |
| *Mustela nivalis* | Native | 58.117433 | 7.432104 | Open Source |
| *Mustela nivalis* | Native | 59.345425 | 10.745513 | Open Source |
| *Mustela nivalis* | Native | 59.907007 | 10.679522 | Open Source |
| *Mustela nivalis* | Native | 60.342239 | 11.520319 | Open Source |
| *Mustela nivalis* | Native | 60.369436 | 11.381691 | Open Source |
| *Mustela nivalis* | Native | 58.827867 | 6.283652999 | Open Source |
| *Mustela nivalis* | Native | 59.11794 | 11.660743 | Open Source |
| *Mustela nivalis* | Native | 58.827867 | 6.283652999 | Open Source |
| *Mustela nivalis* | Native | 60.832072 | 11.747152 | Open Source |
| *Mustela nivalis* | Native | 59.178348 | 11.636857 | Open Source |
| *Mustela nivalis* | Native | 60.616744 | 5.372212999 | Open Source |
| *Mustela nivalis* | Native | 60.876899 | 11.020128 | Open Source |
| *Mustela nivalis* | Native | 58.372273 | 24.633145 | Open Source |
| *Mustela nivalis* | Native | 58.372145 | 24.633453 | Open Source |
| *Mustela nivalis* | Native | 42.83 | -1.72 | Open Source |
| *Mustela nivalis* | Native | 42.83 | -1.72 | Open Source |
| *Mustela nivalis* | Native | 42.83 | -1.72 | Open Source |
| *Mustela nivalis* | Native | 60.89537 | 9.640818 | Open Source |
| *Mustela nivalis* | Native | 58.709883 | 9.104848 | Open Source |
| *Mustela nivalis* | Native | 58.827867 | 6.283652999 | Open Source |
| *Mustela nivalis* | Native | 58.600041 | 6.669191999 | Open Source |
| *Mustela nivalis* | Native | 58.883239 | 6.840698 | Open Source |
| *Mustela nivalis* | Native | 59.198976 | 11.523283 | Open Source |
| *Mustela nivalis* | Native | 60.399822 | 10.610074 | Open Source |
| *Mustela nivalis* | Native | 58.883239 | 6.840698 | Open Source |
| *Mustela nivalis* | Native | 58.480288 | 8.566317001 | Open Source |
| *Mustela nivalis* | Native | 60.149268 | 11.560294 | Open Source |
| *Mustela nivalis* | Native | 59.453865 | 11.531377 | Open Source |
| *Mustela nivalis* | Native | 59.30483 | 11.763367 | Open Source |
| *Mustela nivalis* | Native | 60.227454 | 10.977871 | Open Source |
| *Mustela nivalis* | Native | 59.953952 | 10.712104 | Open Source |
| *Mustela nivalis* | Native | 58.520606 | 6.750488999 | Open Source |
| *Mustela nivalis* | Native | 58.136392 | 6.651207 | Open Source |
| *Mustela nivalis* | Native | 58.376331 | 6.900202 | Open Source |
| *Mustela nivalis* | Native | 61.401896 | 7.721918 | Open Source |
| *Mustela nivalis* | Native | 58.556899 | 6.214024001 | Open Source |
| *Mustela nivalis* | Native | 58.742765 | 6.796905001 | Open Source |
| *Mustela nivalis* | Native | 59.191896 | 7.078320001 | Open Source |
| *Mustela nivalis* | Native | 59.309199 | 11.764148 | Open Source |
| *Mustela nivalis* | Native | 59.209885 | 11.60132 | Open Source |
| *Mustela nivalis* | Native | 61.220677 | 10.246642 | Open Source |
| *Mustela nivalis* | Native | 59.390766 | 9.276793999 | Open Source |
| *Mustela nivalis* | Native | 59.072036 | 11.4703 | Open Source |
| *Mustela nivalis* | Native | 59.145243 | 9.932420999 | Open Source |
| *Mustela nivalis* | Native | 59.02608 | 9.685537 | Open Source |
| *Mustela nivalis* | Native | 60.848714 | 12.201924 | Open Source |
| *Mustela nivalis* | Native | 58.185336 | 7.225382999 | Open Source |
| *Mustela nivalis* | Native | 60.898673 | 7.222769 | Open Source |
| *Mustela nivalis* | Native | 60.578468 | 8.432378001 | Open Source |
| *Mustela nivalis* | Native | 59.752262 | 10.441424 | Open Source |
| *Mustela nivalis* | Native | 59.533299 | 11.473876 | Open Source |
| *Mustela nivalis* | Native | 60.260575 | 10.683852 | Open Source |
| *Mustela nivalis* | Native | 58.109024 | 6.568391999 | Open Source |
| *Mustela nivalis* | Native | 59.130368 | 11.487243 | Open Source |
| *Mustela nivalis* | Native | 59.715397 | 8.015353 | Open Source |
| *Mustela nivalis* | Native | 60.160726 | 11.557787 | Open Source |
| *Mustela nivalis* | Native | 61.12868 | 10.394128 | Open Source |
| *Mustela nivalis* | Native | 59.264057 | 10.136284 | Open Source |
| *Mustela nivalis* | Native | 59.154456 | 8.451611001 | Open Source |
| *Mustela nivalis* | Native | 59.714704 | 9.824371 | Open Source |
| *Mustela nivalis* | Native | 61.13385 | 8.906732999 | Open Source |
| *Mustela nivalis* | Native | 58.804089 | 7.497474001 | Open Source |
| *Mustela nivalis* | Native | 58.024695 | 7.368862 | Open Source |
| *Mustela nivalis* | Native | 60.523504 | 8.123087999 | Open Source |
| *Mustela nivalis* | Native | 59.169482 | 8.619003 | Open Source |
| *Mustela nivalis* | Native | 59.749714 | 9.866475 | Open Source |
| *Mustela nivalis* | Native | 60.801577 | 11.336999 | Open Source |
| *Mustela nivalis* | Native | 58.019424 | 7.515988 | Open Source |
| *Mustela nivalis* | Native | 58.862858 | 7.502630001 | Open Source |
| *Mustela nivalis* | Native | 60.331094 | 11.325699 | Open Source |
| *Mustela nivalis* | Native | 58.032039 | 7.511299 | Open Source |
| *Mustela nivalis* | Native | 60.877848 | 10.932461 | Open Source |
| *Mustela nivalis* | Native | 58.817022 | 7.497931999 | Open Source |
| *Mustela nivalis* | Native | 58.402021 | 6.110664 | Open Source |
| *Mustela nivalis* | Native | 58.155342 | 7.982187 | Open Source |
| *Mustela nivalis* | Native | 59.327846 | 10.804209 | Open Source |
| *Mustela nivalis* | Native | 59.084648 | 8.818923 | Open Source |
| *Mustela nivalis* | Native | 59.749714 | 9.866475 | Open Source |
| *Mustela nivalis* | Native | 58.0294 | 7.364488 | Open Source |
| *Mustela nivalis* | Native | 60.525533 | 8.163782 | Open Source |
| *Mustela nivalis* | Native | 60.002188 | 10.797768 | Open Source |
| *Mustela nivalis* | Native | 58.024695 | 7.368862 | Open Source |
| *Mustela nivalis* | Native | 58.188887 | 8.238476 | Open Source |
| *Mustela nivalis* | Native | 59.286978 | 10.205706 | Open Source |
| *Mustela nivalis* | Native | 59.768385 | 7.422950001 | Open Source |
| *Mustela nivalis* | Native | 58.775294 | 5.901527001 | Open Source |
| *Mustela nivalis* | Native | 59.554231 | 9.816313001 | Open Source |
| *Mustela nivalis* | Native | 60.034065 | 10.355913 | Open Source |
| *Mustela nivalis* | Native | 59.352638 | 10.747965 | Open Source |
| *Mustela nivalis* | Native | 59.772497 | 10.287992 | Open Source |
| *Mustela nivalis* | Native | 59.974658 | 10.508295 | Open Source |
| *Mustela nivalis* | Native | 60.924575 | 10.39941 | Open Source |
| *Mustela nivalis* | Native | 59.469471 | 10.340606 | Open Source |
| *Mustela nivalis* | Native | 58.098596 | 7.847403999 | Open Source |
| *Mustela nivalis* | Native | 58.377363 | 6.897642001 | Open Source |
| *Mustela nivalis* | Native | 59.749288 | 6.693823 | Open Source |
| *Mustela nivalis* | Native | 59.345553 | 10.745863 | Open Source |
| *Mustela nivalis* | Native | 58.242601 | 7.154595999 | Open Source |
| *Mustela nivalis* | Native | 58.552761 | 6.193049001 | Open Source |
| *Mustela nivalis* | Native | 58.377292 | 6.897453 | Open Source |
| *Mustela nivalis* | Native | 58.012168 | 7.418431 | Open Source |
| *Mustela nivalis* | Native | 60.095107 | 11.221126 | Open Source |
| *Mustela nivalis* | Native | 59.469471 | 10.340606 | Open Source |
| *Mustela nivalis* | Native | 60.83163 | 11.844 | Open Source |
| *Mustela nivalis* | Native | 60.740012 | 11.365153 | Open Source |
| *Mustela nivalis* | Native | 58.752957 | 7.510581 | Open Source |
| *Mustela nivalis* | Native | 59.208846 | 10.295187 | Open Source |
| *Mustela nivalis* | Native | 60.002188 | 10.797768 | Open Source |
| *Mustela nivalis* | Native | 58.752957 | 7.510581 | Open Source |
| *Mustela nivalis* | Native | 59.554231 | 9.816313001 | Open Source |
| *Mustela nivalis* | Native | 61.090886 | 11.818191 | Open Source |
| *Mustela nivalis* | Native | 60.376812 | 11.60467 | Open Source |
| *Mustela nivalis* | Native | 60.180905 | 6.577240001 | Open Source |
| *Mustela nivalis* | Native | 59.375375 | 10.836359 | Open Source |
| *Mustela nivalis* | Native | 60.701144 | 11.29185 | Open Source |
| *Mustela nivalis* | Native | 60.779825 | 11.669574 | Open Source |
| *Mustela nivalis* | Native | 60.80166 | 11.761939 | Open Source |
| *Mustela nivalis* | Native | 58.752957 | 7.510581 | Open Source |
| *Mustela nivalis* | Native | 61.539566 | 12.477887 | Open Source |
| *Mustela nivalis* | Native | 60.873419 | 11.831378 | Open Source |
| *Mustela nivalis* | Native | 60.728704 | 11.109699 | Open Source |
| *Mustela nivalis* | Native | 60.80166 | 11.797575 | Open Source |
| *Mustela nivalis* | Native | 61.220111 | 10.457333 | Open Source |
| *Mustela nivalis* | Native | 58.533217 | 6.030642 | Open Source |
| *Mustela nivalis* | Native | 61.075892 | 6.438307 | Open Source |
| *Mustela nivalis* | Native | 58.109532 | 6.574285 | Open Source |
| *Mustela nivalis* | Native | 58.752957 | 7.510581 | Open Source |
| *Mustela nivalis* | Native | 58.222175 | 7.958687001 | Open Source |
| *Mustela nivalis* | Native | 58.109185 | 6.568526 | Open Source |
| *Mustela nivalis* | Native | 59.331256 | 11.262772 | Open Source |
| *Mustela nivalis* | Native | 59.212556 | 10.186958 | Open Source |
| *Mustela nivalis* | Native | 60.643143 | 6.443427 | Open Source |
| *Mustela nivalis* | Native | 60.885996 | 11.191751 | Open Source |
| *Mustela nivalis* | Native | 58.243991 | 7.149637 | Open Source |
| *Mustela nivalis* | Native | 58.245669 | 7.167936 | Open Source |
| *Mustela nivalis* | Native | 59.554231 | 9.816313001 | Open Source |
| *Mustela nivalis* | Native | 61.261024 | 10.430761 | Open Source |
| *Mustela nivalis* | Native | 59.728034 | 6.069665001 | Open Source |
| *Mustela nivalis* | Native | 60.589344 | 8.451862 | Open Source |
| *Mustela nivalis* | Native | 59.908547 | 6.634346 | Open Source |
| *Mustela nivalis* | Native | 59.752257 | 6.814359001 | Open Source |
| *Mustela nivalis* | Native | 59.212556 | 10.186958 | Open Source |
| *Mustela nivalis* | Native | 59.97072 | 6.718922 | Open Source |
| *Mustela nivalis* | Native | 59.728034 | 6.069665001 | Open Source |
| *Mustela nivalis* | Native | 58.094983 | 6.628363 | Open Source |
| *Mustela nivalis* | Native | 58.090729 | 6.634993 | Open Source |
| *Mustela nivalis* | Native | 59.173082 | 10.12457 | Open Source |
| *Mustela nivalis* | Native | 60.160726 | 11.557787 | Open Source |
| *Mustela nivalis* | Native | 58.827867 | 6.283652999 | Open Source |
| *Mustela nivalis* | Native | 60.986291 | 8.970163 | Open Source |
| *Mustela nivalis* | Native | 60.918013 | 11.872773 | Open Source |
| *Mustela nivalis* | Native | 58.752957 | 7.510581 | Open Source |
| *Mustela nivalis* | Native | 60.81441 | 11.382634 | Open Source |
| *Mustela nivalis* | Native | 58.724241 | 5.779122999 | Open Source |
| *Mustela nivalis* | Native | 58.267476 | 7.208630001 | Open Source |
| *Mustela nivalis* | Native | 59.212556 | 10.186958 | Open Source |
| *Mustela nivalis* | Native | 59.406844 | 10.81418 | Open Source |
| *Mustela nivalis* | Native | 59.114094 | 11.646613 | Open Source |
| *Mustela nivalis* | Native | 59.101397 | 11.526077 | Open Source |
| *Mustela nivalis* | Native | 60.005493 | 10.489565 | Open Source |
| *Mustela nivalis* | Native | 60.828591 | 11.348812 | Open Source |
| *Mustela nivalis* | Native | 59.532711 | 10.350973 | Open Source |
| *Mustela nivalis* | Native | 60.18363 | 12.052795 | Open Source |
| *Mustela nivalis* | Native | 58.651725 | 5.909361 | Open Source |
| *Mustela nivalis* | Native | 58.733525 | 5.585464 | Open Source |
| *Mustela nivalis* | Native | 58.099317 | 6.583528 | Open Source |
| *Mustela nivalis* | Native | 59.824527 | 17.578126 | Open Source |
| *Mustela nivalis* | Native | 55.510145 | 14.346868 | Open Source |
| *Mustela nivalis* | Native | 58.893209 | 16.884779 | Open Source |
| *Mustela nivalis* | Native | 61.396211 | 17.038373 | Open Source |
| *Mustela nivalis* | Native | 58.302069 | 26.763831 | Open Source |
| *Mustela nivalis* | Native | 50.383374 | 6.682574999 | Open Source |
| *Mustela nivalis* | Native | 59.520242 | 14.985264 | Open Source |
| *Mustela nivalis* | Native | 56.377937 | 16.072909 | Open Source |
| *Mustela nivalis* | Native | 57.700095 | 12.051879 | Open Source |
| *Mustela nivalis* | Native | 57.700095 | 12.051879 | Open Source |
| *Mustela nivalis* | Native | 57.698885 | 12.054538 | Open Source |
| *Mustela nivalis* | Native | 56.068604 | 14.472641 | Open Source |
| *Mustela nivalis* | Native | 57.700254 | 12.051798 | Open Source |
| *Mustela nivalis* | Native | 57.083024 | 15.883562 | Open Source |
| *Mustela nivalis* | Native | 50.383419 | 6.682558 | Open Source |
| *Mustela nivalis* | Native | 52.082005 | 23.719866 | Open Source |
| *Mustela nivalis* | Native | 56.276814 | 16.486969 | Open Source |
| *Mustela nivalis* | Native | 59.827471 | 17.664894 | Open Source |
| *Mustela nivalis* | Native | 56.873161 | 15.67023 | Open Source |
| *Mustela nivalis* | Native | 55.407946 | 12.841112 | Open Source |
| *Mustela nivalis* | Native | 59.725629 | 16.365166 | Open Source |
| *Mustela nivalis* | Native | 60.202353 | 18.33316 | Open Source |
| *Mustela nivalis* | Native | 58.405941 | 12.270286 | Open Source |
| *Mustela nivalis* | Native | 58.841851 | 12.54818 | Open Source |
| *Mustela nivalis* | Native | 56.197064 | 14.458205 | Open Source |
| *Mustela nivalis* | Native | 57.993251 | 14.911522 | Open Source |
| *Mustela nivalis* | Native | 58.123595 | 13.510545 | Open Source |
| *Mustela nivalis* | Native | 57.684021 | 11.938709 | Open Source |
| *Mustela nivalis* | Native | 60.808549 | 13.674128 | Open Source |
| *Mustela nivalis* | Native | 58.587187 | 16.16292 | Open Source |
| *Mustela nivalis* | Native | 59.734889 | 18.189654 | Open Source |
| *Mustela nivalis* | Native | 60.499599 | 15.811382 | Open Source |
| *Mustela nivalis* | Native | 59.294698 | 18.049903 | Open Source |
| *Mustela nivalis* | Native | 59.186664 | 14.113279 | Open Source |
| *Mustela nivalis* | Native | 45.221054 | 8.21736 | Open Source |
| *Mustela nivalis* | Native | 60.637321 | 16.770909 | Open Source |
| *Mustela nivalis* | Native | 58.190166 | 13.942563 | Open Source |
| *Mustela nivalis* | Native | 57.018227 | 12.340454 | Open Source |
| *Mustela nivalis* | Native | 57.704204 | 12.055777 | Open Source |
| *Mustela nivalis* | Native | 61.396443 | 14.828787 | Open Source |
| *Mustela nivalis* | Native | 57.696255 | 12.053559 | Open Source |
| *Mustela nivalis* | Native | 58.225093 | 11.900854 | Open Source |
| *Mustela nivalis* | Native | 60.458806 | 16.047756 | Open Source |
| *Mustela nivalis* | Native | 60.153967 | 17.697036 | Open Source |
| *Mustela nivalis* | Native | 59.843604 | 17.667867 | Open Source |
| *Mustela nivalis* | Native | 56.1288 | 12.815258 | Open Source |
| *Mustela nivalis* | Native | 57.174135 | 14.013018 | Open Source |
| *Mustela nivalis* | Native | 60.624408 | 15.468585 | Open Source |
| *Mustela nivalis* | Native | 57.700095 | 12.051879 | Open Source |
| *Mustela nivalis* | Native | 58.447473 | 15.577604 | Open Source |
| *Mustela nivalis* | Native | 59.590758 | 15.197923 | Open Source |
| *Mustela nivalis* | Native | 57.828324 | 14.840456 | Open Source |
| *Mustela nivalis* | Native | 59.825755 | 17.801429 | Open Source |
| *Mustela nivalis* | Native | 57.461161 | 12.58961 | Open Source |
| *Mustela nivalis* | Native | 60.229292 | 15.778037 | Open Source |
| *Mustela nivalis* | Native | 61.573881 | 14.305815 | Open Source |
| *Mustela nivalis* | Native | 58.013643 | 14.133581 | Open Source |
| *Mustela nivalis* | Native | 60.454128 | 15.275375 | Open Source |
| *Mustela nivalis* | Native | 51.162712 | 9.119468 | Open Source |
| *Mustela nivalis* | Native | 48.295315 | 13.277386 | Open Source |
| *Mustela nivalis* | Native | 53.425205 | 10.16201 | Open Source |
| *Mustela nivalis* | Native | 54.165802 | 12.147532 | Open Source |
| *Mustela nivalis* | Native | 50.745255 | 11.479168 | Open Source |
| *Mustela nivalis* | Native | 52.566124 | 13.728447 | Open Source |
| *Mustela nivalis* | Native | 48.36697 | 8.823566 | Open Source |
| *Mustela nivalis* | Native | 53.12056 | 8.807109 | Open Source |
| *Mustela nivalis* | Native | 47.753174 | 9.61587 | Open Source |
| *Mustela nivalis* | Native | 51.740894 | 9.950061999 | Open Source |
| *Mustela nivalis* | Native | 53.988293 | 10.405254 | Open Source |
| *Mustela nivalis* | Native | 52.528648 | 13.580303 | Open Source |
| *Mustela nivalis* | Native | 53.373466 | 8.540668 | Open Source |
| *Mustela nivalis* | Native | 49.804989 | 7.755931 | Open Source |
| *Mustela nivalis* | Native | 53.591484 | 8.786316 | Open Source |
| *Mustela nivalis* | Native | 49.214737 | 8.742027 | Open Source |
| *Mustela nivalis* | Native | 51.066429 | 12.029171 | Open Source |
| *Mustela nivalis* | Native | 52.548069 | 13.540564 | Open Source |
| *Mustela nivalis* | Native | 50.087822 | 10.765228 | Open Source |
| *Mustela nivalis* | Native | 50.898567 | 6.147021999 | Open Source |
| *Mustela nivalis* | Native | 50.789986 | 8.87352 | Open Source |
| *Mustela nivalis* | Native | 53.796795 | 8.677364 | Open Source |
| *Mustela nivalis* | Native | 52.530426 | 13.572922 | Open Source |
| *Mustela nivalis* | Native | 48.556149 | -2.831891 | Open Source |
| *Mustela nivalis* | Native | 53.903732 | 10.782738 | Open Source |
| *Mustela nivalis* | Native | 52.963787 | 10.491085 | Open Source |
| *Mustela nivalis* | Native | 51.577221 | 9.935000001 | Open Source |
| *Mustela nivalis* | Native | 54.110565 | 10.743084 | Open Source |
| *Mustela nivalis* | Native | 53.039124 | 11.461823 | Open Source |
| *Mustela nivalis* | Native | 49.951332 | 10.309982 | Open Source |
| *Mustela nivalis* | Native | 51.540035 | 10.193167 | Open Source |
| *Mustela nivalis* | Native | 54.13353 | 10.024166 | Open Source |
| *Mustela nivalis* | Native | 51.400639 | 7.203171 | Open Source |
| *Mustela nivalis* | Native | 50.573452 | 9.658914 | Open Source |
| *Mustela nivalis* | Native | 50.72961 | 11.43651 | Open Source |
| *Mustela nivalis* | Native | 51.306259 | 9.186544 | Open Source |
| *Mustela nivalis* | Native | 48.903793 | 13.192927 | Open Source |
| *Mustela nivalis* | Native | 51.572964 | 10.140123 | Open Source |
| *Mustela nivalis* | Native | 48.923981 | 10.207129 | Open Source |
| *Mustela nivalis* | Native | 42.88016 | -2.642439 | Open Source |
| *Mustela nivalis* | Native | 59.159306 | 15.374774 | Open Source |
| *Mustela nivalis* | Native | 57.018227 | 12.340454 | Open Source |
| *Mustela nivalis* | Native | 56.221275 | 16.427914 | Open Source |
| *Mustela nivalis* | Native | 55.749394 | 12.959123 | Open Source |
| *Mustela nivalis* | Native | 57.356805 | 14.867468 | Open Source |
| *Mustela nivalis* | Native | 56.336719 | 16.054718 | Open Source |
| *Mustela nivalis* | Native | 56.705445 | 13.093511 | Open Source |
| *Mustela nivalis* | Native | 58.578088 | 14.230823 | Open Source |
| *Mustela nivalis* | Native | 57.465925 | 14.123385 | Open Source |
| *Mustela nivalis* | Native | 58.920191 | 17.856109 | Open Source |
| *Mustela nivalis* | Native | 53.457684 | 11.515326 | Open Source |
| *Mustela nivalis* | Native | 49.145279 | 10.705876 | Open Source |
| *Mustela nivalis* | Native | 54.785641 | 9.924774001 | Open Source |
| *Mustela nivalis* | Native | 50.990841 | 6.616997001 | Open Source |
| *Mustela nivalis* | Native | 50.862675 | 6.153717001 | Open Source |
| *Mustela nivalis* | Native | 47.406948 | 11.566887 | Open Source |
| *Mustela nivalis* | Native | 53.957146 | 11.948833 | Open Source |
| *Mustela nivalis* | Native | 52.521965 | 13.576655 | Open Source |
| *Mustela nivalis* | Native | 52.528648 | 13.580303 | Open Source |
| *Mustela nivalis* | Native | 50.907475 | 6.607023 | Open Source |
| *Mustela nivalis* | Native | 51.440365 | 10.953283 | Open Source |
| *Mustela nivalis* | Native | 52.649666 | 12.405088 | Open Source |
| *Mustela nivalis* | Native | 51.790649 | 8.832043 | Open Source |
| *Mustela nivalis* | Native | 49.395557 | 9.313316001 | Open Source |
| *Mustela nivalis* | Native | 51.554394 | 10.058541 | Open Source |
| *Mustela nivalis* | Native | 51.503399 | 9.017029 | Open Source |
| *Mustela nivalis* | Native | 51.023762 | 8.573842 | Open Source |
| *Mustela nivalis* | Native | 50.863834 | 6.120389001 | Open Source |
| *Mustela nivalis* | Native | 50.208328 | 6.807317999 | Open Source |
| *Mustela nivalis* | Native | 53.574772 | 8.913345001 | Open Source |
| *Mustela nivalis* | Native | 50.994202 | 6.849203 | Open Source |
| *Mustela nivalis* | Native | 50.856083 | 6.154806 | Open Source |
| *Mustela nivalis* | Native | 53.73597 | 10.150166 | Open Source |
| *Mustela nivalis* | Native | 50.337902 | 8.714991 | Open Source |
| *Mustela nivalis* | Native | 50.216557 | 9.095478 | Open Source |
| *Mustela nivalis* | Native | 50.541798 | 10.360794 | Open Source |
| *Mustela nivalis* | Native | 50.768108 | 8.962668 | Open Source |
| *Mustela nivalis* | Native | 48.270969 | 13.05665 | Open Source |
| *Mustela nivalis* | Native | 50.217339 | 10.236168 | Open Source |
| *Mustela nivalis* | Native | 49.525654 | 11.625938 | Open Source |
| *Mustela nivalis* | Native | 51.740894 | 9.950061999 | Open Source |
| *Mustela nivalis* | Native | 50.142559 | 10.72948 | Open Source |
| *Mustela nivalis* | Native | 48.160118 | 8.541104999 | Open Source |
| *Mustela nivalis* | Native | 54.176052 | 10.550008 | Open Source |
| *Mustela nivalis* | Native | 51.114326 | 6.185483 | Open Source |
| *Mustela nivalis* | Native | 54.176052 | 10.550008 | Open Source |
| *Mustela nivalis* | Native | 52.875347 | 10.406113 | Open Source |
| *Mustela nivalis* | Native | 53.503109 | 7.057257001 | Open Source |
| *Mustela nivalis* | Native | 48.17181 | 11.615295 | Open Source |
| *Mustela nivalis* | Native | 47.59948 | 10.294828 | Open Source |
| *Mustela nivalis* | Native | 51.262802 | 8.990677 | Open Source |
| *Mustela nivalis* | Native | 53.767384 | 10.085449 | Open Source |
| *Mustela nivalis* | Native | 52.535851 | 9.233797999 | Open Source |
| *Mustela nivalis* | Native | 48.810482 | 13.495411 | Open Source |
| *Mustela nivalis* | Native | 50.420853 | 8.201466001 | Open Source |
| *Mustela nivalis* | Native | 51.694832 | 10.008073 | Open Source |
| *Mustela nivalis* | Native | 50.260925 | 6.95117 | Open Source |
| *Mustela nivalis* | Native | 53.67181 | 10.086436 | Open Source |
| *Mustela nivalis* | Native | 54.176052 | 10.550008 | Open Source |
| *Mustela nivalis* | Native | 51.359669 | 9.054623 | Open Source |
| *Mustela nivalis* | Native | 49.829678 | 9.545403 | Open Source |
| *Mustela nivalis* | Native | 48.838623 | 9.425068 | Open Source |
| *Mustela nivalis* | Native | 50.222115 | 10.25651 | Open Source |
| *Mustela nivalis* | Native | 50.92326 | 8.074812 | Open Source |
| *Mustela nivalis* | Native | 53.146976 | 8.813438 | Open Source |
| *Mustela nivalis* | Native | 54.176052 | 10.550008 | Open Source |
| *Mustela nivalis* | Native | 50.964333 | 6.609026999 | Open Source |
| *Mustela nivalis* | Native | 47.118271 | 11.385956 | Open Source |
| *Mustela nivalis* | Native | 52.582577 | 12.904344 | Open Source |
| *Mustela nivalis* | Native | 53.435516 | 8.592339001 | Open Source |
| *Mustela nivalis* | Native | 49.839809 | 10.119395 | Open Source |
| *Mustela nivalis* | Native | 51.623665 | 7.457513999 | Open Source |
| *Mustela nivalis* | Native | 49.932285 | 7.990408001 | Open Source |
| *Mustela nivalis* | Native | 52.52924 | 9.475308 | Open Source |
| *Mustela nivalis* | Native | 51.155029 | 10.177159 | Open Source |
| *Mustela nivalis* | Native | 54.785641 | 9.924774001 | Open Source |
| *Mustela nivalis* | Native | 49.989422 | 10.135403 | Open Source |
| *Mustela nivalis* | Native | 48.870926 | 9.434166 | Open Source |
| *Mustela nivalis* | Native | 50.894047 | 9.588146 | Open Source |
| *Mustela nivalis* | Native | 50.721241 | 10.179005 | Open Source |
| *Mustela nivalis* | Native | 48.923977 | 9.457237 | Open Source |
| *Mustela nivalis* | Native | 50.72961 | 11.43651 | Open Source |
| *Mustela nivalis* | Native | 54.304455 | 10.563698 | Open Source |
| *Mustela nivalis* | Native | 48.492023 | 12.944847 | Open Source |
| *Mustela nivalis* | Native | 50.217339 | 10.236168 | Open Source |
| *Mustela nivalis* | Native | 50.264656 | 10.304918 | Open Source |
| *Mustela nivalis* | Native | 51.934246 | 7.200699 | Open Source |
| *Mustela nivalis* | Native | 47.288544 | 11.531267 | Open Source |
| *Mustela nivalis* | Native | 48.899174 | 9.418393999 | Open Source |
| *Mustela nivalis* | Native | 50.851223 | 6.165 | Open Source |
| *Mustela nivalis* | Native | 51.788589 | 9.923037 | Open Source |
| *Mustela nivalis* | Native | 55.839134 | 8.258286001 | Open Source |
| *Mustela nivalis* | Native | 53.84721 | 9.951124 | Open Source |
| *Mustela nivalis* | Native | 52.806496 | 12.135086 | Open Source |
| *Mustela nivalis* | Native | 54.435406 | 9.291368 | Open Source |
| *Mustela nivalis* | Native | 54.547874 | 9.660931 | Open Source |
| *Mustela nivalis* | Native | 51.572964 | 10.140123 | Open Source |
| *Mustela nivalis* | Native | 50.010387 | 10.545158 | Open Source |
| *Mustela nivalis* | Native | 49.657391 | 10.844869 | Open Source |
| *Mustela nivalis* | Native | 50.005642 | 10.55563 | Open Source |
| *Mustela nivalis* | Native | 53.533779 | 8.714560999 | Open Source |
| *Mustela nivalis* | Native | 51.591309 | 7.130384001 | Open Source |
| *Mustela nivalis* | Native | 53.151299 | 13.036767 | Open Source |
| *Mustela nivalis* | Native | 51.276306 | 6.407003 | Open Source |
| *Mustela nivalis* | Native | 49.659405 | 8.595772001 | Open Source |
| *Mustela nivalis* | Native | 52.719242 | 23.835697 | Open Source |
| *Mustela nivalis* | Native | 53.592667 | 10.884121 | Open Source |
| *Mustela nivalis* | Native | 52.446651 | 9.319469999 | Open Source |
| *Mustela nivalis* | Native | 49.984344 | 9.736032001 | Open Source |
| *Mustela nivalis* | Native | 50.482307 | 9.9967 | Open Source |
| *Mustela nivalis* | Native | 46.044285 | 22.155905 | Open Source |
| *Mustela nivalis* | Native | 49.7658 | 8.576889 | Open Source |
| *Mustela nivalis* | Native | 49.654739 | 9.855585999 | Open Source |
| *Mustela nivalis* | Native | 53.562946 | 10.87904 | Open Source |
| *Mustela nivalis* | Native | 49.834133 | 7.866662 | Open Source |
| *Mustela nivalis* | Native | 49.991451 | 8.454708999 | Open Source |
| *Mustela nivalis* | Native | 50.833725 | 6.445626999 | Open Source |
| *Mustela nivalis* | Native | 52.528648 | 13.580303 | Open Source |
| *Mustela nivalis* | Native | 48.652077 | 8.941498 | Open Source |
| *Mustela nivalis* | Native | 50.147865 | 8.77327 | Open Source |
| *Mustela nivalis* | Native | 51.359669 | 9.054623 | Open Source |
| *Mustela nivalis* | Native | 54.51281 | 11.182795 | Open Source |
| *Mustela nivalis* | Native | 50.851166 | 6.154639 | Open Source |
| *Mustela nivalis* | Native | 50.416176 | 8.227386 | Open Source |
| *Mustela nivalis* | Native | 53.129162 | 11.114044 | Open Source |
| *Mustela nivalis* | Native | 49.667961 | 8.583241 | Open Source |
| *Mustela nivalis* | Native | 53.146564 | 12.731094 | Open Source |
| *Mustela nivalis* | Native | 51.83461 | 7.281163001 | Open Source |
| *Mustela nivalis* | Native | 48.17181 | 11.615295 | Open Source |
| *Mustela nivalis* | Native | 51.434963 | 6.80603 | Open Source |
| *Mustela nivalis* | Native | 49.267357 | 8.516893 | Open Source |
| *Mustela nivalis* | Native | 54.120399 | 10.594597 | Open Source |
| *Mustela nivalis* | Native | 48.353283 | 11.721382 | Open Source |
| *Mustela nivalis* | Native | 51.064972 | 8.889313 | Open Source |
| *Mustela nivalis* | Native | 47.875999 | 10.724936 | Open Source |
| *Mustela nivalis* | Native | 53.560501 | 8.714905 | Open Source |
| *Mustela nivalis* | Native | 49.273949 | 11.290302 | Open Source |
| *Mustela nivalis* | Native | 50.01469 | 8.394499 | Open Source |
| *Mustela nivalis* | Native | 50.004044 | 10.57683 | Open Source |
| *Mustela nivalis* | Native | 50.861851 | 6.162386001 | Open Source |
| *Mustela nivalis* | Native | 50.861851 | 6.162386001 | Open Source |
| *Mustela nivalis* | Native | 47.875999 | 10.724936 | Open Source |
| *Mustela nivalis* | Native | 50.194073 | 10.310863 | Open Source |
| *Mustela nivalis* | Native | 50.194073 | 10.310863 | Open Source |
| *Mustela nivalis* | Native | 51.112576 | 9.075737 | Open Source |
| *Mustela nivalis* | Native | 47.630535 | 12.202528 | Open Source |
| *Mustela nivalis* | Native | 47.549263 | 12.121718 | Open Source |
| *Mustela nivalis* | Native | 52.873016 | 8.830809 | Open Source |
| *Mustela nivalis* | Native | 53.340099 | 8.748206999 | Open Source |
| *Mustela nivalis* | Native | 53.904415 | 10.721283 | Open Source |
| *Mustela nivalis* | Native | 51.442238 | 10.878696 | Open Source |
| *Mustela nivalis* | Native | 50.183495 | 10.275221 | Open Source |
| *Mustela nivalis* | Native | 54.304455 | 10.563698 | Open Source |
| *Mustela nivalis* | Native | 53.28569 | 13.879166 | Open Source |
| *Mustela nivalis* | Native | 51.373692 | 7.540835001 | Open Source |
| *Mustela nivalis* | Native | 50.861012 | 6.432837999 | Open Source |
| *Mustela nivalis* | Native | 50.307217 | 8.582637001 | Open Source |
| *Mustela nivalis* | Native | 49.164196 | 7.540912999 | Open Source |
| *Mustela nivalis* | Native | 49.666904 | 9.72672 | Open Source |
| *Mustela nivalis* | Native | 50.776661 | 8.955145 | Open Source |
| *Mustela nivalis* | Native | 50.298458 | 8.581593 | Open Source |
| *Mustela nivalis* | Native | 50.188469 | 10.250845 | Open Source |
| *Mustela nivalis* | Native | 52.873528 | 10.123759 | Open Source |
| *Mustela nivalis* | Native | 50.794678 | 8.839746 | Open Source |
| *Mustela nivalis* | Native | 51.373699 | 9.790496 | Open Source |
| *Mustela nivalis* | Native | 51.924561 | 10.494473 | Open Source |
| *Mustela nivalis* | Native | 49.858318 | 8.358905001 | Open Source |
| *Mustela nivalis* | Native | 51.163448 | 9.108791999 | Open Source |
| *Mustela nivalis* | Native | 53.373474 | 10.290392 | Open Source |
| *Mustela nivalis* | Native | 50.966171 | 6.879221999 | Open Source |
| *Mustela nivalis* | Native | 47.54945 | 10.310497 | Open Source |
| *Mustela nivalis* | Native | 50.855446 | 6.094278 | Open Source |
| *Mustela nivalis* | Native | 50.039722 | 10.22975 | Open Source |
| *Mustela nivalis* | Native | 50.861851 | 6.162386001 | Open Source |
| *Mustela nivalis* | Native | 50.604107 | 8.633709001 | Open Source |
| *Mustela nivalis* | Native | 51.064972 | 8.889313 | Open Source |
| *Mustela nivalis* | Native | 52.528648 | 13.580303 | Open Source |
| *Mustela nivalis* | Native | 51.66766 | 6.450713 | Open Source |
| *Mustela nivalis* | Native | 50.194073 | 10.310863 | Open Source |
| *Mustela nivalis* | Native | 51.740894 | 9.950061999 | Open Source |
| *Mustela nivalis* | Native | 48.707447 | 12.760706 | Open Source |
| *Mustela nivalis* | Native | 54.439102 | 12.802334 | Open Source |
| *Mustela nivalis* | Native | 53.71183 | 11.371928 | Open Source |
| *Mustela nivalis* | Native | 52.530426 | 13.572922 | Open Source |
| *Mustela nivalis* | Native | 50.869137 | 6.425628999 | Open Source |
| *Mustela nivalis* | Native | 54.176052 | 10.550008 | Open Source |
| *Mustela nivalis* | Native | 47.720158 | 10.322342 | Open Source |
| *Mustela nivalis* | Native | 52.479435 | 9.346619 | Open Source |
| *Mustela nivalis* | Native | 47.421001 | 11.630917 | Open Source |
| *Mustela nivalis* | Native | 49.260662 | 8.520411999 | Open Source |
| *Mustela nivalis* | Native | 50.344036 | 9.939537 | Open Source |
| *Mustela nivalis* | Native | 51.572964 | 10.140123 | Open Source |
| *Mustela nivalis* | Native | 50.901085 | 9.594498 | Open Source |
| *Mustela nivalis* | Native | 51.485409 | 11.889178 | Open Source |
| *Mustela nivalis* | Native | 48.100899 | 8.945961 | Open Source |
| *Mustela nivalis* | Native | 52.485138 | 10.337373 | Open Source |
| *Mustela nivalis* | Native | 49.657391 | 10.844869 | Open Source |
| *Mustela nivalis* | Native | 51.591309 | 7.130384001 | Open Source |
| *Mustela nivalis* | Native | 49.964638 | 10.183897 | Open Source |
| *Mustela nivalis* | Native | 54.785641 | 9.924774001 | Open Source |
| *Mustela nivalis* | Native | 51.073742 | 11.540237 | Open Source |
| *Mustela nivalis* | Native | 53.764542 | 8.53775 | Open Source |
| *Mustela nivalis* | Native | 50.152092 | 8.653277999 | Open Source |
| *Mustela nivalis* | Native | 50.89502 | 7.970924 | Open Source |
| *Mustela nivalis* | Native | 50.086334 | 12.242546 | Open Source |
| *Mustela nivalis* | Native | 52.508255 | 13.575025 | Open Source |
| *Mustela nivalis* | Native | 49.195557 | 7.184722 | Open Source |
| *Mustela nivalis* | Native | 47.875999 | 10.724936 | Open Source |
| *Mustela nivalis* | Native | 49.25016 | 8.513546 | Open Source |
| *Mustela nivalis* | Native | 54.1082 | 10.669037 | Open Source |
| *Mustela nivalis* | Native | 53.724442 | 10.178318 | Open Source |
| *Mustela nivalis* | Native | 50.358276 | 7.293548999 | Open Source |
| *Mustela nivalis* | Native | 51.162712 | 9.119468 | Open Source |
| *Mustela nivalis* | Native | 50.191956 | 10.18364 | Open Source |
| *Mustela nivalis* | Native | 50.151196 | 8.772411 | Open Source |
| *Mustela nivalis* | Native | 50.778721 | 9.108103001 | Open Source |
| *Mustela nivalis* | Native | 50.298458 | 8.581593 | Open Source |
| *Mustela nivalis* | Native | 50.797054 | 6.254944 | Open Source |
| *Mustela nivalis* | Native | 51.155125 | 10.977313 | Open Source |
| *Mustela nivalis* | Native | 50.775002 | 8.973611 | Open Source |
| *Mustela nivalis* | Native | 51.362106 | 8.805628 | Open Source |
| *Mustela nivalis* | Native | 53.489777 | 9.553168 | Open Source |
| *Mustela nivalis* | Native | 50.020027 | 10.280945 | Open Source |
| *Mustela nivalis* | Native | 50.861012 | 6.432837999 | Open Source |
| *Mustela nivalis* | Native | 54.877003 | 8.429947 | Open Source |
| *Mustela nivalis* | Native | 52.52343 | 10.984612 | Open Source |
| *Mustela nivalis* | Native | 54.176052 | 10.550008 | Open Source |
| *Mustela nivalis* | Native | 46.570663 | 11.81571 | Open Source |
| *Mustela nivalis* | Native | 53.157734 | 10.457096 | Open Source |
| *Mustela nivalis* | Native | 51.226238 | 9.090157 | Open Source |
| *Mustela nivalis* | Native | 53.705242 | 10.139008 | Open Source |
| *Mustela nivalis* | Native | 51.624939 | 7.218476 | Open Source |
| *Mustela nivalis* | Native | 51.718147 | 10.531769 | Open Source |
| *Mustela nivalis* | Native | 50.742191 | 10.226242 | Open Source |
| *Mustela nivalis* | Native | 52.273594 | 9.707165001 | Open Source |
| *Mustela nivalis* | Native | 52.025566 | 7.652320999 | Open Source |
| *Mustela nivalis* | Native | 49.922119 | 8.914117999 | Open Source |
| *Mustela nivalis* | Native | 50.301735 | 8.568089001 | Open Source |
| *Mustela nivalis* | Native | 51.066429 | 12.029171 | Open Source |
| *Mustela nivalis* | Native | 51.739769 | 9.948685001 | Open Source |
| *Mustela nivalis* | Native | 51.740894 | 9.950061999 | Open Source |
| *Mustela nivalis* | Native | 59.763346 | 16.0418 | Open Source |
| *Mustela nivalis* | Native | 58.358725 | 15.624155 | Open Source |
| *Mustela nivalis* | Native | 60.482444 | 16.00867 | Open Source |
| *Mustela nivalis* | Native | 57.414572 | 14.106074 | Open Source |
| *Mustela nivalis* | Native | 57.020731 | 15.386497 | Open Source |
| *Mustela nivalis* | Native | 57.020731 | 15.386497 | Open Source |
| *Mustela nivalis* | Native | 61.573714 | 12.909509 | Open Source |
| *Mustela nivalis* | Native | 61.352722 | 15.051659 | Open Source |
| *Mustela nivalis* | Native | 61.422803 | 14.538622 | Open Source |
| *Mustela nivalis* | Native | 61.122333 | 14.821609 | Open Source |
| *Mustela nivalis* | Native | 61.094013 | 14.657263 | Open Source |
| *Mustela nivalis* | Native | 61.094013 | 14.657263 | Open Source |
| *Mustela nivalis* | Native | 60.770807 | 14.251188 | Open Source |
| *Mustela nivalis* | Native | 60.455585 | 13.351579 | Open Source |
| *Mustela nivalis* | Native | 58.963116 | 17.16279 | Open Source |
| *Mustela nivalis* | Native | 56.606429 | 14.169819 | Open Source |
| *Mustela nivalis* | Native | 57.958168 | 13.530694 | Open Source |
| *Mustela nivalis* | Native | 56.393303 | 12.77805 | Open Source |
| *Mustela nivalis* | Native | 55.425379 | 13.849363 | Open Source |
| *Mustela nivalis* | Native | 57.282292 | 15.282598 | Open Source |
| *Mustela nivalis* | Native | 42.573864 | -7.093272001 | Open Source |
| *Mustela nivalis* | Native | 42.573864 | -7.093272001 | Open Source |
| *Mustela nivalis* | Native | 58.101762 | 15.757654 | Open Source |
| *Mustela nivalis* | Native | 58.830471 | 12.546527 | Open Source |
| *Mustela nivalis* | Native | 58.857088 | 16.701873 | Open Source |
| *Mustela nivalis* | Native | 59.906475 | 17.55874 | Open Source |
| *Mustela nivalis* | Native | 60.045011 | 17.738214 | Open Source |
| *Mustela nivalis* | Native | 59.352276 | 14.075999 | Open Source |
| *Mustela nivalis* | Native | 55.511589 | 12.930719 | Open Source |
| *Mustela nivalis* | Native | 58.695496 | 16.98592 | Open Source |
| *Mustela nivalis* | Native | 57.912085 | 15.768928 | Open Source |
| *Mustela nivalis* | Native | 56.035962 | 13.242497 | Open Source |
| *Mustela nivalis* | Native | 59.800542 | 16.211653 | Open Source |
| *Mustela nivalis* | Native | 59.762663 | 16.211555 | Open Source |
| *Mustela nivalis* | Native | 59.751325 | 16.191136 | Open Source |
| *Mustela nivalis* | Native | 56.83198 | 15.335257 | Open Source |
| *Mustela nivalis* | Native | 57.126704 | 15.414246 | Open Source |
| *Mustela nivalis* | Native | 58.275606 | 15.742131 | Open Source |
| *Mustela nivalis* | Native | 58.383942 | 15.621936 | Open Source |
| *Mustela nivalis* | Native | 58.963074 | 16.629289 | Open Source |
| *Mustela nivalis* | Native | 57.31151 | 15.291311 | Open Source |
| *Mustela nivalis* | Native | 57.126704 | 15.414246 | Open Source |
| *Mustela nivalis* | Native | 57.911995 | 15.769098 | Open Source |
| *Mustela nivalis* | Native | 57.682186 | 11.952561 | Open Source |
| *Mustela nivalis* | Native | 55.577822 | 13.088301 | Open Source |
| *Mustela nivalis* | Native | 57.869584 | 14.2782 | Open Source |
| *Mustela nivalis* | Native | 58.148388 | 15.746946 | Open Source |
| *Mustela nivalis* | Native | 59.831539 | 17.661274 | Open Source |
| *Mustela nivalis* | Native | 59.716226 | 19.028222 | Open Source |
| *Mustela nivalis* | Native | 56.105683 | 13.251201 | Open Source |
| *Mustela nivalis* | Native | 57.725978 | 11.911409 | Open Source |
| *Mustela nivalis* | Native | 59.043159 | 15.313554 | Open Source |
| *Mustela nivalis* | Native | 57.536609 | 13.920276 | Open Source |
| *Mustela nivalis* | Native | 56.295238 | 16.500381 | Open Source |
| *Mustela nivalis* | Native | 58.81738 | 15.36315 | Open Source |
| *Mustela nivalis* | Native | 59.518246 | 14.982309 | Open Source |
| *Mustela nivalis* | Native | 57.442617 | 14.126161 | Open Source |
| *Mustela nivalis* | Native | 59.742178 | 13.396459 | Open Source |
| *Mustela nivalis* | Native | 58.935848 | 17.949974 | Open Source |
| *Mustela nivalis* | Native | 59.413322 | 15.668146 | Open Source |
| *Mustela nivalis* | Native | 57.415458 | 15.114954 | Open Source |
| *Mustela nivalis* | Native | 58.054039 | 14.874008 | Open Source |
| *Mustela nivalis* | Native | 56.940593 | 13.526661 | Open Source |
| *Mustela nivalis* | Native | 59.803077 | 16.073618 | Open Source |
| *Mustela nivalis* | Native | 57.755293 | 13.424334 | Open Source |
| *Mustela nivalis* | Native | 59.798888 | 16.223394 | Open Source |
| *Mustela nivalis* | Native | 59.964853 | 17.838844 | Open Source |
| *Mustela nivalis* | Native | 58.362565 | 11.356035 | Open Source |
| *Mustela nivalis* | Native | 55.403585 | 12.866875 | Open Source |
| *Mustela nivalis* | Native | 56.678955 | 12.715967 | Open Source |
| *Mustela nivalis* | Native | 59.68573 | 17.963871 | Open Source |
| *Mustela nivalis* | Native | 59.751325 | 16.191136 | Open Source |
| *Mustela nivalis* | Native | 56.139558 | 13.445588 | Open Source |
| *Mustela nivalis* | Native | 55.692733 | 13.886616 | Open Source |
| *Mustela nivalis* | Native | 59.762663 | 16.211555 | Open Source |
| *Mustela nivalis* | Native | 59.856436 | 16.158446 | Open Source |
| *Mustela nivalis* | Native | 58.284174 | 13.096772 | Open Source |
| *Mustela nivalis* | Native | 59.698722 | 16.039931 | Open Source |
| *Mustela nivalis* | Native | 59.045543 | 16.996502 | Open Source |
| *Mustela nivalis* | Native | 57.853771 | 14.081442 | Open Source |
| *Mustela nivalis* | Native | 59.414616 | 18.459741 | Open Source |
| *Mustela nivalis* | Native | 58.467746 | 15.047122 | Open Source |
| *Mustela nivalis* | Native | 57.76483 | 12.037757 | Open Source |
| *Mustela nivalis* | Native | 59.811548 | 16.161536 | Open Source |
| *Mustela nivalis* | Native | 59.83009 | 16.206129 | Open Source |
| *Mustela nivalis* | Native | 59.803804 | 16.001115 | Open Source |
| *Mustela nivalis* | Native | 59.819971 | 16.19799 | Open Source |
| *Mustela nivalis* | Native | 59.807916 | 16.207288 | Open Source |
| *Mustela nivalis* | Native | 57.701502 | 16.665267 | Open Source |
| *Mustela nivalis* | Native | 59.811999 | 16.221418 | Open Source |
| *Mustela nivalis* | Native | 60.53712 | 15.387575 | Open Source |
| *Mustela nivalis* | Native | 59.83009 | 16.206129 | Open Source |
| *Mustela nivalis* | Native | 59.685911 | 14.720755 | Open Source |
| *Mustela nivalis* | Native | 56.780404 | 14.587957 | Open Source |
| *Mustela nivalis* | Native | 56.819745 | 16.845442 | Open Source |
| *Mustela nivalis* | Native | 56.712126 | 12.887896 | Open Source |
| *Mustela nivalis* | Native | 56.339692 | 16.501028 | Open Source |
| *Mustela nivalis* | Native | 56.118761 | 13.081465 | Open Source |
| *Mustela nivalis* | Native | 56.302076 | 13.946165 | Open Source |
| *Mustela nivalis* | Native | 56.036404 | 13.246638 | Open Source |
| *Mustela nivalis* | Native | 56.202696 | 15.766583 | Open Source |
| *Mustela nivalis* | Native | 56.639007 | 14.669938 | Open Source |
| *Mustela nivalis* | Native | 58.324429 | 14.719956 | Open Source |
| *Mustela nivalis* | Native | 59.206638 | 18.037848 | Open Source |
| *Mustela nivalis* | Native | 57.076545 | 13.882502 | Open Source |
| *Mustela nivalis* | Native | 57.514533 | 16.003774 | Open Source |
| *Mustela nivalis* | Native | 58.678885 | 13.628063 | Open Source |
| *Mustela nivalis* | Native | 59.811999 | 16.221418 | Open Source |
| *Mustela nivalis* | Native | 59.793962 | 15.977903 | Open Source |
| *Mustela nivalis* | Native | 57.781774 | 14.212255 | Open Source |
| *Mustela nivalis* | Native | 55.55844 | 13.858777 | Open Source |
| *Mustela nivalis* | Native | 55.690084 | 13.49327 | Open Source |
| *Mustela nivalis* | Native | 56.385014 | 12.661592 | Open Source |
| *Mustela nivalis* | Native | 59.258304 | 15.144266 | Open Source |
| *Mustela nivalis* | Native | 58.936228 | 17.943228 | Open Source |
| *Mustela nivalis* | Native | 56.357393 | 14.804469 | Open Source |
| *Mustela nivalis* | Native | 56.310812 | 16.407271 | Open Source |
| *Mustela nivalis* | Native | 57.784508 | 13.411147 | Open Source |
| *Mustela nivalis* | Native | 56.908849 | 12.400012 | Open Source |
| *Mustela nivalis* | Native | 58.401014 | 14.220367 | Open Source |
| *Mustela nivalis* | Native | 58.517721 | 11.98216 | Open Source |
| *Mustela nivalis* | Native | 58.649865 | 15.270192 | Open Source |
| *Mustela nivalis* | Native | 56.365863 | 14.001636 | Open Source |
| *Mustela nivalis* | Native | 57.417567 | 15.106258 | Open Source |
| *Mustela nivalis* | Native | 57.488064 | 11.929501 | Open Source |
| *Mustela nivalis* | Native | 58.847772 | 11.512916 | Open Source |
| *Mustela nivalis* | Native | 57.126704 | 15.414246 | Open Source |
| *Mustela nivalis* | Native | 59.278819 | 14.275523 | Open Source |
| *Mustela nivalis* | Native | 59.851866 | 16.031011 | Open Source |
| *Mustela nivalis* | Native | 59.882013 | 17.354014 | Open Source |
| *Mustela nivalis* | Native | 59.442609 | 17.794368 | Open Source |
| *Mustela nivalis* | Native | 59.224013 | 18.082566 | Open Source |
| *Mustela nivalis* | Native | 59.360692 | 17.041454 | Open Source |
| *Mustela nivalis* | Native | 56.561172 | 16.448476 | Open Source |
| *Mustela nivalis* | Native | 58.585777 | 14.006694 | Open Source |
| *Mustela nivalis* | Native | 56.66939 | 16.6034 | Open Source |
| *Mustela nivalis* | Native | 58.492726 | 15.510761 | Open Source |
| *Mustela nivalis* | Native | 59.705846 | 16.069818 | Open Source |
| *Mustela nivalis* | Native | 58.906322 | 17.904933 | Open Source |
| *Mustela nivalis* | Native | 55.384106 | 13.466986 | Open Source |
| *Mustela nivalis* | Native | 58.363677 | 13.869 | Open Source |
| *Mustela nivalis* | Native | 58.946428 | 17.912748 | Open Source |
| *Mustela nivalis* | Native | 58.737054 | 14.504387 | Open Source |
| *Mustela nivalis* | Native | 56.353969 | 13.956415 | Open Source |
| *Mustela nivalis* | Native | 57.708274 | 11.788753 | Open Source |
| *Mustela nivalis* | Native | 57.488938 | 14.111356 | Open Source |
| *Mustela nivalis* | Native | 56.692602 | 12.883036 | Open Source |
| *Mustela nivalis* | Native | 59.558878 | 18.241235 | Open Source |
| *Mustela nivalis* | Native | 59.598219 | 18.562607 | Open Source |
| *Mustela nivalis* | Native | 61.276341 | 17.018098 | Open Source |
| *Mustela nivalis* | Native | 55.662276 | 13.568181 | Open Source |
| *Mustela nivalis* | Native | 57.465954 | 13.257993 | Open Source |
| *Mustela nivalis* | Native | 56.288403 | 15.530506 | Open Source |
| *Mustela nivalis* | Native | 59.624562 | 15.047221 | Open Source |
| *Mustela nivalis* | Native | 56.424217 | 16.412301 | Open Source |
| *Mustela nivalis* | Native | 57.166543 | 17.036648 | Open Source |
| *Mustela nivalis* | Native | 56.299953 | 10.151149 | Open Source |
| *Mustela nivalis* | Native | 56.214027 | 10.161377 | Open Source |
| *Mustela nivalis* | Native | 56.244207 | 10.133929 | Open Source |
| *Mustela nivalis* | Native | 56.207426 | 10.251854 | Open Source |
| *Mustela nivalis* | Native | 55.970122 | 9.802504 | Open Source |
| *Mustela nivalis* | Native | 55.977439 | 9.782627 | Open Source |
| *Mustela nivalis* | Native | 56.004013 | 9.715267 | Open Source |
| *Mustela nivalis* | Native | 55.956004 | 9.840913001 | Open Source |
| *Mustela nivalis* | Native | 55.861008 | 9.667198001 | Open Source |
| *Mustela nivalis* | Native | 55.770184 | 12.470653 | Open Source |
| *Mustela nivalis* | Native | 52.587278 | 4.722527999 | Open Source |
| *Mustela nivalis* | Native | 51.749576 | 3.828152 | Open Source |
| *Mustela nivalis* | Native | 52.955109 | 5.615779 | Open Source |
| *Mustela nivalis* | Native | 52.955691 | 5.616645 | Open Source |
| *Mustela nivalis* | Native | 52.949773 | 5.632668 | Open Source |
| *Mustela nivalis* | Native | 51.697464 | 3.806209 | Open Source |
| *Mustela nivalis* | Native | 51.619868 | 3.985726999 | Open Source |
| *Mustela putorius* | Native | 36.784599 | -2.5959 | Open Source |
| *Mustela putorius* | Native | 37.0642525 | -3.5530686 | Open Source |
| *Mustela putorius* | Native | 37.1107768 | -2.759285 | Open Source |
| *Mustela putorius* | Native | 37.1425982 | -4.732275 | Open Source |
| *Mustela putorius* | Native | 37.1437613 | -5.1105309 | Open Source |
| *Mustela putorius* | Native | 37.1665922 | -6.8908739 | Open Source |
| *Mustela putorius* | Native | 37.1817919 | -4.0461874 | Open Source |
| *Mustela putorius* | Native | 37.1818603 | -4.047517799 | Open Source |
| *Mustela putorius* | Native | 37.2557463 | -5.5805397 | Open Source |
| *Mustela putorius* | Native | 37.2611431 | -4.7939014 | Open Source |
| *Mustela putorius* | Native | 37.3178201 | -7.0812249 | Open Source |
| *Mustela putorius* | Native | 37.5038703 | -4.4490337 | Open Source |
| *Mustela putorius* | Native | 37.5115641 | -7.3753023 | Open Source |
| *Mustela putorius* | Native | 37.622 | -7.741 | Open Source |
| *Mustela putorius* | Native | 37.7902011 | -3.601445 | Open Source |
| *Mustela putorius* | Native | 37.8061221 | -4.031467399 | Open Source |
| *Mustela putorius* | Native | 37.8171071 | -4.0405655 | Open Source |
| *Mustela putorius* | Native | 37.823616 | -4.066314701 | Open Source |
| *Mustela putorius* | Native | 37.828582 | -3.442389999 | Open Source |
| *Mustela putorius* | Native | 37.8352085 | -3.6064339 | Open Source |
| *Mustela putorius* | Native | 37.851 | -8.088999999 | Open Source |
| *Mustela putorius* | Native | 37.8520602 | -4.8367524 | Open Source |
| *Mustela putorius* | Native | 37.9140029 | -3.5583258 | Open Source |
| *Mustela putorius* | Native | 37.9172532 | -3.6970711 | Open Source |
| *Mustela putorius* | Native | 37.9280862 | -3.4351158 | Open Source |
| *Mustela putorius* | Native | 37.9441975 | -3.5629177 | Open Source |
| *Mustela putorius* | Native | 37.952 | -2.823 | Open Source |
| *Mustela putorius* | Native | 37.9799275 | -3.3805275 | Open Source |
| *Mustela putorius* | Native | 38.0047523 | -4.2078495 | Open Source |
| *Mustela putorius* | Native | 38.0307519 | -3.3423758 | Open Source |
| *Mustela putorius* | Native | 38.0318674 | -3.3139658 | Open Source |
| *Mustela putorius* | Native | 38.0424132 | -3.5248089 | Open Source |
| *Mustela putorius* | Native | 38.0433595 | -3.2540131 | Open Source |
| *Mustela putorius* | Native | 38.0553905 | -3.535537699 | Open Source |
| *Mustela putorius* | Native | 38.0704265 | -3.8613081 | Open Source |
| *Mustela putorius* | Native | 38.0774535 | -3.6472893 | Open Source |
| *Mustela putorius* | Native | 38.1127472 | -3.7510157 | Open Source |
| *Mustela putorius* | Native | 38.174 | -7.056 | Open Source |
| *Mustela putorius* | Native | 38.2122881 | -3.462925 | Open Source |
| *Mustela putorius* | Native | 38.264 | -8.335 | Open Source |
| *Mustela putorius* | Native | 38.522 | -9.144000001 | Open Source |
| *Mustela putorius* | Native | 38.533 | -9.125000001 | Open Source |
| *Mustela putorius* | Native | 38.543 | -8.484 | Open Source |
| *Mustela putorius* | Native | 38.605 | -9.049 | Open Source |
| *Mustela putorius* | Native | 38.764 | -9.252 | Open Source |
| *Mustela putorius* | Native | 38.825 | -7.77 | Open Source |
| *Mustela putorius* | Native | 38.8714561 | -3.7180138 | Open Source |
| *Mustela putorius* | Native | 38.963 | -9.303 | Open Source |
| *Mustela putorius* | Native | 38.963 | -9.303 | Open Source |
| *Mustela putorius* | Native | 39.0115479 | -1.8461323 | Open Source |
| *Mustela putorius* | Native | 39.029 | -8.367 | Open Source |
| *Mustela putorius* | Native | 39.0437528 | -2.2291088 | Open Source |
| *Mustela putorius* | Native | 39.0443527 | -2.2425842 | Open Source |
| *Mustela putorius* | Native | 39.1223421 | -3.1146122 | Open Source |
| *Mustela putorius* | Native | 39.15 | -8.996 | Open Source |
| *Mustela putorius* | Native | 39.195 | -9.003 | Open Source |
| *Mustela putorius* | Native | 39.354 | -8.445 | Open Source |
| *Mustela putorius* | Native | 39.395 | -8.941 | Open Source |
| *Mustela putorius* | Native | 39.4621738 | -1.911621101 | Open Source |
| *Mustela putorius* | Native | 39.551 | -8.39 | Open Source |
| *Mustela putorius* | Native | 39.9948105 | -4.036016499 | Open Source |
| *Mustela putorius* | Native | 40.0257742 | -3.1280136 | Open Source |
| *Mustela putorius* | Native | 40.1404832 | -4.191134 | Open Source |
| *Mustela putorius* | Native | 40.3114561 | -3.6184072 | Open Source |
| *Mustela putorius* | Native | 40.3965028 | -3.7834167 | Open Source |
| *Mustela putorius* | Native | 40.3990521 | -3.788566599 | Open Source |
| *Mustela putorius* | Native | 40.4232325 | -4.0025425 | Open Source |
| *Mustela putorius* | Native | 40.5028032 | -4.0527937 | Open Source |
| *Mustela putorius* | Native | 40.52512458 | -3.380225646 | Open Source |
| *Mustela putorius* | Native | 40.53520567 | -3.380199973 | Open Source |
| *Mustela putorius* | Native | 40.5484305 | -3.492399854 | Open Source |
| *Mustela putorius* | Native | 40.55204742 | -3.493489312 | Open Source |
| *Mustela putorius* | Native | 40.55635758 | -3.523309499 | Open Source |
| *Mustela putorius* | Native | 40.57705558 | -3.423219412 | Open Source |
| *Mustela putorius* | Native | 40.5816277 | -3.4153533 | Open Source |
| *Mustela putorius* | Native | 40.58748226 | -3.414824181 | Open Source |
| *Mustela putorius* | Native | 40.59887617 | -3.359026254 | Open Source |
| *Mustela putorius* | Native | 40.62031428 | -3.508509666 | Open Source |
| *Mustela putorius* | Native | 40.62822004 | -3.340465701 | Open Source |
| *Mustela putorius* | Native | 40.63021523 | -3.339021418 | Open Source |
| *Mustela putorius* | Native | 40.7167532 | -5.2958393 | Open Source |
| *Mustela putorius* | Native | 40.72906135 | -3.509845779 | Open Source |
| *Mustela putorius* | Native | 40.8206297 | -2.6942682 | Open Source |
| *Mustela putorius* | Native | 40.9845147 | -5.6823349 | Open Source |
| *Mustela putorius* | Native | 41.0563627 | -5.8161449 | Open Source |
| *Mustela putorius* | Native | 41.26684 | -6.0786581 | Open Source |
| *Mustela putorius* | Native | 41.3292599 | -4.6918488 | Open Source |
| *Mustela putorius* | Native | 41.5904117 | -4.8180199 | Open Source |
| *Mustela putorius* | Native | 41.6022864 | -5.6674862 | Open Source |
| *Mustela putorius* | Native | 41.6488014 | -4.734764099 | Open Source |
| *Mustela putorius* | Native | 41.8624019 | 2.0750427 | Open Source |
| *Mustela putorius* | Native | 42.1182167 | -1.6767883 | Open Source |
| *Mustela putorius* | Native | 42.1687473 | 0.2354336 | Open Source |
| *Mustela putorius* | Native | 42.308952 | 2.704353 | Open Source |
| *Mustela putorius* | Native | 42.815503 | -8.572888 | Field Data |
| *Mustela putorius* | Native | 42.84854 | -8.908819 | Field Data |
| *Mustela putorius* | Native | 42.870189 | -8.547342 | Field Data |
| *Mustela putorius* | Native | 42.883297 | -8.327259 | Field Data |
| *Mustela putorius* | Native | 42.885274 | -8.157802999 | Field Data |
| *Mustela putorius* | Native | 42.902423 | -8.466788 | Field Data |
| *Mustela putorius* | Native | 42.91696 | -8.347425001 | Field Data |
| *Mustela putorius* | Native | 42.920033 | -8.09147 | Field Data |
| *Mustela putorius* | Native | 42.926325 | -8.135579 | Field Data |
| *Mustela putorius* | Native | 42.928903 | -8.099637 | Field Data |
| *Mustela putorius* | Native | 42.931392 | -8.117443999 | Field Data |
| *Mustela putorius* | Native | 42.93509 | -8.466425 | Field Data |
| *Mustela putorius* | Native | 42.9353123 | -2.2470474 | Open Source |
| *Mustela putorius* | Native | 42.964714 | -2.627061001 | Open Source |
| *Mustela putorius* | Native | 42.9805238 | -2.8308249 | Open Source |
| *Mustela putorius* | Native | 42.981607 | -1.3345814 | Open Source |
| *Mustela putorius* | Native | 43.003511 | -8.419909001 | Field Data |
| *Mustela putorius* | Native | 43.021477 | -7.219502 | Field Data |
| *Mustela putorius* | Native | 43.0243531 | -2.381801599 | Open Source |
| *Mustela putorius* | Native | 43.0431745 | -2.250308999 | Open Source |
| *Mustela putorius* | Native | 43.050916 | -8.401130001 | Field Data |
| *Mustela putorius* | Native | 43.075952 | -8.250249 | Field Data |
| *Mustela putorius* | Native | 43.080305 | -8.35743 | Field Data |
| *Mustela putorius* | Native | 43.103159 | -5.022296999 | Open Source |
| *Mustela putorius* | Native | 43.1205326 | -2.3428345 | Open Source |
| *Mustela putorius* | Native | 43.149206 | -7.321722 | Field Data |
| *Mustela putorius* | Native | 43.2039246 | -2.3698711 | Open Source |
| *Mustela putorius* | Native | 43.2226595 | -5.6534529 | Open Source |
| *Mustela putorius* | Native | 43.242884 | -7.325582 | Field Data |
| *Mustela putorius* | Native | 43.2469846 | -2.259922 | Open Source |
| *Mustela putorius* | Native | 43.259737 | -8.212095 | Field Data |
| *Mustela putorius* | Native | 43.2680188 | -2.2321129 | Open Source |
| *Mustela putorius* | Native | 43.322049 | -7.329699 | Field Data |
| *Mustela putorius* | Native | 43.359306 | -7.99577 | Field Data |
| *Mustela putorius* | Native | 43.3683704 | -4.4479179 | Open Source |
| *Mustela putorius* | Native | 43.4346599 | -5.4344773 | Open Source |
| *Mustela putorius* | Native | 58.385435 | 8.660981001 | Open Source |
| *Mustela putorius* | Native | 60.018752 | 13.133423 | Open Source |
| *Mustela putorius* | Native | 50.561939 | -3.714592 | Open Source |
| *Mustela putorius* | Native | 45.334701 | 3.929834 | Open Source |
| *Mustela putorius* | Native | 57.632 | -7.489529999 | Open Source |
| *Mustela putorius* | Native | 57.604653 | 15.00465 | Open Source |
| *Mustela putorius* | Native | 56.182873 | 13.849084 | Open Source |
| *Mustela putorius* | Native | 50.92691 | 4.32124 | Open Source |
| *Mustela putorius* | Native | 50.204098 | 10.223207 | Open Source |
| *Mustela putorius* | Native | 58.311724 | 12.048169 | Open Source |
| *Mustela putorius* | Native | 58.164986 | 11.786642 | Open Source |
| *Mustela putorius* | Native | 52.78958 | -4.7383 | Open Source |
| *Mustela putorius* | Native | 52.92103 | -3.38098 | Open Source |
| *Mustela putorius* | Native | 52.92017 | -3.924250001 | Open Source |
| *Mustela putorius* | Native | 52.88027 | -3.48073 | Open Source |
| *Mustela putorius* | Native | 52.89454 | -3.49014 | Open Source |
| *Mustela putorius* | Native | 56.55251 | -6.126750001 | Open Source |
| *Mustela putorius* | Native | 52.10773 | -1.8481 | Open Source |
| *Mustela putorius* | Native | 52.89109 | -2.887709999 | Open Source |
| *Mustela putorius* | Native | 52.83252 | -3.07238 | Open Source |
| *Mustela putorius* | Native | 52.771 | -2.418680001 | Open Source |
| *Mustela putorius* | Native | 52.75736 | -2.473299999 | Open Source |
| *Mustela putorius* | Native | 52.76963 | -2.35723 | Open Source |
| *Mustela putorius* | Native | 52.74805 | -2.7449 | Open Source |
| *Mustela putorius* | Native | 52.83377 | -3.03233 | Open Source |
| *Mustela putorius* | Native | 52.43879 | -2.84654 | Open Source |
| *Mustela putorius* | Native | 52.88322 | -3.03202 | Open Source |
| *Mustela putorius* | Native | 52.56779 | -2.782640001 | Open Source |
| *Mustela putorius* | Native | 52.46305 | -3.01341 | Open Source |
| *Mustela putorius* | Native | 52.48046 | -3.03288 | Open Source |
| *Mustela putorius* | Native | 52.82338 | -2.814159999 | Open Source |
| *Mustela putorius* | Native | 52.64406 | -2.4692 | Open Source |
| *Mustela putorius* | Native | 52.57035 | -2.80186 | Open Source |
| *Mustela putorius* | Native | 52.94264 | -2.544589999 | Open Source |
| *Mustela putorius* | Native | 52.70349 | -2.942069999 | Open Source |
| *Mustela putorius* | Native | 52.72741 | -2.840280001 | Open Source |
| *Mustela putorius* | Native | 52.8404 | -2.879579999 | Open Source |
| *Mustela putorius* | Native | 52.8351 | -2.98189 | Open Source |
| *Mustela putorius* | Native | 52.50407 | -2.40141 | Open Source |
| *Mustela putorius* | Native | 52.47888 | -2.613199999 | Open Source |
| *Mustela putorius* | Native | 52.69951 | -2.96257 | Open Source |
| *Mustela putorius* | Native | 52.46201 | -2.33774 | Open Source |
| *Mustela putorius* | Native | 52.78884 | -2.45741 | Open Source |
| *Mustela putorius* | Native | 52.43782 | -2.862340001 | Open Source |
| *Mustela putorius* | Native | 52.65925 | -2.49154 | Open Source |
| *Mustela putorius* | Native | 52.65925 | -2.49154 | Open Source |
| *Mustela putorius* | Native | 52.4207 | -3.02405 | Open Source |
| *Mustela putorius* | Native | 52.70184 | -3.007019999 | Open Source |
| *Mustela putorius* | Native | 52.8044 | -2.99752 | Open Source |
| *Mustela putorius* | Native | 52.44171 | -2.846230001 | Open Source |
| *Mustela putorius* | Native | 52.63865 | -2.661250001 | Open Source |
| *Mustela putorius* | Native | 52.94987 | -2.62878 | Open Source |
| *Mustela putorius* | Native | 52.97782 | -2.39983 | Open Source |
| *Mustela putorius* | Native | 52.77128 | -2.91902 | Open Source |
| *Mustela putorius* | Native | 52.96233 | -2.65129 | Open Source |
| *Mustela putorius* | Native | 52.86099 | -3.11 | Open Source |
| *Mustela putorius* | Native | 52.63395 | -2.607019999 | Open Source |
| *Mustela putorius* | Native | 52.9099 | -2.840830001 | Open Source |
| *Mustela putorius* | Native | 52.82186 | -3.10699 | Open Source |
| *Mustela putorius* | Native | 52.69296 | -2.74936 | Open Source |
| *Mustela putorius* | Native | 52.43135 | -2.88023 | Open Source |
| *Mustela putorius* | Native | 52.68682 | -2.72411 | Open Source |
| *Mustela putorius* | Native | 52.50478 | -2.918529999 | Open Source |
| *Mustela putorius* | Native | 52.45684 | -2.836589999 | Open Source |
| *Mustela putorius* | Native | 52.49962 | -2.601710001 | Open Source |
| *Mustela putorius* | Native | 52.7607 | -2.91553 | Open Source |
| *Mustela putorius* | Native | 52.68267 | -2.66635 | Open Source |
| *Mustela putorius* | Native | 52.83894 | -3.05769 | Open Source |
| *Mustela putorius* | Native | 52.62136 | -2.83677 | Open Source |
| *Mustela putorius* | Native | 52.71276 | -2.877000001 | Open Source |
| *Mustela putorius* | Native | 52.32911 | -2.709450001 | Open Source |
| *Mustela putorius* | Native | 52.70326 | -2.94341 | Open Source |
| *Mustela putorius* | Native | 52.64508 | -2.91112 | Open Source |
| *Mustela putorius* | Native | 52.81103 | -3.07786 | Open Source |
| *Mustela putorius* | Native | 52.49927 | -2.87851 | Open Source |
| *Mustela putorius* | Native | 52.61951 | -2.84412 | Open Source |
| *Mustela putorius* | Native | 52.64612 | -2.89193 | Open Source |
| *Mustela putorius* | Native | 52.84397 | -2.787219999 | Open Source |
| *Mustela putorius* | Native | 52.71844 | -2.699460001 | Open Source |
| *Mustela putorius* | Native | 52.84089 | -2.98871 | Open Source |
| *Mustela putorius* | Native | 52.70765 | -2.53596 | Open Source |
| *Mustela putorius* | Native | 52.79643 | -2.92837 | Open Source |
| *Mustela putorius* | Native | 52.43868 | -2.72739 | Open Source |
| *Mustela putorius* | Native | 52.93303 | -2.58948 | Open Source |
| *Mustela putorius* | Native | 52.73346 | -2.421509999 | Open Source |
| *Mustela putorius* | Native | 52.87086 | -3.04205 | Open Source |
| *Mustela putorius* | Native | 52.49631 | -2.99281 | Open Source |
| *Mustela putorius* | Native | 52.75182 | -2.88298 | Open Source |
| *Mustela putorius* | Native | 52.31653 | -2.70778 | Open Source |
| *Mustela putorius* | Native | 52.69493 | -2.326140001 | Open Source |
| *Mustela putorius* | Native | 52.41775 | -3.006409999 | Open Source |
| *Mustela putorius* | Native | 52.8129 | -2.68763 | Open Source |
| *Mustela putorius* | Native | 52.83908 | -3.04285 | Open Source |
| *Mustela putorius* | Native | 52.5413 | -2.48085 | Open Source |
| *Mustela putorius* | Native | 52.72383 | -2.83873 | Open Source |
| *Mustela putorius* | Native | 52.71473 | -2.85336 | Open Source |
| *Mustela putorius* | Native | 52.94264 | -2.544589999 | Open Source |
| *Mustela putorius* | Native | 52.88499 | -2.65607 | Open Source |
| *Mustela putorius* | Native | 52.52174 | -2.818 | Open Source |
| *Mustela putorius* | Native | 52.81375 | -2.68771 | Open Source |
| *Mustela putorius* | Native | 52.9661 | -2.41163 | Open Source |
| *Mustela putorius* | Native | 52.88525 | -3.040909999 | Open Source |
| *Mustela putorius* | Native | 52.32512 | -2.59266 | Open Source |
| *Mustela putorius* | Native | 52.62385 | -3.038389999 | Open Source |
| *Mustela putorius* | Native | 52.68419 | -2.71371 | Open Source |
| *Mustela putorius* | Native | 52.70527 | -2.79249 | Open Source |
| *Mustela putorius* | Native | 52.65545 | -2.27862 | Open Source |
| *Mustela putorius* | Native | 52.70702 | -2.53581 | Open Source |
| *Mustela putorius* | Native | 52.41125 | -2.72914 | Open Source |
| *Mustela putorius* | Native | 52.83155 | -2.55879 | Open Source |
| *Mustela putorius* | Native | 52.87074 | -3.119379999 | Open Source |
| *Mustela putorius* | Native | 52.90405 | -2.860569999 | Open Source |
| *Mustela putorius* | Native | 52.60139 | -2.97814 | Open Source |
| *Mustela putorius* | Native | 52.64698 | -2.89128 | Open Source |
| *Mustela putorius* | Native | 52.62488 | -2.84718 | Open Source |
| *Mustela putorius* | Native | 52.70246 | -2.66369 | Open Source |
| *Mustela putorius* | Native | 52.45396 | -2.727640001 | Open Source |
| *Mustela putorius* | Native | 52.75744 | -2.913470001 | Open Source |
| *Mustela putorius* | Native | 52.61392 | -2.686500001 | Open Source |
| *Mustela putorius* | Native | 52.75313 | -2.88819 | Open Source |
| *Mustela putorius* | Native | 52.34416 | -2.58785 | Open Source |
| *Mustela putorius* | Native | 52.44808 | -3.01895 | Open Source |
| *Mustela putorius* | Native | 52.8325 | -2.6791 | Open Source |
| *Mustela putorius* | Native | 52.58095 | -2.35289 | Open Source |
| *Mustela putorius* | Native | 52.89181 | -2.856829999 | Open Source |
| *Mustela putorius* | Native | 52.76382 | -2.774289999 | Open Source |
| *Mustela putorius* | Native | 52.8342 | -2.73249 | Open Source |
| *Mustela putorius* | Native | 52.94264 | -2.544589999 | Open Source |
| *Mustela putorius* | Native | 52.44058 | -2.84658 | Open Source |
| *Mustela putorius* | Native | 52.86757 | -2.81914 | Open Source |
| *Mustela putorius* | Native | 52.75653 | -2.908189999 | Open Source |
| *Mustela putorius* | Native | 52.53096 | -2.42377 | Open Source |
| *Mustela putorius* | Native | 52.62666 | -2.96688 | Open Source |
| *Mustela putorius* | Native | 52.82915 | -2.60009 | Open Source |
| *Mustela putorius* | Native | 52.94264 | -2.544589999 | Open Source |
| *Mustela putorius* | Native | 52.85994 | -2.78491 | Open Source |
| *Mustela putorius* | Native | 52.68708 | -2.94306 | Open Source |
| *Mustela putorius* | Native | 52.7703 | -2.35939 | Open Source |
| *Mustela putorius* | Native | 52.83654 | -2.986900001 | Open Source |
| *Mustela putorius* | Native | 52.74139 | -2.40359 | Open Source |
| *Mustela putorius* | Native | 52.39848 | -2.84482 | Open Source |
| *Mustela putorius* | Native | 52.38313 | -3.00232 | Open Source |
| *Mustela putorius* | Native | 52.58499 | -2.599109999 | Open Source |
| *Mustela putorius* | Native | 52.49071 | -2.990410001 | Open Source |
| *Mustela putorius* | Native | 52.39781 | -2.791380001 | Open Source |
| *Mustela putorius* | Native | 52.85102 | -2.9651 | Open Source |
| *Mustela putorius* | Native | 52.75866 | -2.35744 | Open Source |
| *Mustela putorius* | Native | 52.3298 | -2.57423 | Open Source |
| *Mustela putorius* | Native | 52.88912 | -2.85529 | Open Source |
| *Mustela putorius* | Native | 52.84768 | -3.086119999 | Open Source |
| *Mustela putorius* | Native | 52.69578 | -2.32622 | Open Source |
| *Mustela putorius* | Native | 52.65617 | -2.87144 | Open Source |
| *Mustela putorius* | Native | 52.43035 | -2.8997 | Open Source |
| *Mustela putorius* | Native | 52.70708 | -2.91537 | Open Source |
| *Mustela putorius* | Native | 52.5454 | -2.834590001 | Open Source |
| *Mustela putorius* | Native | 52.91769 | -3.04318 | Open Source |
| *Mustela putorius* | Native | 52.72395 | -2.83999 | Open Source |
| *Mustela putorius* | Native | 52.684 | -2.74477 | Open Source |
| *Mustela putorius* | Native | 52.90492 | -2.85137 | Open Source |
| *Mustela putorius* | Native | 52.75679 | -2.36817 | Open Source |
| *Mustela putorius* | Native | 52.70527 | -2.79249 | Open Source |
| *Mustela putorius* | Native | 52.50863 | -2.98219 | Open Source |
| *Mustela putorius* | Native | 52.73589 | -2.542009999 | Open Source |
| *Mustela putorius* | Native | 52.86084 | -2.78493 | Open Source |
| *Mustela putorius* | Native | 52.84585 | -3.089040001 | Open Source |
| *Mustela putorius* | Native | 52.67901 | -2.67665 | Open Source |
| *Mustela putorius* | Native | 52.63756 | -2.51937 | Open Source |
| *Mustela putorius* | Native | 52.6273 | -2.38037 | Open Source |
| *Mustela putorius* | Native | 52.58032 | -2.68154 | Open Source |
| *Mustela putorius* | Native | 52.56187 | -2.31195 | Open Source |
| *Mustela putorius* | Native | 52.54807 | -2.41361 | Open Source |
| *Mustela putorius* | Native | 52.64508 | -2.91112 | Open Source |
| *Mustela putorius* | Native | 52.70677 | -2.69632 | Open Source |
| *Mustela putorius* | Native | 52.64193 | -2.7145 | Open Source |
| *Mustela putorius* | Native | 52.37361 | -2.63084 | Open Source |
| *Mustela putorius* | Native | 52.78427 | -2.663450001 | Open Source |
| *Mustela putorius* | Native | 52.86925 | -3.08666 | Open Source |
| *Mustela putorius* | Native | 52.8115 | -2.61491 | Open Source |
| *Mustela putorius* | Native | 52.70781 | -2.81474 | Open Source |
| *Mustela putorius* | Native | 52.40109 | -2.837 | Open Source |
| *Mustela putorius* | Native | 52.3648 | -2.75261 | Open Source |
| *Mustela putorius* | Native | 52.69259 | -2.80558 | Open Source |
| *Mustela putorius* | Native | 52.60683 | -2.972349999 | Open Source |
| *Mustela putorius* | Native | 52.96644 | -2.71835 | Open Source |
| *Mustela putorius* | Native | 52.85135 | -2.990500001 | Open Source |
| *Mustela putorius* | Native | 52.84076 | -3.003549999 | Open Source |
| *Mustela putorius* | Native | 52.72383 | -2.83873 | Open Source |
| *Mustela putorius* | Native | 52.42429 | -2.728619999 | Open Source |
| *Mustela putorius* | Native | 52.56365 | -2.31786 | Open Source |
| *Mustela putorius* | Native | 52.74801 | -2.780009999 | Open Source |
| *Mustela putorius* | Native | 52.42905 | -2.89305 | Open Source |
| *Mustela putorius* | Native | 52.65587 | -2.28971 | Open Source |
| *Mustela putorius* | Native | 52.58038 | -2.54961 | Open Source |
| *Mustela putorius* | Native | 52.48304 | -2.81794 | Open Source |
| *Mustela putorius* | Native | 52.96234 | -2.673400001 | Open Source |
| *Mustela putorius* | Native | 52.69491 | -2.31586 | Open Source |
| *Mustela putorius* | Native | 52.90113 | -2.510550001 | Open Source |
| *Mustela putorius* | Native | 52.43088 | -2.827199999 | Open Source |
| *Mustela putorius* | Native | 52.92269 | -3.04189 | Open Source |
| *Mustela putorius* | Native | 52.61212 | -2.3448 | Open Source |
| *Mustela putorius* | Native | 52.70631 | -3.01008 | Open Source |
| *Mustela putorius* | Native | 52.41894 | -2.97755 | Open Source |
| *Mustela putorius* | Native | 52.65617 | -2.87144 | Open Source |
| *Mustela putorius* | Native | 52.70327 | -2.94193 | Open Source |
| *Mustela putorius* | Native | 52.42171 | -2.84622 | Open Source |
| *Mustela putorius* | Native | 52.83197 | -2.98591 | Open Source |
| *Mustela putorius* | Native | 52.31263 | -2.705440001 | Open Source |
| *Mustela putorius* | Native | 52.61996 | -2.600920001 | Open Source |
| *Mustela putorius* | Native | 52.80197 | -3.070149999 | Open Source |
| *Mustela putorius* | Native | 52.71843 | -2.700939999 | Open Source |
| *Mustela putorius* | Native | 52.64045 | -2.65979 | Open Source |
| *Mustela putorius* | Native | 52.98095 | -2.69625 | Open Source |
| *Mustela putorius* | Native | 52.85082 | -2.46994 | Open Source |
| *Mustela putorius* | Native | 52.7623 | -2.86466 | Open Source |
| *Mustela putorius* | Native | 52.71865 | -2.329280001 | Open Source |
| *Mustela putorius* | Native | 52.97543 | -2.718500001 | Open Source |
| *Mustela putorius* | Native | 52.43031 | -2.89933 | Open Source |
| *Mustela putorius* | Native | 52.74091 | -2.84535 | Open Source |
| *Mustela putorius* | Native | 52.68775 | -2.71968 | Open Source |
| *Mustela putorius* | Native | 52.63598 | -2.6137 | Open Source |
| *Mustela putorius* | Native | 52.71954 | -2.84079 | Open Source |
| *Mustela putorius* | Native | 52.89575 | -3.038269999 | Open Source |
| *Mustela putorius* | Native | 52.85673 | -2.99122 | Open Source |
| *Mustela putorius* | Native | 52.7075 | -2.72445 | Open Source |
| *Mustela putorius* | Native | 52.75305 | -2.77114 | Open Source |
| *Mustela putorius* | Native | 52.84096 | -2.98537 | Open Source |
| *Mustela putorius* | Native | 52.83575 | -2.98525 | Open Source |
| *Mustela putorius* | Native | 52.94518 | -2.66442 | Open Source |
| *Mustela putorius* | Native | 52.68574 | -2.717650001 | Open Source |
| *Mustela putorius* | Native | 52.84435 | -2.988260001 | Open Source |
| *Mustela putorius* | Native | 52.8151 | -2.68788 | Open Source |
| *Mustela putorius* | Native | 52.94264 | -2.544589999 | Open Source |
| *Mustela putorius* | Native | 52.38099 | -2.72974 | Open Source |
| *Mustela putorius* | Native | 52.84837 | -3.10841 | Open Source |
| *Mustela putorius* | Native | 52.71669 | -2.83119 | Open Source |
| *Mustela putorius* | Native | 52.45774 | -2.83661 | Open Source |
| *Mustela putorius* | Native | 52.89001 | -2.8568 | Open Source |
| *Mustela putorius* | Native | 52.96975 | -2.69793 | Open Source |
| *Mustela putorius* | Native | 52.467 | -2.92069 | Open Source |
| *Mustela putorius* | Native | 52.63759 | -2.39302 | Open Source |
| *Mustela putorius* | Native | 52.46807 | -3.20681 | Open Source |
| *Mustela putorius* | Native | 52.69465 | -2.4002 | Open Source |
| *Mustela putorius* | Native | 52.83677 | -3.09921 | Open Source |
| *Mustela putorius* | Native | 52.84376 | -2.96138 | Open Source |
| *Mustela putorius* | Native | 52.69053 | -2.844009999 | Open Source |
| *Mustela putorius* | Native | 52.87835 | -2.71689 | Open Source |
| *Mustela putorius* | Native | 52.38073 | -3.12457 | Open Source |
| *Mustela putorius* | Native | 52.46325 | -2.994939999 | Open Source |
| *Mustela putorius* | Native | 52.83823 | -3.036890001 | Open Source |
| *Mustela putorius* | Native | 52.8115 | -2.61491 | Open Source |
| *Mustela putorius* | Native | 52.45352 | -3.0293 | Open Source |
| *Mustela putorius* | Native | 52.62485 | -2.291720001 | Open Source |
| *Mustela putorius* | Native | 52.44808 | -2.80258 | Open Source |
| *Mustela putorius* | Native | 52.73704 | -2.99006 | Open Source |
| *Mustela putorius* | Native | 52.70598 | -2.940510001 | Open Source |
| *Mustela putorius* | Native | 58.123595 | 11.758821 | Open Source |
| *Mustela putorius* | Native | 57.596581 | 12.893142 | Open Source |
| *Mustela putorius* | Native | 56.007737 | 13.614633 | Open Source |
| *Mustela putorius* | Native | 56.843277 | 15.980697 | Open Source |
| *Mustela putorius* | Native | 56.19551 | 15.086864 | Open Source |
| *Mustela putorius* | Native | 57.160054 | 12.366415 | Open Source |
| *Mustela putorius* | Native | 43.998647 | 10.224717 | Open Source |
| *Mustela putorius* | Native | 43.2823 | 10.980781 | Open Source |
| *Mustela putorius* | Native | 50.397053 | 8.141743001 | Open Source |
| *Mustela putorius* | Native | 53.652805 | 8.972491 | Open Source |
| *Mustela putorius* | Native | 50.342667 | 8.106065 | Open Source |
| *Mustela putorius* | Native | 49.849316 | 7.536431 | Open Source |
| *Mustela putorius* | Native | 54.823803 | 9.233537 | Open Source |
| *Mustela putorius* | Native | 57.06254 | 13.510195 | Open Source |
| *Mustela putorius* | Native | 58.266389 | 11.888224 | Open Source |
| *Mustela putorius* | Native | 59.50842 | 11.613124 | Open Source |
| *Mustela putorius* | Native | 59.927071 | 11.671254 | Open Source |
| *Mustela putorius* | Native | 55.576482 | 13.087546 | Open Source |
| *Mustela putorius* | Native | 55.919958 | 13.632932 | Open Source |
| *Mustela putorius* | Native | 57.74376 | 11.923482 | Open Source |
| *Mustela putorius* | Native | 56.772347 | 15.882187 | Open Source |
| *Mustela putorius* | Native | 57.743117 | 11.896829 | Open Source |
| *Mustela putorius* | Native | 50.437939 | 10.353928 | Open Source |
| *Mustela putorius* | Native | 50.730064 | 8.706767 | Open Source |
| *Mustela putorius* | Native | 59.386265 | 13.310841 | Open Source |
| *Mustela putorius* | Native | 59.414611 | 11.719286 | Open Source |
| *Mustela putorius* | Native | 59.554627 | 11.393027 | Open Source |
| *Mustela putorius* | Native | 50.105057 | 10.184755 | Open Source |
| *Mustela putorius* | Native | 55.69096 | 13.493513 | Open Source |
| *Mustela putorius* | Native | 57.845573 | 11.877552 | Open Source |
| *Mustela putorius* | Native | 58.554088 | 13.901977 | Open Source |
| *Mustela putorius* | Native | 45.028081 | 7.710066 | Open Source |
| *Mustela putorius* | Native | 57.169134 | 13.636219 | Open Source |
| *Mustela putorius* | Native | 51.988133 | 0.557725 | Open Source |
| *Mustela putorius* | Native | 59.779261 | 14.787725 | Open Source |
| *Mustela putorius* | Native | 57.924629 | 12.084712 | Open Source |
| *Mustela putorius* | Native | 56.146024 | 14.641326 | Open Source |
| *Mustela putorius* | Native | 55.999344 | 12.788102 | Open Source |
| *Mustela putorius* | Native | 50.262142 | 6.789512 | Open Source |
| *Mustela putorius* | Native | 59.143354 | 15.397501 | Open Source |
| *Mustela putorius* | Native | 57.049166 | 8.892018001 | Open Source |
| *Mustela putorius* | Native | 57.581808 | 10.230375 | Open Source |
| *Mustela putorius* | Native | 55.042661 | 11.965933 | Open Source |
| *Mustela putorius* | Native | 57.031281 | 10.000681 | Open Source |
| *Mustela putorius* | Native | 55.931609 | 11.681504 | Open Source |
| *Mustela putorius* | Native | 55.794465 | 8.80899 | Open Source |
| *Mustela putorius* | Native | 55.292511 | 12.01515 | Open Source |
| *Mustela putorius* | Native | 54.890381 | 9.061379 | Open Source |
| *Mustela putorius* | Native | 55.883086 | 11.590568 | Open Source |
| *Mustela putorius* | Native | 55.584903 | 11.316272 | Open Source |
| *Mustela putorius* | Native | 55.539218 | 11.591597 | Open Source |
| *Mustela putorius* | Native | 55.6512 | 11.283212 | Open Source |
| *Mustela putorius* | Native | 54.905717 | 9.833701001 | Open Source |
| *Mustela putorius* | Native | 57.590332 | 10.304923 | Open Source |
| *Mustela putorius* | Native | 54.801968 | 11.636475 | Open Source |
| *Mustela putorius* | Native | 55.751266 | 11.960185 | Open Source |
| *Mustela putorius* | Native | 55.827613 | 11.624676 | Open Source |
| *Mustela putorius* | Native | 55.567431 | 11.314831 | Open Source |
| *Mustela putorius* | Native | 55.445275 | 11.7976 | Open Source |
| *Mustela putorius* | Native | 56.455743 | 9.683695001 | Open Source |
| *Mustela putorius* | Native | 55.551799 | 8.324441999 | Open Source |
| *Mustela putorius* | Native | 55.951439 | 11.454272 | Open Source |
| *Mustela putorius* | Native | 55.926866 | 11.728839 | Open Source |
| *Mustela putorius* | Native | 56.913624 | 10.218427 | Open Source |
| *Mustela putorius* | Native | 55.973595 | 11.774882 | Open Source |
| *Mustela putorius* | Native | 55.470097 | 11.922115 | Open Source |
| *Mustela putorius* | Native | 55.223506 | 12.150409 | Open Source |
| *Mustela putorius* | Native | 55.960075 | 12.536167 | Open Source |
| *Mustela putorius* | Native | 55.920094 | 11.714372 | Open Source |
| *Mustela putorius* | Native | 55.717175 | 11.710831 | Open Source |
| *Mustela putorius* | Native | 57.093829 | 9.054955 | Open Source |
| *Mustela putorius* | Native | 54.823803 | 11.944533 | Open Source |
| *Mustela putorius* | Native | 55.581938 | 10.170726 | Open Source |
| *Mustela putorius* | Native | 55.944612 | 12.383173 | Open Source |
| *Mustela putorius* | Native | 55.223248 | 9.997033 | Open Source |
| *Mustela putorius* | Native | 54.936944 | 11.969868 | Open Source |
| *Mustela putorius* | Native | 55.97777 | 12.398529 | Open Source |
| *Mustela putorius* | Native | 55.914715 | 11.652248 | Open Source |
| *Mustela putorius* | Native | 56.005117 | 12.5131 | Open Source |
| *Mustela putorius* | Native | 56.384117 | 10.171474 | Open Source |
| *Mustela putorius* | Native | 55.914715 | 11.652248 | Open Source |
| *Mustela putorius* | Native | 55.059001 | 10.66052 | Open Source |
| *Mustela putorius* | Native | 57.542266 | 10.19922 | Open Source |
| *Mustela putorius* | Native | 55.554814 | 8.118638 | Open Source |
| *Mustela putorius* | Native | 57.581575 | 10.203273 | Open Source |
| *Mustela putorius* | Native | 57.582023 | 10.15037 | Open Source |
| *Mustela putorius* | Native | 57.582023 | 10.15037 | Open Source |
| *Mustela putorius* | Native | 55.294892 | 8.763442999 | Open Source |
| *Mustela putorius* | Native | 55.310648 | 11.732818 | Open Source |
| *Mustela putorius* | Native | 55.407316 | 12.109915 | Open Source |
| *Mustela putorius* | Native | 55.539218 | 11.591597 | Open Source |
| *Mustela putorius* | Native | 55.81953 | 11.574175 | Open Source |
| *Mustela putorius* | Native | 57.208746 | 9.820177 | Open Source |
| *Mustela putorius* | Native | 55.288787 | 11.85064 | Open Source |
| *Mustela putorius* | Native | 55.432941 | 11.951732 | Open Source |
| *Mustela putorius* | Native | 55.515553 | 11.098236 | Open Source |
| *Mustela putorius* | Native | 55.288113 | 12.03298 | Open Source |
| *Mustela putorius* | Native | 55.922937 | 11.666097 | Open Source |
| *Mustela putorius* | Native | 55.990933 | 11.909011 | Open Source |
| *Mustela putorius* | Native | 55.256982 | 12.085213 | Open Source |
| *Mustela putorius* | Native | 55.283281 | 12.003739 | Open Source |
| *Mustela putorius* | Native | 56.127671 | 8.790209 | Open Source |
| *Mustela putorius* | Native | 55.958772 | 11.764911 | Open Source |
| *Mustela putorius* | Native | 54.936656 | 8.661569 | Open Source |
| *Mustela putorius* | Native | 56.902918 | 10.208817 | Open Source |
| *Mustela putorius* | Native | 55.467718 | 12.111159 | Open Source |
| *Mustela putorius* | Native | 55.25084 | 10.641873 | Open Source |
| *Mustela putorius* | Native | 55.46711 | 11.888552 | Open Source |
| *Mustela putorius* | Native | 55.185227 | 12.036545 | Open Source |
| *Mustela putorius* | Native | 55.833341 | 12.262468 | Open Source |
| *Mustela putorius* | Native | 55.455402 | 12.138586 | Open Source |
| *Mustela putorius* | Native | 55.57697 | 11.315154 | Open Source |
| *Mustela putorius* | Native | 56.756365 | 8.561446 | Open Source |
| *Mustela putorius* | Native | 55.908162 | 12.498301 | Open Source |
| *Mustela putorius* | Native | 55.599921 | 11.29725 | Open Source |
| *Mustela putorius* | Native | 56.926612 | 8.509177 | Open Source |
| *Mustela putorius* | Native | 55.861462 | 9.845471999 | Open Source |
| *Mustela putorius* | Native | 56.281396 | 10.041081 | Open Source |
| *Mustela putorius* | Native | 56.307361 | 10.4443 | Open Source |
| *Mustela putorius* | Native | 55.222598 | 12.166364 | Open Source |
| *Mustela putorius* | Native | 55.222598 | 12.166364 | Open Source |
| *Mustela putorius* | Native | 55.311092 | 12.059309 | Open Source |
| *Mustela putorius* | Native | 57.639335 | 10.441633 | Open Source |
| *Mustela putorius* | Native | 55.654497 | 12.236068 | Open Source |
| *Mustela putorius* | Native | 57.007443 | 8.939821001 | Open Source |
| *Mustela putorius* | Native | 56.459988 | 9.919593 | Open Source |
| *Mustela putorius* | Native | 55.787064 | 12.34345 | Open Source |
| *Mustela putorius* | Native | 55.431884 | 12.070913 | Open Source |
| *Mustela putorius* | Native | 55.394545 | 12.107237 | Open Source |
| *Mustela putorius* | Native | 55.237822 | 9.373096001 | Open Source |
| *Mustela putorius* | Native | 55.407316 | 12.109915 | Open Source |
| *Mustela putorius* | Native | 55.804239 | 12.093129 | Open Source |
| *Mustela putorius* | Native | 55.804239 | 12.093129 | Open Source |
| *Mustela putorius* | Native | 55.232069 | 12.213968 | Open Source |
| *Mustela putorius* | Native | 55.462928 | 9.694819 | Open Source |
| *Mustela putorius* | Native | 55.491592 | 11.862398 | Open Source |
| *Mustela putorius* | Native | 55.256202 | 12.024471 | Open Source |
| *Mustela putorius* | Native | 55.942239 | 11.754186 | Open Source |
| *Mustela putorius* | Native | 55.651952 | 11.50312 | Open Source |
| *Mustela putorius* | Native | 56.086653 | 8.259492 | Open Source |
| *Mustela putorius* | Native | 55.958772 | 11.764911 | Open Source |
| *Mustela putorius* | Native | 54.995221 | 8.972333 | Open Source |
| *Mustela putorius* | Native | 57.244343 | 9.842609 | Open Source |
| *Mustela putorius* | Native | 55.805528 | 11.98322 | Open Source |
| *Mustela putorius* | Native | 57.09269 | 9.017427001 | Open Source |
| *Mustela putorius* | Native | 57.067966 | 9.885611 | Open Source |
| *Mustela putorius* | Native | 55.44223 | 12.199648 | Open Source |
| *Mustela putorius* | Native | 55.254403 | 11.99498 | Open Source |
| *Mustela putorius* | Native | 55.223506 | 12.150409 | Open Source |
| *Mustela putorius* | Native | 55.307585 | 12.199224 | Open Source |
| *Mustela putorius* | Native | 57.538442 | 10.440437 | Open Source |
| *Mustela putorius* | Native | 57.538442 | 10.440437 | Open Source |
| *Mustela putorius* | Native | 56.992141 | 8.858362001 | Open Source |
| *Mustela putorius* | Native | 55.645784 | 9.511433999 | Open Source |
| *Mustela putorius* | Native | 56.926612 | 8.509177 | Open Source |
| *Mustela putorius* | Native | 55.747896 | 12.533587 | Open Source |
| *Mustela putorius* | Native | 55.639948 | 8.710356 | Open Source |
| *Mustela putorius* | Native | 55.747896 | 12.533587 | Open Source |
| *Mustela putorius* | Native | 56.858947 | 9.932213001 | Open Source |
| *Mustela putorius* | Native | 55.154501 | 12.098519 | Open Source |
| *Mustela putorius* | Native | 55.747896 | 12.533587 | Open Source |
| *Mustela putorius* | Native | 57.740185 | 10.627812 | Open Source |
| *Mustela putorius* | Native | 55.129929 | 10.3805 | Open Source |
| *Mustela putorius* | Native | 55.481285 | 11.514902 | Open Source |
| *Mustela putorius* | Native | 56.690032 | 9.91303 | Open Source |
| *Mustela putorius* | Native | 55.161198 | 8.702645 | Open Source |
| *Mustela putorius* | Native | 57.112732 | 9.069992 | Open Source |
| *Mustela putorius* | Native | 55.390463 | 11.243303 | Open Source |
| *Mustela putorius* | Native | 56.450355 | 9.586695 | Open Source |
| *Mustela putorius* | Native | 55.398457 | 8.661219 | Open Source |
| *Mustela putorius* | Native | 56.972368 | 8.451347 | Open Source |
| *Mustela putorius* | Native | 56.892579 | 9.832344 | Open Source |
| *Mustela putorius* | Native | 55.951174 | 12.335786 | Open Source |
| *Mustela putorius* | Native | 55.232069 | 12.213968 | Open Source |
| *Mustela putorius* | Native | 55.273023 | 12.224884 | Open Source |
| *Mustela putorius* | Native | 55.401118 | 11.358153 | Open Source |
| *Mustela putorius* | Native | 54.900931 | 8.753719 | Open Source |
| *Mustela putorius* | Native | 55.940332 | 11.682972 | Open Source |
| *Mustela putorius* | Native | 55.139085 | 11.979127 | Open Source |
| *Mustela putorius* | Native | 55.370034 | 11.495385 | Open Source |
| *Mustela putorius* | Native | 55.942052 | 11.716854 | Open Source |
| *Mustela putorius* | Native | 56.970868 | 10.222615 | Open Source |
| *Mustela putorius* | Native | 55.014977 | 11.925947 | Open Source |
| *Mustela putorius* | Native | 55.258607 | 11.845692 | Open Source |
| *Mustela putorius* | Native | 55.422811 | 12.123131 | Open Source |
| *Mustela putorius* | Native | 55.914255 | 12.099713 | Open Source |
| *Mustela putorius* | Native | 55.154501 | 12.098519 | Open Source |
| *Mustela putorius* | Native | 55.222598 | 12.166364 | Open Source |
| *Mustela putorius* | Native | 56.473312 | 9.829056001 | Open Source |
| *Mustela putorius* | Native | 55.747896 | 12.533587 | Open Source |
| *Mustela putorius* | Native | 55.312728 | 12.043861 | Open Source |
| *Mustela putorius* | Native | 55.083575 | 11.762405 | Open Source |
| *Mustela putorius* | Native | 55.972223 | 12.251024 | Open Source |
| *Mustela putorius* | Native | 55.25143 | 9.644119 | Open Source |
| *Mustela putorius* | Native | 56.669579 | 8.4225 | Open Source |
| *Mustela putorius* | Native | 57.048588 | 9.088019 | Open Source |
| *Mustela putorius* | Native | 56.669579 | 8.4225 | Open Source |
| *Mustela putorius* | Native | 57.048588 | 9.088019 | Open Source |
| *Mustela putorius* | Native | 57.519172 | 10.457576 | Open Source |
| *Mustela putorius* | Native | 55.857216 | 8.234834 | Open Source |
| *Mustela putorius* | Native | 55.237822 | 9.373096001 | Open Source |
| *Mustela putorius* | Native | 55.968241 | 9.226374001 | Open Source |
| *Mustela putorius* | Native | 55.390463 | 11.243303 | Open Source |
| *Mustela putorius* | Native | 57.02796 | 8.937399001 | Open Source |
| *Mustela putorius* | Native | 55.680775 | 12.081476 | Open Source |
| *Mustela putorius* | Native | 56.440387 | 9.487896999 | Open Source |
| *Mustela putorius* | Native | 55.390463 | 11.243303 | Open Source |
| *Mustela putorius* | Native | 55.237822 | 9.373096001 | Open Source |
| *Mustela putorius* | Native | 56.176988 | 9.775735 | Open Source |
| *Mustela putorius* | Native | 55.649523 | 11.492156 | Open Source |
| *Mustela putorius* | Native | 55.583023 | 11.224666 | Open Source |
| *Mustela putorius* | Native | 56.075419 | 12.321702 | Open Source |
| *Mustela putorius* | Native | 55.221096 | 9.399443 | Open Source |
| *Mustela putorius* | Native | 56.033497 | 9.464441 | Open Source |
| *Mustela putorius* | Native | 56.385438 | 10.504545 | Open Source |
| *Mustela putorius* | Native | 55.690624 | 12.328697 | Open Source |
| *Mustela putorius* | Native | 55.121203 | 12.052828 | Open Source |
| *Mustela putorius* | Native | 56.913624 | 10.218427 | Open Source |
| *Mustela putorius* | Native | 56.913624 | 10.218427 | Open Source |
| *Mustela putorius* | Native | 55.223506 | 12.150409 | Open Source |
| *Mustela putorius* | Native | 55.275708 | 11.876253 | Open Source |
| *Mustela putorius* | Native | 55.363344 | 12.06219 | Open Source |
| *Mustela putorius* | Native | 54.929911 | 11.947445 | Open Source |
| *Mustela putorius* | Native | 57.093829 | 9.054955 | Open Source |
| *Mustela putorius* | Native | 55.747896 | 12.533587 | Open Source |
| *Mustela putorius* | Native | 55.747896 | 12.533587 | Open Source |
| *Mustela putorius* | Native | 55.482186 | 9.200492999 | Open Source |
| *Mustela putorius* | Native | 55.439719 | 11.662326 | Open Source |
| *Mustela putorius* | Native | 55.256919 | 12.110001 | Open Source |
| *Mustela putorius* | Native | 56.153995 | 9.908874 | Open Source |
| *Mustela putorius* | Native | 56.26186 | 8.802504 | Open Source |
| *Mustela putorius* | Native | 55.879379 | 9.944280001 | Open Source |
| *Mustela putorius* | Native | 55.28974 | 9.533914 | Open Source |
| *Mustela putorius* | Native | 57.10598 | 9.988544999 | Open Source |
| *Mustela putorius* | Native | 55.717582 | 12.50708 | Open Source |
| *Mustela putorius* | Native | 57.065706 | 8.999010999 | Open Source |
| *Mustela putorius* | Native | 55.398457 | 8.661219 | Open Source |
| *Mustela putorius* | Native | 56.196227 | 8.688838 | Open Source |
| *Mustela putorius* | Native | 57.086363 | 9.032998999 | Open Source |
| *Mustela putorius* | Native | 57.112732 | 9.069992 | Open Source |
| *Mustela putorius* | Native | 55.849217 | 9.590944 | Open Source |
| *Mustela putorius* | Native | 55.031095 | 8.849169001 | Open Source |
| *Mustela putorius* | Native | 56.888352 | 9.876434 | Open Source |
| *Mustela putorius* | Native | 56.706187 | 10.037984 | Open Source |
| *Mustela putorius* | Native | 55.836173 | 8.549856999 | Open Source |
| *Mustela putorius* | Native | 55.031095 | 8.849169001 | Open Source |
| *Mustela putorius* | Native | 56.79418 | 9.884059 | Open Source |
| *Mustela putorius* | Native | 55.524426 | 9.719964 | Open Source |
| *Mustela putorius* | Native | 55.465268 | 8.58313 | Open Source |
| *Mustela putorius* | Native | 57.062737 | 10.191382 | Open Source |
| *Mustela putorius* | Native | 56.046669 | 12.556239 | Open Source |
| *Mustela putorius* | Native | 56.033497 | 9.464441 | Open Source |
| *Mustela putorius* | Native | 56.458637 | 8.871307001 | Open Source |
| *Mustela putorius* | Native | 57.226085 | 10.543792 | Open Source |
| *Mustela putorius* | Native | 57.068391 | 9.049474999 | Open Source |
| *Mustela putorius* | Native | 55.643508 | 12.104394 | Open Source |
| *Mustela putorius* | Native | 55.562419 | 11.960456 | Open Source |
| *Mustela putorius* | Native | 55.445275 | 11.7976 | Open Source |
| *Mustela putorius* | Native | 56.011214 | 9.669646999 | Open Source |
| *Mustela putorius* | Native | 55.958772 | 11.764911 | Open Source |
| *Mustela putorius* | Native | 55.92909 | 12.496537 | Open Source |
| *Mustela putorius* | Native | 57.532958 | 10.285184 | Open Source |
| *Mustela putorius* | Native | 55.379923 | 9.091378999 | Open Source |
| *Mustela putorius* | Native | 55.273636 | 12.294134 | Open Source |
| *Mustela putorius* | Native | 55.908717 | 11.980388 | Open Source |
| *Mustela putorius* | Native | 55.92909 | 12.496537 | Open Source |
| *Mustela putorius* | Native | 55.908717 | 11.980388 | Open Source |
| *Mustela putorius* | Native | 55.468019 | 12.144733 | Open Source |
| *Mustela putorius* | Native | 56.921605 | 10.198677 | Open Source |
| *Mustela putorius* | Native | 55.482442 | 9.303075999 | Open Source |
| *Mustela putorius* | Native | 54.833099 | 11.766943 | Open Source |
| *Mustela putorius* | Native | 56.107533 | 8.980288001 | Open Source |
| *Mustela putorius* | Native | 57.434617 | 10.488129 | Open Source |
| *Mustela putorius* | Native | 55.752934 | 12.539097 | Open Source |
| *Mustela putorius* | Native | 56.833204 | 8.529361 | Open Source |
| *Mustela putorius* | Native | 57.479788 | 9.994335 | Open Source |
| *Mustela putorius* | Native | 55.233212 | 9.643054 | Open Source |
| *Mustela putorius* | Native | 56.165541 | 8.954863999 | Open Source |
| *Mustela putorius* | Native | 55.960075 | 12.536167 | Open Source |
| *Mustela putorius* | Native | 56.41811 | 8.70037 | Open Source |
| *Mustela putorius* | Native | 57.459137 | 9.997490999 | Open Source |
| *Mustela putorius* | Native | 55.293289 | 11.82678 | Open Source |
| *Mustela putorius* | Native | 56.083173 | 9.973797 | Open Source |
| *Mustela putorius* | Native | 55.747896 | 12.533587 | Open Source |
| *Mustela putorius* | Native | 55.747896 | 12.533587 | Open Source |
| *Mustela putorius* | Native | 55.533595 | 8.191268 | Open Source |
| *Mustela putorius* | Native | 55.747896 | 12.533587 | Open Source |
| *Mustela putorius* | Native | 55.747896 | 12.533587 | Open Source |
| *Mustela putorius* | Native | 55.747896 | 12.533587 | Open Source |
| *Mustela putorius* | Native | 55.747896 | 12.533587 | Open Source |
| *Mustela putorius* | Native | 56.155157 | 9.491327 | Open Source |
| *Mustela putorius* | Native | 56.624062 | 9.939192999 | Open Source |
| *Mustela putorius* | Native | 54.823057 | 9.248624 | Open Source |
| *Mustela putorius* | Native | 55.141992 | 11.818077 | Open Source |
| *Mustela putorius* | Native | 57.740185 | 10.627812 | Open Source |
| *Mustela putorius* | Native | 55.141992 | 11.818077 | Open Source |
| *Mustela putorius* | Native | 57.077367 | 9.065983 | Open Source |
| *Mustela putorius* | Native | 57.048588 | 9.088019 | Open Source |
| *Mustela putorius* | Native | 57.048588 | 9.088019 | Open Source |
| *Mustela putorius* | Native | 57.049166 | 8.892018001 | Open Source |
| *Mustela putorius* | Native | 57.232419 | 9.738801999 | Open Source |
| *Mustela putorius* | Native | 55.423678 | 11.401648 | Open Source |
| *Mustela putorius* | Native | 57.054238 | 9.121316999 | Open Source |
| *Mustela putorius* | Native | 56.764466 | 8.322025 | Open Source |
| *Mustela putorius* | Native | 55.449167 | 11.580959 | Open Source |
| *Mustela putorius* | Native | 57.040524 | 9.051218 | Open Source |
| *Mustela putorius* | Native | 57.186309 | 9.587010999 | Open Source |
| *Mustela putorius* | Native | 55.449167 | 11.580959 | Open Source |
| *Mustela putorius* | Native | 55.592975 | 12.130485 | Open Source |
| *Mustela putorius* | Native | 55.592975 | 12.130485 | Open Source |
| *Mustela putorius* | Native | 56.092176 | 9.548129 | Open Source |
| *Mustela putorius* | Native | 56.255498 | 10.696257 | Open Source |
| *Mustela putorius* | Native | 55.41757 | 12.118908 | Open Source |
| *Mustela putorius* | Native | 56.672927 | 9.673033999 | Open Source |
| *Mustela putorius* | Native | 55.767566 | 12.414234 | Open Source |
| *Mustela putorius* | Native | 56.420414 | 8.830748 | Open Source |
| *Mustela putorius* | Native | 55.401118 | 11.358153 | Open Source |
| *Mustela putorius* | Native | 55.401118 | 11.358153 | Open Source |
| *Mustela putorius* | Native | 54.914213 | 8.732485 | Open Source |
| *Mustela putorius* | Native | 55.319243 | 11.727545 | Open Source |
| *Mustela putorius* | Native | 55.644497 | 12.092279 | Open Source |
| *Mustela putorius* | Native | 56.450115 | 9.865735001 | Open Source |
| *Mustela putorius* | Native | 55.554061 | 9.539996999 | Open Source |
| *Mustela putorius* | Native | 55.583799 | 12.273434 | Open Source |
| *Mustela putorius* | Native | 56.887584 | 9.633280001 | Open Source |
| *Mustela putorius* | Native | 57.03013 | 8.892057 | Open Source |
| *Mustela putorius* | Native | 55.79278 | 12.525221 | Open Source |
| *Mustela putorius* | Native | 56.450775 | 9.720141001 | Open Source |
| *Mustela putorius* | Native | 55.370034 | 11.495385 | Open Source |
| *Mustela putorius* | Native | 56.154286 | 9.690373 | Open Source |
| *Mustela putorius* | Native | 55.531777 | 9.503997001 | Open Source |
| *Mustela putorius* | Native | 55.585195 | 11.55573 | Open Source |
| *Mustela putorius* | Native | 57.09269 | 9.017427001 | Open Source |
| *Mustela putorius* | Native | 54.868509 | 11.866982 | Open Source |
| *Mustela putorius* | Native | 55.513856 | 12.183565 | Open Source |
| *Mustela putorius* | Native | 56.078019 | 9.632085001 | Open Source |
| *Mustela putorius* | Native | 56.926612 | 8.509177 | Open Source |
| *Mustela putorius* | Native | 56.947546 | 10.205806 | Open Source |
| *Mustela putorius* | Native | 56.994624 | 8.832116999 | Open Source |
| *Mustela putorius* | Native | 57.093829 | 9.054955 | Open Source |
| *Mustela putorius* | Native | 56.006854 | 12.316789 | Open Source |
| *Mustela putorius* | Native | 57.58721 | 10.349825 | Open Source |
| *Mustela putorius* | Native | 56.401016 | 10.859282 | Open Source |
| *Mustela putorius* | Native | 56.265981 | 10.582526 | Open Source |
| *Mustela putorius* | Native | 57.084597 | 9.972714 | Open Source |
| *Mustela putorius* | Native | 55.258499 | 12.12837 | Open Source |
| *Mustela putorius* | Native | 56.6255 | 9.405336 | Open Source |
| *Mustela putorius* | Native | 57.048588 | 9.088019 | Open Source |
| *Mustela putorius* | Native | 57.048588 | 9.088019 | Open Source |
| *Mustela putorius* | Native | 55.662668 | 12.035345 | Open Source |
| *Mustela putorius* | Native | 57.092168 | 9.893284 | Open Source |
| *Mustela putorius* | Native | 55.208353 | 11.73451 | Open Source |
| *Mustela putorius* | Native | 56.039174 | 9.516834 | Open Source |
| *Mustela putorius* | Native | 56.886451 | 9.253723 | Open Source |
| *Mustela putorius* | Native | 55.648332 | 12.304053 | Open Source |
| *Mustela putorius* | Native | 55.570882 | 9.488607001 | Open Source |
| *Mustela putorius* | Native | 57.053393 | 8.962332001 | Open Source |
| *Mustela putorius* | Native | 55.240411 | 9.538836001 | Open Source |
| *Mustela putorius* | Native | 55.969006 | 9.721485001 | Open Source |
| *Mustela putorius* | Native | 56.571072 | 8.812759 | Open Source |
| *Mustela putorius* | Native | 56.655109 | 10.173618 | Open Source |
| *Mustela putorius* | Native | 55.620405 | 9.533207 | Open Source |
| *Mustela putorius* | Native | 56.571072 | 8.812759 | Open Source |
| *Mustela putorius* | Native | 57.068391 | 9.049474999 | Open Source |
| *Mustela putorius* | Native | 55.748155 | 11.999094 | Open Source |
| *Mustela putorius* | Native | 54.895549 | 8.758585999 | Open Source |
| *Mustela putorius* | Native | 57.082171 | 10.085421 | Open Source |
| *Mustela putorius* | Native | 55.820861 | 12.574671 | Open Source |
| *Mustela putorius* | Native | 56.216103 | 8.672392 | Open Source |
| *Mustela putorius* | Native | 55.922937 | 11.666097 | Open Source |
| *Mustela putorius* | Native | 55.908717 | 11.980388 | Open Source |
| *Mustela putorius* | Native | 56.931572 | 10.281687 | Open Source |
| *Mustela putorius* | Native | 55.802011 | 9.696745999 | Open Source |
| *Mustela putorius* | Native | 55.458792 | 11.832524 | Open Source |
| *Mustela putorius* | Native | 55.56467 | 10.199184 | Open Source |
| *Mustela putorius* | Native | 55.770265 | 12.503839 | Open Source |
| *Mustela putorius* | Native | 55.565568 | 9.347559 | Open Source |
| *Mustela putorius* | Native | 56.947546 | 10.205806 | Open Source |
| *Mustela putorius* | Native | 57.154318 | 9.766949999 | Open Source |
| *Mustela putorius* | Native | 55.859247 | 9.818152 | Open Source |
| *Mustela putorius* | Native | 57.121603 | 9.791097 | Open Source |
| *Mustela putorius* | Native | 55.272993 | 11.9789 | Open Source |
| *Mustela putorius* | Native | 54.963369 | 9.427947 | Open Source |
| *Mustela putorius* | Native | 55.485363 | 11.795106 | Open Source |
| *Mustela putorius* | Native | 55.747896 | 12.533587 | Open Source |
| *Mustela putorius* | Native | 55.552754 | 12.220859 | Open Source |
| *Mustela putorius* | Native | 55.658299 | 12.184952 | Open Source |
| *Mustela putorius* | Native | 55.658299 | 12.184952 | Open Source |
| *Mustela putorius* | Native | 57.270528 | 9.654331001 | Open Source |
| *Mustela putorius* | Native | 55.658299 | 12.184952 | Open Source |
| *Mustela putorius* | Native | 55.444293 | 12.192271 | Open Source |
| *Mustela putorius* | Native | 57.475895 | 10.456169 | Open Source |
| *Mustela putorius* | Native | 55.889841 | 9.819755 | Open Source |
| *Mustela putorius* | Native | 56.003439 | 8.453736999 | Open Source |
| *Mustela putorius* | Native | 55.619937 | 9.497013 | Open Source |
| *Mustela putorius* | Native | 55.619937 | 9.497013 | Open Source |
| *Mustela putorius* | Native | 59.521513 | 11.586084 | Open Source |
| *Mustela putorius* | Native | 59.415333 | 11.669394 | Open Source |
| *Mustela putorius* | Native | 59.369325 | 10.704693 | Open Source |
| *Mustela putorius* | Native | 57.116121 | 15.374891 | Open Source |
| *Mustela putorius* | Native | 58.489393 | 11.448912 | Open Source |
| *Mustela putorius* | Native | 56.371928 | 16.535065 | Open Source |
| *Mustela putorius* | Native | 56.858382 | 16.245044 | Open Source |
| *Mustela putorius* | Native | 56.116993 | 13.135993 | Open Source |
| *Mustela putorius* | Native | 59.387417 | 13.184555 | Open Source |
| *Mustela putorius* | Native | 57.731863 | 12.899754 | Open Source |
| *Mustela putorius* | Native | 56.262378 | 15.031707 | Open Source |
| *Mustela putorius* | Native | 48.724434 | 13.689374 | Open Source |
| *Mustela putorius* | Native | 52.570408 | 12.001528 | Open Source |
| *Mustela putorius* | Native | 52.054626 | 4.864924 | Open Source |
| *Mustela putorius* | Native | 50.714405 | 6.471969 | Open Source |
| *Mustela putorius* | Native | 50.879753 | 7.094738001 | Open Source |
| *Mustela putorius* | Native | 52.691734 | 6.614628001 | Open Source |
| *Mustela putorius* | Native | 49.349155 | 7.757968001 | Open Source |
| *Mustela putorius* | Native | 49.394794 | 8.222015 | Open Source |
| *Mustela putorius* | Native | 49.890339 | 8.18339 | Open Source |
| *Mustela putorius* | Native | 50.684078 | 6.754285001 | Open Source |
| *Mustela putorius* | Native | 48.874706 | 10.263184 | Open Source |
| *Mustela putorius* | Native | 51.823772 | 6.738309999 | Open Source |
| *Mustela putorius* | Native | 51.317616 | 12.630676 | Open Source |
| *Mustela putorius* | Native | 54.200298 | 9.971531 | Open Source |
| *Mustela putorius* | Native | 50.275738 | 8.761597 | Open Source |
| *Mustela putorius* | Native | 50.049896 | 10.926857 | Open Source |
| *Mustela putorius* | Native | 50.190529 | 10.215397 | Open Source |
| *Mustela putorius* | Native | 50.303345 | 7.073568 | Open Source |
| *Mustela putorius* | Native | 50.095036 | 7.063522001 | Open Source |
| *Mustela putorius* | Native | 52.582558 | 10.073218 | Open Source |
| *Mustela putorius* | Native | 50.565662 | 6.583214 | Open Source |
| *Mustela putorius* | Native | 51.137569 | 12.142296 | Open Source |
| *Mustela putorius* | Native | 53.036671 | 6.582355 | Open Source |
| *Mustela putorius* | Native | 50.611828 | 6.642540001 | Open Source |
| *Mustela putorius* | Native | 53.977417 | 10.605583 | Open Source |
| *Mustela putorius* | Native | 53.061283 | 8.473721001 | Open Source |
| *Mustela putorius* | Native | 51.907082 | 7.63494 | Open Source |
| *Mustela putorius* | Native | 49.367954 | 7.730598 | Open Source |
| *Mustela putorius* | Native | 51.353756 | 12.45903 | Open Source |
| *Mustela putorius* | Native | 52.046738 | 4.827375 | Open Source |
| *Mustela putorius* | Native | 51.818378 | 7.178192001 | Open Source |
| *Mustela putorius* | Native | 49.375668 | 7.562714 | Open Source |
| *Mustela putorius* | Native | 50.556095 | 6.447717 | Open Source |
| *Mustela putorius* | Native | 50.706978 | 6.901345 | Open Source |
| *Mustela putorius* | Native | 52.959213 | 11.316903 | Open Source |
| *Mustela putorius* | Native | 47.729519 | 9.078395 | Open Source |
| *Mustela putorius* | Native | 48.231075 | 13.373837 | Open Source |
| *Mustela putorius* | Native | 51.757801 | 13.208313 | Open Source |
| *Mustela putorius* | Native | 50.623821 | 6.666881999 | Open Source |
| *Mustela putorius* | Native | 45.194695 | 8.114997 | Open Source |
| *Mustela putorius* | Native | 45.188933 | 8.118553 | Open Source |
| *Mustela putorius* | Native | 55.74813 | 13.539794 | Open Source |
| *Mustela putorius* | Native | 55.63858 | 13.049575 | Open Source |
| *Mustela putorius* | Native | 59.380788 | 13.456844 | Open Source |
| *Mustela putorius* | Native | 57.196908 | 14.598279 | Open Source |
| *Mustela putorius* | Native | 57.839716 | 11.79346 | Open Source |
| *Mustela putorius* | Native | 56.015998 | 14.462382 | Open Source |
| *Mustela putorius* | Native | 58.961907 | 15.145039 | Open Source |
| *Mustela putorius* | Native | 43.483163 | 10.386972 | Open Source |
| *Mustela putorius* | Native | 56.225564 | 10.578974 | Open Source |
| *Mustela putorius* | Native | 47.729551 | 9.078457 | Open Source |
| *Mustela putorius* | Native | 48.637757 | -4.293650001 | Open Source |
| *Mustela putorius* | Native | 55.670924 | 13.495193 | Open Source |
| *Mustela putorius* | Native | 58.110187 | 13.491887 | Open Source |
| *Mustela putorius* | Native | 56.632298 | 15.051614 | Open Source |
| *Mustela putorius* | Native | 52.71669 | -2.76383 | Open Source |
| *Mustela putorius* | Native | 54.38641 | -2.58587 | Open Source |
| *Mustela putorius* | Native | 50.74096 | -2.3748 | Open Source |
| *Mustela putorius* | Native | 51.87806 | 0.437069999 | Open Source |
| *Mustela putorius* | Native | 51.828 | -9.96E-02 | Open Source |
| *Mustela putorius* | Native | 52.3296 | -2.0236 | Open Source |
| *Mustela putorius* | Native | 51.87414 | 0.49497 | Open Source |
| *Mustela putorius* | Native | 51.91101 | 0.86999 | Open Source |
| *Mustela putorius* | Native | 52.6776 | -2.39117 | Open Source |
| *Mustela putorius* | Native | 52.49464 | -2.22899 | Open Source |
| *Mustela putorius* | Native | 51.9319 | -7.40E-03 | Open Source |
| *Mustela putorius* | Native | 50.40329 | -4.31616 | Open Source |
| *Mustela putorius* | Native | 52.43803 | -2.21252 | Open Source |
| *Mustela putorius* | Native | 57.6779 | -2.7499 | Open Source |
| *Mustela putorius* | Native | 52.47702 | -2.62496 | Open Source |
| *Mustela putorius* | Native | 52.66914 | -2.172229999 | Open Source |
| *Mustela putorius* | Native | 54.173478 | -2.736591 | Open Source |
| *Mustela putorius* | Native | 52.6897 | -2.5242 | Open Source |
| *Mustela putorius* | Native | 52.63495 | -2.1854 | Open Source |
| *Mustela putorius* | Native | 51.88693 | 0.692090001 | Open Source |
| *Mustela putorius* | Native | 52.12536 | -1.989 | Open Source |
| *Mustela putorius* | Native | 52.54147 | -2.176160001 | Open Source |
| *Mustela putorius* | Native | 50.74309 | -2.36893 | Open Source |
| *Mustela putorius* | Native | 53.9648 | -2.76285 | Open Source |
| *Mustela putorius* | Native | 51.64495 | 1.83E-02 | Open Source |
| *Mustela putorius* | Native | 53.1955 | -2.839800001 | Open Source |
| *Mustela putorius* | Native | 50.71432 | -2.519059999 | Open Source |
| *Mustela putorius* | Native | 52.68964 | -2.5333 | Open Source |
| *Mustela putorius* | Native | 51.91586 | 0.87394 | Open Source |
| *Mustela putorius* | Native | 52.8934 | -1.024200001 | Open Source |
| *Mustela putorius* | Native | 54.17093 | -2.75032 | Open Source |
| *Mustela putorius* | Native | 56.4643 | -5.698199999 | Open Source |
| *Mustela putorius* | Native | 52.954932 | -2.676847 | Open Source |
| *Mustela putorius* | Native | 52.47034 | -2.240640001 | Open Source |
| *Mustela putorius* | Native | 54.28493 | -2.72859 | Open Source |
| *Mustela putorius* | Native | 52.78064 | -2.93471 | Open Source |
| *Mustela putorius* | Native | 56.88728 | -2.248609999 | Open Source |
| *Mustela putorius* | Native | 43.152235 | 11.148421 | Open Source |
| *Mustela putorius* | Native | 56.659224 | 9.697067001 | Open Source |
| *Mustela putorius* | Native | 57.159997 | 9.770644 | Open Source |
| *Mustela putorius* | Native | 57.130859 | 9.78457 | Open Source |
| *Mustela putorius* | Native | 55.845783 | 12.334042 | Open Source |
| *Mustela putorius* | Native | 55.878564 | 10.191212 | Open Source |
| *Mustela putorius* | Native | 55.917109 | 9.554949 | Open Source |
| *Mustela putorius* | Native | 56.14552 | 9.576066 | Open Source |
| *Mustela putorius* | Native | 56.462513 | 9.706408999 | Open Source |
| *Mustela putorius* | Native | 55.160913 | 9.506521 | Open Source |
| *Mustela putorius* | Native | 55.69709 | 8.236179001 | Open Source |
| *Mustela putorius* | Native | 56.973954 | 8.821109999 | Open Source |
| *Mustela putorius* | Native | 55.418444 | 12.225208 | Open Source |
| *Mustela putorius* | Native | 56.72582 | 10.010948 | Open Source |
| *Mustela putorius* | Native | 55.260974 | 11.963533 | Open Source |
| *Mustela putorius* | Native | 55.406994 | 11.725502 | Open Source |
| *Mustela putorius* | Native | 55.323616 | 11.724782 | Open Source |
| *Mustela putorius* | Native | 55.583196 | 11.560321 | Open Source |
| *Mustela putorius* | Native | 55.850413 | 9.62357 | Open Source |
| *Mustela putorius* | Native | 55.343204 | 8.674994 | Open Source |
| *Mustela putorius* | Native | 55.804299 | 12.093649 | Open Source |
| *Mustela putorius* | Native | 57.001323 | 10.117591 | Open Source |
| *Mustela putorius* | Native | 55.468783 | 8.591738 | Open Source |
| *Mustela putorius* | Native | 55.940596 | 8.536978 | Open Source |
| *Mustela putorius* | Native | 57.076419 | 10.100412 | Open Source |
| *Mustela putorius* | Native | 55.049454 | 10.685899 | Open Source |
| *Mustela putorius* | Native | 56.971681 | 9.871674 | Open Source |
| *Mustela putorius* | Native | 57.052589 | 8.86837 | Open Source |
| *Mustela putorius* | Native | 55.059692 | 10.648155 | Open Source |
| *Mustela putorius* | Native | 55.042237 | 11.987629 | Open Source |
| *Mustela putorius* | Native | 55.580604 | 10.172329 | Open Source |
| *Mustela putorius* | Native | 55.689101 | 11.680441 | Open Source |
| *Mustela putorius* | Native | 55.438443 | 11.790224 | Open Source |
| *Mustela putorius* | Native | 55.787216 | 8.806787001 | Open Source |
| *Mustela putorius* | Native | 56.918092 | 10.231876 | Open Source |
| *Mustela putorius* | Native | 54.909155 | 9.834185 | Open Source |
| *Mustela putorius* | Native | 55.590254 | 11.314116 | Open Source |
| *Mustela putorius* | Native | 57.019625 | 9.994369 | Open Source |
| *Mustela putorius* | Native | 55.90057 | 12.473881 | Open Source |
| *Mustela putorius* | Native | 44.653901 | 8.680539999 | Open Source |
| *Mustela putorius* | Native | 57.760953 | 16.182189 | Open Source |
| *Mustela putorius* | Native | 48.396965 | 1.992098 | Open Source |
| *Mustela putorius* | Native | 59.409272 | 13.593999 | Open Source |
| *Mustela putorius* | Native | 57.024525 | 12.321463 | Open Source |
| *Mustela putorius* | Native | 57.706911 | 12.957273 | Open Source |
| *Mustela putorius* | Native | 57.699994 | 12.053109 | Open Source |
| *Mustela putorius* | Native | 58.510425 | 13.170317 | Open Source |
| *Mustela putorius* | Native | 57.938899 | 11.834639 | Open Source |
| *Mustela putorius* | Native | 56.497633 | 15.601266 | Open Source |
| *Mustela putorius* | Native | 56.011519 | 13.164137 | Open Source |
| *Mustela putorius* | Native | 56.041909 | 13.096197 | Open Source |
| *Mustela putorius* | Native | 59.168322 | 15.372771 | Open Source |
| *Mustela putorius* | Native | 57.698885 | 12.054538 | Open Source |
| *Mustela putorius* | Native | 59.593127 | 16.25002 | Open Source |
| *Mustela putorius* | Native | 57.562488 | 15.127324 | Open Source |
| *Mustela putorius* | Native | 57.716887 | 14.587562 | Open Source |
| *Mustela putorius* | Native | 57.716887 | 14.587562 | Open Source |
| *Mustela putorius* | Native | 56.780576 | 15.885394 | Open Source |
| *Mustela putorius* | Native | 59.180774 | 15.340395 | Open Source |
| *Mustela putorius* | Native | 57.995684 | 13.559369 | Open Source |
| *Mustela putorius* | Native | 58.29822 | 13.600242 | Open Source |
| *Mustela putorius* | Native | 56.175193 | 13.68675 | Open Source |
| *Mustela putorius* | Native | 58.740966 | 17.178339 | Open Source |
| *Mustela putorius* | Native | 57.481632 | 11.946408 | Open Source |
| *Mustela putorius* | Native | 45.530672 | 8.171226 | Open Source |
| *Mustela putorius* | Native | 44.701769 | 8.790566 | Open Source |
| *Mustela putorius* | Native | 52.29113 | 8.831997 | Open Source |
| *Mustela putorius* | Native | 53.649063 | 10.911594 | Open Source |
| *Mustela putorius* | Native | 50.192123 | 10.267324 | Open Source |
| *Mustela putorius* | Native | 51.208927 | 6.085739 | Open Source |
| *Mustela putorius* | Native | 42.964714 | -2.627061001 | Open Source |
| *Mustela putorius* | Native | 61.566513 | 29.496827 | Open Source |
| *Mustela putorius* | Native | 49.813953 | 7.506666 | Open Source |
| *Mustela putorius* | Native | 50.063972 | 8.823395 | Open Source |
| *Mustela putorius* | Native | 51.318222 | 6.305722999 | Open Source |
| *Mustela putorius* | Native | 48.08519 | 9.631233 | Open Source |
| *Mustela putorius* | Native | 49.964638 | 10.183897 | Open Source |
| *Mustela putorius* | Native | 50.994946 | 11.364305 | Open Source |
| *Mustela putorius* | Native | 54.415134 | 9.249201 | Open Source |
| *Mustela putorius* | Native | 49.960884 | 10.050259 | Open Source |
| *Mustela putorius* | Native | 51.321903 | 6.246473001 | Open Source |
| *Mustela putorius* | Native | 48.070557 | 9.609999999 | Open Source |
| *Mustela putorius* | Native | 48.717842 | 9.357795 | Open Source |
| *Mustela putorius* | Native | 49.964638 | 10.183897 | Open Source |
| *Mustela putorius* | Native | 51.615708 | -1.049852 | Open Source |
| *Mustela putorius* | Native | 43.190436 | 11.141658 | Open Source |
| *Mustela putorius* | Native | 40.538303 | 16.643764 | Open Source |
| *Mustela putorius* | Native | 43.084928 | 10.985036 | Open Source |
| *Mustela putorius* | Native | 41.600266 | 14.264989 | Open Source |
| *Mustela putorius* | Native | 52.90901 | -3.86538 | Open Source |
| *Mustela putorius* | Native | 51.8594 | -3.234400001 | Open Source |
| *Mustela putorius* | Native | 51.6835 | -3.4862 | Open Source |
| *Mustela putorius* | Native | 52.58596 | -3.19034 | Open Source |
| *Mustela putorius* | Native | 53.50944 | -2.2661 | Open Source |
| *Mustela putorius* | Native | 55.77716 | -4.93135 | Open Source |
| *Mustela putorius* | Native | 52.911 | -1.86687 | Open Source |
| *Mustela putorius* | Native | 54.06429 | -1.967119999 | Open Source |
| *Mustela putorius* | Native | 52.56365 | -2.31786 | Open Source |
| *Mustela putorius* | Native | 52.49137 | -3.44996 | Open Source |
| *Mustela putorius* | Native | 53.36769 | -2.07285 | Open Source |
| *Mustela putorius* | Native | 50.64217 | -2.065 | Open Source |
| *Mustela putorius* | Native | 50.71141 | -2.535809999 | Open Source |
| *Mustela putorius* | Native | 52.05544 | 0.543059999 | Open Source |
| *Mustela putorius* | Native | 56.67272 | -2.4985 | Open Source |
| *Mustela putorius* | Native | 52.97918 | -2.39612 | Open Source |
| *Mustela putorius* | Native | 53.2632 | -1.756340001 | Open Source |
| *Mustela putorius* | Native | 54.462398 | -2.600011 | Open Source |
| *Mustela putorius* | Native | 53.50944 | -2.2661 | Open Source |
| *Mustela putorius* | Native | 52.6488 | -3.1492 | Open Source |
| *Mustela putorius* | Native | 52.33469 | -2.182669999 | Open Source |
| *Mustela putorius* | Native | 52.08842 | 0.11423 | Open Source |
| *Mustela putorius* | Native | 52.99665 | -2.4149 | Open Source |
| *Mustela putorius* | Native | 52.05598 | -2.190279999 | Open Source |
| *Mustela putorius* | Native | 54.4321 | -3.507899999 | Open Source |
| *Mustela putorius* | Native | 52.84076 | -3.003549999 | Open Source |
| *Mustela putorius* | Native | 52.38011 | -1.799377999 | Open Source |
| *Mustela putorius* | Native | 52.91908 | -2.1405 | Open Source |
| *Mustela putorius* | Native | 52.9587 | -1.921809999 | Open Source |
| *Mustela putorius* | Native | 51.76188 | -0.99438 | Open Source |
| *Mustela putorius* | Native | 52.82598 | -3.70746 | Open Source |
| *Mustela putorius* | Native | 54.5258 | -2.284600001 | Open Source |
| *Mustela putorius* | Native | 52.1972 | -0.0736 | Open Source |
| *Mustela putorius* | Native | 51.87943 | 0.45748 | Open Source |
| *Mustela putorius* | Native | 54.35986 | -2.789619999 | Open Source |
| *Mustela putorius* | Native | 51.30528 | -1.33505 | Open Source |
| *Mustela putorius* | Native | 52.81206 | -3.23508 | Open Source |
| *Mustela putorius* | Native | 52.03628 | -2.128970001 | Open Source |
| *Mustela putorius* | Native | 52.6194 | -0.574600001 | Open Source |
| *Mustela putorius* | Native | 51.0483 | -0.3426 | Open Source |
| *Mustela putorius* | Native | 54.64635 | -2.82042 | Open Source |
| *Mustela putorius* | Native | 51.76186 | 0.488769999 | Open Source |
| *Mustela putorius* | Native | 50.99131 | -0.70309 | Open Source |
| *Mustela putorius* | Native | 54.2463 | -2.88781 | Open Source |
| *Mustela putorius* | Native | 50.89971 | -0.3397 | Open Source |
| *Mustela putorius* | Native | 53.048185 | -2.345956 | Open Source |
| *Mustela putorius* | Native | 52.02511 | -2.56471 | Open Source |
| *Mustela putorius* | Native | 53.15224 | -3.834039999 | Open Source |
| *Mustela putorius* | Native | 52.5521 | -1.73523 | Open Source |
| *Mustela putorius* | Native | 52.8612 | -3.1103 | Open Source |
| *Mustela putorius* | Native | 52.5454 | -2.834590001 | Open Source |
| *Mustela putorius* | Native | 52.12386 | -1.00451 | Open Source |
| *Mustela putorius* | Native | 52.48338 | -2.76788 | Open Source |
| *Mustela putorius* | Native | 54.53 | -2.252000001 | Open Source |
| *Mustela putorius* | Native | 52.2167 | 1.95E-02 | Open Source |
| *Mustela putorius* | Native | 52.0454 | -2.91345 | Open Source |
| *Mustela putorius* | Native | 53.26023 | -4.43091 | Open Source |
| *Mustela putorius* | Native | 54.1113 | -2.76401 | Open Source |
| *Mustela putorius* | Native | 52.03143 | -3.171179999 | Open Source |
| *Mustela putorius* | Native | 52.09643 | -3.606459999 | Open Source |
| *Mustela putorius* | Native | 50.84136 | -2.460819999 | Open Source |
| *Mustela putorius* | Native | 51.896 | -2.15182 | Open Source |
| *Mustela putorius* | Native | 54.63979 | -1.17259 | Open Source |
| *Mustela putorius* | Native | 54.58152 | -3.555780001 | Open Source |
| *Mustela putorius* | Native | 51.86841 | 0.51063 | Open Source |
| *Mustela putorius* | Native | 52.65661 | -1.879470001 | Open Source |
| *Mustela putorius* | Native | 53.26592 | -3.552500001 | Open Source |
| *Mustela putorius* | Native | 50.9023 | -0.5395 | Open Source |
| *Mustela putorius* | Native | 52.79652 | -1.695190001 | Open Source |
| *Mustela putorius* | Native | 52.068 | 0.1679 | Open Source |
| *Mustela putorius* | Native | 51.45262 | -1.312769999 | Open Source |
| *Mustela putorius* | Native | 52.88545 | -3.63399 | Open Source |
| *Mustela putorius* | Native | 50.91946 | -1.811450001 | Open Source |
| *Mustela putorius* | Native | 52.69493 | -2.326140001 | Open Source |
| *Mustela putorius* | Native | 54.16712 | -2.750945 | Open Source |
| *Mustela putorius* | Native | 53.40526 | -0.94026 | Open Source |
| *Mustela putorius* | Native | 52.59323 | -2.126910001 | Open Source |
| *Mustela putorius* | Native | 52.02726 | -2.156629999 | Open Source |
| *Mustela putorius* | Native | 52.39245 | 1.284459999 | Open Source |
| *Mustela putorius* | Native | 50.79038 | -2.74711 | Open Source |
| *Mustela putorius* | Native | 51.67128 | -1.19812 | Open Source |
| *Mustela putorius* | Native | 54.57953 | -1.392730001 | Open Source |
| *Mustela putorius* | Native | 51.93344 | 0.36733 | Open Source |
| *Mustela putorius* | Native | 53.04851 | -2.4885 | Open Source |
| *Mustela putorius* | Native | 50.85756 | -1.994980001 | Open Source |
| *Mustela putorius* | Native | 50.75401 | -2.933439999 | Open Source |
| *Mustela putorius* | Native | 52.86703 | -3.66599 | Open Source |
| *Mustela putorius* | Native | 53.25058 | -2.258490001 | Open Source |
| *Mustela putorius* | Native | 52.67274 | -2.16781 | Open Source |
| *Mustela putorius* | Native | 52.82186 | -3.10699 | Open Source |
| *Mustela putorius* | Native | 52.2188 | -8.30E-03 | Open Source |
| *Mustela putorius* | Native | 53.28606 | -4.44287 | Open Source |
| *Mustela putorius* | Native | 51.93914 | -2.16651 | Open Source |
| *Mustela putorius* | Native | 50.81215 | -2.759239999 | Open Source |
| *Mustela putorius* | Native | 54.31744 | -2.63815 | Open Source |
| *Mustela putorius* | Native | 51.7076 | -1.7853 | Open Source |
| *Mustela putorius* | Native | 52.03659 | -2.39429 | Open Source |
| *Mustela putorius* | Native | 50.82168 | -2.786360001 | Open Source |
| *Mustela putorius* | Native | 51.12983 | -1.78487 | Open Source |
| *Mustela putorius* | Native | 52.0246 | -2.12165 | Open Source |
| *Mustela putorius* | Native | 52.66184 | -3.15396 | Open Source |
| *Mustela putorius* | Native | 52.1683 | 3.22E-02 | Open Source |
| *Mustela putorius* | Native | 52.74501 | -3.9443 | Open Source |
| *Mustela putorius* | Native | 52.944 | -3.625779999 | Open Source |
| *Mustela putorius* | Native | 50.6669 | -2.46335 | Open Source |
| *Mustela putorius* | Native | 52.7342 | -3.2159 | Open Source |
| *Mustela putorius* | Native | 53.0551 | -1.6062 | Open Source |
| *Mustela putorius* | Native | 52.05326 | -2.50235 | Open Source |
| *Mustela putorius* | Native | 52.04543 | 0.518829999 | Open Source |
| *Mustela putorius* | Native | 51.76188 | -0.99438 | Open Source |
| *Mustela putorius* | Native | 52.82048 | -3.71466 | Open Source |
| *Mustela putorius* | Native | 53.06484 | -2.27221 | Open Source |
| *Mustela putorius* | Native | 52.49219 | -3.455880001 | Open Source |
| *Mustela putorius* | Native | 52.2192 | -6.70E-03 | Open Source |
| *Mustela putorius* | Native | 52.93118 | -3.94073 | Open Source |
| *Mustela putorius* | Native | 52.91794 | -3.557870001 | Open Source |
| *Mustela putorius* | Native | 51.92149 | -0.29354 | Open Source |
| *Mustela putorius* | Native | 52.45684 | -2.836589999 | Open Source |
| *Mustela putorius* | Native | 54.93624 | -1.968679999 | Open Source |
| *Mustela putorius* | Native | 52.68935 | -1.180339999 | Open Source |
| *Mustela putorius* | Native | 52.12481 | 0.10872 | Open Source |
| *Mustela putorius* | Native | 52.10648 | -2.56355 | Open Source |
| *Mustela putorius* | Native | 52.14297 | -2.29586 | Open Source |
| *Mustela putorius* | Native | 52.81864 | -3.717559999 | Open Source |
| *Mustela putorius* | Native | 52.99122 | -1.57315 | Open Source |
| *Mustela putorius* | Native | 52.91773 | -3.57423 | Open Source |
| *Mustela putorius* | Native | 52.8146 | -2.6877 | Open Source |
| *Mustela putorius* | Native | 50.69572 | -2.45089 | Open Source |
| *Mustela putorius* | Native | 52.06542 | -2.37704 | Open Source |
| *Mustela putorius* | Native | 52.70117 | -1.99404 | Open Source |
| *Mustela putorius* | Native | 52.2189 | -7.50E-03 | Open Source |
| *Mustela putorius* | Native | 53.04851 | -2.4885 | Open Source |
| *Mustela putorius* | Native | 51.59185 | -3.066 | Open Source |
| *Mustela putorius* | Native | 52.31263 | -2.705440001 | Open Source |
| *Mustela putorius* | Native | 52.2424 | -2.25771 | Open Source |
| *Mustela putorius* | Native | 51.68459 | -2.22777 | Open Source |
| *Mustela putorius* | Native | 52.10724 | -2.373740001 | Open Source |
| *Mustela putorius* | Native | 52.0772 | 0.1934 | Open Source |
| *Mustela putorius* | Native | 56.50571 | -3.417519999 | Open Source |
| *Mustela putorius* | Native | 52.41632 | -2.27858 | Open Source |
| *Mustela putorius* | Native | 51.48334 | -3.5949 | Open Source |
| *Mustela putorius* | Native | 51.88716 | 0.43175 | Open Source |
| *Mustela putorius* | Native | 54.231984 | -3.180912001 | Open Source |
| *Mustela putorius* | Native | 53.082321 | -2.748491999 | Open Source |
| *Mustela putorius* | Native | 50.95602 | -1.53866 | Open Source |
| *Mustela putorius* | Native | 52.05992 | -2.6162 | Open Source |
| *Mustela putorius* | Native | 51.86885 | -3.15533 | Open Source |
| *Mustela putorius* | Native | 52.72741 | -2.840280001 | Open Source |
| *Mustela putorius* | Native | 52.8359 | -4.6809 | Open Source |
| *Mustela putorius* | Native | 52.0945 | -3.6124 | Open Source |
| *Mustela putorius* | Native | 52.84837 | -3.10841 | Open Source |
| *Mustela putorius* | Native | 52.99172 | -2.248 | Open Source |
| *Mustela putorius* | Native | 54.526 | -3.4475 | Open Source |
| *Mustela putorius* | Native | 52.65587 | -2.28971 | Open Source |
| *Mustela putorius* | Native | 52.21681 | -3.38E-03 | Open Source |
| *Mustela putorius* | Native | 52.72395 | -2.83999 | Open Source |
| *Mustela putorius* | Native | 50.90186 | -0.57002 | Open Source |
| *Mustela putorius* | Native | 52.62415 | -3.738 | Open Source |
| *Mustela putorius* | Native | 51.37511 | -2.550920001 | Open Source |
| *Mustela putorius* | Native | 52.44707 | -3.539729999 | Open Source |
| *Mustela putorius* | Native | 54.37878 | -3.323229999 | Open Source |
| *Mustela putorius* | Native | 52.58327 | -3.19027 | Open Source |
| *Mustela putorius* | Native | 54.90423 | -2.71969 | Open Source |
| *Mustela putorius* | Native | 53.15515 | -2.322220001 | Open Source |
| *Mustela putorius* | Native | 50.82496 | -1.769239999 | Open Source |
| *Mustela putorius* | Native | 52.8108 | -3.078399999 | Open Source |
| *Mustela putorius* | Native | 54.52385 | -3.44844 | Open Source |
| *Mustela putorius* | Native | 51.11026 | -3.21202 | Open Source |
| *Mustela putorius* | Native | 51.95221 | -0.01588 | Open Source |
| *Mustela putorius* | Native | 51.7693 | -1.8161 | Open Source |
| *Mustela putorius* | Native | 51.84061 | -3.09945 | Open Source |
| *Mustela putorius* | Native | 52.2888 | -4.29E-02 | Open Source |
| *Mustela putorius* | Native | 54.23315 | -3.02622 | Open Source |
| *Mustela putorius* | Native | 52.48816 | -3.340859999 | Open Source |
| *Mustela putorius* | Native | 51.2312 | -1.2379 | Open Source |
| *Mustela putorius* | Native | 54.27209 | -2.90672 | Open Source |
| *Mustela putorius* | Native | 52.97782 | -2.39983 | Open Source |
| *Mustela putorius* | Native | 52.8521 | -4.686 | Open Source |
| *Mustela putorius* | Native | 52.4508 | -1.0613 | Open Source |
| *Mustela putorius* | Native | 51.97564 | -4.58352 | Open Source |
| *Mustela putorius* | Native | 52.1844 | -4.48E-02 | Open Source |
| *Mustela putorius* | Native | 52.7482 | -2.7799 | Open Source |
| *Mustela putorius* | Native | 51.29648 | -1.291430001 | Open Source |
| *Mustela putorius* | Native | 51.86331 | 0.580069999 | Open Source |
| *Mustela putorius* | Native | 52.78834 | -3.76085 | Open Source |
| *Mustela putorius* | Native | 52.77306 | -4.09232 | Open Source |
| *Mustela putorius* | Native | 50.87577 | -0.30075 | Open Source |
| *Mustela putorius* | Native | 52.92064 | -3.92538 | Open Source |
| *Mustela putorius* | Native | 52.41477 | -2.42228 | Open Source |
| *Mustela putorius* | Native | 52.68094 | -1.95484 | Open Source |
| *Mustela putorius* | Native | 53.45904 | -0.922360001 | Open Source |
| *Mustela putorius* | Native | 50.77432 | -2.15914 | Open Source |
| *Mustela putorius* | Native | 52.7483 | -2.7453 | Open Source |
| *Mustela putorius* | Native | 52.93859 | -3.627069999 | Open Source |
| *Mustela putorius* | Native | 52.40259 | -1.631760001 | Open Source |
| *Mustela putorius* | Native | 52.53096 | -2.42377 | Open Source |
| *Mustela putorius* | Native | 51.86545 | -2.80814 | Open Source |
| *Mustela putorius* | Native | 51.8693 | -2.307899999 | Open Source |
| *Mustela putorius* | Native | 53.07256 | -0.833519999 | Open Source |
| *Mustela putorius* | Native | 52.14557 | 0.233860001 | Open Source |
| *Mustela putorius* | Native | 52.699 | -1.947999999 | Open Source |
| *Mustela putorius* | Native | 52.84089 | -2.98871 | Open Source |
| *Mustela putorius* | Native | 52.7565 | -2.91789 | Open Source |
| *Mustela putorius* | Native | 53.17611 | -3.579930001 | Open Source |
| *Mustela putorius* | Native | 51.85199 | -1.89179 | Open Source |
| *Mustela putorius* | Native | 51.96161 | -0.36407 | Open Source |
| *Mustela putorius* | Native | 50.89973 | -0.34112 | Open Source |
| *Mustela putorius* | Native | 53.536 | -2.7147 | Open Source |
| *Mustela putorius* | Native | 53.2448 | -2.254 | Open Source |
| *Mustela putorius* | Native | 52.8115 | -2.61491 | Open Source |
| *Mustela putorius* | Native | 50.78696 | -2.73329 | Open Source |
| *Mustela putorius* | Native | 51.05798 | -1.5141 | Open Source |
| *Mustela putorius* | Native | 50.85042 | -0.62984 | Open Source |
| *Mustela putorius* | Native | 53.25596 | -2.26452 | Open Source |
| *Mustela putorius* | Native | 53.17762 | -2.729360001 | Open Source |
| *Mustela putorius* | Native | 52.04543 | 0.518829999 | Open Source |
| *Mustela putorius* | Native | 52.0904 | -3.1919 | Open Source |
| *Mustela putorius* | Native | 56.73922 | -2.682469999 | Open Source |
| *Mustela putorius* | Native | 53.1264 | -2.6787 | Open Source |
| *Mustela putorius* | Native | 51.61417 | -2.73579 | Open Source |
| *Mustela putorius* | Native | 51.84156 | 0.50478 | Open Source |
| *Mustela putorius* | Native | 52.10199 | -1.53935 | Open Source |
| *Mustela putorius* | Native | 52.90467 | -3.609439999 | Open Source |
| *Mustela putorius* | Native | 52.7703 | -2.35939 | Open Source |
| *Mustela putorius* | Native | 51.15249 | -1.34726 | Open Source |
| *Mustela putorius* | Native | 53.05252 | -2.373669999 | Open Source |
| *Mustela putorius* | Native | 52.90113 | -2.510550001 | Open Source |
| *Mustela putorius* | Native | 51.95968 | -2.42032 | Open Source |
| *Mustela putorius* | Native | 52.98286 | -3.397840001 | Open Source |
| *Mustela putorius* | Native | 52.6553 | -3.1761 | Open Source |
| *Mustela putorius* | Native | 51.77517 | -2.023139999 | Open Source |
| *Mustela putorius* | Native | 52.94548 | -3.51421 | Open Source |
| *Mustela putorius* | Native | 53.05783 | -2.39908 | Open Source |
| *Mustela putorius* | Native | 53.19186 | -3.19209 | Open Source |
| *Mustela putorius* | Native | 54.52385 | -3.44844 | Open Source |
| *Mustela putorius* | Native | 52.87928 | -3.62485 | Open Source |
| *Mustela putorius* | Native | 52.8325 | -2.6791 | Open Source |
| *Mustela putorius* | Native | 52.09338 | -2.57725 | Open Source |
| *Mustela putorius* | Native | 52.45707 | 0.8811 | Open Source |
| *Mustela putorius* | Native | 52.6551 | -3.177300001 | Open Source |
| *Mustela putorius* | Native | 52.37111 | -3.880820001 | Open Source |
| *Mustela putorius* | Native | 53.71731 | -2.665930001 | Open Source |
| *Mustela putorius* | Native | 52.8521 | -4.686 | Open Source |
| *Mustela putorius* | Native | 52.9412 | -3.63312 | Open Source |
| *Mustela putorius* | Native | 52.31882 | -2.4144 | Open Source |
| *Mustela putorius* | Native | 52.65701 | -2.36437 | Open Source |
| *Mustela putorius* | Native | 51.6835 | -3.4862 | Open Source |
| *Mustela putorius* | Native | 53.04872 | -2.78536 | Open Source |
| *Mustela putorius* | Native | 51.99618 | 0.878859999 | Open Source |
| *Mustela putorius* | Native | 50.76666 | -2.4771 | Open Source |
| *Mustela putorius* | Native | 51.98827 | -2.795060001 | Open Source |
| *Mustela putorius* | Native | 54.5472 | -2.4093 | Open Source |
| *Mustela putorius* | Native | 52.90405 | -2.860569999 | Open Source |
| *Mustela putorius* | Native | 52.03802 | -2.49198 | Open Source |
| *Mustela putorius* | Native | 52.56556 | -1.72482 | Open Source |
| *Mustela putorius* | Native | 51.52297 | -1.67709 | Open Source |
| *Mustela putorius* | Native | 53.14026 | -3.794649999 | Open Source |
| *Mustela putorius* | Native | 51.64567 | -3.26094 | Open Source |
| *Mustela putorius* | Native | 53.1278 | -0.767900001 | Open Source |
| *Mustela putorius* | Native | 52.41017 | -2.20503 | Open Source |
| *Mustela putorius* | Native | 52.3211 | -1.547199999 | Open Source |
| *Mustela putorius* | Native | 52.75182 | -2.88298 | Open Source |
| *Mustela putorius* | Native | 52.90663 | -3.90246 | Open Source |
| *Mustela putorius* | Native | 51.9605 | -2.707980001 | Open Source |
| *Mustela putorius* | Native | 50.96461 | -1.475850001 | Open Source |
| *Mustela putorius* | Native | 52.6627 | -3.352499999 | Open Source |
| *Mustela putorius* | Native | 52.467 | -2.92069 | Open Source |
| *Mustela putorius* | Native | 52.03073 | -0.47285 | Open Source |
| *Mustela putorius* | Native | 51.86515 | -2.298390001 | Open Source |
| *Mustela putorius* | Native | 57.65169 | -7.4816 | Open Source |
| *Mustela putorius* | Native | 57.43162 | -7.388489999 | Open Source |
| *Mustela putorius* | Native | 57.45968 | -7.33536 | Open Source |
| *Mustela putorius* | Native | 57.42772 | -7.37457 | Open Source |
| *Mustela putorius* | Native | 57.16918 | -7.401499999 | Open Source |
| *Mustela putorius* | Native | 57.4352 | -7.38902 | Open Source |
| *Mustela putorius* | Native | 57.13915 | -7.36731 | Open Source |
| *Mustela putorius* | Native | 58.46959 | -6.29769 | Open Source |
| *Mustela putorius* | Native | 57.39898 | -7.35201 | Open Source |
| *Mustela putorius* | Native | 57.45534 | -7.402010001 | Open Source |
| *Mustela putorius* | Native | 57.42951 | -7.374829999 | Open Source |
| *Mustela putorius* | Native | 57.24069 | -7.433559999 | Open Source |
| *Mustela putorius* | Native | 57.43094 | -7.383380001 | Open Source |
| *Mustela putorius* | Native | 57.38595 | -7.4251 | Open Source |
| *Mustela putorius* | Native | 57.33083 | -7.377839999 | Open Source |
| *Mustela putorius* | Native | 57.35556 | -7.35733 | Open Source |
| *Mustela putorius* | Native | 58.4976 | -6.26676 | Open Source |
| *Mustela putorius* | Native | 57.50171 | -7.263439999 | Open Source |
| *Mustela putorius* | Native | 57.16972 | -7.40986 | Open Source |
| *Mustela putorius* | Native | 57.33978 | -7.37915 | Open Source |
| *Mustela putorius* | Native | 52.52962 | -2.421540001 | Open Source |
| *Mustela putorius* | Native | 52.98882 | -2.05359 | Open Source |
| *Mustela putorius* | Native | 52.63056 | -1.33879 | Open Source |
| *Mustela putorius* | Native | 52.67338 | -0.66078 | Open Source |
| *Mustela putorius* | Native | 52.4969 | -1.23769 | Open Source |
| *Mustela putorius* | Native | 52.6292 | -1.01377 | Open Source |
| *Mustela putorius* | Native | 52.5321 | -1.25919 | Open Source |
| *Mustela putorius* | Native | 52.75888 | -0.66853 | Open Source |
| *Mustela putorius* | Native | 57.676 | -2.71339 | Open Source |
| *Mustela putorius* | Native | 57.19913 | -2.50226 | Open Source |
| *Mustela putorius* | Native | 57.67412 | -2.91447 | Open Source |
| *Mustela putorius* | Native | 57.0902 | -3.89113 | Open Source |
| *Mustela putorius* | Native | 51.6336 | -4.97591 | Open Source |
| *Mustela putorius* | Native | 51.36069 | -2.453799999 | Open Source |
| *Mustela putorius* | Native | 51.49591 | -2.35431 | Open Source |
| *Mustela putorius* | Native | 51.33387 | -2.410480001 | Open Source |
| *Mustela putorius* | Native | 51.55762 | -2.64328 | Open Source |
| *Mustela putorius* | Native | 51.50343 | -2.685740001 | Open Source |
| *Mustela putorius* | Native | 51.55754 | -2.6577 | Open Source |
| *Mustela putorius* | Native | 51.35023 | -2.74089 | Open Source |
| *Mustela putorius* | Native | 51.30595 | -2.62541 | Open Source |
| *Mustela putorius* | Native | 51.85073 | 0.227950001 | Open Source |
| *Mustela putorius* | Native | 51.85073 | 0.227950001 | Open Source |
| *Mustela putorius* | Native | 58.24198 | -3.44526 | Open Source |
| *Mustela putorius* | Native | 58.57177 | -4.75553 | Open Source |
| *Mustela putorius* | Native | 57.96185 | -4.027250001 | Open Source |
| *Mustela putorius* | Native | 58.56353 | -4.72049 | Open Source |
| *Mustela putorius* | Native | 58.2695 | -3.39353 | Open Source |
| *Mustela putorius* | Native | 58.56443 | -4.720559999 | Open Source |
| *Mustela putorius* | Native | 58.22664 | -3.45144 | Open Source |
| *Mustela putorius* | Native | 58.26092 | -3.515910001 | Open Source |
| *Mustela putorius* | Native | 57.98751 | -3.937360001 | Open Source |
| *Mustela putorius* | Native | 58.29378 | -3.391069999 | Open Source |
| *Mustela putorius* | Native | 58.35835 | -3.40045 | Open Source |
| *Mustela putorius* | Native | 58.54262 | -3.46964 | Open Source |
| *Mustela putorius* | Native | 58.47389 | -3.0604 | Open Source |
| *Mustela putorius* | Native | 58.56837 | -4.746670001 | Open Source |
| *Mustela putorius* | Native | 58.51868 | -3.36091 | Open Source |
| *Mustela putorius* | Native | 58.57514 | -4.766119999 | Open Source |
| *Mustela putorius* | Native | 58.51543 | -3.144930001 | Open Source |
| *Mustela putorius* | Native | 58.56449 | -4.76012 | Open Source |
| *Mustela putorius* | Native | 57.16918 | -7.401499999 | Open Source |
| *Mustela putorius* | Native | 59.546824 | 16.504504 | Open Source |
| *Mustela putorius* | Native | 60.010298 | 13.141203 | Open Source |
| *Mustela putorius* | Native | 59.354639 | 12.959114 | Open Source |
| *Mustela putorius* | Native | 58.162384 | 13.489165 | Open Source |
| *Mustela putorius* | Native | 59.027116 | 15.028554 | Open Source |
| *Mustela putorius* | Native | 58.863155 | 11.492049 | Open Source |
| *Mustela putorius* | Native | 56.795608 | 13.149045 | Open Source |
| *Mustela putorius* | Native | 55.846845 | 13.323444 | Open Source |
| *Mustela putorius* | Native | 59.339997 | 16.489206 | Open Source |
| *Mustela putorius* | Native | 57.709267 | 11.740936 | Open Source |
| *Mustela putorius* | Native | 56.62414 | 13.7614 | Open Source |
| *Mustela putorius* | Native | 58.67053 | 13.633237 | Open Source |
| *Mustela putorius* | Native | 56.202806 | 13.752425 | Open Source |
| *Mustela putorius* | Native | 56.202806 | 13.752425 | Open Source |
| *Mustela putorius* | Native | 57.346502 | 12.553678 | Open Source |
| *Mustela putorius* | Native | 59.793858 | 16.194828 | Open Source |
| *Mustela putorius* | Native | 57.536295 | 12.41513 | Open Source |
| *Mustela putorius* | Native | 59.205636 | 17.017092 | Open Source |
| *Mustela putorius* | Native | 59.421092 | 14.007107 | Open Source |
| *Mustela putorius* | Native | 56.027726 | 13.068466 | Open Source |
| *Mustela putorius* | Native | 55.874593 | 14.178514 | Open Source |
| *Mustela putorius* | Native | 59.138461 | 14.416846 | Open Source |
| *Mustela putorius* | Native | 58.0543 | 12.762581 | Open Source |
| *Mustela putorius* | Native | 55.594267 | 13.766951 | Open Source |
| *Mustela putorius* | Native | 57.002942 | 14.528893 | Open Source |
| *Mustela putorius* | Native | 50.919845 | 4.338602 | Open Source |
| *Mustela putorius* | Native | 51.201 | 3.521 | Open Source |
| *Mustela putorius* | Native | 44.688549 | 8.418102001 | Open Source |
| *Mustela putorius* | Native | 56.792647 | 15.945959 | Open Source |
| *Mustela putorius* | Native | 57.839678 | 11.964032 | Open Source |
| *Mustela putorius* | Native | 59.553329 | 13.475214 | Open Source |
| *Mustela putorius* | Native | 59.188252 | 12.559867 | Open Source |
| *Mustela putorius* | Native | 56.698881 | 16.137542 | Open Source |
| *Mustela putorius* | Native | 56.268065 | 14.888776 | Open Source |
| *Mustela putorius* | Native | 56.808534 | 12.958036 | Open Source |
| *Mustela putorius* | Native | 59.199463 | 12.219307 | Open Source |
| *Mustela putorius* | Native | 55.644547 | 14.120825 | Open Source |
| *Mustela putorius* | Native | 56.593394 | 15.682501 | Open Source |
| *Mustela putorius* | Native | 55.92634 | 13.796191 | Open Source |
| *Mustela putorius* | Native | 58.46535 | 15.563303 | Open Source |
| *Mustela putorius* | Native | 57.134704 | 12.250092 | Open Source |
| *Mustela putorius* | Native | 58.111947 | 13.476777 | Open Source |
| *Mustela putorius* | Native | 55.635218 | 13.05509 | Open Source |
| *Mustela putorius* | Native | 58.395454 | 12.295744 | Open Source |
| *Mustela putorius* | Native | 55.668923 | 13.102081 | Open Source |
| *Mustela putorius* | Native | 57.929747 | 13.554024 | Open Source |
| *Mustela putorius* | Native | 58.621101 | 16.094639 | Open Source |
| *Mustela putorius* | Native | 58.620259 | 16.093678 | Open Source |
| *Mustela putorius* | Native | 55.69497 | 13.214919 | Open Source |
| *Mustela putorius* | Native | 55.775832 | 13.797835 | Open Source |
| *Mustela putorius* | Native | 58.67053 | 13.633237 | Open Source |
| *Mustela putorius* | Native | 59.683599 | 17.375574 | Open Source |
| *Mustela putorius* | Native | 57.36713 | 12.331407 | Open Source |
| *Mustela putorius* | Native | 58.413635 | 12.023797 | Open Source |
| *Mustela putorius* | Native | 55.376344 | 13.092505 | Open Source |
| *Mustela putorius* | Native | 57.065206 | 12.278137 | Open Source |
| *Mustela putorius* | Native | 55.453885 | 12.989396 | Open Source |
| *Mustela putorius* | Native | 55.56511 | 13.139083 | Open Source |
| *Mustela putorius* | Native | 57.736428 | 11.782923 | Open Source |
| *Mustela putorius* | Native | 57.642092 | 16.292583 | Open Source |
| *Mustela putorius* | Native | 57.832605 | 13.057803 | Open Source |
| *Mustela putorius* | Native | 57.832605 | 13.057803 | Open Source |
| *Mustela putorius* | Native | 58.566102 | 16.112255 | Open Source |
| *Mustela putorius* | Native | 56.962687 | 14.397344 | Open Source |
| *Mustela putorius* | Native | 58.282426 | 13.571011 | Open Source |
| *Mustela putorius* | Native | 56.726156 | 15.976259 | Open Source |
| *Mustela putorius* | Native | 59.436676 | 13.416482 | Open Source |
| *Mustela putorius* | Native | 55.553338 | 13.808543 | Open Source |
| *Mustela putorius* | Native | 58.976285 | 16.226242 | Open Source |
| *Mustela putorius* | Native | 56.679754 | 14.519578 | Open Source |
| *Mustela putorius* | Native | 55.774903 | 13.161056 | Open Source |
| *Mustela putorius* | Native | 55.989592 | 14.074948 | Open Source |
| *Mustela putorius* | Native | 58.968793 | 16.204979 | Open Source |
| *Mustela putorius* | Native | 56.28196 | 12.530178 | Open Source |
| *Mustela putorius* | Native | 57.194714 | 15.156744 | Open Source |
| *Mustela putorius* | Native | 58.776216 | 17.08239 | Open Source |
| *Mustela putorius* | Native | 58.598688 | 16.098232 | Open Source |
| *Mustela putorius* | Native | 57.965154 | 12.463199 | Open Source |
| *Mustela putorius* | Native | 58.671319 | 16.403596 | Open Source |
| *Mustela putorius* | Native | 59.32665 | 14.982992 | Open Source |
| *Mustela putorius* | Native | 55.574461 | 13.738825 | Open Source |
| *Mustela putorius* | Native | 55.574461 | 13.738825 | Open Source |
| *Mustela putorius* | Native | 57.354528 | 14.100523 | Open Source |
| *Mustela putorius* | Native | 59.705946 | 14.54023 | Open Source |
| *Mustela putorius* | Native | 56.610043 | 16.226646 | Open Source |
| *Mustela putorius* | Native | 60.089038 | 13.026766 | Open Source |
| *Mustela putorius* | Native | 57.999488 | 15.438411 | Open Source |
| *Mustela putorius* | Native | 59.178044 | 10.967846 | Open Source |
| *Mustela putorius* | Native | 60.507627 | 5.091121001 | Open Source |
| *Mustela putorius* | Native | 54.489166 | 9.850170001 | Open Source |
| *Mustela putorius* | Native | 50.097954 | 10.164328 | Open Source |
| *Mustela putorius* | Native | 42.842922 | 20.729797 | Open Source |
| *Mustela putorius* | Native | 53.280643 | 10.800856 | Open Source |
| *Mustela putorius* | Native | 53.701889 | 10.277538 | Open Source |
| *Mustela putorius* | Native | 50.172722 | 10.298395 | Open Source |
| *Mustela putorius* | Native | 52.023647 | 13.039991 | Open Source |
| *Mustela putorius* | Native | 53.229572 | 8.983125999 | Open Source |
| *Mustela putorius* | Native | 54.143486 | 10.507849 | Open Source |
| *Mustela putorius* | Native | 50.506821 | 9.116807 | Open Source |
| *Mustela putorius* | Native | 48.725628 | 9.325521 | Open Source |
| *Mustela putorius* | Native | 49.823868 | 7.457521 | Open Source |
| *Mustela putorius* | Native | 51.818378 | 7.178192001 | Open Source |
| *Mustela putorius* | Native | 53.027863 | 14.196902 | Open Source |
| *Mustela putorius* | Native | 54.164543 | 10.571079 | Open Source |
| *Mustela putorius* | Native | 53.660877 | 10.036783 | Open Source |
| *Mustela putorius* | Native | 53.19722 | 8.243968001 | Open Source |
| *Mustela putorius* | Native | 52.160145 | 8.247341999 | Open Source |
| *Mustela putorius* | Native | 49.304672 | 8.557706001 | Open Source |
| *Mustela putorius* | Native | 50.023853 | 9.707190001 | Open Source |
| *Mustela putorius* | Native | 50.83913 | 6.431936999 | Open Source |
| *Mustela putorius* | Native | 53.423458 | 8.540668001 | Open Source |
| *Mustela putorius* | Native | 52.471382 | 8.317165 | Open Source |
| *Mustela putorius* | Native | 50.704994 | 6.627716999 | Open Source |
| *Mustela putorius* | Native | 52.986515 | 10.592151 | Open Source |
| *Mustela putorius* | Native | 53.657051 | 10.199947 | Open Source |
| *Mustela putorius* | Native | 51.689053 | 9.044065 | Open Source |
| *Mustela putorius* | Native | 58.030016 | 24.88687 | Open Source |
| *Mustela putorius* | Native | 42.505854 | 12.858683 | Open Source |
| *Mustela putorius* | Native | 58.266747 | 24.64739 | Open Source |
| *Mustela putorius* | Native | 40.229428 | 16.633301 | Open Source |
| *Mustela putorius* | Native | 45.144287 | 7.935998 | Open Source |
| *Mustela putorius* | Native | 43.10932 | 12.3037 | Open Source |
| *Mustela putorius* | Native | 59.220708 | 10.978563 | Open Source |
| *Mustela putorius* | Native | 60.911629 | 11.637216 | Open Source |
| *Mustela putorius* | Native | 59.133152 | 11.375672 | Open Source |
| *Mustela putorius* | Native | 59.449368 | 10.859069 | Open Source |
| *Mustela putorius* | Native | 59.282417 | 11.282436 | Open Source |
| *Mustela putorius* | Native | 59.59365 | 11.087681 | Open Source |
| *Mustela putorius* | Native | 59.720543 | 11.492013 | Open Source |
| *Mustela putorius* | Native | 59.852145 | 10.671069 | Open Source |
| *Mustela putorius* | Native | 59.852145 | 10.671069 | Open Source |
| *Mustela putorius* | Native | 60.218179 | 12.034883 | Open Source |
| *Mustela putorius* | Native | 59.720385 | 11.492399 | Open Source |
| *Mustela putorius* | Native | 59.09049 | 11.399846 | Open Source |
| *Mustela putorius* | Native | 58.5474 | 12.341145 | Open Source |
| *Mustela putorius* | Native | 56.116663 | 13.846901 | Open Source |
| *Mustela putorius* | Native | 58.798542 | 12.425515 | Open Source |
| *Mustela putorius* | Native | 58.318929 | 12.048762 | Open Source |
| *Mustela putorius* | Native | 58.259051 | 13.529994 | Open Source |
| *Mustela putorius* | Native | 57.50714 | 12.676361 | Open Source |
| *Mustela putorius* | Native | 58.020385 | 13.564938 | Open Source |
| *Mustela putorius* | Native | 57.758806 | 12.979254 | Open Source |
| *Mustela putorius* | Native | 57.836211 | 12.307665 | Open Source |
| *Mustela putorius* | Native | 56.453498 | 15.987371 | Open Source |
| *Mustela putorius* | Native | 55.781571 | 13.781522 | Open Source |
| *Mustela putorius* | Native | 57.127245 | 15.111954 | Open Source |
| *Mustela putorius* | Native | 57.169294 | 12.538298 | Open Source |
| *Mustela putorius* | Native | 58.67053 | 13.633237 | Open Source |
| *Mustela putorius* | Native | 59.467235 | 12.773612 | Open Source |
| *Mustela putorius* | Native | 56.100803 | 14.113342 | Open Source |
| *Mustela putorius* | Native | 59.524899 | 15.963188 | Open Source |
| *Mustela putorius* | Native | 55.539047 | 14.007942 | Open Source |
| *Mustela putorius* | Native | 55.717944 | 13.266239 | Open Source |
| *Mustela putorius* | Native | 59.299579 | 15.214901 | Open Source |
| *Mustela putorius* | Native | 43.085646 | 10.982144 | Open Source |
| *Mustela putorius* | Native | 55.577822 | 13.088301 | Open Source |
| *Mustela putorius* | Native | 57.08276 | 12.282476 | Open Source |
| *Mustela putorius* | Native | 59.840919 | 13.136019 | Open Source |
| *Mustela putorius* | Native | 59.90084 | 13.171728 | Open Source |
| *Mustela putorius* | Native | 56.843277 | 15.980697 | Open Source |
| *Mustela putorius* | Native | 55.644547 | 14.120825 | Open Source |
| *Mustela putorius* | Native | 56.773351 | 12.784239 | Open Source |
| *Mustela putorius* | Native | 58.071526 | 15.206591 | Open Source |
| *Mustela putorius* | Native | 53.070889 | 8.597553 | Open Source |
| *Mustela putorius* | Native | 53.598209 | 8.684692001 | Open Source |
| *Mustela putorius* | Native | 51.622921 | 6.426486999 | Open Source |
| *Mustela putorius* | Native | 50.398342 | 8.89781 | Open Source |
| *Mustela putorius* | Native | 53.402779 | 8.514404001 | Open Source |
| *Mustela putorius* | Native | 53.036312 | 10.45804 | Open Source |
| *Mustela putorius* | Native | 53.708496 | 10.313416 | Open Source |
| *Mustela putorius* | Native | 52.671803 | 8.657742 | Open Source |
| *Mustela putorius* | Native | 50.028694 | 10.220032 | Open Source |
| *Mustela putorius* | Native | 53.724442 | 10.178318 | Open Source |
| *Mustela putorius* | Native | 58.958104 | 13.127 | Open Source |
| *Mustela putorius* | Native | 57.847003 | 12.676136 | Open Source |
| *Mustela putorius* | Native | 56.740584 | 12.988363 | Open Source |
| *Mustela putorius* | Native | 57.304921 | 13.931154 | Open Source |
| *Mustela putorius* | Native | 58.19544 | 13.970006 | Open Source |
| *Mustela putorius* | Native | 58.19544 | 13.970006 | Open Source |
| *Mustela putorius* | Native | 56.823417 | 15.879097 | Open Source |
| *Mustela putorius* | Native | 55.645064 | 13.705848 | Open Source |
| *Mustela putorius* | Native | 57.474388 | 12.391702 | Open Source |
| *Mustela putorius* | Native | 59.108408 | 15.065286 | Open Source |
| *Mustela putorius* | Native | 58.434384 | 12.193076 | Open Source |
| *Mustela putorius* | Native | 59.1606 | 16.923056 | Open Source |
| *Mustela putorius* | Native | 55.913388 | 13.449163 | Open Source |
| *Mustela putorius* | Native | 56.05253 | 12.773028 | Open Source |
| *Mustela putorius* | Native | 55.828811 | 14.087569 | Open Source |
| *Mustela putorius* | Native | 50.948696 | 6.756935 | Open Source |
| *Mustela putorius* | Native | 50.764984 | 8.962902 | Open Source |
| *Mustela putorius* | Native | 50.541798 | 10.360794 | Open Source |
| *Mustela putorius* | Native | 52.499268 | 9.359407 | Open Source |
| *Mustela putorius* | Native | 49.999989 | 12.064233 | Open Source |
| *Mustela putorius* | Native | 51.781689 | 9.907644999 | Open Source |
| *Mustela putorius* | Native | 48.593002 | 10.619371 | Open Source |
| *Mustela putorius* | Native | 50.763058 | 8.942624 | Open Source |
| *Mustela putorius* | Native | 50.764259 | 6.436701 | Open Source |
| *Mustela putorius* | Native | 52.001423 | 10.407572 | Open Source |
| *Mustela putorius* | Native | 53.201233 | 9.200271 | Open Source |
| *Mustela putorius* | Native | 54.228165 | 10.578032 | Open Source |
| *Mustela putorius* | Native | 53.673431 | 8.540665999 | Open Source |
| *Mustela putorius* | Native | 47.606121 | 10.007413 | Open Source |
| *Mustela putorius* | Native | 51.242577 | 6.883621001 | Open Source |
| *Mustela putorius* | Native | 52.52924 | 9.475308 | Open Source |
| *Mustela putorius* | Native | 53.652168 | 8.779964 | Open Source |
| *Mustela putorius* | Native | 50.87093 | 8.822143 | Open Source |
| *Mustela putorius* | Native | 54.270855 | 10.255968 | Open Source |
| *Mustela putorius* | Native | 50.40633 | 8.92828 | Open Source |
| *Mustela putorius* | Native | 53.623436 | 8.540667 | Open Source |
| *Mustela putorius* | Native | 53.027863 | 14.196902 | Open Source |
| *Mustela putorius* | Native | 51.781689 | 9.907644999 | Open Source |
| *Mustela putorius* | Native | 51.359669 | 9.054623 | Open Source |
| *Mustela putorius* | Native | 53.423458 | 8.540668001 | Open Source |
| *Mustela putorius* | Native | 51.268147 | 6.413269 | Open Source |
| *Mustela putorius* | Native | 54.291374 | 10.208761 | Open Source |
| *Mustela putorius* | Native | 47.565872 | 10.251446 | Open Source |
| *Mustela putorius* | Native | 53.758408 | 10.031881 | Open Source |
| *Mustela putorius* | Native | 50.457615 | 8.269615 | Open Source |
| *Mustela putorius* | Native | 53.017418 | 11.473074 | Open Source |
| *Mustela putorius* | Native | 50.149666 | 10.757299 | Open Source |
| *Mustela putorius* | Native | 50.968639 | 6.869105 | Open Source |
| *Mustela putorius* | Native | 50.191956 | 10.18364 | Open Source |
| *Mustela putorius* | Native | 53.223484 | 8.62399 | Open Source |
| *Mustela putorius* | Native | 53.056637 | 11.240625 | Open Source |
| *Mustela putorius* | Native | 53.201233 | 9.200271 | Open Source |
| *Mustela putorius* | Native | 49.645672 | 8.458958 | Open Source |
| *Mustela putorius* | Native | 54.292583 | 10.493488 | Open Source |
| *Mustela putorius* | Native | 54.75246 | 9.900066001 | Open Source |
| *Mustela putorius* | Native | 52.705692 | 9.787241001 | Open Source |
| *Mustela putorius* | Native | 50.190006 | 10.300283 | Open Source |
| *Mustela putorius* | Native | 53.88871 | 10.796556 | Open Source |
| *Mustela putorius* | Native | 50.526821 | 9.065427 | Open Source |
| *Mustela putorius* | Native | 52.454857 | 9.269542999 | Open Source |
| *Mustela putorius* | Native | 53.533779 | 8.714560999 | Open Source |
| *Mustela putorius* | Native | 54.383369 | 10.236559 | Open Source |
| *Mustela putorius* | Native | 53.166946 | 10.391006 | Open Source |
| *Mustela putorius* | Native | 50.991711 | 6.822599 | Open Source |
| *Mustela putorius* | Native | 49.022694 | 7.807314999 | Open Source |
| *Mustela putorius* | Native | 54.298969 | 10.53493 | Open Source |
| *Mustela putorius* | Native | 52.503944 | 9.463379001 | Open Source |
| *Mustela putorius* | Native | 53.013439 | 8.805113 | Open Source |
| *Mustela putorius* | Native | 51.616871 | 6.462879 | Open Source |
| *Mustela putorius* | Native | 50.658539 | 8.64428 | Open Source |
| *Mustela putorius* | Native | 49.960167 | 8.522300999 | Open Source |
| *Mustela putorius* | Native | 59.421092 | 14.007107 | Open Source |
| *Mustela putorius* | Native | 57.671739 | 11.980696 | Open Source |
| *Mustela putorius* | Native | 56.411015 | 12.746106 | Open Source |
| *Mustela putorius* | Native | 57.937354 | 12.504639 | Open Source |
| *Mustela putorius* | Native | 57.459977 | 12.384875 | Open Source |
| *Mustela putorius* | Native | 56.901232 | 14.799654 | Open Source |
| *Mustela putorius* | Native | 56.800213 | 12.890546 | Open Source |
| *Mustela putorius* | Native | 58.478803 | 12.40277 | Open Source |
| *Mustela putorius* | Native | 58.481363 | 12.140911 | Open Source |
| *Mustela putorius* | Native | 58.954958 | 12.622183 | Open Source |
| *Mustela putorius* | Native | 56.467688 | 15.009941 | Open Source |
| *Mustela putorius* | Native | 56.778352 | 12.775229 | Open Source |
| *Mustela putorius* | Native | 59.338577 | 16.252706 | Open Source |
| *Mustela putorius* | Native | 58.29603 | 13.071467 | Open Source |
| *Mustela putorius* | Native | 59.353984 | 13.958283 | Open Source |
| *Mustela putorius* | Native | 56.203721 | 15.010633 | Open Source |
| *Mustela putorius* | Native | 55.555915 | 13.113436 | Open Source |
| *Mustela putorius* | Native | 56.823417 | 15.879097 | Open Source |
| *Mustela putorius* | Native | 55.684119 | 13.179058 | Open Source |
| *Mustela putorius* | Native | 56.248481 | 14.829658 | Open Source |
| *Mustela putorius* | Native | 56.898298 | 14.679981 | Open Source |
| *Mustela putorius* | Native | 56.904209 | 14.659598 | Open Source |
| *Mustela putorius* | Native | 56.968544 | 14.969526 | Open Source |
| *Mustela putorius* | Native | 56.968544 | 14.969526 | Open Source |
| *Mustela putorius* | Native | 55.86422 | 13.650619 | Open Source |
| *Mustela putorius* | Native | 56.325778 | 14.910156 | Open Source |
| *Mustela putorius* | Native | 56.390002 | 14.905961 | Open Source |
| *Mustela putorius* | Native | 56.089739 | 13.227675 | Open Source |
| *Mustela putorius* | Native | 59.518246 | 14.982309 | Open Source |
| *Mustela putorius* | Native | 59.279925 | 14.916148 | Open Source |
| *Mustela putorius* | Native | 56.869135 | 13.952534 | Open Source |
| *Mustela putorius* | Native | 59.210248 | 14.07024 | Open Source |
| *Mustela putorius* | Native | 57.893092 | 13.523005 | Open Source |
| *Mustela putorius* | Native | 59.022215 | 15.676159 | Open Source |
| *Mustela putorius* | Native | 57.06966 | 14.930485 | Open Source |
| *Mustela putorius* | Native | 56.017951 | 14.158087 | Open Source |
| *Mustela putorius* | Native | 59.28355 | 14.921313 | Open Source |
| *Mustela putorius* | Native | 56.065235 | 14.602627 | Open Source |
| *Mustela putorius* | Native | 55.835269 | 13.560518 | Open Source |
| *Mustela putorius* | Native | 56.843277 | 15.980697 | Open Source |
| *Mustela putorius* | Native | 56.90878 | 14.010332 | Open Source |
| *Mustela putorius* | Native | 59.508247 | 14.976901 | Open Source |
| *Mustela putorius* | Native | 57.024579 | 14.703148 | Open Source |
| *Mustela putorius* | Native | 56.903959 | 12.865222 | Open Source |
| *Mustela putorius* | Native | 56.893647 | 15.872503 | Open Source |
| *Mustela putorius* | Native | 56.127333 | 12.614287 | Open Source |
| *Mustela putorius* | Native | 55.552332 | 13.781261 | Open Source |
| *Mustela putorius* | Native | 56.659181 | 15.76431 | Open Source |
| *Mustela putorius* | Native | 59.087841 | 15.683237 | Open Source |
| *Mustela putorius* | Native | 59.376277 | 14.064572 | Open Source |
| *Mustela putorius* | Native | 57.126704 | 15.414246 | Open Source |
| *Mustela putorius* | Native | 55.787997 | 12.966561 | Open Source |
| *Mustela putorius* | Native | 58.478803 | 12.40277 | Open Source |
| *Mustela putorius* | Native | 58.411146 | 11.328654 | Open Source |
| *Mustela putorius* | Native | 56.989354 | 12.874619 | Open Source |
| *Mustela putorius* | Native | 56.05253 | 12.773028 | Open Source |
| *Mustela putorius* | Native | 59.150712 | 15.392749 | Open Source |
| *Mustela putorius* | Native | 58.048268 | 12.46841 | Open Source |
| *Mustela putorius* | Native | 55.877757 | 12.817216 | Open Source |
| *Mustela putorius* | Native | 58.310072 | 15.724668 | Open Source |
| *Mustela putorius* | Native | 58.438804 | 12.2017 | Open Source |
| *Mustela putorius* | Native | 60.100871 | 18.403435 | Open Source |
| *Mustela putorius* | Native | 55.690074 | 13.492794 | Open Source |
| *Mustela putorius* | Native | 57.461103 | 14.125307 | Open Source |
| *Mustela putorius* | Native | 50.532978 | 12.131693 | Open Source |
| *Mustela putorius* | Native | 56.70335 | 9.423112001 | Open Source |
| *Mustela putorius* | Native | 55.976449 | 9.784553001 | Open Source |
| *Mustela putorius* | Native | 46.75032 | 22.35205 | Open Source |
| *Mustela nivalis* | Introduced | 37.7499 | -25.589 | Field Data |
| *Mustela nivalis* | Introduced | 37.7692 | -25.741 | Field Data |
| *Mustela nivalis* | Introduced | 37.83 | -25.669 | Field Data |
| *Mustela nivalis* | Introduced | 38.656195 | -27.27155 | Field Data |
| *Mustela nivalis* | Introduced | 38.654883 | -27.206117 | Field Data |
| *Mustela nivalis* | Introduced | 38.654892 | -27.279371 | Field Data |
| *Mustela nivalis* | Introduced | 38.65576 | -27.24339 | Field Data |
| *Mustela nivalis* | Introduced | 38.658767 | -27.233245 | Field Data |
| *Mustela nivalis* | Introduced | 38.6637 | -27.295869 | Field Data |
| *Mustela nivalis* | Introduced | 38.679602 | -27.27688 | Field Data |
| *Mustela nivalis* | Introduced | 38.684431 | -27.195542 | Field Data |
| *Mustela nivalis* | Introduced | 38.698398 | -27.175889 | Field Data |
| *Mustela nivalis* | Introduced | 38.70409167 | -27.04499167 | Field Data |
| *Mustela nivalis* | Introduced | 38.717619 | -27.061446 | Field Data |
| *Mustela nivalis* | Introduced | 38.72587 | -27.251461 | Field Data |
| *Mustela nivalis* | Introduced | 38.728503 | -27.255737 | Field Data |
| *Mustela nivalis* | Introduced | 38.729634 | -27.157603 | Field Data |
| *Mustela nivalis* | Introduced | 38.733241 | -27.140908 | Field Data |
| *Mustela nivalis* | Introduced | 38.7366 | -27.15763333 | Field Data |
| *Mustela nivalis* | Introduced | 38.7368 | -27.147496 | Field Data |
| *Mustela nivalis* | Introduced | 38.740222 | -27.058257 | Field Data |
| *Mustela nivalis* | Introduced | 38.751864 | -27.088741 | Field Data |
| *Mustela nivalis* | Introduced | 38.766468 | -27.375409 | Field Data |
| *Mustela nivalis* | Introduced | 38.772768 | -27.357283 | Field Data |
| *Mustela nivalis* | Introduced | 38.779157 | -27.252129 | Field Data |
| *Mustela nivalis* | Introduced | 38.781665 | -27.355065 | Field Data |
| *Mustela nivalis* | Introduced | 38.794622 | -27.245064 | Field Data |
| *Mustela nivalis* | Introduced | 38.796852 | -27.257696 | Field Data |
| *Mustela nivalis* | Introduced | 38.798752 | -27.252309 | Field Data |
| *Mustela putorius* | Introduced | 36.964 | -25.158 | Field Data |
| *Mustela putorius* | Introduced | 37.787 | -25.361 | Field Data |
| *Mustela putorius* | Introduced | 38.453 | -28.164 | Field Data |
| *Mustela putorius* | Introduced | 38.454492 | -28.172035 | Field Data |
| *Mustela putorius* | Introduced | 38.459 | -28.306 | Field Data |
| *Mustela putorius* | Introduced | 38.461 | -28.299 | Field Data |
| *Mustela putorius* | Introduced | 38.498 | -28.305 | Field Data |
| *Mustela putorius* | Introduced | 38.504 | -28.318 | Field Data |
| *Mustela putorius* | Introduced | 38.516976 | -28.378148 | Field Data |
| *Mustela putorius* | Introduced | 38.540234 | -28.460664 | Field Data |
| *Mustela putorius* | Introduced | 38.542545 | -28.442145 | Field Data |
| *Mustela putorius* | Introduced | 38.598318 | -28.62299 | Field Data |
| *Mustela putorius* | Introduced | 38.717295 | -27.151748 | Field Data |
| *Mustela putorius* | Introduced | 38.731972 | -27.256435 | Field Data |
| *Mustela putorius* | Introduced | 38.739717 | -27.262559 | Field Data |
| *Mustela putorius* | Introduced | 38.748848 | -27.263246 | Field Data |
| *Mustela putorius* | Introduced | 38.757009 | -27.264336 | Field Data |
| *Mustela putorius* | Introduced | 38.778157 | -27.252129 | Field Data |
| *Mustela putorius* | Introduced | 38.794628 | -27.196053 | Field Data |
| *Mustela putorius* | Introduced | 39.429 | -31.236 | Field Data |
| *Mustela putorius* | Introduced | 39.441 | -31.194 | Field Data |
| *Mustela putorius* | Introduced | 39.492 | -31.199 | Field Data |
| *Mustela putorius* | Introduced | 39.499 | -31.208 | Field Data |
| *Mustela putorius* | Introduced | 37.787 | -25.361 | Field Data |
