# Supplementary material for: Modelling the distribution of Mustela nivalis and M. putorius in the Azores archipelago based on native and introduced ranges
Source: PLoS One. 2020 Aug 7;15(8):e0237216. doi: 10.1371/journal.pone.0237216 (PMC7413552; doi:10.1371/journal.pone.0237216)
Supplement: S3 File — (DOCX) [file pone.0237216.s003.docx]

**S3 File.** Detailed explanation about obtained environmental data.

We selected a candidate set of 16 variables divided into four categories, namely: (1) topographic variables (two variables); (2) climatic variables (three variables); (3) landscape variables (seven variables) and (4) human-disturbance variables (three variables).

The topographic variables included altimetry and slope, and were retrieved from the digital elevation model available in WorldClim 1.4. The climatic variables included annual mean temperature, temperature annual range, annual precipitation and precipitation seasonality, and were obtained from WorldClim 1.4 (http://www.worldclim.org/). We obtained seven landscape variables from CORINE 2006 Land Cover (CLC2006; http://dataservice.eea.europa.eu). The CLC2006 is a semi-automated classification of satellite data into land cover/land use classes and is produced by the European Environmental Agency, and has a resolution of 100 m and a minimum mapping unit of 25 ha [1]. We used the CLC2006 to produce a land use/land cover map with nine classes (urban areas, industrial areas, agricultural areas, livestock areas, scrub and/or herbaceous vegetation, forestry areas, deciduous and mixed forests, uncovered areas, and wetlands and water bodies) by aggregating the 44 categories of the CLC2006. We used this map to calculate the percentage of each land use/land cover class in the 1 km^2^ cell, and used this percentage to extract our four landscape-variables: % forest areas, % coniferous forests, % deciduous and mixed forests, and % shrub and/or herbaceous vegetation. Additionally, we calculated the number of patches (total number of individual polygons) and total edge length (total meters of perimeter of the individual polygons) by 1 km^2^ cell. We calculated four human-disturbance variables based on percent cover by cell of agricultural areas and artificial areas (as described above). Population density was obtained from the global human settlement population grid (http://ghsl.jrc.ec.europa.eu/ghs_pop.php). We conducted all spatial data processing using the ArcGIS software (ArcGIS 10 ArcMap v. 10.1). All variables were scaled to 1km^2^ grids for both study areas.

**References**

**1.** Bossard M, Feranec J, Otahel J. CORINE land cover technical guide: Addendum 2000. 2000.
