# Supplementary material for: Modelling the distribution of Mustela nivalis and M. putorius in the Azores archipelago based on native and introduced ranges
Source: PLoS One. 2020 Aug 7;15(8):e0237216. doi: 10.1371/journal.pone.0237216 (PMC7413552; doi:10.1371/journal.pone.0237216)
Supplement: S4 File — (DOCX) [file pone.0237216.s004.docx]

**S4 File.** Correlation analysis between variables in native and introduced ranges.

To minimize model overfitting associated with potential correlation among predictor variables, we performed a pair-wise correlation analysis to identify and exclude highly correlated variables (r > 0.75), using the Spearman rank correlation coefficient. Correlation analysis were performed with R Studio software [1]. Highly correlated variables are remarked with a square in Tables S1 and S2. Consequently, the following variables were excluded the variables list: annual mean temperature (bio1), percent cover of coniferous forest (%f_con), percent cover of deciduous forest (%f_dec) and number of patches (n_pat).

**References**

**1.** RStudio Team. RStudio: Integrated Development for R. RStudio, Inc., Boston, MA; 2015. URL <http://www.rstudio.com/>

**Table S1.** Pair-wise correlation analysis between Azorean introduced range. Significant interactions (p-value < 0.05) are represented in bold. Highly correlated variables are remarked with a square (r > 0.75).

| Variables | alti | slop | bio1 | bio7 | bio12 | bio15 | land | %fore | %f_con | %f_dec | %scru | n_pat | edge | %agri | %arti | popu |
| --- | --- | --- | --- | --- | --- | --- | --- | --- | --- | --- | --- | --- | --- | --- | --- | --- |
| alti | **1.00** | **0.36** | **-0.99** | **0.60** | **0.70** | **-0.38** | **0.41** | **0.07** | **0.24** | **-0.10** | **0.40** | **-0.33** | **-0.41** | **-0.32** | **-0.40** | **-0.61** |
| slop | **0.36** | **1.00** | **-0.34** | **0.21** | **0.22** | **-0.36** | **0.31** | **0.28** | **0.12** | **0.24** | **0.16** | **-0.07** | **-0.08** | **-0.19** | **-0.31** | **-0.20** |
| bio1 | **-0.99** | **-0.34** | **1.00** | **-0.59** | **-0.71** | **0.35** | **-0.39** | **-0.05** | **-0.23** | **0.12** | **-0.40** | **0.33** | **0.41** | **0.30** | **0.38** | **0.60** |
| bio7 | **0.60** | **0.21** | **-0.59** | **1.00** | **0.09** | **-0.23** | **0.26** | **0.09** | **0.20** | **-0.06** | **0.10** | **-0.21** | **-0.26** | **-0.13** | **-0.15** | **-0.32** |
| bio12 | **0.70** | **0.22** | **-0.71** | **0.09** | **1.00** | **-0.22** | **0.22** | **-0.10** | **0.05** | **-0.17** | **0.37** | **-0.24** | **-0.28** | **-0.26** | **-0.26** | **-0.47** |
| bio15 | **-0.38** | **-0.36** | **0.35** | **-0.23** | **-0.22** | **1.00** | **-0.29** | **-0.14** | **-0.08** | **-0.10** | **-0.34** | **0.18** | **0.15** | **0.36** | **0.23** | **0.27** |
| land | **0.41** | **0.31** | **-0.39** | **0.26** | **0.22** | **-0.29** | **1.00** | **0.48** | **0.33** | **0.32** | **0.27** | **-0.09** | **-0.12** | **-0.49** | **-0.34** | **-0.34** |
| %fore | **0.07** | **0.28** | **-0.05** | **0.09** | **-0.10** | **-0.14** | **0.48** | **1.00** | **0.51** | **0.82** | **-0.05** | **0.30** | **0.26** | **-0.39** | **-0.14** | **-0.10** |
| %f_con | **0.24** | **0.12** | **-0.23** | **0.20** | **0.05** | **-0.08** | **0.33** | **0.51** | **1.00** | 0.02 | **0.05** | **0.13** | **0.09** | **-0.24** | **-0.15** | **-0.19** |
| %f_dec | **-0.10** | **0.24** | **0.12** | **-0.06** | **-0.17** | **-0.10** | **0.32** | **0.82** | 0.02 | **1.00** | **-0.10** | **0.32** | **0.30** | **-0.28** | **-0.07** | 0.02 |
| %scru | **0.40** | **0.16** | **-0.40** | **0.10** | **0.37** | **-0.34** | **0.27** | **-0.05** | **0.05** | **-0.10** | **1.00** | **-0.05** | **-0.08** | **-0.62** | **-0.23** | **-0.33** |
| n_pat | **-0.33** | **-0.07** | **0.33** | **-0.21** | **-0.24** | **0.18** | **-0.09** | **0.30** | **0.13** | **0.32** | **-0.05** | **1.00** | **0.82** | 0.03 | **0.32** | **0.31** |
| edge | **-0.41** | **-0.08** | **0.41** | **-0.26** | **-0.28** | **0.15** | **-0.12** | **0.26** | **0.09** | **0.30** | **-0.08** | **0.82** | **1.00** | -0.03 | **0.32** | **0.33** |
| %agri | **-0.32** | **-0.19** | **0.30** | **-0.13** | **-0.26** | **0.36** | **-0.49** | **-0.39** | **-0.24** | **-0.28** | **-0.62** | 0.03 | -0.03 | **1.00** | 0.00 | **0.26** |
| %arti | **-0.40** | **-0.31** | **0.38** | **-0.15** | **-0.26** | **0.23** | **-0.34** | **-0.14** | **-0.15** | **-0.07** | **-0.23** | **0.32** | **0.32** | 0.00 | **1.00** | **0.40** |
| popu | **-0.61** | **-0.20** | **0.60** | **-0.32** | **-0.47** | **0.27** | **-0.34** | **-0.10** | **-0.19** | 0.02 | **-0.33** | **0.31** | **0.33** | **0.26** | **0.40** | **1.00** |

**Table S2.** Pair-wise correlation analysis between European native range. Significant interactions (p-value < 0.05) are represented in bold. Highly correlated variables are remarked with a square (r > 0.75).

| Variables | alti | slop | bio1 | bio7 | bio12 | bio15 | land | %fore | %f_con | %f_dec | %scru | n_pat | edge | %agri | %arti | popu |
| --- | --- | --- | --- | --- | --- | --- | --- | --- | --- | --- | --- | --- | --- | --- | --- | --- |
| alti | **1.00** | **0.12** | **-0.09** | **0.20** | **0.27** | **0.22** | **0.17** | 0.01 | 0.00 | 0.01 | 0.01 | **0.06** | **0.12** | **-0.03** | **-0.02** | **-0.29** |
| slop | **0.12** | **1.00** | 0.01 | **-0.04** | **0.07** | **0.06** | **0.04** | 0.00 | 0.00 | 0.00 | 0.00 | **0.04** | **0.04** | 0.00 | 0.01 | **-0.05** |
| bio1 | **-0.09** | 0.01 | **1.00** | **-0.32** | **-0.14** | **-0.23** | **-0.21** | **-0.02** | **-0.02** | 0.01 | **-0.02** | **0.03** | **0.03** | **0.04** | 0.00 | **0.30** |
| bio7 | **0.20** | **-0.04** | **-0.32** | **1.00** | **-0.51** | **0.46** | **-0.07** | 0.01 | 0.01 | 0.00 | -0.01 | **0.08** | **0.08** | **-0.04** | 0.01 | **-0.14** |
| bio12 | **0.27** | **0.07** | **-0.14** | **-0.51** | **1.00** | **-0.06** | **0.24** | 0.01 | -0.01 | 0.01 | **0.02** | 0.01 | 0.01 | -0.01 | -0.01 | **-0.03** |
| bio15 | **0.22** | **0.06** | **-0.23** | **0.46** | **-0.06** | **1.00** | **0.07** | 0.01 | 0.01 | 0.01 | -0.01 | **0.09** | **0.12** | **-0.04** | 0.00 | **-0.23** |
| land | **0.17** | **0.04** | **-0.21** | **-0.07** | **0.24** | **0.07** | **1.00** | 0.01 | 0.01 | 0.01 | 0.00 | 0.00 | **0.04** | **-0.02** | **-0.04** | **-0.36** |
| %fore | 0.01 | 0.00 | **-0.02** | 0.01 | 0.01 | 0.01 | 0.01 | **1.00** | **0.78** | **0.33** | 0.00 | **-0.03** | **-0.02** | 0.00 | 0.00 | **-0.02** |
| %f_con | 0.00 | 0.00 | **-0.02** | 0.01 | -0.01 | 0.01 | 0.01 | **0.78** | **1.00** | 0.00 | 0.00 | **-0.02** | **-0.02** | 0.00 | 0.00 | **-0.02** |
| %f_dec | 0.01 | 0.00 | 0.01 | 0.00 | 0.01 | 0.01 | 0.01 | **0.33** | 0.00 | **1.00** | 0.00 | -0.01 | -0.01 | 0.00 | 0.00 | 0.00 |
| %scru | 0.01 | 0.00 | **-0.02** | -0.01 | 0.02 | -0.01 | 0.00 | 0.00 | 0.00 | 0.00 | **1.00** | -0.01 | -0.01 | 0.00 | 0.00 | **-0.02** |
| n_pat | **0.06** | **0.04** | **0.03** | **0.08** | 0.01 | **0.09** | 0.00 | **-0.03** | **-0.02** | -0.01 | -0.01 | **1.00** | **0.75** | **-0.09** | 0.01 | **0.17** |
| edge | **0.12** | **0.04** | **0.03** | **0.08** | 0.01 | **0.12** | **0.04** | **-0.02** | **-0.02** | -0.01 | -0.01 | **0.75** | **1.00** | **-0.09** | 0.00 | **0.11** |
| %agri | **-0.03** | 0.00 | **0.04** | **-0.04** | -0.01 | **-0.04** | **-0.02** | 0.00 | 0.00 | 0.00 | 0.00 | **-0.09** | **-0.09** | **1.00** | 0.00 | 0.00 |
| %arti | **-0.02** | 0.01 | 0.00 | 0.01 | -0.01 | 0.00 | **-0.04** | 0.00 | 0.00 | 0.00 | 0.00 | 0.01 | 0.00 | 0.00 | **1.00** | **0.05** |
| popu | **-0.29** | **-0.05** | **0.30** | **-0.14** | **-0.03** | **-0.23** | **-0.36** | **-0.02** | **-0.02** | 0.00 | **-0.02** | **0.17** | **0.11** | 0.00 | **0.05** | **1.00** |
