# Supplementary material for: Modelling the distribution of Mustela nivalis and M. putorius in the Azores archipelago based on native and introduced ranges
Source: PLoS One. 2020 Aug 7;15(8):e0237216. doi: 10.1371/journal.pone.0237216 (PMC7413552; doi:10.1371/journal.pone.0237216)
Supplement: S5 File — (DOCX) [file pone.0237216.s005.docx]

**S5 File**. Figure detailing the stepwise modeling process.

STEP I: select input data for species presence and background points. Species records (from native European range and non-native Azorean range) were filtered to avoid pseudo-replication and spatial biases. Background points were selected so that they reflect the same biases as the species presence records. To mimic this bias we included environmental filtering variables to produce a mask from which to randomly+bias draw potential background points. Input data was then entered into Maxent to select parameter ranges. Best models were selected using AUC. STEP II: Once parameter sets were selected, we used environmental variables as candidates for predicting species presence in the native and introduced range. These included topographic, climate, landscape and human variables. Best models were selected using AICc, and we used these to produce maps of potential range in the Azores as well as uncertainty maps.


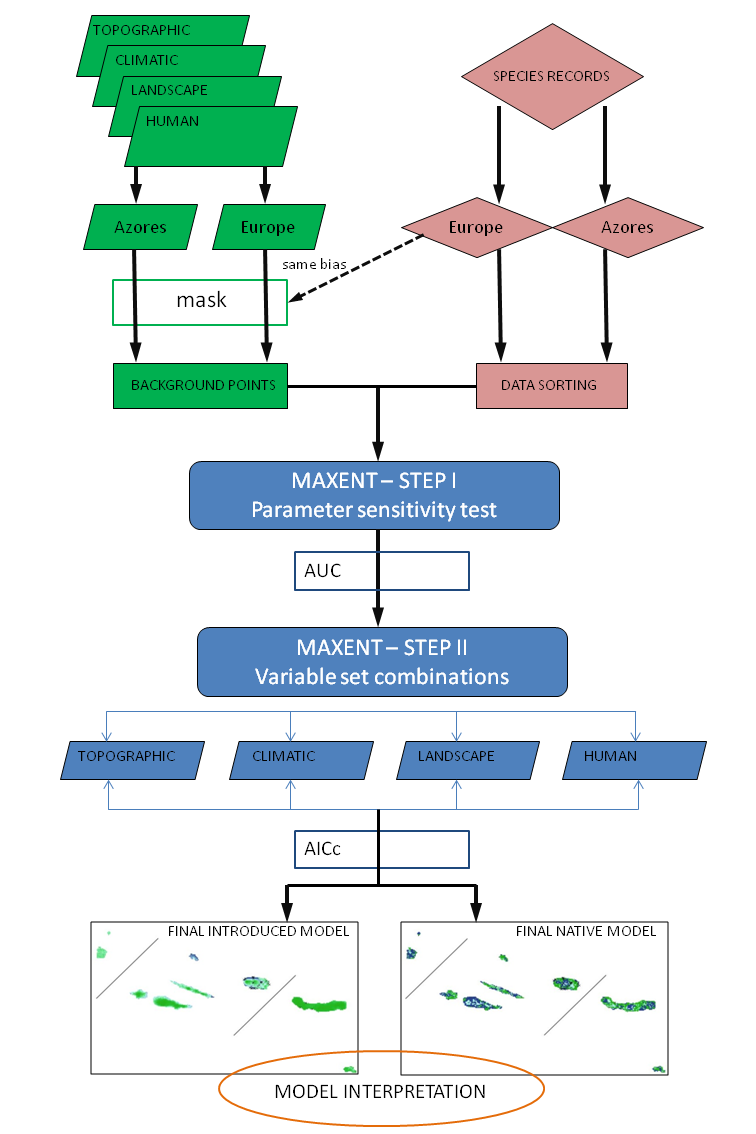


**Fig S1**. Stepwise modeling process.
