# Supplementary material for: Modelling the distribution of Mustela nivalis and M. putorius in the Azores archipelago based on native and introduced ranges
Source: PLoS One. 2020 Aug 7;15(8):e0237216. doi: 10.1371/journal.pone.0237216 (PMC7413552; doi:10.1371/journal.pone.0237216)
Supplement: S6 File — (DOCX) [file pone.0237216.s006.docx]

**S6 File**. Combination of sets of candidate predictor variables.

**Table S1.** Combination of sets of candidate predictor variables to perform the model selection. n=number of variables included in the model.

| **Model** | **Variables** | **n** |
| --- | --- | --- |
| topographic + climatic + landscape + human | altimetry + slope + clim_bio7 + clim_bio12 + clim_bio15 + landcover + %forest_areas + %scrub_herb + edge_density + %agricultural_areas + artificial_areas + population_density | 12 |
| topographic + climatic + landscape | altimetry + slope + clim_bio7 + clim_bio12 + clim_bio15 + landcover + %forest_areas + %scrub_herb + edge_density | 9 |
| topographic + climatic + human | altimetry + slope + clim_bio7 + clim_bio12 + clim_bio15 + %agricultural_areas + artificial_areas + population_density | 8 |
| climatic + landscape + human | clim_bio7 + clim_bio12 + clim_bio15 + landcover + %forest_areas + %scrub_herb + edge_density + %agricultural_areas + artificial_areas + population_density | 10 |
| topographic + climatic | altimetry + slope + clim_bio7 + clim_bio12 + clim_bio15 | 5 |
| topographic + landscape | altimetry + slope + landcover + %forest_areas + %scrub_herb + edge_density | 6 |
| climatic + landscape | clim_bio7 + clim_bio12 + clim_bio15 + landcover + %forest_areas + %scrub_herb + edge_density | 7 |
| climatic + human | clim_bio7 + clim_bio12 + clim_bio15 + %agricultural_areas + artificial_areas + population_density | 6 |
| landscape + human | landcover + %forest_areas + %scrub_herb + edge_density + %agricultural_areas + artificial_areas + population_density | 7 |
| topographic | altimetry + slope | 2 |
| climatic | clim_bio7 + clim_bio12 + clim_bio15 | 3 |
| landscape | landcover + %forest_areas + %scrub_herb + edge_density | 4 |
| human | %agricultural_areas + artificial_areas + population_density | 3 |
