# Supplementary material for: Modelling the distribution of Mustela nivalis and M. putorius in the Azores archipelago based on native and introduced ranges
Source: PLoS One. 2020 Aug 7;15(8):e0237216. doi: 10.1371/journal.pone.0237216 (PMC7413552; doi:10.1371/journal.pone.0237216)
Supplement: S7 File — (DOCX) [file pone.0237216.s007.docx]

**S7 File.** Maps of uncertainty in predictions.

We created an overlap of n-1 models (n= number of occurrence records) based on the introduced range to test how many times a given cell was predicted to be suitable (Habitat Suitability Index > 0.5) for both study species.


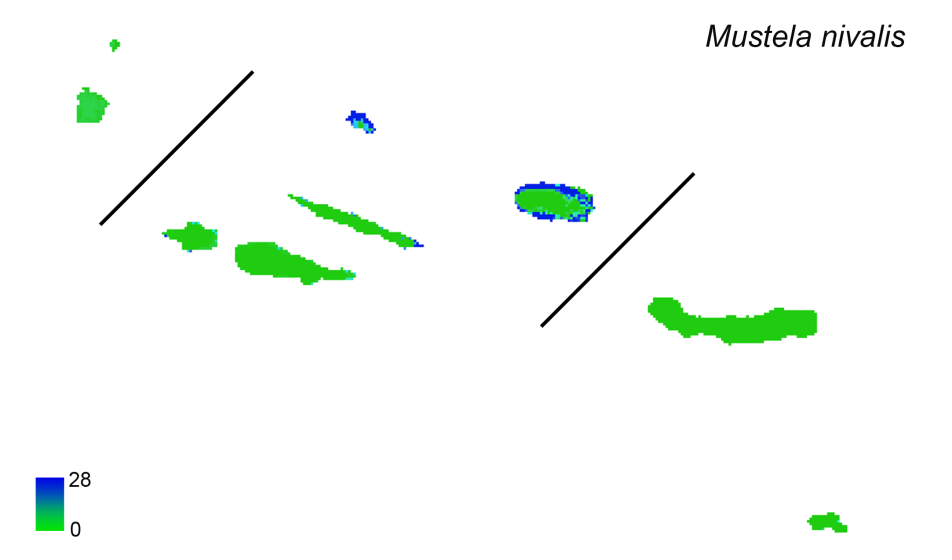


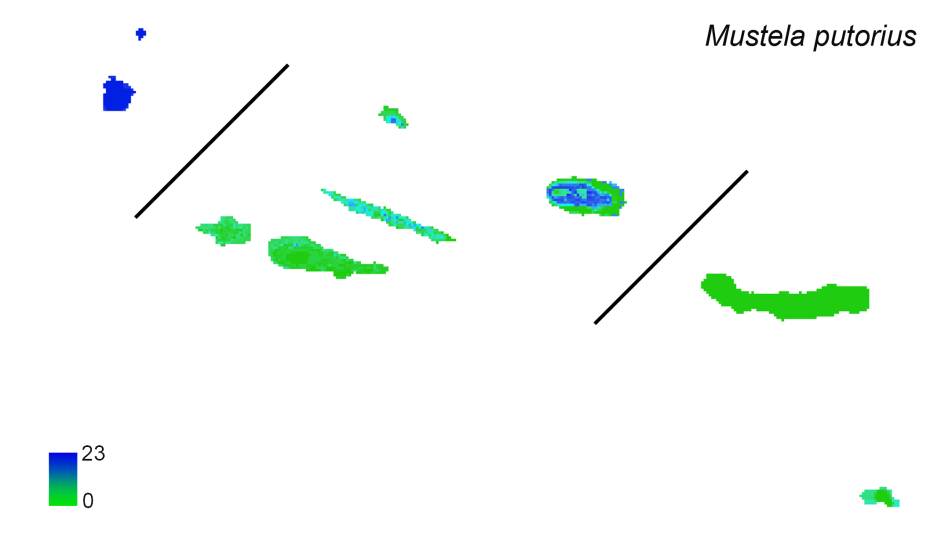


**Fig S1**. Uncertainty in prediction, for *M. nivalis* and *M. putorius*.
