# Supplementary material for: Modelling the distribution of Mustela nivalis and M. putorius in the Azores archipelago based on native and introduced ranges
Source: PLoS One. 2020 Aug 7;15(8):e0237216. doi: 10.1371/journal.pone.0237216 (PMC7413552; doi:10.1371/journal.pone.0237216)
Supplement: Attachment — Submitted filename: Response to Reviewers.docx [file pone.0237216.s008.docx]

**Response to Reviewers**

***Reviewer #1:***

*This paper is a review of a previous paper submitted to PLOS One. Its aim is to model habitat suitability in the Azores archipelago for two introduced mustelid species, Mustela nivalis and Mustela putorius. For each species, the authors fit two models: (i) one model fitted to a dataset of detections collected over the whole Europe (native range), and (ii) one model fitted to a dataset of detections collected only in the Azores islands (introduced range). For each species, the authors then compare the maps predicted by the two models (introduced and native), and show that the maps obtained with the two datasets are different. The map predicted using data collected over the whole Europe does not correspond to the map predicted using data collected in the Azores. The authors conclude that this difference indicates that species distribution models should be carefully interpreted when used to predict the range of a species in an area based on data collected in another area.

In my review of the previous manuscript, I expressed several major concerns related to the methods used to reach the aim, as well as several minor comments. Many of my comments have been satisfactorily taken into account. However, I think that we disagree on my main comment: I think that the comparison between native and introduced models carried out in this paper does not allow to draw conclusions on the ability to predict introduced range from the native range, because of the very different scales of study for the two areas.

It is possible that I was not clear enough, so that I explain more clearly below why I think that the methodology in this paper is very problematic. Moreover, the more precise description that the authors now give of the methodology used to correct for sampling bias raises another major methodological problem.

I describe these two problems below.*

**Authors**: We are grateful for your detailed review. You have raised two major concerns regarding the methodology: (i) scale and (ii) bias correction. In summary we believe these concerns are not fully warranted because of the way we designed our study, the comparison between islands and continents, the predictor variables selected, and the methods chosen. In general, we followed methodologies, software and analysis described as best practices used in SDM (Araujo et al. 2019).

Indeed, we agree that SDM transferability should be better studied, which is perhaps extremely important when using these tools for, for example, studies that project range expansions or considerable range contractions – changing dramatically spatial extent of predictor beyond the information range, and invasive species. One way to ameliorate the spatial extent effect could be by having similar sampling intensities, or iteratively transfer models across spatial locations or points in time. This would make for very nice studies, but much beyond our simple analysis. Below we provide much more detail to our reasoning for the issue of scale and bias correction.

Araújo MB, Anderson RP, Barbosa AM, Beale CM, Dormann, C. F., Early, R., et al. Standards for distribution models in biodiversity assessments. Sci Adv. 2019; 5(1): eaat4858.

******

*** On the concept of scale.

In my previous review, my main criticism was related to the difference of geographical scales between the model fitted in the native range and the model fitted in the Azores archipelago. I expressed it in my previous review by noting that results may vary strongly between scales, and comparing a model fitted using data collected over continental scale and a model collected on a highly local scale is meaningless, as the two models are necessarily describing very different processes, so that they are necessarily returning different results.*

**Authors**: Thank you for your comment, this is indeed a major aspect to consider in any SDM study. Soberon (2007) illustrated that species respond to environmental conditions like habitat type, food resources, etc at local scales, and to environmental conditions like bioclimate and topography at larger scales and that in order to fully understand factors that drive species ranges we would need to derive nested models at these scales. Unfortunately, the literature on SDM has yet to get to this stage but many examples are now emerging that combine both bioclimatic and habitat variables and show that there is an interplay of the importance of the two (Santos et al. 2017). This is further complicated when dealing with invasive species which likely change both associations with habitat and bioclimatic parameters in the invaded range – that is what makes them invasive in the first place. Further, as for our case, there is a complication because we are studying islands. The spatial extent of the invaded area is spatially confined because these are islands, and therefore any conversion of this spatial extent to a continuous continental surface would be arbitrary, and add spurious edge effects for the continental models which would not be present in the islands. To account for all these aspects we carefully designed our study so that:

1. We included the same predictor variables in both the islands and the continental area. These include both bioclimatic variables – to represent processes at large scales, and habitat variables – to represent processes at local scales. In the text: ”We selected (1) two topographic variables (e.g., slope) in order to describe the physical environment; (2) four climatic variables (e.g., annual temperature, precipitation seasonality) to describe the bioclimatic conditions; (3) a set of seven landscape level variables, including habitat type, vegetation structure (herbs, shrubs or trees), land use types, and landscape heterogeneity (e.g., number of patches); and (4) three human disturbance variables, related with human population (e.g., population density) and their activities (% of artificial areas, % of agricultural areas).”

2. We collected the same variables in the islands and the continent. To represent the continent, we did not analyze any continuous surface, but rather chose to select 10,000 isolated cells, randomly selected from whole Europe.

3. We experimented with the scenario you proposed to restrict the native-area to a small portion of the native range of both species that would match the spatial extent of the Azores. We were confronted with several choices that we were not comfortable making – (i) where to seed the area? (ii) Which shape should it take? (iii) are we inducing arbitrarious edges? (iv) if we created 2,250 km^2^ areas, how do we then justify the differences between the models? (v) how do we control for very uneven sample size within the Azores, like continental areas? Additionally, the particular environmental conditions of the Macaronesian archipelagos, and particularly of the Azores, are not "comparable" with a continental area with a similar extent. Therefore, we opted to sample from the native-range and used 10,000 isolated cells.

4. We are unaware of where do the species invade from, so this would also limit selecting certain areas within the European range.

5. Of course it is possible that mismatched spatial extents of study areas affect model transferability, however this is an aspect seldom analyzed in SDM studies. Transferability studies are few and far in between, and there are yet no clear guidelines on how to do them properly because indeed we are transferring models developed with a given range of a variable to areas where the range of values the variable can take is different – this is the case for invasive species. Further studies should do an experiment on this topics.

Soberón J. Grinnellian and Eltonian niches and geographic distributions of species. Ecol Lett. 2007; 10(12): 1115−1123.

Santos MJ, Smith AB, Thorne JH. et al. The relative influence of change in habitat and climate on elevation range limits in small mammals in Yosemite National Park, California, U.S.A. Clim Chang Responses. 2017; 4: 7.

*To this criticism, the authors replied: "we believe this effect is not affecting the model outputs because we used the same spatial resolution and the same data sources for both study areas."

Actually, the concept of scale in Ecology is more general than just the resolution of the study (Dungan et al. 2002): the concept of scale involves many aspects (grain, lag, support, etc.).*

**Authors**: We agree and see above our comment to this. Indeed what we are talking about is the extent of the study area.

*But in this study, the most problematic aspect is the extent of the study area, rather than its resolution. Dungan et al. (2002) illustrates clearly how the results of a study might change strongly when the scale changes. Another reference here -- a seminal one actually -- is Johnson (1980), who defines in the discussion four orders of habitat selection (first order: geographical range, second order: distribution of animals in a region, third order: distribution of animal locations within their home ranges, fourth order: selection of food items at the locations). The processes and preferences at a scale are not necessarily the same than those at another. In the present study, the authors compare first order selection (whole Europe) with second order selection (distribution within the islands). Many other authors have stressed the importance of this scale and variability of the results according to the scale considered (e.g. Pearce and Boyce 2006, Soberon and Peterson 2005).*

**Authors**: Thank you for your comment, this is indeed a major aspect to consider in any SDM study. As mentioned above, we took the approach from Soberon (2007), which illustrated that species respond to environmental conditions like habitat type, food resources, etc at local scales, and to environmental conditions like bioclimate and topography at larger scales and that in order to fully understand factors that drive species ranges we would need to derive nested models at these scales. Unfortunately, the literature on SDM has yet to get to this stage but many examples are now emerging that combine both bioclimatic and habitat variables and show that there is an interplay of the importance of the two (Santos et al. 2017). This is further complicated when dealing with invasive species which likely change both associations with habitat and bioclimatic parameters in the invaded range – that is what makes them invasive in the first place.

More specifically, our selected native area is not strictly a continental area, as we are sampling 10,000 isolated cells within Europe (Fig R1; see ESM1 for more details). Therefore, the extent (i.e. the total area) used during the modeling process is similar between regions (extent of introduced range: 2,250 background points, extent of native range: 3,000 background points). As the native area is formed by isolated cells obtained from a continental area, the scale is not necessarily a problem. To address Johnson (1980) orders of habitat selection, we chose variables that represent processes at each of the orders: (1) First order: geographical range – we use bioclimatic variables; (2) Second order: distribution of animals in a region – we use habitat and topographic variables; (3) Third order: distribution of animal locations within their home ranges – vegetation structure (herbs, shrubs or trees), human presence; (4) Fourth order: selection of food items at the locations – vegetation structure.

**Fig R1.** Distribution of background points exhibiting the same spatial bias than presence data (a) in areas with high density of presence data and (b) in areas with low density of presence data. In (a) and (b) we can observe examples of isolated cells which form the native-area. The conditions of this native area model are not strictly influenced by the scale or the mainland effect.

Soberón J. Grinnellian and Eltonian niches and geographic distributions of species. Ecol Lett. 2007; 10(12): 1115−1123.

Santos MJ, Smith AB, Thorne JH. et al. The relative influence of change in habitat and climate on elevation range limits in small mammals in Yosemite National Park, California, U.S.A. Clim Chang Responses. 2017; 4: 7.

Phillips SJ, Dudík M, Elith J, Graham CH, Lehmann A, Leathwick J, Ferrier S. Sample selection bias and presence‐only distribution models: implications for background and pseudo‐absence data. Ecol Appl. 2009; 19(1): 181−197.

Ferrier S, Watson G, Pearce J, Drielsma M. Extended statistical approaches to modelling spatial pattern in biodiversity in northeast New South Wales. I. Species-level modelling. Biodivers Conserv. 2002; 11(12): 2275−2307.

*More concretely, here, any analysis of environmental variables classically used for SDM on the continental scale in Europe will mainly show large environmental patterns. For example, on a continental scale, the effect of elevation on the presence of a species will be driven by the differences between mountainous areas (the Alps) and other areas. That is, by the differences of weather, climate, vegetation, snow cover, etc. between Alpine and non-Alpine climates. Of course, other climatic variables will also account for it to some extent, but no variable will synthetize the difference between non-Alpine and Alpine climate as efficiently as the elevation (by definition !). Therefore, any effect of the elevation in SDM models at this scale will summarize the difference of environments (vegetation, climate, etc.) between Alpine and non-Alpine environments.* *Therefore, if -- for example -- a species is absent or rarer in the Alps than in the rest of the continent, it will be because the Alpine climate is less suitable for the species than non-Alpine climates (whatever the reason, e.g. too much snow in winter, rocky soils, etc.). This will be synthetized by a negative effect of the elevation in the SDM*

*On the other hand, the elevation, in the Azores archipelago, has a very different meaning. In these islands, high elevation areas are not characterized by a "more Alpine climate" than other areas. It has a very different meaning. In these islands, for example, there is probably a very strong correlation between elevation and the distance to the sea. So that elevation here rather represents this distance (e.g. further from the sea = less urban, etc. -- maybe a better habitat for the species?). Thus the meaning of elevation for the species is not the same, just because we do not work at the same scale. Of course, my example here describes a fictious species (I do not know what are the selection patterns for weasel and ferret at this scale), but it illustrates why results are expected to vary between two scales, even within the native range (e.g. the results will not be the same if we compare a model fitted on the whole Europe and an model fitted e.g. on similar sized areas in continental Portugal).*

**Authors**: Indeed, interpretation of the effect of elevation is different for the Alps and the Azores, but we do not believe this is linked to scale but rather we are talking about the second order from Johnson (1980) and the local conditions in Soberon’s work or the geographic factors that determine species distributions in mountains from Grinnell or Humboldt. Indeed, we are dealing with mountains in islands and not continental mountains. We carefully interpret the coefficients (direction and magnitude) in light of the context in which they occur. Often the proxy elevation is used to represent the variety of conditions that occur with zonations in mountains (Schrodt et al. 2019). The specific mechanisms by which individuals and species respond to these are seldom not represented in SDMs. This gets back to the use of SDM to predict distribution, habitat selection, or individual movements – and in the ecological literature for each of these processes there are different types of models. SDM, as the name states, refers to geographical distributions and some authors even argue they should not be used to infer habitat use or animal movements as inferred from Johnson (1980) orders of habitat selection across scales.

Schrodt F, Santos MJ, Bailey JJ, Field R. Challenges and opportunities for biogeography—What can we still learn from von Humboldt?. J Biogeogr. 2019; 46(8): 1631−1642.

*The same is true for e.g. climatic variables. The effect of these variables on a continental scale will likely reflect the differences in densities between the different climates in Europe. If a species is less abundant in e.g. areas with continental climates (with less precipitation), then the effect of clim_bio12 in a SDM on a continental scale will mainly represent this large scale pattern. At the scale of the Azores Islands, this variable will not have the same meaning, as there is no continental climate in the Azores islands. Etc.*

**Authors**: Indeed, interpretation of the effect of any variable is different due to the context and in the discussion we state how we interpret these effects. This is true for any SDM study.

*If the aim is to predict the introduced range from data collected in the native range, it would make much more sense to compare this archipelago with areas *of similar sizes* located in similar climates, with a similar elevation range, etc.*

**Authors**: Thank you for the suggestion. As mentioned above we experimented with it, and our conclusion was that the criteria to select areas of similar extent to that of the Azores, located in similar climates and with a similar elevation range would be extremely arbitrary. First, selecting random 2,250 km^2^ areas in a continent that is continuous would suffer from many of the issues you mention above as to why we should do it. Second, there are no similar climates to the Azores in continental Europe, and third, as in your explanation, the Alps and the mountains of the Azores are different in the way they affect species distributions therefore not warranting making these choices. Therefore we chose to represent our native area by non-connected cells, i.e., sampling on the native range.

*I suggested this approach in my previous review, but the authors replied:*

*"We concluded that it would be difficult to justify selecting sub areas within continental Europe that would match the size of the Azores to conduct the analysis as suggested. This is because there could be an even bigger effect due to this selection than the potential effects of modeling a smaller area using information from a larger area."*

*I do not understand why it would be difficult to justify it. I do not think that there would be a bigger effect in this selection. The authors have a very precise description of their study area according to numerous environmental variables (climate, elevation, etc.). It would be easy to select randomly numerous places of similar sizes in Europe. The standardized environmental variables (i.e. minus the mean and divided by the standard deviation) then each define a dimension in a multidimensional space (one dimension is the average temperature, one is the elevation, on is the precipitation, etc.). Each one of these random areas would define a point in this space. The Azores archipelago also defines a point in this space. Then, we can select a sample of -- say -- 10 random places with the smallest Euclidean distance in this multidimensional space to Azores. The resulting sample will be a random and objective sample of by areas taken in the native range with sizes and environmental conditions similar to the target area. This would be a better approach in my opinion: if the aim is to infer the effect of a factor on a process by comparing two sets of areas -- one with the factor and one without -- it is better to design the study so that only that factor varies and the other variables are identical. Here, to try to find one or several study areas in the native range with sizes and conditions similar to those of the Azores archipelago, with only "introduced/native" differing. On the other hand, as exemplified above, ignoring the effect of scale will lead to erroneous conclusions.*

**Authors**: Thanks for your suggestion. Although we respectfully disagree, we tried to perform the suggested analysis. First, we created a polygon with similar extension to the Azores (2,250 km^2^; 75x30 cells). After that, we randomly generated 100 polygons across whole Europe (see Fig R2). The polygons were generated in areas with presence data (i.e. all polygons possess occurrence-records of the target species). We calculated the mean value of the bioclimatic variables for each polygon. Means of the bioclimatic variables were also calculated for the Azores. Finally, we performed a PCA analysis and selected the polygons with the smallest Euclidean distance in this multidimensional space to Azores (see Fig R3).

**Fig R2**. A total of 100 2,250 km^2^-cells (75x30 km) were randomly generated. All generated cells contain presence data.

**Fig R3**. PCA Analysis results performed using four bioclimatic variables: bio1 - annual mean temperature; bio7 - temperature annual range; bio12 - annual precipitation; and bio15 - precipitation seasonality. Numbers correspond the generated cell's ID; 101 corresponds to the Azores.

However, due to the large extension of Europe and the disperse spatial location of the occurrence records, the polygon with most occurrence data had only 30 records (our native area, i.e., Europe, has more than 1,500 occurrence records).

The results of the PCA showed that the polygons with more similar conditions to the Azores had less than 10 occurrence records (polygons ID 10 and 9, see Fig R3). These polygons were located in the occidental area of the Iberian peninsula, near to the coast line.

In conclusion, although with the analysis suggested by the reviewer the extent of the study was similar to the Azores (2,250 km^2^ *vs* 2,300 km^2^), in our study the extent of the native area used to perform the models was also similar (3,000; i.e.10,000 total background points, from which MaxEnt randomly selected 30%). However, the number of presence data used to run the models is dramatically lower using 75x30 polygons in comparison with our selected native-area.

*The authors further note:*

*"Further, we believe that this suggestion is not in line with other approaches to model invasive species ranges. Several authors have also modeled invasive species range from information retrieved from the native range (e.g., Broennimann et al., 2007; Fitzpatrick et al., 2007; Rödder et al., 2009; Bidinger et al., 2012); This is because invasive species are yet to be in equilibrium with their environment in the invaded ranges thus becoming even more important to include a broader set of parameter ranges from the native range to predict areas with potential for invasion."*

*However, none of these works compare areas with so large size differences. Broenniman et al. (2007) predict the distribution of spotted knapweed in North America with a model fitted with data collected in its native range in western Europe, two areas covering similar sizes. Fitzpatrick et al. (2007) compare the distribution of red ants in tropical south America and tropical north America, again at similar scales and in similar climates. Rödder et al. (2009) study the slider turtle in their native range (North America), and the invasive range is also on a continental scale. Finally, Bidinger et al. (2012) studies the distribution of Harlequin ladybird in their native range in eastern Asia, and in their introduced range (area of similar size in Europe).*

*I fully endorse the aim of the present study. From a conservation as well as ecological point of view, it is essential to find a way to predict the introduced range from the native range of invasive species. I agree on the importance of understanding how the introduced range might differ from the native range to identify how a species adapt to a new environment. I do not disagree on the aim, but on the method. The problem of scale is not a minor one in ecology. Comparing two areas of very different sizes amounts to compare a process at two very different scales. Therefore, different results are expected, even if there is no difference between native and introduced ranges.*

**Authors**: At the moment we have conducted three analysis:

1. Analysis with 10,000 cell across Europe and the Azores ─ in the original manuscript;
2. Additional analysis of the occidental part of the Iberian peninsula as the native-area (comprising mainly the north-west of Spain and Portugal) and the Azores. (not in the manuscript). We selected this area because: (1) it comprises a large area to where both species are native, thus including most relevant ecological requirements of the study species; and (2) it is the closest native distribution area for the study species. Now, the results of the PCA (Fig R3) showed that this native area has similar environmental conditions to the Azores. We received criticism for this approach because it is unknown where the original introduced individuals originated from, as they easily could have originated from another region in their large native range. By restricting the study to a small portion of the native range of both species the authors are not capturing the full range of environments these species can inhabit and thus will impact the model predictions and comparisons with the invaded range model;
3. We attempted the proposed approach of generating 2,250 km^2^ areas and select those with closer climatic properties to the Azores, but as explained above we ran into limitations of sample sizes

So we are facing a trade-off, of restricting analyses to randomly selected areas of the same size of the Azores (as mentioned above, a bit difficult to justify their choice) and have small samples, or run models sampling from the native range in a similar area as the Azores (10,000 cells is a similar number of cells to those used for the Azores models). Therefore, selecting a smaller area, the number of native records would be dramatically lower and would not reflect the native range. This is the reason why other reply was "we believe that this suggestion is not in line with other approaches to model invasive species ranges [...] This is because invasive species are yet to be in equilibrium with their environment in the invaded ranges thus becoming even more important to include a broader set of parameter ranges from the native range to predict areas with potential for invasion."

*******
** On the correction of sampling effort.

The authors rightly explained that the biased data collection characterizing the dataset would lead to biased inference if it was not taken into account. They use the "target group" method of Philips et al. (2009) to collect a biased sample of background points exhibiting the same bias as the presence records. The authors did not describe clearly their approach in the previous version of the manuscript. It is now more clearly described.

The "target group" approach of Philips et al. aims at distinguishing whether the absence of detection of the focus species at one place is caused by the absence of the species itself or by the absence of data collection. The idea is to define a "target group" containing many species for which we think that the collection or observation activities of collectors is similar to those of the focus species. For example, to model the SDM of a particular bird species in a citizen science program, it would make sense to use the whole set of bird species studied in the citizen science program as the target group used to select background points. The hope is that, at any point where data collection for the focus species has occurred, at least one species of the target group was present and reported by the same data collection (even if the focus species itself is not). So that the presence of a species of the target group and the absence of the focus species ensures that the absence of reported detection for this focus species is likely due to the actual absence of the species.

This method allows to correct -- to some extent, it is impossible to completely account for all the bias in such contexts -- the collection bias because the target group is usually made of a large number of species, and at least one is supposed to be present where data collection has occurred. In the paper of Philips, for example, the target group contains from 7 to 52 species.

In the present paper, the target group was defined by just the ferret and the weasel. This is a small target group! It means that any area unsuitable for both species, but* *where data collection has actually occurred will not be present in the data. The background sample will be defined by the set of habitat conditions allowing the presence of the weasel and/or the ferret (well, a biased sample of it, but this is the aim of the target group method to obtain such a biased sample of background points). So that modelling the presence of the weasel (resp. ferret) in a set of locations defined so that either the ferret or the weasel are present, is not a model of the species distribution. It is a model of the niche difference between the two species: the model predicts the probability of presence of one species given that at least one of the two species is present.

So that the "native" model describes the niche differences between ferret and weasel modelled at the scale of the continent species, whereas the "introduced" model describes theses differences at the scale of the archipelago. In other words, the models do not focus on the potential distribution of the species as indicated in the paper, but on the differences between the two species. Which may be of interest, but is not the actual aim of the study.*

**Authors**: We did not use exactly the target-group method proposed by Phillips et al. 2009. We only based our biased background data on the reasoning of Phillips et al. 2009 about the necessity to account for bias in the selection of the background points according to presence data, but we did not considered a target-group species to select presence-absence data. We used the MaxEnt software to perform the models, which takes as input presence-only data and a set of environmental variables across a user-defined area, in our case, 10,000 isolated background points across whole Europe.

We clarified this in the new version of the ESM1 File. Now it reads: "Phillips et al. 2009, proposed to select background data that exhibits the same bias as the presence data. For example, if the presence data are taken from a determined portion of the study area, then the background data should be taken from the same areas (Ferrier et al., 2002; Phillips et al., 2009). Following this reasoning, we (i) created a grid comprising all native area (i.e., Europe), (ii) randomly selected 10,000 cells within <10 km of the species presence cells, and (iii) used 3,000 random background points from this selection to run the native-based models in MaxEnt."

Phillips SJ, Dudík M, Elith J, Graham CH, Lehmann A, Leathwick J, Ferrier S. Sample selection bias and presence‐only distribution models: implications for background and pseudo‐absence data. Ecol Appl. 2009; 19(1): 181−197.

Ferrier S, Watson G, Pearce J, Drielsma M. Extended statistical approaches to modelling spatial pattern in biodiversity in northeast New South Wales. I. Species-level modelling. Biodivers Conserv. 2002; 11(12): 2275−2307.

******
** References

Dungan, J.; Perry, J.; Dale, M.; Legendre, P.; Citron-Pousty, S.; Fortin, M.; Jakomulska, A.; Miriti, M. & Rosenberg, M. 2002. A balanced view of scale in spatial statistical analysis. Ecography 25, 626-640

Johnson, D. 1980. The comparison of usage and availability measurements for evaluating resource preference. Ecology 61, 65-71

Levin, S. 1992. The problem of pattern and scale in Ecology. Ecology 73, 1943-1967

Soberon, J. & Peterson, A. 2005. Interpretation of models of fundamental ecological niches and species' distributional areas. Biodiversity Informatics 2, 1-10

Pearce, J. & Boyce, M. 2006. Modelling distribution and abundance with presence-only data. Journal of Applied Ecology 43, 405-412

Elith, J. & Leathwick, J. R. 2009. Species distribution models: ecological explanation and prediction across space and time. Annual Review of Ecology, Evolution, and Systematics 40, 677

***Reviewer #2:***

*The authors have addressed the concerns expressed in the previous review. The explanation and map provided in Appendix S1 make the procedure much clearer and more transparent, and the dataset provided in appendix S2 adds significant value and usefulness to the manuscript. All other supplementary materials also help make the methodology clearer. Although not everything was done the way I would have done it myself, I am generally satisfied with the current version of the manuscript and I believe the authors have appropriately explained and defended their work.*

**Authors**: Thanks for your comment. We are pleased that you are satisfied with the manuscript.
